# Supplementary material for: Systematic review of the relationship between burn-out and spiritual health in doctors
Source: BMJ Open. 2023 Aug 8;13(8):e068402. doi: 10.1136/bmjopen-2022-068402 (PMC10414094; doi:10.1136/bmjopen-2022-068402)
Supplement: Supplementary data [file bmjopen-2022-068402supp001.pdf]

Embase (accessed via Ovid):

1. exp religion/ or religio*.mp. or religiosity.mp. or exp philosophy/ or exp spiritual healing/ or theology/ or sikh.mp. or jain.mp. or bahai.mp. or pagan.mp. or shinto.mp. or cao dai.mp. or zoroast*.mp. or spirit*.mp. or Faith.mp. or holistic health.mp. or existential.mp. or salutogenesis.mp. or spiritual*.mp. or religious personnel/ or chaplain*.mp. or Buddhism.mp. or Christian*.mp. or Hinduism.mp. or islam.mp. or Judaism.mp. or humanism.mp. or Confucian*.mp. or "sense of coherence".mp.

2. exp job stress/ or compassion fatigue.mp. or occupational stress.mp. or burnout.mp. or exp burnout/ or exp Maslach Burnout Inventory/ or "work related stress*".mp. or "occupational stress*".mp. or "work stress*".mp. or "job stress".mp. or Job Satisfaction/ or "job satisfaction".mp. or moral injury.mp. or burn&out.mp.

3. exp medical personnel/ or medical personnel.mp. or physician$.mp. or allergist$.mp. or cardiologists.mp. or dermatologists.mp. or endocrinologists.mp. or gastroenterologists.mp. or general practitioner$.mp. or geriatrician$.mp. or nephrologist$.mp. or neurologist$.mp. or occupational health physician$.mp. or oncologist$.mp. or ophthalmologist$.mp. or pathologist$.mp. or p$ediatrician$.mp. or neonatologist$.mp. or physiatrist$.mp. or pulmonologist$.mp. or psychistrist$.mp. or radiologist$.mp. or rheumatologist$.mp. or surgeon$.mp. or urologist$.mp. or doctor*.mp. or medical staff.mp. or an$esthetist$.mp. or medical school/ or medical faculty.mp.

Filter, from:
https://www.cochranelibrary.com/cdsr/doi/10.1002/14651858.MR000041.pub2/appendices

epidemiology/ or exp clinical study/ or cohort analysis/ or case study/ or follow up/ or cross-sectional study/ or observational study/ or correlational study/ or epidemiologic.ab,ti. Or case control.ab,ti. Or case referent.ab,ti. Or case stud$.ab,ti. Or case series.ab,ti. Or cohort?.ab,ti. Or cross sectional.ab,ti. Or follow up.ab,ti. Or longitudinal.ab,ti. Or retrospective$.ab,ti. Or prospective$.ab,ti. Or observational.ab,ti. Or adverse effect?.ab,ti. or (Controlled before and after).ab,ti. Or Interrupted time series.ab,ti. or Correlational.ab,ti. Or ecological stud$.ab,ti. or Descriptive stud$.ab,ti.

Not Editorial or letter

Psych info(accessed via Ovid)::

1. exp spirituality/ or exp religion/ or exp religiosity/ or exp religious beliefs/ or religious experiences/ or exp soul/ or exp spiritual care/ or religio*.mp. or religiosity.mp. or exp philosophy/ or exp faith/ or theology/ or sikh.mp. or jain.mp. or bahai.mp. or pagan.mp. or shinto.mp. or cao dai.mp. or zoroast*.mp. or spirit*.mp. or Faith.mp. or holistic health.mp. or existential.mp. or salutogenesis.mp. or spiritual*.mp. or chaplain*.mp. or Buddhism.mp. or Christian*.mp. or Hinduism.mp. or islam.mp. or Judaism.mp. or humanism.mp. or Confucian*.mp. or exp philosophies/ or exp religious personnel/ or god concepts/ or theology/ or "sense of coherence".mp.
2. exp occupational stress/ or occupational neurosis/ or "quality of work life"/ or exp moral injury/ or job satisfaction/ or compassion fatigue.mp. or occupational stress.mp. or burnout.mp. or "Maslach Burnout Inventory".mp. or "work related stress*".mp. or "occupational stress*".mp. or "work stress*".mp. or "job stress".mp. or "job satisfaction".mp. or moral injury.mp. or burn&out.mp.
3. exp medical personnel/ or medical personnel.mp. or physician$.mp. or allergist$.mp. or cardiologists.mp. or dermatologists.mp. or endocrinologists.mp. or gastroenterologists.mp. or general practitioner$.mp. or geriatrician$.mp. or nephrologist$.mp. or neurologist$.mp. or occupational health physician$.mp. or oncologist$.mp. or ophthalmologist$.mp. or pathologist$.mp. or p$ediatrician$.mp. or neonatologist$.mp. or physiatrist$.mp. or pulmonologist$.mp. or psychistrist$.mp. or radiologist$.mp. or rheumatologist$.mp. or surgeon$.mp. or urologist$.mp. or doctor*.mp. or medical staff.mp. or an$esthetist$.mp. or medical faculty.mp.
4.
(epidemiologic or case control or case referent or case stud$ or case series or cohort? or cross sectional or follow up or longitudinal or retrospective$ or prospective$ or observational or adverse effect? or (Controlled before and after) or Interrupted time series or Correlational or ecological stud$ or Descriptive stud$).ab,ti. Or exp experimental design or epidemiology/

Medline(accessed via Ovid)::

1. exp philosophy/ or exp religion/ or exp "religion and medicine"/ or exp "religion and psychology"/ or "religion and science"/ or exp religious philosophies/ or exp theology/ or religio*.mp. or sikh.mp. or jain.mp. or bahai.mp. or Humanism.mp. or pagan.mp. or shinto.mp. or cao dai.mp. or zoroast*.mp. or spirit*.mp. or faith.mp. or exp holistic health/ or existential.mp. or salutogenesis.mp. or exp Spiritual Therapies/ or religious personnel/ or holistic health/ or "sense of coherence"/

2.
exp occupational stress/ or compassion fatigue.mp. or occupational stress.mp. or burnout.mp. or caregiver burnout/ or exp burnout, psychological/ or "Maslach Burnout Inventory".mp. or "work related stress*".mp. or exp Occupational Stress/ or "occupational stress*".mp. or "work stress*".mp. or "job stress".mp. or Job Satisfaction/ or "job satisfaction".mp. or moral injury.mp. or "work-life balance"/ or burn&out.mp.

3.
anesthetists/ or anesthesiologists/ or faculty, medical/ or exp medical staff/ or exp physicians/ or medical personnel.mp. or physician$.mp. or allergist$.mp. or cardiologists.mp. or dermatologists.mp. or endocrinologists.mp. or gastroenterologists.mp. or general practitioner$.mp. or geriatrician$.mp. or nephrologist$.mp. or neurologist$.mp. or occupational health physician$.mp. or oncologist$.mp. or ophthalmologist$.mp. or pathologist$.mp. or p$ediatrician$.mp. or neonatologist$.mp. or physiatrist$.mp. or pulmonologist$.mp. or psychistrist$.mp. or radiologist$.mp. or rheumatologist$.mp. or surgeon$.mp. or urologist$.mp. or doctor*.mp. or medical staff.mp. or an$esthetist$.mp. or medical faculty.mp.

4. Epidemiologic Studies/ or exp Case Control Studies/ or exp Cohort Studies/ or Cross-Sectional Studies/ or (epidemiologic adj (study or studies)).ab,ti. Or case control.ab,ti. Or (cohort adj (study or studies)).ab,ti. Or cross sectional.ab,ti. Or cohort analy$.ab,ti. or (follow up adj (study or studies)).ab,ti. Or longitudinal.ab,ti. Or retrospective$.ab,ti. Or prospective$.ab,ti. Or (observ$ adj3 (study or studies)).ab,ti. Or adverse effect?.ab,ti.

5. editorial or letter

Web of science accessed via Clarivate:

1. TS=(religio* or philosop* or spirit* or theolog* or sikh or jain or bahai or pagan or shinto or "cao dai" or zoroast* or Faith or "holistic health" or existential or salutogenesis or spiritual* or chaplain* or Buddh* or Christian* or Hindu* or islam* or Juda* or humanism or Confucian* or "sense of coherence")
2. TS=(burnout or "compassion fatigue" or "Maslach Burnout Inventory" or "work related stress*" or "occupational stress*" or "work stress*" or "job stress" or "job satisfaction" or "moral injury" or burn&out)

3. (TS=("medical personnel" or physician$ or allergist$ or cardiologist$ or dermatologist$ or endocrinologist$ or gastroenterologist$ or general practitioner$ or geriatrician$ or nephrologist$ or neurologist$ or occupational health physician$ or oncologist$ or ophthalmologist$ or pathologist$ or p$ediatrician* or neonatologist$ or physiatrist$ or pulmonologist$ or psychistrist$ or radiologist$ or rheumatologist$ or surgeon$ or urologist$ or doctor* or "medical staff" or an$esthetist* or "medical school" or "medical faculty")
4. AND DOCUMENT TYPES: (Article OR Abstract of Published Item OR Correction OR Correction, Addition OR Data Paper OR Database Review OR Discussion OR Early Access OR Meeting Abstract OR Meeting Summary OR News Item OR Note OR Proceedings Paper OR Record Review OR Review OR Script)

Scopus accessed via https://www.scopus.com/home.uri:

1. Title-abs-key(religio* or philosop* or spirit* or theolog* or sikh or jain or bahai or pagan or shinto or "cao dai" or zoroast* or Faith or "holistic health" or existential or salutogenesis or spiritual* or chaplain* or Buddh* or Christian* or Hindu* or islam* or Juda* or humanism or Confucian* or "sense of coherence")
2. Title-abs-key(burnout or "compassion fatigue" or "Maslach Burnout Inventory" or "work related stress*" or "occupational stress*" or "work stress*" or "job stress" or "job satisfaction" or "moral injury" or burn&out)
3. Title-abs-key("medical personnel" or physician$ or allergist$ or cardiologist$ or dermatologist$ or endocrinologist$ or gastroenterologist$ or general practitioner$ or geriatrician$ or nephrologist$ or neurologist$ or occupational health physician$ or oncologist$ or ophthalmologist$ or pathologist$ or p$ediatrician* or neonatologist$ or physiatrist$ or pulmonologist$ or psychistrist$ or radiologist$ or rheumatologist$ or surgeon$ or urologist$ or doctor* or "medical staff" or an$esthetist* or "medical school" or "medical faculty")

All searches concluded by 8th March 2022
