# Supplementary material for: Systematic review of the relationship between burn-out and spiritual health in doctors
Source: BMJ Open. 2023 Aug 8;13(8):e068402. doi: 10.1136/bmjopen-2022-068402 (PMC10414094; doi:10.1136/bmjopen-2022-068402)
Supplement: Supplementary data [file bmjopen-2022-068402supp002.pdf]

Systematic Review Protocol & Support Template

**Title of the review**	A systematic review: Is there a relationship between burnout scores in doctors and their spiritual health
**First reviewer**	Orla Whitehead
**Team of reviewers**	Ishbel Orla Whitehead,<br>Suzanne Moffatt,<br>Stephanie Warwick,<br>Gemma Frances<br>Spiersa, Tafadzwa<br>Patience Kunonga,<br>Eugene Yee Hing Tang,<br>Barbara Hanratty
**Supervisor/Project PI**	Professor Barbara<br>Hanratty
**Clinical Portfolio Group**	
**Project title (if different from review title)**	

**Support** – please state if advice/training or personnel required at each stage	
**SR overview**	
**Protocol development**	“”
**Literature searching**	Advice taken from Catherine Richmond, information scientist at the University
**Quality appraisal**	
**Data Extraction**	“”
**Synthesis**	“”
**Writing up**	“”

Whitehead IO, *et al. BMJ Open* 2023; 13:e068402. doi: 10.1136/bmjopen-2022-068402

**1. Background to review**<br>Brief introduction to the subject of the review, including rationale for undertaking the review and overall aim
Burnout and moral injury are two terms used to describe a form of psychological distress associated with work as a doctor. Burnout is defined by the World Health Organisation not as an illness, but as an occupational phenomenon, resulting in exhaustion, mental distance or feelings of negativity or cynicism towards the job, and reduced professional efficacy.[1] Others describe a loss of meaning in work, and objectification of patients and their families, rather than engaging with their humanity.[2] Moral injury is a term taken from military personnel returning from battle, where the realties conflict with our moral and ethical code.[3] It has been argued that doctors caught between patient and societal demands, limited resources, and their own ethical code and training, are suffering injury analogous to soldiers, leaving them wounded and hopeless.[3] Reframing burnout as moral injury replaces a view of a defective individual, to that of a defective system, which could be more effective in terms of change[4], and move the dialogue away from expecting doctors to become more and more resilient in the face of increasing challenges. The co-option of the term from conflict has been critised[5], and arguments made that while moral injury can occur in medicine, burn out is not synonymous, and is more common.[6] While both the terms burnout and moral injury place the issue in the context of occupation, it is evident that mental illness accompanies these issues, as suicide rates for female doctors are up to 4 times those of age-matched controls.[7] With current recruitment and retention issues, we cannot afford to lose good doctors to burnout.[2] Supporting doctors' holistic health has to become a priority, to help prevent and treat burnout and moral injury.<br><br>Holistic health includes spiritual health. Literature discussing how doctors should address their patients' spiritual health often mention that self awareness of spiritual health, needs, and distress, is key to be able to begin to meet patients' needs in this area. In a recent survey of GPs in England, 69% described themselves as 'a spiritual person', with 48% also describing themselves as 'a religious person'. These GPs defined spiritual health as self-actualisation and meaning, transcendence and relationships beyond the self, and expressions of spirituality. Self-actualisation included concepts of being able to follow their personal ethical and moral codes. Meaning included having personal meaning to life and relationships. Transcendence included concepts of a spiritual sphere, a concept of a soul, and relationships with communities, friends, family, nature and/or the divine. Expressions of spirituality included both personal and group religious and spiritual practice, for example attendance at church, religious meetings, meditation, prayer and mindfulness practice. Burnout could be seen as contrasting with these definitions of spiritual health, as mental distance from others and the job, negativity and lack of meaning in the job, and lack of relationships with patients and their families. Spiritual health as defined by GPs requires meaning, following their ethical code. Could burnout and spiritual distress among doctors be related phenomena? Viewing burnout through the lens of spiritual health and distress, as well as other lenses and perspectives, could generate potential beneficial interventions for both prevention and treatment of burnout.<br><br>Aims:<br>To investigate the association between burnout in doctors and spiritual health, by conducting a systematic review of studies which use a measure burnout with measures of aspects of spiritual health, spirituality, religiosity or spiritual distress.

**2. Specific objectives**
1. To investigate the quantity and quality of data addressing doctors' burnout, and spiritual heath<br>2. To investigate any link between doctors' burnout and spiritual health, spirituality, spiritual distress or religiosity.

Whitehead IO, *et al. BMJ Open* 2023; 13:e068402. doi: 10.1136/bmjopen-2022-068402

**3. a) Criteria for including studies in the review**<br>If the PICOS format does not fit the research question of interest, please split up the question into separate concepts and put one under each heading	
**i. Population, or participants and conditions of interest**	Qualified doctors/medical practitioners, worldwide of either sex and any age, whether they have experienced any level of burnout or not.
**ii. Interventions or exposures**	Quantification of burnout (for example, use of a burnout inventory such as the Maslach Burnout Inventory, or the Copenhagen Burnout Inventory, or others)
**iii. Comparisons or control groups**	Quantification of spiritual health, spirituality, spiritual distress, or religiosity, either using a scale (for example the Spiritual Wellbeing Scale, Spiritual Distress Scale, or other measures of spiritual health or religiosity) or a yes/no or self-rating of 'spirituality' or religiosity.
**iv. Outcomes of interest**	Associations between spiritual health, spiritual distress or religiosity with burnout scores.
**v. Setting**	Any setting where doctors/medical practitioners work (primary, secondary or tertiary care, including laboratory or other non-patient facing settings)
**vi. Study designs**	All research studies with a quantification of burnout, and spiritual health, wellbeing, distress or religiosity, or similar.

**3. b) Criteria for excluding studies not covered in inclusion criteria**<br>Any specific populations excluded, date range, language, whether abstracts or full text available, etc
Medical students, nursing staff, or other hospital staff where doctors are not included.<br>Course evaluations<br>Editorials, book chapters, or other non-research study articles<br>No specific mention of spiritual health, spirituality, or religiosity.<br>No quantification of burnout, or spiritual health, wellbeing or distress, or similar.

**4. Search methods**	
**Electronic databases**<br>Please list all databases that are to be searched and include the interface (eg NHS, EBSCO, etc) and date ranges searched for each	PUBMED/MEDLINE<br>EMBASE<br>PsychInfo<br>Web of Science<br>Scopus
**Other methods used for identifying relevant research**<br>ie contacting experts and reference checking	Reference checking and hand searching of these. Contacting experts in this field.
**Journals hand searched**<br>If any are to be hand searched, please list which journals and date searched from, including a rationale.	

**5. Methods of review**	
**Details of methods**<br>Number of reviewers, how agreements to be reached and disagreements dealt with, etc.	Data extraction will be carried out by the primary reviewer, with 10% checked by a second reviewer
**Quality assessment**<br>Tools or checklists used with references or URLs	Appraisal of Cross-sectional Studies tool (AXIS).[8]

Whitehead IO, *et al. BMJ Open* 2023; 13:e068402. doi: 10.1136/bmjopen-2022-068402

**Data extraction** What information is to be collected on each included study. If databases or forms on Word or Excel are used and how this is recorded and by how many reviewers	Custom made word table to be used
**Narrative synthesis** Details of what and how synthesis will be done	Synthesis of findings regarding the research question (are burnout and spiritual health related)<br>Synthesis of measures used for burnout, and spiritual health
**Meta-analysis** Details of what and how analysis and testing will be done. If no meta-analysis is to be conducted, please give reason.	If possible, meta-analysis will be conducted of the relationship between burnout scores (e.g. the Maslach Burnout Inventory) and spiritual health scores (e.g. the Brief COPE). If different validated scores are used, effect sizes will be compared.
**Grading evidence** System used, if any, such as GRADE	

**6. Presentation of results**	
**Additional material** Summary tables, flowcharts, etc, to be included in the final paper	
**Outputs from review** Papers and target journals, conference presentations, reports, etc	Conference presentation at the Society for Academic Primary Care Annual Scientific Meeting<br>Submission to journal

**7. Timeline for review – when do you aim to complete each stage of the review**	
**Protocol**	July 2020
**Literature searching**	July 2020
**Quality appraisal**	August 2020
**Data extraction**	September 2020
**Synthesis**	September 2020
**Writing up**	October 2020

1. World Heath Organisation. Burn-out an "occupational phenomenon": International Classification of Diseases 2019 [Available from: https://www.who.int/mental_health/evidence/burn-out/en/.
2. Fred HL, Scheid MS. Physician Burnout: Causes, Consequences, and (?) Cures. *Tex Heart Inst J* 2018;45(4):198-202. doi: 10.14503/THIJ-18-6842
3. Talbot SG, Dean W. Physicians aren't 'burning out.'They're suffering from moral injury. *Stat* 2018
4. Dean W, Talbot S, Dean A. Reframing Clinician Distress: Moral Injury Not Burnout. *Fed Pract* 2019;36(9):400-02.
5. Asken MJ. Physician burnout: moral injury is a questionable term. *BMJ* 2019;365:l2375. doi: 10.1136/bmj.l2375
6. Parker J. Moral injury and burnout are not the same 2019 [Available from: https://blogs.bmj.com/bmj/2019/06/06/joshua-parker-moral-injury-and-burnout-are-not-the-same/ accessed 11/12/2019.
7. Practitioner Health Programme. Suicide in doctors and other health professionals 2019 [Available from: https://php.nhs.uk/resources/suicide-in-doctors/ accessed 11/12/2019.
8. Downes MJ, Brennan ML, Williams HC, et al. Development of a critical appraisal tool to assess the quality of cross-sectional studies (AXIS). *BMJ Open* 2016;6(12):e011458. doi: 10.1136/bmjopen-2016-011458
