# Supplementary material for: Systematic review of the relationship between burn-out and spiritual health in doctors
Source: BMJ Open. 2023 Aug 8;13(8):e068402. doi: 10.1136/bmjopen-2022-068402 (PMC10414094; doi:10.1136/bmjopen-2022-068402)
Supplement: Supplementary data [file bmjopen-2022-068402supp003.pdf]

Table 1: Findings from each study

Study	Comparisons of Burnout and Spiritual Health	Estimate or test statistic (95% confidence intervals in brackets, if given)	P value	The association of burnout (or similar concepts) with spiritual health (or similar concepts)
Alosaimi et al(1) 2018 Saudi Arabia	Perceived stress score was compared with use of religious coping (Brief-COPE)	Spearman's rho −0.09	0.030	Lower stress scores in those with higher religious coping
	Perceived stress score was compared for those who use religious coping (open ended question)	Number of participants:<br>Low PSS-66<br>Medium PSS- 48<br>High PSS- 33	0.004	
Antonsdottir et al(2) 2022 USA	Religion was compared with each domain of the MBI	DP β EE β PA β<br>Christian −2.73*** −4.97** 1.76*<br>Jewish −0.55 −1.82 0.03<br>Spiritual, not religious −2.35** −2.64 1.86<br>Other −2.58*** −3.59 1.53	*** p<0.001<br>**p<0.01<br>*p<0.05	Lower burnout scores in those with a religious/spirituality.
Azoulay(3) 2020 Europe	Anxiety and depression scores were compared with religiosity			Burnout score and religiosity not commented on.
Baruah(4) 2019 India	MBI compared with type of religion (no non-religious)	EE: χ2= 1.881	DP p=0.7579<br>EE p=0.3905<br>PA p=1.00	No association found
Ben-Itzak(5) 2015 Israel	The extent to which work provides meaning was associated with burnout scores	β=-3.144 (0.045- 6.24)	P<0.05	Higher work related meaning was associated with lower burnout scores.

Büssing et al(6) 2014 Germany	The aspects of spirituality domains were compared with the cool down index and perceived work burden	Aspects of spirituality domains / Cool down index / Perceived work burden<br>*Religious orientation* ρ=−0.078 ρ=−0.044<br>*Search for insight/wisdom* ρ=−0.107 ρ=−0.036<br>*Conscious interactions* ρ=−0.14* ρ=−0.165*<br>*Transcendence conviction* ρ=−0.106 ρ=−0.044	*P<0.01	No relationship- no statistical association between spiritual score and cool down reactions or perceived work burden
Chor et al(7) 2021 Singapore	55.8% used religious coping more than acts of gratitude from the public.			Burnout and religious coping use not compared.
Clark(8) 2007 USA	The statistical model chosen assumes a relationship between spirituality and job satisfaction via integration and self-actualisation	χ^2 0.614	p- 0.433	Higher job satisfaction was associated with higher spirituality
Correia et al(9) 2020 Portugal	Religiosity was compared with burnout score domains	Exhaustion- β= 0.07<br>Disengagement- β= 0.02		No association
Das(10) 2016 India	Religion was compared with burnout domains	EE- χ^2= 1.556<br>DP- χ^2= 2.917<br>PA- χ^2= 1.556	EE- p= 0.212<br>DP- p= 0.233<br>PA- p= 0.459	No association

Doolittle et al(11)2013<br>USA	Correlation between SIBS score and MBI	EE- r=-0.11<br>DP- r=-0.13<br>PA- r=0.28	EE- p=0.25<br>DP- p=0.17<br>PA- p=0.003	No statistical association
Doolittle(12) 2020<br>USA	Three domains from the Durel are reported as Pearson's correlation coefficients	EE / DP / PA<br>Organized religion: − 0.12 / − 0.21 / + 0.14<br>Private practices: − 0.21 / − 0.12 / + 0.23<br>Religiosity: − 0.18 / − 0.18 / + 0.20		No association
Doolittle and Windish(13) 2015<br>USA	Spearman's rank correlation coefficient	EE- 'not significant'<br>DP- 'not significant'<br>PA- 0.35	PA- p=0.03	No association
Frank(14) 1999<br>USA	Religious fervour was compared with desire to become a physician again	Odds ratio- 1.3 (1.1-1.6)		No direct association found
Glasberg et al(15) 2007<br>Brazil	Religious identity was compared with MBI scores		p=0.038	Higher burnout in the non-religious
Glebocka et al(16) 2007<br>Poland	Hobfall's gains and losses were compared with MBI scores (translated), correlation coefficients reported	EE / DP / Professional Cynicism<br>Spiritual resources gain: 0.14 / 0.13 / 0.29<br>Spiritual resources loss: 0.09 / 0.35* / -0.06	*p<0.05	Positive correlation between burnout and spiritual resource loss.
Gribben et al(17) 2019<br>USA	Compares prayer/meditation and compassion fatigue	β=0.12	p<0.001	Higher compassion fatigue in those using spiritual type behaviours

Guay et al(18) 2019 Americas	Number of participants with burnout compared by religion.	χ^2 values not given	Religious affiliation p= 0.184 Importance of spirituality p=0.984 Importance of religion p=0.084	No association
Guest et al(19) 2011 USA	Spirituality mentioned as protective factor against burnout	Numeric data not given		MBI and spirituality not compared
Harper et al(20) 2020 USA	Believer/non-believers MBI scores compared	χ^2 values not given	p=0.69	No association
Koh et al(21) 2015 Singapore	The odds of being burned out and answering 'no' to being spiritual were compared.	odds ratios:<br>High DP- 2.83 (1.2-6.3)<br>Low PA- 2.85 (1.4-6.0)<br>High EE- 1.67 (0.76-3.64)<br>Overall burnout- 1.80 (0.86-3.71)		Those who answered 'no' to the spiritual question had lower personal accomplishment
Lal et al(22) 2020 India	MBI domains were compared with whether religious or spiritual beliefs influence work	ANOVA values not given	PA p=0.002	No overall association
Leonelli et al(23) 2017 Brazil	Perceived stress was compared with religious activity	β= 3.1 [0.3- 5.8]	p=0.03	Lower religious activity associated with higher perceived stress
Leu et al(24) 2020 Switzerland and Australia	Atheism vs any religion was compared with burnout	β= -52.5	p=0.13	No association

Macuka et al(25) 2020 Croatia	Compares spiritual question and burnout scores	EE- β= -0.20<br>r=-0.19<br>Disengagement- β=-0.18<br>r=-0.19	p<0.05<br>p<0.05<br>p<0.05<br>p<0.05	Religiosity was associated with lower emotional exhaustion.
Macuka et al 2021(26) Croatia	Spiritual question and job satisfaction were compared	r=0.15	p>0.05	Higher job satisfaction in those with higher religiosity
Mantri et al 2021(27) USA	Compares religiosity and moral injury scores	data not given		Moral injury symptoms and burnout inversely related to religiosity.
McKinley et al 2020(28) full data from Dr McKinley's thesis(29)	Religious coping was compared with burnout score	Unadjusted β= -0.42	p<0.01	Higher religious coping associated with lower burnout
Ntantana et al(30) 2017 Greece	Spirituality and burnout were compared, only EE reported	OR for high burnout given high EE- 1.482 (0.823-2.670)		No association
Purviss et al(31) 2019 USA	Religion (Catholics vs other) was compared with burnout score.		p = 0.026	No association
Ramondetta et al(32) 2011 Greece	Correlation between religiosity and Work Related Strain Inventory (WRSI)	Duke religiosity index and WRSI r=0.08<br>Intrinsic religiosity scale and WRSI r=0.05		No association
Roslan et al(33) 2021 Malaysia	Irregular spirituality routines were compared with burnout scores	OR- 2.24 (1.49–3.37))	p<0.001	Work related burnout was associated with irregular spirituality routines

Salmoirago-Blotcher(34) 2016 USA	Various religious/spiritual questions were compared with burnout scores	**Organized religiosity**	**Adjusted OR**			No association
		Never	Ref.			
		Any	0.89 (0.36 to 2.20)			
		**Religious Affiliation**				
		None	Ref.			
		Any	0.51 (0.19 to 1.39)			
		Private prayer				
		Never	Ref.			
		Any	0.66 (0.27 to 1.60)			
		Meditation				
		Never	Ref.			
		Any	0.56 (0.19 to 1.62)			
		**Self-rated spirituality**				
		Slightly/not spiritual	Ref.			
		Moderately spiritual	1.14 (0.45 to 2.88)			
		Very spiritual	0.15 (0.02 to 1.46)			
		**Religious rest**				
		Never	Ref.			
		Any	0.51 (0.16 to 1.65)			
		"I try hard to carry my religious beliefs over into all my life"				

		Disagree Ref.<br>Agree 0.70 (0.28 to 1.74)		
Schmidt and Roffler(35) 2021<br><br>Quantitative data given by author, unpublished(36)	Correlation between spiritual score and burnout score	DP ρ= -0.37<br>EE ρ= -0.55<br>PA ρ= 0.62	p<0.05<br>p<0.05<br>p<0.05	Association between all domains of the burnout score and the spiritual score.
See et al(37) 2018<br>Large study, across Asia	Compared religious background or belief and high burnout	OR 0.69 (0.49–0.96)	P- 0.027	Religiosity was found to be protective against burnout in medical doctors (and nurses).
Shetach et al(38) 2015<br>Israel	Job satisfaction scores for Christians were compared with non-Christians	Religion β<br>Christian 0.46<br>Non-Christian 0.41	p>0.05	No association
St Onge et al(39) 2022<br>USA	Spiritual question was compared with burnout scores	Personal burnout β= -0.77<br>Work burnout β= -1.87885<br>Patient burnout β= −1.65<br><br>Odds ratio for overall burnout: 0.55(0.23-1.31)	p>0.05<br>p>0.05<br>p>0.05<br><br>p= 0.18	No association
Suttle et al(40) 2020<br>Asia	Mean MBI scores were compared with yes/no to spiritual question	Mean<br>EE- 26.77<br>DP- 11.278<br>PA-36.620	 p= 0.036<br>p= 0.011<br>p= 0.021	Higher burnout scores were associated with identifying as non-spiritual.
Teixeira et al(41) 2013<br>Portugal	Burnout scores in the religious were compared to non-religious	Odds ratio for burnout: 0.907	p=0.747	No association

Watson(42)<br>2019<br>USA	Mean burnout domain scores were compared with religious/spiritual question	Spirituality/religion<br>Mean EE<br>Mean DP<br>Mean PA	Disagree<br>3.98<br>3.95<br>5.31	Neutral<br>3.80<br>3.71<br>5.87	Agree<br>3.41<br>3.18<br>5.65	 p=0.36<br>p=0.13<br>p=0.43	Higher emotional exhaustion and lower personal accomplishment were correlated with those who disagreed with the spiritual question. No correlation with depersonalization. One-way ANOVA showed no difference between means.

MBI- Maslach Burnout Inventory DP- Depersonalisation EE- Emotional Exhaustion PA- Personal Accomplishment
SIBS- Hatch Spiritual Involvement and Beliefs Scale WRSI- Work Related Strain Inventory
Brief COPE- Brief Coping Orientation to Problems Experienced Inventory

1. Alosaimi FD, Alawad HS, Alamri AK, Saeed AI, Aljuaydi KA, Alotaibi AS, et al. Stress and coping among consultant physicians working in Saudi Arabia. Annals of Saudi Medicine. 2018;38(3):214-24.
2. Antonsdottir I, Rushton CH, Nelson KE, Heinze KE, Swoboda SM, Hanson GC. Burnout and moral resilience in interdisciplinary healthcare professionals. Journal of Clinical Nursing. 2022;31(1):196-208.
3. Azoulay E, De Waele J, Ferrer R, Staudinger T, Borkowska M, Povoa P, et al. Symptoms of burnout in intensive care unit specialists facing the COVID-19 outbreak. Annals of Intensive Care. 2020;10(1):110.
4. Baruah A, Das S, Dutta A, Das B, Sharma T, Hazarika M. DEGREE AND FACTORS OF BURNOUT AMONG EMERGENCY HEALTHCARE WORKERS IN INDIA. Int J Sci Res (Ahmedabad). 2019;8(4):41-5.
5. Ben-Itzhak S, Dvash J, Maor M, Rosenberg N, Halpern P. Sense of meaning as a predictor of burnout in emergency physicians in Israel: a national survey. Clinical and Experimental Emergency Medicine. 2015;2(4):217-25.
6. Büssing A, Lotzke D, Glockler M, Heusser P. Influence of Spirituality on Cool Down Reactions, Work Engagement, and Life Satisfaction in Anthroposophic Health Care Professionals. Evidence-Based Complementary and Alternative Medicine. 2015.
7. Chor WPD, Ng WM, Cheng L, Situ W, Chong JW, Ng LYA, et al. Burnout amongst emergency healthcare workers during the COVID-19 pandemic: A multi-center study. American Journal of Emergency Medicine. 2021;46:700-2.
8. Clark L, Leedy S, McDonald L, Muller B, Lamb C, Mendez T, et al. Spirituality and job satisfaction among hospice interdisciplinary team members. Journal of Palliative Medicine. 2007;10(6):1321-8.
9. Correia I, Almeida AE. Organizational Justice, Professional Identification, Empathy, and Meaningful Work During COVID-19 Pandemic: Are They Burnout Protectors in Physicians and Nurses? FRONTIERS IN PSYCHOLOGY. 2020;11.
10. Das S, Barman S, Datta S, Bardhan N, Baishya M, Das B, et al. Degree of burnout among emergency healthcare workers and factors influencing level of burnout: a pilot study. Delhi Psychiatry Journal. 2016;19:36-47.
11. Doolittle BR, Windish DM, Seelig CB. Burnout, coping, and spirituality among internal medicine resident physicians. Journal of graduate medical education. 2013;5(2):257-61.

12. Doolittle BR. Association of Burnout with Emotional Coping Strategies, Friendship, and Institutional Support Among Internal Medicine Physicians. Journal of Clinical Psychology in Medical Settings. 2020.
13. Doolittle BR, Windish DM. Correlation of burnout syndrome with specific coping strategies, behaviors, and spiritual attitudes among interns at Yale University, New Haven, USA. Journal of Educational Evaluation for Health Professions. 2015;12.
14. Frank E, McMurray J, Linzer M, Elon L. Career satisfaction of US women physicians - Results from the Women Physicians' Health Study. ARCHIVES OF INTERNAL MEDICINE. 1999;159(13):1417-26.
15. Glasberg J, Horiuti L, Novais M, Canavezzi A, Miranda V, Chicoli F, et al. Prevalence of the burnout syndrome among Brazilian medical oncologists. REVISTA DA ASSOCIACAO MEDICA BRASILEIRA. 2007;53(1):85-9.
16. Glebocka A, Lisowska E, Glebocka A, Lisowska E. Professional burnout and stress among polish physicians explained by the Hobfoll resources theory. JOURNAL OF PHYSIOLOGY AND PHARMACOLOGY. 2007;58:243-52.
17. Gribben J, Kase S, Waldman E, Weintraub A, Gribben JL, Kase SM, et al. A Cross-Sectional Analysis of Compassion Fatigue, Burnout, and Compassion Satisfaction in Pediatric Critical Care Physicians in the United States*. PEDIATRIC CRITICAL CARE MEDICINE. 2019;20(3):213-22.
18. Guay MD, Reyes Donoso MM, Lopez Saca JM, Riveros Rios ME, Pastrana T. Spirituality and Religiosity and Burnout in Latin-American Palliative Care Health Care Professionals (LAPC) (S818). Journal of Pain and Symptom Management. 2019;57(2):490-1.
19. Guest RS, Baser R, Li Y, Scardino PT, Brown AE, Kissane DW. Cancer surgeons' distress and well-being, II: modifiable factors and the potential for organizational interventions. Annals of surgical oncology. 2011;18(5):1236-42.
20. Harper L, Alshammari D, Ferdynus C, Kalfa N. Burnout amongst members of the French-speaking Society of Pediatric and Adolescent Urology (SFUPA). Are there specific risk factors? Journal of pediatric urology. 2020;16(4):482-6.
21. Koh MYH, Chong PH, Neo PSH, Ong YJ, Yong WC, Ong WY, et al. Burnout, psychological morbidity and use of coping mechanisms among palliative care practitioners: A multi-centre cross-sectional study. Palliative Medicine. 2015;29(7):633-42.
22. Lal A, Tharyan A, Tharyan P. The prevalence, determinants and the role of empathy and religious or spiritual beliefs on job stress, job satisfaction, coping, burnout, and mental health in medical and surgical faculty of a teaching hospital: A cross-sectional survey. La Revue de Médecine Interne. 2020.
23. Leonelli LB, Andreoni S, Martins P, Kozasa EH, Salvo VLd, Sopezki D, et al. Perceived stress among Primary Health Care Professionals in Brazil. Estresse percebido em profissionais da Estrategia Saude da Familia. 2017;20(2):286-98.
24. Leu S, Vuille-dit-Bille R, Fink L, Soll C, Staerkle R, Leu S, et al. Burnout in Swiss and Australian surgeons in training-a cross-sectional study. EUROPEAN SURGERY-ACTA CHIRURGICA AUSTRIACA.
25. Macuka I, Junakovic I, Bozic D, Macuka I, Junakovic IT, Bozic D. Burnout among Palliative Care Professionals. DRUSTVENA ISTRAZIVANJA. 2020;29(2):287-308.
26. Macuka I, Tucak Junakovic I. A Cross-Sectional Study of Job Satisfaction and Intention to Leave Job in Palliative Care in Croatia. Journal of palliative care. 2021:8258597211046704.
27. Mantri S, Lawson JM, Wang ZZ, Koenig HG. Prevalence and Predictors of Moral Injury Symptoms in Health Care Professionals. JOURNAL OF NERVOUS AND MENTAL DISEASE. 2021;209(3):174-80.
28. McKinley N, McCain RS, Convie L, Clarke M, Dempster M, Campbell WJ, et al. Resilience, burnout and coping mechanisms in UK doctors: a cross-sectional study. BMJ Open. 2020;10(1):e031765.
29. McKinley NC. Resilience, professional quality of life and coping mechanisms in doctors and medical students.: Queens University Belfast; 2021.
30. Ntantana A, Matamis D, Savvidou S, Giannakou M, Gouva M, Nakos G, et al. Burnout and job satisfaction of intensive care personnel and the relationship with personality and religious traits: An observational, multicenter, cross-sectional study. Intensive and Critical Care Nursing. 2017;41:11-7.

31. Purvis T, Saylor D, Powell B, Biba G, Conti D, Crowe T, et al. Burnout and Resilience Among Neurosciences Critical Care Unit Staff. NEUROCRITICAL CARE. 2019;31(2):406-10.
32. Ramondetta L, Urbauer D, Brown A, Richardson G, Thaker P, Koenig H, et al. Work related stress among gynecologic oncologists. GYNECOLOGIC ONCOLOGY. 2011;123(2):365-9.
33. Roslan NS, Yusoff MSB, Ab Razak A, Morgan K, Shauki NIA, Kukreja A, et al. Training Characteristics, Personal Factors and Coping Strategies Associated with Burnout in Junior Doctors: A Multi-Center Study. HEALTHCARE. 2021;9(9).
34. Salmoirago-Blotcher E, Fitchett G, Leung K, Volturo G, Boudreaux E, Crawford S, et al. An exploration of the role of religion/spirituality in the promotion of physicians' wellbeing in Emergency Medicine. Preventive Medicine Reports. 2016;3:189-95.
35. Schmidt C, Roffler M. Coping with the Practice of Medicine: Religion, Spirituality, and Other Personal Strategies. JOURNAL OF RELIGION & HEALTH. 2021;60(3):2092-108.
36. Schmidt C, Roffler M. Who isn't burned out? The role of spirituality in graduate medical education (GME). 2018.
37. See K, Zhao M, Nakataki E, Chittawatanarat K, Fang W, Faruq M, et al. Professional burnout among physicians and nurses in Asian intensive care units: a multinational survey. INTENSIVE CARE MEDICINE. 2018;44(12):2079-90.
38. Shetach A, Marcus O, Shetach A, Marcus O. The critical managerial capabilities of medical and nursing managers in an Israeli hospital. EVIDENCE-BASED HRM-A GLOBAL FORUM FOR EMPIRICAL SCHOLARSHIP. 2015;3(1):81-102.
39. St Onge JE, Allespach H, Diaz Y, Poitier A, Tamariz L, Paidas C, et al. Burnout: exploring the differences between U.S. and international medical graduates. BMC medical education. 2022;22(1):69.
40. Suttle M, Chase M, Sasser W, Moore-Clingenpeel M, Maa T, Werner J, et al. Burnout in Pediatric Critical Care Medicine Fellows*. CRITICAL CARE MEDICINE. 2020;48(6):872-80.
41. Teixeira C, Ribeiro O, Fonseca AM, Carvalho AS. Burnout in intensive care units - a consideration of the possible prevalence and frequency of new risk factors: A descriptive correlational multicentre study. BMC Anesthesiology. 2013;13:38.
42. Watson AG, Saggar V, MacDowell C, McCoy JV. Self-reported modifying effects of resilience factors on perceptions of workload, patient outcomes, and burnout in physician-attendees of an international emergency medicine conference. Psychology, health & medicine. 2019;24(10):1220-34.
