# Supplementary material for: Systematic review of the relationship between burn-out and spiritual health in doctors
Source: BMJ Open. 2023 Aug 8;13(8):e068402. doi: 10.1136/bmjopen-2022-068402 (PMC10414094; doi:10.1136/bmjopen-2022-068402)
Supplement: Supplementary data [file bmjopen-2022-068402supp004.pdf]

Table 2: Study details, AXIS quality assessment, spiritual wellness assessment and burnout score used

Study	Study design	AXIS Quality rating	Population	Measure of spirituality	Measure of burnout
Alosaimi et al(1) 2018 Saudi Arabia	Cross-sectional study	16	582 consultant physicians	Brief COPE (2 items to score religious coping)	Perceived stress Scale
Antonsdottir et al(2) 2022 USA	Cross-sectional study	12	639 (96 physicians)	Religious affiliation (including ‘spiritual but not religious”)	MBI
Azoulay(3) 2020 Europe	Cross-sectional study	12	1132 respondents- ICU specialists	Self-rated religiosity 1-100	MBI
Baruah(4) 2019 India	Cross-sectional study	15	68 participants (doctors, nurses, and paramedics)- Emergency Department	Religion	MBI
Ben-Itzak(5) 2015 Israel	Cross-sectional study	10	70 emergency physicians	“My job gives me meaning” yes/no<br>Open ended questions: 1. Self-fulfilment<br>2. Meaningful relationships<br>3. Excitement and adrenaline rush<br>4. Providing meaningful assistance.	MBI
Büssing et al(6) 2014 Germany	Cross-sectional study	16	489 anthroposophic medical staff (41% physicians)	Aspects of Spirituality Questionnaire (ASQ)	Cool down Index<br>Utrecht Work engagement scale.

Chor et al(7) 2021 Singapore	Cross-sectional study	14	337 participants (127 doctors) Emergency department	Use of religious coping	Copenhagen Burnout Inventory
Clark(8) 2007 USA	Cross-sectional study	14	215 hospice staff, 2.3% physicians	Jarel spiritual wellbeing scale	8-item job satisfaction scale
Correia et al(9) 2020 Portugal	Cross-sectional study	12	497 health professionals working during COVID-19 (229 physicians)	"Regardless of whether you belong to a particular religion, how religious would you say you are?"	Oldenburg Burnout Inventory
Das(10) 2016 India	Cross-sectional study	12	7 emergency department participants (4 doctors)	Religion	MBI
Doolittle et al(11)2013 USA	Cross-sectional study	17	108 hospital residents	Hatch Spiritual Involvement and Beliefs Scale (SIBS), chosen to try and avoid cultural bias.	MBI
Doolittle(12) 2020 USA	Cross-sectional study	17	337 internal medicine physicians	COPE and DUREL, self reported spiritual and/or religious	ProQoL (domains of burnout, secondary stress, and compassion satisfaction)
Doolittle and Windish(13) 2015 USA	Cross-sectional study	15	Interns 66 in the summer, and 53 in winter cohorts	Coping Orientation to Problems Experienced (COPE), Hatch SIBS, self perception of spirituality and religiosity.	MBI

Frank(14) 1999 USA	Cross-sectional study	17	4501 female physicians	"Religious identification" none, low strong or very strong	"Are you generally satisfied with your career?" "If you lived your life again, would you become a physician?"
Glasberg et al(15) 2007 Brazil	Cross-sectional study	16	102 oncologists	Religious affiliation	MBI
Glebocka et al(16) 2007 Poland	Cross-sectional study	8	77 people (41 doctors)	Hobfoll's Questionnaire of Self-Assessment of Gains and Losses)7 statements of 'spiritual resources' gained in last 12 months)	MBI
Gribben et al(17) 2019 USA	Cross-sectional study	18	390 paediatric emergency medicine trainees	Prayer/meditation as self care activity	Compassion Fatigue and Satisfaction Self-Test for Helpers (adapted)
Guay et al(18) 2019 Americas	Cross-sectional study	8	221 (RR 63%) health care professions, 58.8% physicians,	Importance of religion<br>Importance of spirituality<br>Self-definition as religious person<br>Self-definition as spiritual person<br>Frequency of religious practice (praying/meditation)	Respondents categorised as burned out if they agreed with: ''I am definitely burning out and have one or more symptoms of burnout, such as physical and emotional exhaustion,'' ''The symptoms of burnout that I'm experiencing won't go away. I think about frustration at work a lot,'' ''I feel completely burned out and often wonder if I can go on. I am at the point where I may need some changes or may need to seek some sort of help.''<br><br>Respondents were categorized as not burned out if they agreed to either of the statements: (1) ''I am definitely burning out and have one or more symptoms of burnout, such as physical and emotional exhaustion, (2) Occasionally I am under stress, and I don't always have as much energy as I once did, but I don't feel burned out.''

Guest et al(19) 2011 USA	Cross-sectional study	10	72 cancer surgeons	Linear Analog Scale Assessment of Quality of Life (LASA QOL) which includes spiritual QoL	MBI
Harper et al(20) 2020 USA	Cross-sectional study	18	70 Urologists	Believer/non-believer	MBI
Koh et al(21) 2015 Singapore	Cross-sectional study	19	273 palliative care staff (28.1% doctors)	Single question: “Do you consider yourself spiritual?”	MBI
Lal et al(22) 2020 India	Cross-sectional study	19	345 of medical and surgical faculty	Single question- “My religious or spiritual beliefs strongly influence my approach to my work and patients.”	MBI<br>Job stress and job satisfaction questionnaire<br>Jefferson scale of physician empathy
Leonelli et al(23) 2017 Brazil	Cross-sectional study	19	450 primary health care professionals (27 doctors)	Religious activity	Perceived stress scale (PSS)continuous
Leu et al(24) 2020 Switzerland and Australia	Cross-sectional study	16	412 participants (237 Swiss and 175 Australian)	Religion	MBI
Macuka et al(25) 2020 Croatia	Cross-sectional study	12	123 palliative care staff (18 doctors)	“Whether or not you consider yourself a believer or not, how much of a spiritual person you consider yourself, that is, how much you are interested in the sacred or the supernatural?” likert scale 1-5	Oldenburg burnout inventory
Macuka et al 2021(26) Croatia		9			
Mantri et al 2021(27) USA	Cross-sectional study	18	181 participants	Religious affiliation, and ‘religious commitment’ using Belief into Action (BIAC) scale	Moral Injury Symptom Scale-Healthcare Professionals version and MBI

McKinley et al 2020(28) full data from Dr McKinley's thesis(29)	Cross-sectional study	17	1382 participants, 69% hospital, 31% primary care	BRIEF COPE, includes religious coping	ProQOL-V includes burnout, compassion satisfaction and secondary traumatic stress sub domains.
Ntantana et al(30) 2017 Greece	Cross-sectional study	18	149 physicians, 320 nurses from intensive care	Religious and Spiritual attitudes questionnaire(31), designed by Büssing for use with patients, rather than staff. The authors report it was chosen as it avoids exclusive religious terminology, however in the paper by Büssing referred to, they report that a positive attitude towards religion and spirituality is necessary for some aspects of the scale.	MBI
Purviss et al(32) 2019 USA	Cross-sectional study	16	64 neurosciences critical care staff (10 doctors)	Religion	MBI
Ramondetta et al(33) 2011 Greece	Cross-sectional study	15	273 oncologists	Religious affiliation, self-rated religiosity and spirituality, Intrinsic Religiosity Survey, and Duke religion Index (DRI- a measure organisational religiosity)	Work related strain inventory (WRSI)- correlates with MBI
Roslan et al(34) 2021 Malaysia	Cross-sectional study	20	754 hospital interns	BRIEF COPE, includes religious coping, and descriptors of regular versus irregular spirituality routines based on several major religions professed in the country.	Copenhagen Burnout Inventory
Salmoirago-Blotcher(35) 2016 USA	Cross-sectional study	16	138 emergency physicians	Religious affiliation, Self-rated spirituality, Fetzer Institute Multidimensional Measurement of Religiousness/Spirituality for Use in Health Research- used to assess religious/spiritual practice, and religious commitment ("I try hard to carry my religious beliefs over into all my other dealings in life), questions added to assess religious rest, and spiritual counsel.	2 item MBI

Schmidt and Roffler(36) 2021<br><br>Quantitative data given by author, unpublished(37)	Cross-sectional study	10	65 medical education faculty	The Functional Assessment of Chronic Illness Therapy-Spiritual Well-Being Scale, a modified version for non-illness (FACIT-Sp-Non-Illness)	MBI
See et al(38) 2018<br>Large study, across Asia	Cross-sectional study	16	992 intensive care physicians	Religious background or belief	MBI
Shetach et al(39) 2015<br>Israel	Cross-sectional study	13	107 respondents (40 doctors)	Religion- (Christian vs non-Christian)	Job satisfaction questionnaire
St Onge et al(40) 2022<br>USA	Cross-sectional study	17	222 interns	"My spiritual beliefs are very important in helping me cope with stressors."	Copenhagen Burnout Inventory
Suttle et al(41) 2020<br>Asia	Cross-sectional study	12	47 programme directors and 187 fellows in paediatric critical care	"Do you consider yourself to be a spiritual person?" yes/no	MBI
Teixeira et al(42) 2013<br>Portugal	Cross-sectional study	14	300 Intensive Care staff (82 physicians)	Religion yes/no	MBI
Watson(43) 2019<br>USA	Cross-sectional study	13	54 emergency physicians	Single question- "spirituality/religion is important in my life"	MBI

MBI-Maslach Burnout Inventory

1. Alosaimi FD, Alawad HS, Alamri AK, Saeed AI, Aljuaydi KA, Alotaibi AS, et al. Stress and coping among consultant physicians working in Saudi Arabia. Annals of Saudi Medicine. 2018;38(3):214-24.
2. Antonsdottir I, Rushton CH, Nelson KE, Heinze KE, Swoboda SM, Hanson GC. Burnout and moral resilience in interdisciplinary healthcare professionals. Journal of Clinical Nursing. 2022;31(1):196-208.
3. Azoulay E, De Waele J, Ferrer R, Staudinger T, Borkowska M, Povoa P, et al. Symptoms of burnout in intensive care unit specialists facing the COVID-19 outbreak. Annals of Intensive Care. 2020;10(1):110.
4. Baruah A, Das S, Dutta A, Das B, Sharma T, Hazarika M. DEGREE AND FACTORS OF BURNOUT AMONG EMERGENCY HEALTHCARE WORKERS IN INDIA. Int J Sci Res (Ahmedabad). 2019;8(4):41-5.
5. Ben-Itzhak S, Dvash J, Maor M, Rosenberg N, Halpern P. Sense of meaning as a predictor of burnout in emergency physicians in Israel: a national survey. Clinical and Experimental Emergency Medicine. 2015;2(4):217-25.
6. Büssing A, Lotzke D, Glockler M, Heusser P. Influence of Spirituality on Cool Down Reactions, Work Engagement, and Life Satisfaction in Anthroposophic Health Care Professionals. Evidence-Based Complementary and Alternative Medicine. 2015.
7. Chor WPD, Ng WM, Cheng L, Situ W, Chong JW, Ng LYA, et al. Burnout amongst emergency healthcare workers during the COVID-19 pandemic: A multi-center study. American Journal of Emergency Medicine. 2021;46:700-2.
8. Clark L, Leedy S, McDonald L, Muller B, Lamb C, Mendez T, et al. Spirituality and job satisfaction among hospice interdisciplinary team members. Journal of Palliative Medicine. 2007;10(6):1321-8.
9. Correia I, Almeida AE. Organizational Justice, Professional Identification, Empathy, and Meaningful Work During COVID-19 Pandemic: Are They Burnout Protectors in Physicians and Nurses? FRONTIERS IN PSYCHOLOGY. 2020;11.
10. Das S, Barman S, Datta S, Bardhan N, Baishya M, Das B, et al. Degree of burnout among emergency healthcare workers and factors influencing level of burnout: a pilot study. Delhi Psychiatry Journal. 2016;19:36-47.
11. Doolittle BR, Windish DM, Seelig CB. Burnout, coping, and spirituality among internal medicine resident physicians. Journal of graduate medical education. 2013;5(2):257-61.
12. Doolittle BR. Association of Burnout with Emotional Coping Strategies, Friendship, and Institutional Support Among Internal Medicine Physicians. Journal of Clinical Psychology in Medical Settings. 2020.
13. Doolittle BR, Windish DM. Correlation of burnout syndrome with specific coping strategies, behaviors, and spiritual attitudes among interns at Yale University, New Haven, USA. Journal of Educational Evaluation for Health Professions. 2015;12.
14. Frank E, McMurray J, Linzer M, Elon L. Career satisfaction of US women physicians - Results from the Women Physicians' Health Study. ARCHIVES OF INTERNAL MEDICINE. 1999;159(13):1417-26.
15. Glasberg J, Horiuti L, Novais M, Canavezzi A, Miranda V, Chicoli F, et al. Prevalence of the burnout syndrome among Brazilian medical oncologists. REVISTA DA ASSOCIACAO MEDICA BRASILEIRA. 2007;53(1):85-9.
16. Glebocka A, Lisowska E, Glebocka A, Lisowska E. Professional burnout and stress among polish physicians explained by the Hobfoll resources theory. JOURNAL OF PHYSIOLOGY AND PHARMACOLOGY. 2007;58:243-52.
17. Gribben J, Kase S, Waldman E, Weintraub A, Gribben JL, Kase SM, et al. A Cross-Sectional Analysis of Compassion Fatigue, Burnout, and Compassion Satisfaction in Pediatric Critical Care Physicians in the United States*. PEDIATRIC CRITICAL CARE MEDICINE. 2019;20(3):213-22.
18. Guay MD, Reyes Donoso MM, Lopez Saca JM, Riveros Rios ME, Pastrana T. Spirituality and Religiosity and Burnout in Latin-American Palliative Care Health Care Professionals (LAPC) (S818). Journal of Pain and Symptom Management. 2019;57(2):490-1.

19. Guest RS, Baser R, Li Y, Scardino PT, Brown AE, Kissane DW. Cancer surgeons' distress and well-being, II: modifiable factors and the potential for organizational interventions. Annals of surgical oncology. 2011;18(5):1236-42.
20. Harper L, Alshammari D, Ferdynus C, Kalfa N. Burnout amongst members of the French-speaking Society of Pediatric and Adolescent Urology (SFUPA). Are there specific risk factors? Journal of pediatric urology. 2020;16(4):482-6.
21. Koh MYH, Chong PH, Neo PSH, Ong YJ, Yong WC, Ong WY, et al. Burnout, psychological morbidity and use of coping mechanisms among palliative care practitioners: A multi-centre cross-sectional study. Palliative Medicine. 2015;29(7):633-42.
22. Lal A, Tharyan A, Tharyan P. The prevalence, determinants and the role of empathy and religious or spiritual beliefs on job stress, job satisfaction, coping, burnout, and mental health in medical and surgical faculty of a teaching hospital: A cross-sectional survey. La Revue de Médecine Interne. 2020.
23. Leonelli LB, Andreoni S, Martins P, Kozasa EH, Salvo VLd, Sopezki D, et al. Perceived stress among Primary Health Care Professionals in Brazil. Estresse percebido em profissionais da Estrategia Saude da Familia. 2017;20(2):286-98.
24. Leu S, Vuille-dit-Bille R, Fink L, Soll C, Staerkle R, Leu S, et al. Burnout in Swiss and Australian surgeons in training-a cross-sectional study. EUROPEAN SURGERY-ACTA CHIRURGICA AUSTRIACA.
25. Macuka I, Junakovic I, Bozic D, Macuka I, Junakovic IT, Bozic D. Burnout among Palliative Care Professionals. DRUSTVENA ISTRAZIVANJA. 2020;29(2):287-308.
26. Macuka I, Tucak Junakovic I. A Cross-Sectional Study of Job Satisfaction and Intention to Leave Job in Palliative Care in Croatia. Journal of palliative care. 2021:8258597211046704.
27. Mantri S, Lawson JM, Wang ZZ, Koenig HG. Prevalence and Predictors of Moral Injury Symptoms in Health Care Professionals. JOURNAL OF NERVOUS AND MENTAL DISEASE. 2021;209(3):174-80.
28. McKinley N, McCain RS, Convie L, Clarke M, Dempster M, Campbell WJ, et al. Resilience, burnout and coping mechanisms in UK doctors: a cross-sectional study. BMJ Open. 2020;10(1):e031765.
29. McKinley NC. Resilience, professional quality of life and coping mechanisms in doctors and medical students.: Queens University Belfast; 2021.
30. Ntantana A, Matamis D, Savvidou S, Giannakou M, Gouva M, Nakos G, et al. Burnout and job satisfaction of intensive care personnel and the relationship with personality and religious traits: An observational, multicenter, cross-sectional study. Intensive and Critical Care Nursing. 2017;41:11-7.
31. Büssing A, Ostermann T, Matthiessen PF. Role of religion and spirituality in medical patients: confirmatory results with the SpREUK questionnaire. Health and quality of life outcomes. 2005;3(1):10.
32. Purvis T, Saylor D, Powell B, Biba G, Conti D, Crowe T, et al. Burnout and Resilience Among Neurosciences Critical Care Unit Staff. NEUROCRITICAL CARE. 2019;31(2):406-10.
33. Ramondetta L, Urbauer D, Brown A, Richardson G, Thaker P, Koenig H, et al. Work related stress among gynecologic oncologists. GYNECOLOGIC ONCOLOGY. 2011;123(2):365-9.
34. Roslan NS, Yusoff MSB, Ab Razak A, Morgan K, Shauki NIA, Kukreja A, et al. Training Characteristics, Personal Factors and Coping Strategies Associated with Burnout in Junior Doctors: A Multi-Center Study. HEALTHCARE. 2021;9(9).
35. Salmoirago-Blotcher E, Fitchett G, Leung K, Volturo G, Boudreaux E, Crawford S, et al. An exploration of the role of religion/spirituality in the promotion of physicians' wellbeing in Emergency Medicine. Preventive Medicine Reports. 2016;3:189-95.
36. Schmidt C, Roffler M. Coping with the Practice of Medicine: Religion, Spirituality, and Other Personal Strategies. JOURNAL OF RELIGION & HEALTH. 2021;60(3):2092-108.
37. Schmidt C, Roffler M. Who isn’t burned out? The role of spirituality in graduate medical education (GME). 2018.

38. See K, Zhao M, Nakataki E, Chittawatanarat K, Fang W, Faruq M, et al. Professional burnout among physicians and nurses in Asian intensive care units: a multinational survey. INTENSIVE CARE MEDICINE. 2018;44(12):2079-90.

39. Shetach A, Marcus O, Shetach A, Marcus O. The critical managerial capabilities of medical and nursing managers in an Israeli hospital. EVIDENCE-BASED HRM-A GLOBAL FORUM FOR EMPIRICAL SCHOLARSHIP. 2015;3(1):81-102.

40. St Onge JE, Allespach H, Diaz Y, Poitier A, Tamariz L, Paidas C, et al. Burnout: exploring the differences between U.S. and international medical graduates. BMC medical education. 2022;22(1):69.

41. Suttle M, Chase M, Sasser W, Moore-Clingenpeel M, Maa T, Werner J, et al. Burnout in Pediatric Critical Care Medicine Fellows*. CRITICAL CARE MEDICINE. 2020;48(6):872-80.

42. Teixeira C, Ribeiro O, Fonseca AM, Carvalho AS. Burnout in intensive care units - a consideration of the possible prevalence and frequency of new risk factors: A descriptive correlational multicentre study. BMC Anesthesiology. 2013;13:38.

43. Watson AG, Saggar V, MacDowell C, McCoy JV. Self-reported modifying effects of resilience factors on perceptions of workload, patient outcomes, and burnout in physician-attendees of an international emergency medicine conference. Psychology, health & medicine. 2019;24(10):1220-34.
