# Supplementary material for: Systematic review of the relationship between burn-out and spiritual health in doctors
Source: BMJ Open. 2023 Aug 8;13(8):e068402. doi: 10.1136/bmjopen-2022-068402 (PMC10414094; doi:10.1136/bmjopen-2022-068402)
Supplement: Author's manuscript [file bmjopen-2022-068402.draft_revisions.pdf]

BMJ Open

BMJ Open is committed to open peer review. As part of this commitment we make the peer review history of every article we publish publicly available.

When an article is published we post the peer reviewers' comments and the authors' responses online. We also post the versions of the paper that were used during peer review. These are the versions that the peer review comments apply to.

The versions of the paper that follow are the versions that were submitted during the peer review process. They are not the versions of record or the final published versions. They should not be cited or distributed as the published version of this manuscript.

BMJ Open is an open access journal and the full, final, typeset and author-corrected version of record of the manuscript is available on our site with no access controls, subscription charges or pay-per-view fees (http://bmjopen.bmj.com).

If you have any questions on BMJ Open's open peer review process please email info.bmjopen@bmj.com

BMJ Open

A systematic review of the relationship between burnout and spiritual health in doctors

Journal:	*BMJ Open*
Manuscript ID	bmjopen-2022-068402
Article Type:	Original research
Date Submitted by the Author:	16-Sep-2022

Complete List of Authors:	Whitehead, Orla; Newcastle University Population Health Sciences Institute, Biomedical Research Building<br>Moffatt, Suzanne; Newcastle University Population Health Sciences Institute<br>Warwick, Stephanie; Newcastle University Population Health Sciences Institute, Biomedical Research Building<br>Spiers, Gemma; Newcastle University, Population & Health Sciences Institute<br>Kunonga, Tafadzwa; Newcastle University Population Health Sciences Institute<br>Tang, Eugene; Newcastle University Faculty of Medical Sciences<br>Hanratty, Barbara; Newcastle University Population Health Sciences Institute

Keywords:	Human resource management < HEALTH SERVICES ADMINISTRATION & MANAGEMENT, MENTAL HEALTH, OCCUPATIONAL & INDUSTRIAL MEDICINE

SCHOLARONE™
Manuscripts

BMJ

I, the Submitting Author has the right to grant and does grant on behalf of all authors of the Work (as defined in the below author licence), an exclusive licence and/or a non-exclusive licence for contributions from authors who are: i) UK Crown employees; ii) where BMJ has agreed a CC-BY licence shall apply, and/or iii) in accordance with the terms applicable for US Federal Government officers or employees acting as part of their official duties; on a worldwide, perpetual, irrevocable, royalty-free basis to BMJ Publishing Group Ltd ("BMJ") its licensees and where the relevant Journal is co-owned by BMJ to the co-owners of the Journal, to publish the Work in this journal and any other BMJ products and to exploit all rights, as set out in our licence.

The Submitting Author accepts and understands that any supply made under these terms is made by BMJ to the Submitting Author unless you are acting as an employee on behalf of your employer or a postgraduate student of an affiliated institution which is paying any applicable article publishing charge ("APC") for Open Access articles. Where the Submitting Author wishes to make the Work available on an Open Access basis (and intends to pay the relevant APC), the terms of reuse of such Open Access shall be governed by a Creative Commons licence – details of these licences and which Creative Commons licence will apply to this Work are set out in our licence referred to above.

Other than as permitted in any relevant BMJ Author's Self Archiving Policies, I confirm this Work has not been accepted for publication elsewhere, is not being considered for publication elsewhere and does not duplicate material already published. I confirm all authors consent to publication of this Work and authorise the granting of this licence.

For peer review only

A systematic review of the relationship between burnout and spiritual health in doctors

Short title: Doctors' burnout and spiritual health- a systematic review

Ishbel Orla Whitehead[a], Suzanne Moffatt[b], Stephanie Warwick[a], Gemma Frances Spiers[a], Tafadzwa Patience Kunonga[a], Eugene Yee Hing Tang[a], Barbara Hanratty[a]

[a]Population Health Sciences Institute, Faculty of Medical Sciences, Newcastle University, Biomedical Research Building, Campus for Ageing and Vitality, Newcastle upon Tyne, NE4 5PL

[b] Population and Health Sciences Institute, Newcastle University, Baddiley-Clark, Newcastle upon Tyne, NE2 4AX

Corresponding author: Orla Whitehead. Email: orla.whitehead@newcastle.ac.uk orcid ID- 0000-0002-4171-8583

Declarations

Competing Interests: None declared.

Registration of review: Prospero- CRD42020200145

https://www.crd.york.ac.uk/prospero/display_record.php?RecordID=200145

Funding statement: OW is funded by the National Institute for Health Research (NIHR) on an in practice fellowship, grant number NIHR301074. The views expressed are those of the authors and not necessarily those of the NIHR or the Department of Health and Social Care. BH and SM are funded by the NIHR Applied Research Collaboration (ARC) North East and North Cumbria

Ethics approval: Not required

Contributorship: Conception and design of study: OW, SM, CJ and BH. Search design and searching: OW with assistance from Catherine Richmond and Hannah O'Keefe. Screening records: OW, SM, GS, ET, PK, BH. Data curation: OW, BH. Visualisation and rafting the manuscript: OW, SM, CJ, BH Revising the manuscript critically for important intellectual content and authorizing for publication: OW, SM, CJ, BH, GS, PK, ET

For peer review only - http://bmjopen.bmj.com/site/about/guidelines.xhtml

Abstract

Objective: To investigate the relationship between burnout and spiritual health amongst doctors

Design: Systematic literature review and narrative synthesis of cross -sectional studies

Setting: Any setting, worldwide

Participants: Doctors (and other healthcare staff if assessed alongside doctors)

Main outcome measure: Burnout measures and spiritual health assessment

Inclusion criteria: Any study including doctors that had any measure of burnout and spiritual health (or related concepts)

Quality Assessment: Appraisal of Cross-sectional Studies tool

Results: Five databases were searched from inception to March 2022, yielding 1049 studies. 40 studies met eligibility criteria and were included in this review. Low reported levels of spirituality were associated with high burnout scores, and vice versa. Religion was not significantly associated with lower levels of burnout. Few studies reported statistically significant findings, few used validated spiritual scores, and most were vulnerable to sampling bias.

Conclusions: Published research suggests that burnout is linked to spiritual health in doctors but not to religion. Robust research is needed to confirm these findings and develop effective interventions.

Prospero registration CRD42020200145.

For peer review only - http://bmjopen.bmj.com/site/about/guidelines.xhtml

Strengths and limitations of this study

- This is a novel, global, systematic review looking at the relationship between doctors' burnout and their spiritual health.
- A comprehensive, systematic approach was taken, with broad search terms, as there is inconsistency in headings used in different databases.
- Studies were heterogenous, and therefore a narrative analysis was conducted, as meta-analysis was not possible.

Keywords

Burnout, spiritual health, wellbeing, spirituality, religion, compassion fatigue, moral injury

Introduction

Burnout is defined by the World Health Organisation as an occupational phenomenon, resulting in exhaustion, mental distance or feelings of negativity or cynicism towards the job, and reduced professional efficacy.[1] This has long been a concern for doctors, and the COVID-19 pandemic has increased attention on the health and wellbeing of health professionals.[2 3] Doctors' descriptions of burnout include a loss of meaning in work, and objectification of patients and their families, rather than engaging with their humanity.[4] Burnout has been equated with moral injury,[5] referring to the harm caused when someone is required to act contrary to their internal ethical code.[6] As health services globally are placed under increasing pressure, there is growing concern around physician burnout, moral injury and related concepts causing harm to the workforce.[7 8]

The biomedical model that shapes much modern medical practice, has so far been unable to offer a comprehensive and holistic understanding of the burnout phenomenon.[9-11] This, along with the framing of burnout as an occupational phenomenon rather than a mental illness,[1] suggests that a different lens may be needed to develop effective approaches to burnout . In a recent survey, general practitioners (GPs) defined spiritual health in ways that overlapped with current constructs of burnout.[12] Spiritual health was framed by doctors as a positive concept.[12] Spiritual health encompasses meaning, purpose, self-actualisation,

For peer review only - http://bmjopen.bmj.com/site/about/guidelines.xhtml

transcendence and relationships with others beyond the self.[12] Spiritual or religious practices, including mindfulness and yoga, were also included in spiritual health definitions, and both are promoted as approaches to reduce burnout in the workplace.[13 14] Being true to a personal or religious ethical code, was also an aspect of spiritual health reported by GPs.[12] The concepts of spiritual health and burnout have been found to overlap in other groups of professionals.[15-17] This raises the possibility that promoting spiritual health may offer an effective approach to prevention and mitigation of burnout for doctors, with opportunities for intervention at an individual and organizational level.

This systematic review asks whether there is a measured association between burnout and spiritual health amongst doctors. It will consider the quantity and quality of quantitative evidence on burnout, spiritual health, and related concepts including spiritual wellbeing and distress, and religiosity by comparing all studies that have performed a measure of burnout and a measure of spiritual health, both in the broadest terms, in qualified doctors worldwide.

Methods

Searches and selection of studies

Studies to be included in the review were identified by searching the following databases: (1) EMBASE (1974–March 2022), (2) PsycINFO (1806– March 2022), (3) Ovid MEDLINE (1946–March 2022), (4) Web of Science and (5) Scopus. The search strategy below was developed in Embase and adapted for use in other databases by an information scientist. For comprehensiveness both MESH headings and key terms were used. A Cochrane filter[18] and term 'not editorial or letter' was added to filter out non-studies and opinion pieces.

All studies that included doctors as participants, with any assessment of both burnout (or similar concepts) and spiritual health (or similar concepts) were included. The outcomes considered were:

1. Burnout, moral injury, job satisfaction, compassion fatigue, work stress, or similar concepts.

2. Spiritual health, wellness, wellbeing, spirituality, religiosity or similar concepts.

For peer review only - http://bmjopen.bmj.com/site/about/guidelines.xhtml

Data extraction and synthesis

All records were double screened at title and abstract, with disagreements resolved by discussion with a third reviewer, or taken to full text screening. Rayyan software was used to manage records.[19] Attempts were made to retrieve all articles, with requests to authors. Emails to authors suggested that there could be publication bias, and therefore studies from 'grey literature' were included. Data extraction was performed by one reviewer and a proportion (10%) checked by a second reviewer. Study quality was assessed using the Appraisal of Cross-sectional Studies tool (AXIS).[20] While this tool does not give a 'numerical score', it assessed the methodology and internal consistency of the studies, as well as risk of bias, and as such allows assessment of the merits of each criterion as applied to that study. Heterogeneity in study design, scales, and methods of analysis meant that meta-analysis was not possible, and a narrative synthesis was conducted. This analysed the assessments used for burnout and spiritual health, and synthesised the data, including common biases.

The review was registered on Prospero, available from:
https://www.crd.york.ac.uk/prospero/display_record.php?ID=CRD42020200145

Patient and public Involvement

This systematic review was prompted by patient and public involvement in a previous study looking at spiritual health in primary care. The public felt that the health of doctors, and burnout, was a priority for this topic.

Results

Forty studies met the inclusion criteria, from screening of the 1049 records identified in the electronic searches (Fig 1). All included studies provided an assessment of burnout and spiritual health (or related concepts) but there was a high level of heterogeneity in the measures used.

For peer review only - http://bmjopen.bmj.com/site/about/guidelines.xhtml

FIG 1 HERE

The number of study participants ranged from 4501 in the largest study[21] to 7 in the smallest.[22] Twenty studies included other healthcare staff in addition to the medical staff participants.[22-41] Four studies included primary care physicians,[30 42-44] with most studies involving doctors from intensive or critical care, emergency departments and internal medicine.[21-24 27 35 36 38 39 42 44-54](Figure 2) No studies were identified including doctors in psychiatry or public health. Data from doctors were not always analysed separately.[23 24 26 27 32 37 39 40]

The oldest study was also the largest, published in 1999 [21]. Three studies were conducted during the COVID-19 pandemic [39 40 47]. A USA study showing lower burnout scores in those who had a faith (including 'spiritual but not religious') [39], a Singapore study with participants who preferred religious coping to thanks from the public [40] and a Portuguese study which showed no association between burnout scores and religiosity in doctors [47]. Five of the forty studies reported findings from surveys in 2020 onwards.[39 40 45 47 55]

FIGURE 2 HERE

FIGURE 3 HERE

Almost half of the studies (19) were from the USA [21 25 26 32 35 39 41 42 48-50 52-54 56-60] reflecting the dominance of North American researchers in this area (Figure 3). Twelve studies included participants from Europe.[24 27 31 33 37 38 43 45-47 51 61] The largest Asian study recruited widely, pooling data from diverse countries in wealth, religion and culture, such as Saudi Arabia, Laos and Bangladesh.[36]

Study Quality

For peer review only - http://bmjopen.bmj.com/site/about/guidelines.xhtml

The majority of studies were assessed to be poor quality, with risks of selection bias, response bias, and publication bias. One study, Roslan et al[44], met all the AXIS criteria fully, including justification of sample size, and there were seven studies which were of otherwise high quality as assessed by the AXIS tool (meeting 18-19 of the 20 criteria)[28-30 32 34 50 57]. Three studies were not yet published in full, and therefore not had peer review.[26 41 43] Authors reported difficulty publishing their findings.[26 62] This, as well as other studies where data was gathered on burnout and spiritual health, but not all comparison data was published,[23 33 40 43 44 56] may have introduced publication bias. These were included as it was suspected this topic may be affected by publication bias. The majority of studies did not use a validated measure of spiritual health, with many studies using non-validated assessments of burnout.

Relationship between burnout and spiritual health in doctors

These heterogenous studies drew a wide range of conclusions (Table 1). Seventeen studies found an association between spiritual health or related concepts and lower levels of burnout or related concepts,[25 27 28 30-33 36 39 41 43 44 46 54 59 63 64] seventeen studies found no statistically significant association,[21-23 26 29 35 37 38 42 47-49 51-53 61 65] and one study found higher compassion fatigue in those using spiritual type behaviours.[50] Across the eight studies rated high in quality, evidence remained inconsistent. There was evidence of: an inverse association between religiosity and burnout [30 32]; no association between spiritual concepts and burnout scores;[51 57] an association between prayer and meditation and higher compassion fatigue [50]; an association between irregular spirituality routines and burnout [44]; a link between no spirituality and low personal accomplishment;[28] and an association between spirituality and high emotional exhaustion on univariate analysis but not on multivariate analysis.[34] One study found an association between personal accomplishment and burnout, on exclusion of respondents who omitted the spirituality question.[29] Where statistically significant associations between spiritual health (or similar), or religiosity, and burnout (or similar) were found, the effects were typically small.

The removal of studies rated low in quality (score 12 or under on AXIS criteria) does not enhance consistency the findings. Further, there does not appear to be a pattern in terms of whether an association is shown based

For peer review only - http://bmjopen.bmj.com/site/about/guidelines.xhtml

on country, whether that country has a state religion, but this assessment is limited by the predominance of the USA in these studies. The three most recent studies from the COVID pandemic[39 40 47] do not clarify the direction of evidence, with one study finding an association,[39] one did in nurses but not doctors,[47] and one where participants agreed they used religious coping, but did not compare religious coping and burnout scores.[40]

Studies which looked at wider spiritual health only, rather than asking about religion, were more likely to find an association between burnout and spiritual health. Seven studies of those which asked or measure spirituality and not religion found an association,[25 27 28 31 41 46 59] and three studies did not.[24 49 53] Religion, or religious coping, alone does not appear to have a consistent effect on burnout rates, however, wider spiritual health may have some effect.

Table 1: Findings from each study

Study	Comparisons of Burnout and Spiritual Health	Estimate or test statistic (95% confidence intervals in brackets, if given)	P value	The association of burnout (or similar concepts) with spiritual health (or similar concepts)
Alosaimi et al[63] 2018 Saudi Arabia	Perceived stress score was compared with use of religious coping (Brief-COPE)	Spearman's rho −0.09	0.030	Lower stress scores in those with higher religious coping
	Perceived stress score was compared for those who use religious coping (open ended question)	Number of participants:<br>Low PSS-66<br>Medium PSS- 48<br>High PSS- 33	0.004	
Antonsdottir et al[39] 2022 USA	Religion was compared with each domain of the MBI	DP β / EE β / PA β<br>Christian −2.73*** / −4.97** / 1.76*<br>Jewish −0.55 / −1.82 / 0.03<br>Spiritual, not religious −2.35** / −2.64 / 1.86	*** p<0.001<br>**p<0.01<br>*p<0.05	Lower burnout scores in those with a religious/spirituality.

For peer review only - http://bmjopen.bmj.com/site/about/guidelines.xhtml

		Other −2.58*** −3.59 1.53		
Azoulay[45] 2020 Europe	Anxiety and depression scores were compared with religiosity			Burnout score and religiosity not commented on.
Baruah[23] 2019 India	MBI compared with type of religion (no non-religious)	EE: χ2= 1.881	DP p=0.7579<br>EE p=0.3905<br>PA p=1.00	No association found
Ben-Itzak[46] 2015 Israel	The extent to which work provides meaning was associated with burnout scores	β=-3.144 (0.045- 6.24)	P<0.05	Higher work related meaning was associated with lower burnout scores.
Büssing et al[66] 2014 Germany	The aspects of spirituality domains were compared with the cool down index and perceived work burden	Aspects of spirituality domains / Cool down index / Perceived work burden<br>*Religious orientation* ρ=−0.078 ρ=−0.044<br>*Search for insight/wisdom* ρ=−0.107 ρ=−0.036<br>*Conscious interactions* ρ=−0.14* ρ=−0.165*<br>*Transcendence conviction* ρ=−0.106 ρ=−0.044	*P<0.01	No relationship- no statistical association between spiritual score and cool down reactions or perceived work burden
Chor et al[40] 2021 Singapore	55.8% used religious coping more than acts of gratitude from the public.			Burnout and religious coping use not compared.
Clark[25] 2007 USA	The statistical model chosen assumes a relationship between spirituality and job satisfaction via integration and self-actualisation	χ^2 0.614	p- 0.433	Higher job satisfaction was associated with higher spirituality
Correia et al[47] 2020 Portugal	Religiosity was compared with burnout score domains	Exhaustion- β= 0.07<br>Disengagement- β= 0.02		No association

For peer review only - http://bmjopen.bmj.com/site/about/guidelines.xhtml

Das[22] 2016 India	Religion was compared with burnout domains	EE- χ^2= 1.556<br>DP- χ^2= 2.917<br>PA- χ^2= 1.556	EE- p= 0.212<br>DP- p= 0.233<br>PA- p= 0.459	No association
Doolittle et al[49]2013 USA	Correlation between SIBS score and MBI	EE- r=-0.11<br>DP- r=-0.13<br>PA- r=0.28	EE- p=0.25<br>DP- p=0.17<br>PA- p=0.003	No statistical association
Doolittle[48] 2020 USA	Three domains from the Durel are reported as Pearson's correlation coefficients	EE / DP / PA<br>Organized religion − 0.12 / − 0.21 / + 0.14<br>Private practices − 0.21 / − 0.12 / + 0.23<br>Religiosity − 0.18 / − 0.18 / + 0.20		No association
Doolittle and Windish[67] 2015 USA	Spearman's rank correlation coefficient	EE- 'not significant'<br>DP- 'not significant'<br>PA- 0.35	PA- p=0.03	No association
Frank[21] 1999 USA	Religious fervor was compared with desire to become a physician again	Odds ratio- 1.3 (1.1-1.6)		No direct association found
Glasberg et al[64] 2007 Brazil	Religious identity was compared with MBI scores		p=0.038	Higher burnout in the non-religious
Glebocka et al[27] 2007 Poland	Hobfall's gains and loses were compared with MBI scores (translated), correlation coefficients reported	EE / DP / Professional Cynicism<br>Spiritual resources gain 0.14 / 0.13 / 0.29<br>Spiritual resources loss 0.09 / 0.35* / -0.06	*p<0.05	Positive correlation between burnout and spiritual resource loss.
Gribben et al[50] 2019 USA	Compares prayer/meditation and compassion fatigue	β=0.12	p<0.001	Higher compassion fatigue in those using spiritual type behaviours
Guay et al[26] 2019 Americas	Number of participants with burnout compared by religion.	χ^2 values not given	Religious affiliation p= 0.184<br>Importance of	No association

			spirituality<br>$p=0.984$<br>Importance of religion<br>$p=0.084$	
Guest et al[56]<br>2011<br>USA	Spirituality mentioned as protective factor against burnout	Numeric data not given		MBI and spirituality not compared
Harper et al[57]<br>2020<br>USA	Believer/non-believers MBI scores compared	χ^2 values not given	$p=0.69$	No association
Koh et al[28] 2015<br>Singapore	The odds of being burned out and answering 'no' to being spiritual were compared.	odds ratios:<br>High DP- 2.83 (1.2-6.3)<br>Low PA- 2.85 (1.4-6.0)<br>High EE- 1.67 (0.76-3.64)<br>Overall burnout- 1.80 (0.86-3.71)		Those who answered 'no' to the spiritual question had lower personal accomplishment
Lal et al[68] 2020<br>India	MBI domains were compared with whether religious or spiritual beliefs influence work	ANOVA values not given	PA<br>$p=0.002$	No overall association
Leonelli et al[30]<br>2017<br>Brazil	Perceived stress was compared with religious activity	$\beta= 3.1$ [0.3- 5.8]	$p=0.03$	Lower religious activity associated with higher perceived stress
Leu et al[61] 2020<br>Switzerland and Australia	Atheism vs any religion was compared with burnout	$\beta= -52.5$	$p=0.13$	No association
Macuka et al[33]<br>2020<br>Croatia	Compares spiritual question and burnout scores	EE- $\beta= -0.20$<br>$r=-0.19$<br>Disengagement- $\beta=-0.18$<br>$r=-0.19$	$p<0.05$<br>$p<0.05$<br>$p<0.05$<br>$p<0.05$	Religiosity was associated with lower emotional exhaustion.
Macuka et al<br>2021[31]<br>Croatia	Spiritual question and job satisfaction were compared	$r=0.15$	$p>0.05$	Higher job satisfaction in those with higher religiosity
Mantri et al<br>2021[32]<br>USA	Compares religiosity and moral injury scores	data not given		Moral injury symptoms and burnout inversely related to religiosity.

McKinley et al 2020[43]<br>full data from Dr McKinley's thesis[69]	Religious coping was compared with burnout score	Unadjusted β= -0.42	p<0.01	Higher religious coping associated with lower burnout
Ntantana et al[51] 2017<br>Greece	Spirituality and burnout were compared, only EE reported	OR for high burnout given high EE- 1.482 (0.823-2.670)		No association
Purviss et al[35] 2019<br>USA	Religion (Catholics vs other) was compared with burnout score.		p = 0.026	No association
Ramondetta et al[58] 2011<br>Greece	Correlation between religiosity and Work Related Strain Inventory (WRSI)	Duke religiosity index and WRSI r=0.08<br>Intrinsic religiosity scale and WRSI r=0.05		No association
Roslan et al[44] 2021<br>Malaysia	Irregular spirituality routines were compared with burnout scores	OR- 2.24 (1.49–3.37))	p<0.001	Work related burnout was associated with irregular spirituality routines
Salmoirago-Blotcher[52] 2016<br>USA	Various religious/spiritual questions were compared with burnout scores	**Organized religiosity** — **Adjusted OR**<br>Never — Ref.<br>Any — 0.89 (0.36 to 2.20)<br>**Religious Affiliation**<br>None — Ref.<br>Any — 0.51 (0.19 to 1.39)<br>**Private prayer**<br>Never — Ref.<br>Any — 0.66 (0.27 to 1.60)<br>**Meditation**<br>Never — Ref.<br>Any — 0.56 (0.19 to 1.62)<br>**Self-rated spirituality**<br>Slightly/not spiritual — Ref.		No association

For peer review only - http://bmjopen.bmj.com/site/about/guidelines.xhtml

		Moderately spiritual 1.14 (0.45 to 2.88)<br>Very spiritual 0.15 (0.02 to 1.46)<br>**Religious rest**<br>Never Ref.<br>Any 0.51 (0.16 to 1.65)<br>**"I try hard to carry my religious beliefs over into all my life"**<br>Disagree Ref.<br>Agree 0.70 (0.28 to 1.74)		
Schmidt and Roffler[62] 2021<br><br>Quantitative data given by author, unpublished[41]	Correlation between spiritual score and burnout score	DP ρ= -0.37<br>EE ρ= -0.55<br>PA ρ= 0.62	$p<0.05$<br>$p<0.05$<br>$p<0.05$	Association between all domains of the burnout score and the spiritual score.
See et al[36] 2018 Large study, across Asia	Compared religious background or belief and high burnout	OR 0.69 (0.49–0.96)	P- 0.027	Religiosity was found to be protective against burnout in doctors (and nurses).
Shetach et al[37] 2015 Israel	Job satisfaction scores for Christians were compared with non-Christians	Religion β<br>Christian 0.46<br>Non-Christian 0.41	$p>0.05$	No association
St Onge et al[53] 2022 USA	Spiritual question was compared with burnout scores	Personal burnout β= -0.77<br>Work burnout β= -1.87885<br>Patient burnout β= −1.65<br><br>Odds ratio for overall burnout: 0.55(0.23-1.31)	$p>0.05$<br>$p>0.05$<br>$p>0.05$<br><br>p= 0.18	No association
Suttle et al[59] 2020 Asia	Mean MBI scores were compared with yes/no to spiritual question	Mean<br>EE- 26.77<br>DP- 11.278<br>PA-36.620	 p= 0.036<br>p= 0.011<br>p= 0.021	Higher burnout scores were associated with identifying as non-spiritual.
Teixeira et al[38] 2013 Portugal	Burnout scores in the religious were compared to non-religious	Odds ratio for burnout: 0.907	p=0.747	No association

Watson[70] 2019<br>USA	Mean burnout domain scores were compared with religious/spiritual question	Spirituality/religion<br>Mean EE<br>Mean DP<br>Mean PA	Disagree<br><br>3.98<br>3.95<br>5.31	Neutral<br><br>3.80<br>3.71<br>5.87	 p=0.36<br>p=0.13<br>p=0.43	Higher emotional exhaustion and lower personal accomplishment were correlated with those who disagreed with the spiritual question. No correlation with depersonalization. One-way ANOVA showed no difference between means.

MBI- Maslach Burnout Inventory DP- Depersonalisation EE- Emotional Exhaustion PA- Personal Accomplishment

SIBS- Hatch Spiritual Involvement and Beliefs Scale WRSI- Work Related Strain Inventory

Brief COPE- Brief Coping Orientation to Problems Experienced Inventory

For peer review only

For peer review only - http://bmjopen.bmj.com/site/about/guidelines.xhtml

Table 2: Study details, AXIS quality assessment, spiritual wellness assessment and burnout score used

Study	Study design	Population	Measure of spirituality	Measure of burnout
Alosaimi et al[63] 2018 Saudi Arabia	Cross-sectional study	582 consultant physicians	Brief COPE (2 items to score religious coping)	Perceived stress Scale
Antonsdottir et al[39] 2022 USA	Cross-sectional study	639 (96 physicians)	Religious affiliation (including 'spiritual but not religious")	MBI
Azoulay[45] 2020 Europe	Cross-sectional study	1132 respondents- ICU specialists	Self rated religiosity 1-100	MBI
Baruah[23] 2019 India	Cross-sectional study	68 participants (doctors, nurses and paramedics)- Emergency Department	Religion	MBI
Ben-Itzak[46] 2015 Israel	Cross-sectional study	70 emergency physicians	"My job gives me meaning"yes/no<br>Open ended questions:<br>1. Self-fulfillment<br>2. Meaningful relationships<br>3. Excitement and adrenaline rush<br>4. Providing meaningful assistance.	MBI
Büssing et al[66] 2014 Germany	Cross-sectional study	489 anthroposophic medical staff (41% physicians)	Aspects of Spirituality Questionnaire (ASQ)	Cool down Index<br>Utrecht Work engagement scale.
Chor et al[40] 2021 Singapore	Cross-sectional study	337 participants (127 doctors) Emergency department	Use of religious coping	Copenhagen Burnout Inventory
Clark[25] 2007 USA	Cross-sectional study	215 hospice staff, 2.3% physicians	Jarel spiritual wellbeing scale	8-item job satisfaction scale
Correia et al[47] 2020 Portugal	Cross-sectional study	497 health professionals working during COVID-19 (229 physicians)	"Regardless of whether you	Oldenburg Burnout Inventory

For peer review only - http://bmjopen.bmj.com/site/about/guidelines.xhtml

			belong to a particular religion, how religious would you say you are?"	
Das[22] 2016 India	Cross-sectional study	7 emergency department participants (4 doctors)	Religion	MBI
Doolittle et al[49]2013 USA	Cross-sectional study	108 hospital residents	Hatch Spiritual Involvement and Beliefs Scale (SIBS), chosen to try and avoid cultural bias.	MBI
Doolittle[48] 2020 USA	Cross-sectional study	337 internal medicine physicians	COPE and DUREL, self reported spiritual and/or religious	ProQoL (domains of burnout, secondary stress and compassion satisfaction)
Doolittle and Windish[67] 2015 USA	Cross-sectional study	Interns 66 in the summer, and 53 in winter cohorts	Coping Orientation to Problems Experienced (COPE), Hatch SIBS, self perception of spirituality and religiosity.	MBI
Frank[21] 1999 USA	Cross-sectional study	4501 female physicians	"Religious identification" none, low strong or very strong	"Are you generally satisfied with your career?" "If you lived your life again, would you become a physician?"
Glasberg et al[64] 2007 Brazil	Cross-sectional study	102 oncologists	Religious affiliation	MBI
Glebocka et al[27] 2007 Poland	Cross-sectional study	77 people (41 doctors)	Hobfoll's Questionnaire of Self-Assessment of Gains and Losses)7 statements of 'spiritual resources' gained in last 12 months)	MBI
Gribben et al[50] 2019 USA	Cross-sectional study	390 paediatric emergency medicine trainees	Prayer/meditation as self care activity	Compassion Fatigue and Satisfaction Self-Test for Helpers (adapted)
Guay et al[26] 2019 Americas	Cross-sectional study	221 (RR 63%) health care professions, 58.8% physicians,	Importance of religion<br>Importance of spirituality<br>Self-definition as religious person<br>Self-definition as spiritual person	Respondents categorised as burned out if they agreed with: ''I am definitely burning out and have one or more symptoms of burnout, such as physical and emotional exhaustion,'' ''The symptoms of burnout that I'm experiencing won't go away. I think about frustration at

			Frequency of religious practice (praying/meditation)	work a lot,'' ''I feel completely burned out and often wonder if I can go on. I am at the point where I may need some changes or may need to seek some sort of help.''<br><br>Respondents were categorized as not burned out if they agreed to either of the statements: (1) ''I am definitely burning out and have one or more symptoms of burnout, such as physical and emotional exhaustion, (2) Occasionally I am under stress, and I don't always have as much energy as I once did, but I don't feel burned out.''
Guest et al[56] 2011 USA	Cross-sectional study	72 cancer surgeons	Linear Analog Scale Assessment of Quality of Life (LASA QOL) which includes spiritual QoL	MBI
Harper et al[57] 2020 USA	Cross-sectional study	70 Urologists	Believer/non-believer	MBI
Koh et al[28] 2015 Singapore	Cross-sectional study	273 palliative care staff (28.1% doctors)	Single question: "Do you consider yourself spiritual?"	MBI
Lal et al[68] 2020 India	Cross-sectional study	345 of medical and surgical faculty	Single question- "My religious or spiritual beliefs strongly influence my approach to my work and patients."	MBI<br>Job stress and job satisfaction questionnaire<br>Jefferson scale of physician empathy
Leonelli et al[30] 2017 Brazil	Cross-sectional study	450 primary health care professionals (27 doctors)	Religious activity	Perceived stress scale (PSS)continuous
Leu et al[61] 2020 Switzerland and Australia	Cross-sectional study	412 participants (237 Swiss and 175 Australian)	Religion	MBI
Macuka et al[33] 2020 Croatia	Cross-sectional study	123 palliative care staff (18 doctors)	"Whether or not you consider yourself a believer or not, how much of a spiritual person you consider yourself, that is, how much you are interested	Oldenburg burnout inventory

For peer review only - http://bmjopen.bmj.com/site/about/guidelines.xhtml

			in the sacred or the supernatural?" likert scale 1-5	
Macuka et al 2021[31] Croatia				
Mantri et al 2021[32] USA	Cross-sectional study	181 participants	Religious affiliation, and 'religious commitment' using Belief into Action (BIAC) scale	Moral Injury Symptom Scale-Healthcare Professionals version and MBI
McKinley et al 2020[43] full data from Dr McKinley's thesis[69]	Cross-sectional study	1382 participants, 69% hospital, 31% primary care	BRIEF COPE, includes religious coping	ProQOL-V includes burnout, compassion satisfaction and secondary traumatic stress sub domains.
Ntantana et al[51] 2017 Greece	Cross-sectional study	149 physicians, 320 nurses from intensive care	Religious and Spiritual attitudes questionnaire[71], designed by Büssing for use with patients, rather than staff. The authors report it was chosen as it avoids exclusive religious terminology, however in the paper by Büssing referred to, they report that a positive attitude towards religion and spirituality is necessary for some aspects of the scale.	MBI
Purviss et al[35] 2019 USA	Cross-sectional study	64 neurosciences critical care staff (10 doctors)	Religion	MBI
Ramondetta et al[58] 2011 Greece	Cross-sectional study	273 oncologists	Religious affiliation, self-rated religiosity and spirituality, Intrinsic Religiosity Survey, and Duke religion Index (DRI- a measure organisational religiosity)	Work related strain inventory (WRSI)- correlates with MBI
Roslan et al[44] 2021 Malaysia	Cross-sectional study	754 hospital interns	BRIEF COPE, includes religious coping, and descriptors of regular versus irregular spirituality routines based on several major	Copenhagen Burnout Inventory

			religions professed in the country.	
Salmoirago-Blotcher[52] 2016 USA	Cross-sectional study	138 emergency physicians	Religious affiliation, Self-rated spirituality, Fetzer Institute Multidimensional Measurement of Religiousness/Spirituality for Use in Health Research- used to assess religious/spiritual practice, and religious commitment ("I try hard to carry my religious beliefs over into all my other dealings in life), questions added to assess religious rest, and spiritual counsel.	2 item MBI
Schmidt and Roffler[62] 2021<br><br>Quantitative data given by author, unpublished[41]	Cross-sectional study	65 medical education faculty	The Functional Assessment of Chronic Illness Therapy- Spiritual Well-Being Scale, a modified version for non-illness (FACIT-Sp-Non-Illness)	MBI
See et al[36] 2018 Large study, across Asia	Cross-sectional study	992 intensive care physicians	Religious background or belief	MBI
Shetach et al[37] 2015 Israel	Cross-sectional study	107 respondents (40 doctors)	Religion- (Christian vs non-Christian)	Job satisfaction questionnaire
St Onge et al[53] 2022 USA	Cross-sectional study	222 interns	"My spiritual beliefs are very important in helping me cope with stressors."	Copenhagen Burnout Inventory
Suttle et al[59] 2020 Asia	Cross-sectional study	47 programme directors and 187 fellows in paediatric critical care	"Do you consider yourself to be a spiritual person?" yes/no	MBI
Teixeira et al[38] 2013 Portugal	Cross-sectional study	300 Intensive Care staff (82 physicians)	Religion yes/no	MBI
Watson[70] 2019 USA	Cross-sectional study	54 emergency physicians	Single question- "spirituality/religion is important in my life"	MBI

For peer review only - http://bmjopen.bmj.com/site/about/guidelines.xhtml

MBI-Maslach Burnout Inventory

Quantification of burnout

Twenty one studies[22 23 27-29 35 36 38 39 41 42 45 46 49 51 54 56 57 59 61 64] used the complete Maslach Burnout Inventory (MBI) [72] (Table 1). One study used a 2-item ‘MBI’ [52] in an emergency department population, that has been found to correlate with the full MBI in a similar population.[73] Two studies used the Oldenburg Inventory,[33 47] a measure similar to MBI, but more applicable to people not directly involved in patient care. It also measures engagement with work, not including professional accomplishment [74]. Three more recent studies [40 44 53] used the Copenhagen Burnout Inventory (CBI)[75], a free to use (MBI is commercially available) measure of personal, work and patient related burnout, which identifies a similar burned out group to the MBI [76]. Other tools used included the perceived stress scale (PSS) [30 63], Cool Down Index [24] and Work Related Strain Inventory (WRSI) [58]. Whilst these do not measure burnout directly, they have been found to correlate with burnout as measured by the MBI.[58 77 24 78] The Professional quality of life (proQOLl) scale used by two studies [43 48] measures compassion fatigue, which is considered to be a phenomenon including burnout by the ProQOL authors [79]. One study [50] used the Compassion Fatigue Test for Helpers. Clark et al [25] used an 8 item job satisfaction scale, which included some domains that could be considered as spiritual, for example the sense of calling, meaning and purpose in life and being of worth [25]. Frank et al[21] also measured job satisfaction and desire to become a physician again. Shetach et al [37] also used a job satisfaction questionnaire, translated into Hebrew, designed for an unpublished thesis in marketing, and not validated with healthcare staff.

Assessing spiritual health, wellbeing, spirituality and/or religiosity

An array of methods were used to assess spiritual health or similar concepts (table 2). Validated tools include the Functional Assessment of Chronic Illness Therapy- Spiritual Well-Being Scale , a modified version for non-illness (FACIT-Sp-Non-Illness)[80]; the Jarel spiritual wellbeing scale,[81] and Hatch spiritual involvement and belief scale [82]. Büssing et al[24] used the Aspects of Spirituality questionnaire, developed by the authors in a niche setting

For peer review only

of a particular philosophical institution [83]. The Fetzer Institute Multidimensional Measurement of Religiousness/Spirituality for Use in Health Research [84] was also used to assess religious practice, along with general, unvalidated, questions about religious commitment, and spiritualty [52]. A systematic review identified the FACIT and the Spirituality Index of Wellbeing [85] as being the highest most well validated instruments [86], however only one unpublished study [41] used the former, and none used the latter. Many studies used general coping scores, such as Brief COPE [87] and the Hobfoll's Questionnaire [88], general quality of life scores or including spiritual practice in general questions, which have aspects of spiritual health as part of a wider assessment. Some studies used single questions, e.g. "my job gives me meaning",[79] "my religious and spiritual beliefs strongly influence my work and patients" [29], "Spirituality/religion is important in my life" [54] or "do you consider yourself spiritual"[28 59]. Seventeen studies which asked about religion only (table 2). The Duke University religion index (DUREL) [89] measures organisational religiosity, measuring religious practice, and was used alongside Hoge's Intrinsic Religiosity Scale by Ramondetta et al [58]. The difference between spirituality and religiosity is not always delineated by the authors: Frank et al used religiosity data asking how 'strongly' participants were religious, and then argue that physicians could be encouraged to explore spirituality to increase satisfaction [21], however spirituality is not addressed in the data, and Macuka et al discussed religiosity, but their question appeared to ask more generally about spirituality [33]. Ramondetta et al asked participants whether they were religious, spiritual, both or neither, but only quantified religiosity.[58]

Discussion

Key findings

There is increasing attention on both burnout and on the relationship between doctors' burnout and their spiritual health. This has become even more critical in the aftermath of the COVID-19 pandemic. Most of the research in this review reported positive associations between higher levels of spiritual health or related concepts, and lower levels of burnout or related phenomena. A small number of studies found no association, with two describing greater distress with higher levels of religiosity. Similar to other reviews in this area, research from the USA is dominant, with few studies from more secular areas. The one high quality study on the topic

For peer review only - http://bmjopen.bmj.com/site/about/guidelines.xhtml

found that spirituality routines lower the risk of burnout [44]. Few studies reported statistically significant findings and the marked study heterogeneity did not support metanalysis. While this review suggests that there is probably a link between spiritual health and burnout in doctors, the quality of the evidence is poor, and high quality robust quantitative studies of the relationship between spiritual health and burnout in doctors is needed.

Comparison with other work

To our knowledge, this is the most comprehensive review of burnout and spiritual health in doctors to date. This review offers a different perspective from other work in this area that utilise a biomedical approach to burnout rather than a holistic approach to include spiritual health. [90-92] Williams et al's systematic review of the consequences of physician burnout considered the physical and mental health implications of burnout in their review, but neglected to mention spiritual health or distress.[91] Chow et al's scoping review of religion and spirituality in residents included discussion of the link between reduced burnout and spirituality [93]. Sibeoni et al's systematic review and metasynthesis of physician's views of burnout includes aspects of spiritual health in 'calmness or letting go', but did not analyse spiritual health as an aspect of overall health [94]. These authors reported that individual factors (including spiritual) and relational factors were more important than organizational concerns in protecting doctors from burnout [94]. Current emphasis is on the role of COVID-19 in doctors' burnout[95], with Jefferson et al finding studies considered gender and age effects on burnout during COVID [96] but spiritual health is not mentioned. While this review is limited by the quality of the studies included, there does appear to be merit in the hypothesis of looking wider than the biomedical model.

Common study limitations

There were a number of limitations common to the studies in this review: response bias, social desirability bias, sampling bias, survivorship/selection bias, and cultural or language bias. There also appears to be a publication bias, with the topic of spiritual health being controversial- with data on spiritual health not reported in the published paper, despite being collected [43 45]. The majority of studies were vulnerable to non-response and social desirability bias. Response rates were highly variable, range 5-99%. Use of gatekeepers, such as heads of departments [79] or directors[36 38 59] could cause sampling bias, as could use of convenience sampling at conferences.[45 48 54] Non-responders were seldom (eight studies) described. [21 24 28 32 44 50 55 68] Pressures from the COVID pandemic may have affected studies carried out during this period.[39 40 47] Social desirability bias was

For peer review only - http://bmjopen.bmj.com/site/about/guidelines.xhtml

possible due to staff feeling unable to respond honestly in very religious, or very secular environments, or share the true depth of their burnout symptoms[28]. Studies that took place in the work setting were vulnerable to survivorship/selection bias.[26-30 34 54] Purvis et al excluded residents, as they were not permanent staff.[35]

Just under half (n=19) of the studies used the Maslach Burnout Inventory (MBI) [72] a measure of burnout often considered the 'gold standard'[97] burnout inventory, Often, studies presented data on the influence of factors such as spiritual health on burnout, when one domain only was affected.[28 29 49] The use of other inventories were not justified as to whether this was because these were better tools in their context, because they are free to use, or another reason.

Despite there being multiple available scales of religiosity or spiritual health and wellbeing,[86 98 99] few studies used a validated tool to assess personal spiritual health or related concepts. With the growth of the "spiritual, but not religious"[39] and secular societies[100], there is merit in using validated tool to assess current spiritual health state.[86] The single questions about spiritual wellbeing or similar were untested and unvalidated, and therefore may have introduced bias to the studies, and limit the utility of the data.

The use of measures in English with populations whose first language is not English may affect the quality of results. In some studies, measures were translated into the native language.[22-24 27 33 37 64 79] However in other studies, English measures were used, without regard to the language and culture of participants,[28 36 45 63] or without stating which language/translation was used.

Strengths and Limitations of this review

This review is broad, and novel, including a wide definition of aspects of spiritual health. The lack of consistency in indexing for spiritual health and religiosity across electronic database (Medical Subject Headings) meant development of comprehensive searches was a challenge. However, the diverse range and number of papers screened suggests that the searches were sufficiently broad. While most studies are from the USA, the other studies are diverse in country of setting, and cover multiple different cultures and faiths. An objective assessment was made of study quality, using the AXIS tool. Unfortunately, heterogeneity of the measures and analysis used in the studies has meant meta analysis was not possible. Some published abstracts were

For peer review only - http://bmjopen.bmj.com/site/about/guidelines.xhtml

For peer review only

unobtainable, and publication bias likely affects this review, although the direction of this effect is unknown. This was mitigated by the inclusion of unpublished data on the topic.[26 41 43]

Future research

While there have been studies that have identified this topic as being of interest, these have rarely used validated measures of both variables- burnout and spiritual health. Research utilising accepted and validated tools to measure both spiritual health and burnout are needed, especially in Northern Europe, where research is limited. Future works should also strive to recruit study populations that not limited to people with specific interest in the topic or strong religious views. Identification, or refutation, of a link between spiritual health and burnout will allow fresh assessment of current organizational and individual interventions for burnout. Developing and evaluating interventions that improve doctors' spiritual health may mitigate against burnout.

Conclusions

A link between spiritual health and burnout in doctors has been hypothesised by multiple studies, and interest in the link appears to be growing. However, studies investigating this have been vulnerable to many methodological flaws. Despite this, and limited evidence for efficacy [101], practices rooted in spiritual practice, such as mindfulness, meditation, and yoga, are recommended to prevent burnout.[35] Since there does not appear to be a relationship between the narrower concept of religiosity and burnout, research into any association between broader spiritual health and burnout, using validated tools, is needed to examine the hypothesis that spiritual health and burnout are associated concepts, and to prompt research into interventions that could prevent burnout in doctors.

References

1. World Heath Organisation. Burn-out an "occupational phenomenon": International Classification of Diseases 2019 [Available from: https://www.who.int/mental_health/evidence/burn-out/en/.
2. Locke T. Medscape UK Doctors' Burnout & Lifestyle Survey 2020 2020 [Available from: https://www.medscape.com/slideshow/uk-doctors-burnout-2020-6013312?src=mkm_ret_11012020_mscpmrk_uk_burnout_int&faf=1 accessed 22/03/2021.

For peer review only - http://bmjopen.bmj.com/site/about/guidelines.xhtml

3. Lemaire JB, Wallace JE. Burnout among doctors. *BMJ* 2017;358:j3360. doi: 10.1136/bmj.j3360
4. Fred HL, Scheid MS. Physician Burnout: Causes, Consequences, and (?) Cures. *Tex Heart Inst J* 2018;45(4):198-202. doi: 10.14503/THIJ-18-6842
5. Dean W, Talbot S, Dean A. Reframing Clinician Distress: Moral Injury Not Burnout. *Fed Pract* 2019;36(9):400-02.
6. Talbot SG, Dean W. Physicians aren't 'burning out.'They're suffering from moral injury. *Stat* 2018
7. Sundararaman L, Chow SY, Elakkumanan LB, et al. Physician Burnout: A Pandemic Uncovered by a Pandemic! *ASA Monitor* 2022;86(1):19-20. doi: 10.1097/01.ASM.0000806016.89323.c0
8. Melnikow J, Padovani A, Miller M. Frontline physician burnout during the COVID-19 pandemic: national survey findings. *BMC Health Services Research* 2022;22(1):365. doi: 10.1186/s12913-022-07728-6
9. Engebretsen KM, Bjorbækmo WS. Naked in the eyes of the public: A phenomenological study of the lived experience of suffering from burnout while waiting for recognition to be ill. *Journal of Evaluation in Clinical Practice* 2019;25(6):1017-26. doi: https://doi.org/10.1111/jep.13244
10. Engebretsen KM. Suffering without a medical diagnosis. A critical view on the biomedical attitudes towards persons suffering from burnout and the implications for medical care. *Journal of Evaluation in Clinical Practice* 2018;24(5):1150-57. doi: https://doi.org/10.1111/jep.12986
11. Heinemann LV, Heinemann T. Burnout Research: Emergence and Scientific Investigation of a Contested Diagnosis. *SAGE Open* 2017;7(1):2158244017697154. doi: 10.1177/2158244017697154
12. Whitehead O, Jagger C, Hanratty B. What do doctors understand by spiritual health? A survey of UK general practitioners. *BMJ Open* 2021;11(8):e045110. doi: 10.1136/bmjopen-2020-045110
13. Goodman MJ, Schorling JB. A Mindfulness Course Decreases Burnout and Improves Well-Being among Healthcare Providers. *The International Journal of Psychiatry in Medicine* 2012;43(2):119-28. doi: 10.2190/PM.43.2.b
14. Ireland MJ, Clough B, Gill K, et al. A randomized controlled trial of mindfulness to reduce stress and burnout among intern medical practitioners. *Medical Teacher* 2017;39(4):409-14. doi: 10.1080/0142159X.2017.1294749
15. Hardiman P, Simmonds JG. Spiritual well-being, burnout and trauma in counsellors and psychotherapists. *Mental Health, Religion & Culture* 2013;16(10):1044-55. doi: 10.1080/13674676.2012.732560
16. Wachholtz A, Rogoff M. The relationship between spirituality and burnout among medical students. *J Contemp Med Educ* 2013;1(2):83-91. doi: 10.5455/jcme.20130104060612
17. Lizano EL, Godoy AJ, Allen N. Spirituality and worker well-being: Examining the relationship between spirituality, job burnout, and work engagement. *Journal of Religion & Spirituality in Social Work: Social Thought* 2019;38(2):197-216. doi: 10.1080/15426432.2019.1577787
18. Cochrane library. Search strategies to identify observational studies in MEDLINE and Embase [Available from: https://www.cochranelibrary.com/cdsr/doi/10.1002/14651858.MR000041.pub2/appendices accessed 27/08/2020.
19. Ouzzani M, Hammady H, Fedorowicz Z, et al. Rayyan—a web and mobile app for systematic reviews. *Systematic Reviews* 2016;5(1):210. doi: 10.1186/s13643-016-0384-4
20. Downes MJ, Brennan ML, Williams HC, et al. Development of a critical appraisal tool to assess the quality of cross-sectional studies (AXIS). *BMJ Open* 2016;6(12):e011458. doi: 10.1136/bmjopen-2016-011458
21. Frank E, McMurray J, Linzer M, et al. Career satisfaction of US women physicians - Results from the Women Physicians' Health Study. *ARCHIVES OF INTERNAL MEDICINE* 1999;159(13):1417-26.
22. Das S, Barman S, Datta S, et al. Degree of burnout among emergency healthcare workers and factors influencing level of burnout: a pilot study. *Delhi Psychiatry Journal* 2016;19:36-47.
23. Baruah A, Das S, Dutta A, et al. DEGREE AND FACTORS OF BURNOUT AMONG EMERGENCY HEALTHCARE WORKERS IN INDIA. *Int J Sci Res (Ahmedabad)* 2019;8(4):41-45. doi: 10.15373/22778179
24. Büssing A, Loetzke D, Gloeckler M, et al. Influence of Spirituality on Cool Down Reactions, Work Engagement, and Life Satisfaction in Anthroposophic Health Care Professionals. *Evidence-Based Complementary and Alternative Medicine* 2015
25. Clark L, Leedy S, McDonald L, et al. Spirituality and job satisfaction among hospice interdisciplinary team members. *Journal of Palliative Medicine* 2007;10(6):1321-28. doi: 10.1089/jpm.2007.0035
26. Guay MD, Reyes Donoso MM, Lopez Saca JM, et al. Spirituality and Religiosity and Burnout in Latin-American Palliative Care Health Care Professionals (LAPC) (S818). *Journal of Pain and Symptom Management* 2019;57(2):490-91.
27. Glebocka A, Lisowska E, Glebocka A, et al. Professional burnout and stress among polish physicians explained by the Hobfoll resources theory. *JOURNAL OF PHYSIOLOGY AND PHARMACOLOGY* 2007;58:243-52.

For peer review only - http://bmjopen.bmj.com/site/about/guidelines.xhtml

28. Koh MYH, Chong PH, Neo PSH, et al. Burnout, psychological morbidity and use of coping mechanisms among palliative care practitioners: A multi-centre cross-sectional study. *Palliative Medicine* 2015;29(7):633-42.
29. Lal A, Tharyan A, Tharyan P. The prevalence, determinants and the role of empathy and religious or spiritual beliefs on job stress, job satisfaction, coping, burnout, and mental health in medical and surgical faculty of a teaching hospital: A cross-sectional survey. *Revue de Medecine Interne* 2020;41(4):232-40.
30. Leonelli LB, Andreoni S, Martins P, et al. Perceived stress among Primary Health Care Professionals in Brazil. *Estresse percebido em profissionais da Estrategia Saude da Familia* 2017;20(2):286-98.
31. Macuka I, Tucak Junakovic I. A Cross-Sectional Study of Job Satisfaction and Intention to Leave Job in Palliative Care in Croatia. *Journal of palliative care* 2021:8258597211046704.
32. Mantri S, Lawson JM, Wang ZZ, et al. Prevalence and Predictors of Moral Injury Symptoms in Health Care Professionals. *JOURNAL OF NERVOUS AND MENTAL DISEASE* 2021;209(3):174-80.
33. Macuka I, Junakovic I, Bozic D, et al. Burnout among Palliative Care Professionals. *DRUSTVENA ISTRAZIVANJA* 2020;29(2):287-308.
34. Ntantana A, Matamis D, Savvidou S, et al. Burnout and job satisfaction of intensive care personnel and the relationship with personality and religious traits: An observational, multicenter, cross-sectional study. *Intensive and Critical Care Nursing* 2017;41:11-17.
35. Purvis T, Saylor D, Powell B, et al. Burnout and Resilience Among Neurosciences Critical Care Unit Staff. *NEUROCRITICAL CARE* 2019;31(2):406-10.
36. See K, Zhao M, Nakataki E, et al. Professional burnout among physicians and nurses in Asian intensive care units: a multinational survey. *INTENSIVE CARE MEDICINE* 2018;44(12):2079-90.
37. Shetach A, Marcus O, Shetach A, et al. The critical managerial capabilities of medical and nursing managers in an Israeli hospital. *EVIDENCE-BASED HRM-A GLOBAL FORUM FOR EMPIRICAL SCHOLARSHIP* 2015;3(1):81-102.
38. Teixeira C, Ribeiro O, Fonseca AM, et al. Burnout in intensive care units - a consideration of the possible prevalence and frequency of new risk factors: A descriptive correlational multicentre study. *BMC Anesthesiology* 2013;13:38.
39. Antonsdottir I, Rushton CH, Nelson KE, et al. Burnout and moral resilience in interdisciplinary healthcare professionals. *Journal of Clinical Nursing* 2022;31(1):196-208.
40. Chor WPD, Ng WM, Cheng L, et al. Burnout amongst emergency healthcare workers during the COVID-19 pandemic: A multi-center study. *American Journal of Emergency Medicine* 2021;46:700-02.
41. Schmidt C, Roffler M. Who isn’t burned out? The role of spirituality in graduate medical education (GME), 2018.
42. Doolittle B, Windish D, Doolittle BR, et al. Correlation of burnout syndrome with specific coping strategies, behaviors, and spiritual attitudes among interns at Yale University, New Haven, USA. *JOURNAL OF EDUCATIONAL EVALUATION FOR HEALTH PROFESSIONS* 2015;12
43. McKinley N, McCain RS, Convie L, et al. Resilience, burnout and coping mechanisms in UK doctors: a cross-sectional study. *BMJ open* 2020;10(1):e031765.
44. Roslan NS, Yusoff MSB, Ab Razak A, et al. Training Characteristics, Personal Factors and Coping Strategies Associated with Burnout in Junior Doctors: A Multi-Center Study. *HEALTHCARE* 2021;9(9)
45. Azoulay E, De Waele J, Ferrer R, et al. Symptoms of burnout in intensive care unit specialists facing the COVID-19 outbreak. *Annals of Intensive Care* 2020;10(1):110.
46. Ben-Itzhak S, Dvash J, Maor M, et al. Sense of meaning as a predictor of burnout in emergency physicians in Israel: a national survey. *Clinical and Experimental Emergency Medicine* 2015;2(4):217-25. doi: 10.15441/ceem.15.074
47. Correia I, Almeida AE. Organizational Justice, Professional Identification, Empathy, and Meaningful Work During COVID-19 Pandemic: Are They Burnout Protectors in Physicians and Nurses? *FRONTIERS IN PSYCHOLOGY* 2020;11
48. Doolittle BR. Association of Burnout with Emotional Coping Strategies, Friendship, and Institutional Support Among Internal Medicine Physicians. *JOURNAL OF CLINICAL PSYCHOLOGY IN MEDICAL SETTINGS* 2020
49. Doolittle BR, Windish DM, Seelig CB. Burnout, coping, and spirituality among internal medicine resident physicians. *J Grad Med Educ* 2013;5(2):257-61. doi: 10.4300/JGME-D-12-00136.1
50. Gribben J, Kase S, Waldman E, et al. A Cross-Sectional Analysis of Compassion Fatigue, Burnout, and Compassion Satisfaction in Pediatric Critical Care Physicians in the United States*. *PEDIATRIC CRITICAL CARE MEDICINE* 2019;20(3):213-22.

For peer review only - http://bmjopen.bmj.com/site/about/guidelines.xhtml

51. Ntantana A, Matamis D, Savvidou S, et al. Burnout and job satisfaction of intensive care personnel and the relationship with personality and religious traits: An observational, multicenter, cross-sectional study. *Intensive and Critical Care Nursing* 2017;41:11-17. doi: 10.1016/j.iccn.2017.02.009
52. Salmoirago-Blotcher E, Fitchett G, Leung K, et al. An exploration of the role of religion/spirituality in the promotion of physicians' wellbeing in Emergency Medicine. *Preventive Medicine Reports* 2016;3:189-95.
53. St Onge JE, Allespach H, Diaz Y, et al. Burnout: exploring the differences between U.S. and international medical graduates. *BMC medical education* 2022;22(1):69.
54. Watson A, Saggar V, MacDowell C, et al. Self-reported modifying effects of resilience factors on perceptions of workload, patient outcomes, and burnout in physician-attendees of an international emergency medicine conference. *PSYCHOLOGY HEALTH & MEDICINE* 2019;24(10):1220-34.
55. Mantri S, Lawson J, Wang Z, et al. Identifying Moral Injury in Healthcare Professionals: The Moral Injury Symptom Scale-HP. *JOURNAL OF RELIGION & HEALTH* 2021
56. Guest RS, Baser R, Li Y, et al. Cancer surgeons' distress and well-being, II: modifiable factors and the potential for organizational interventions. *Annals of surgical oncology* 2011;18(5):1236-42.
57. Harper L, Alshammari D, Ferdynus C, et al. Burnout amongst members of the French-speaking Society of Pediatric and Adolescent Urology (SFUPA). Are there specific risk factors? *Journal of pediatric urology* 2020;16(4):482-86.
58. Ramondetta L, Urbauer D, Brown A, et al. Work related stress among gynecologic oncologists. *GYNECOLOGIC ONCOLOGY* 2011;123(2):365-69.
59. Suttle M, Chase M, Sasser W, et al. Burnout in Pediatric Critical Care Medicine Fellows*. *CRITICAL CARE MEDICINE* 2020;48(6):872-80.
60. Yoon J, Rasinski K, Curlin F, et al. Conflict and emotional exhaustion in obstetrician-gynaecologists: a national survey. *JOURNAL OF MEDICAL ETHICS* 2010;36(12):731-35.
61. Leu S, Vuille-dit-Bille R, Fink L, et al. Burnout in Swiss and Australian surgeons in training-a cross-sectional study. *EUROPEAN SURGERY-ACTA CHIRURGICA AUSTRIACA*
62. Schmidt C, Roffler M. Coping with the Practice of Medicine: Religion, Spirituality, and Other Personal Strategies. *JOURNAL OF RELIGION & HEALTH* 2021;60(3):2092-108.
63. Alosaimi FD, Alawad HS, Alamri AK, et al. Stress and coping among consultant physicians working in Saudi Arabia. *Annals of Saudi Medicine* 2018;38(3):214-24. doi: 10.5144/0256-4947.2018.214
64. Glasberg J, Horiuti L, Novais M, et al. Prevalence of the burnout syndrome among Brazilian medical oncologists. *REVISTA DA ASSOCIACAO MEDICA BRASILEIRA* 2007;53(1):85-89.
65. Ramondetta LM, Urbauer D, Brown AJ, et al. Work related stress among gynecologic oncologists. *GYNECOLOGIC ONCOLOGY* 2011;123(2):365-69. doi: 10.1016/j.ygyno.2011.06.010
66. Büssing A, Lotzke D, Glockler M, et al. Influence of Spirituality on Cool Down Reactions, Work Engagement, and Life Satisfaction in Anthroposophic Health Care Professionals. *Evidence-Based Complementary and Alternative Medicine* 2015 doi: 10.1155/2015/754814
67. Doolittle BR, Windish DM. Correlation of burnout syndrome with specific coping strategies, behaviors, and spiritual attitudes among interns at Yale University, New Haven, USA. *Journal of Educational Evaluation for Health Professions* 2015;12 doi: 10.3352/jeehp.2015.12.41
68. Lal A, Tharyan A, Tharyan P. The prevalence, determinants and the role of empathy and religious or spiritual beliefs on job stress, job satisfaction, coping, burnout, and mental health in medical and surgical faculty of a teaching hospital: A cross-sectional survey. *La Revue de Médecine Interne* 2020 doi: https://doi.org/10.1016/j.revmed.2019.12.005
69. McKinley NC. Resilience, professional quality of life and coping mechanisms in doctors and medical students. Queens University Belfast, 2021.
70. Watson AG, Saggar V, MacDowell C, et al. Self-reported modifying effects of resilience factors on perceptions of workload, patient outcomes, and burnout in physician-attendees of an international emergency medicine conference. *Psychology, health & medicine* 2019;24(10):1220-34.
71. Büssing A, Ostermann T, Matthiessen PF. Role of religion and spirituality in medical patients: confirmatory results with the SpREUK questionnaire. *Health and quality of life outcomes* 2005;3(1):10.
72. Maslach C, Leiter MP. The Truth About Burnout: How Organizations Cause Personal Stress and What to Do About It: Wiley 2008.
73. Li-Sauerwine S, Rebillot K, Melamed M, et al. A 2-Question Summative Score Correlates with the Maslach Burnout Inventory. *West J Emerg Med* 2020;21(3):610-17. doi: 10.5811/westjem.2020.2.45139
74. Demerouti E, Bakker AB. The Oldenburg Burnout Inventory: A good alternative to measure burnout and engagement. *Handbook of stress and burnout in health care* 2008:65-78.
75. Kristensen TS, Borritz M, Villadsen E, et al. Copenhagen burnout inventory. *Work & Stress* 1999
76. Winwood PC, Winefield AH. Comparing Two Measures of Burnout Among Dentists in Australia. *International Journal of Stress Management* 2004;11(3):282-89. doi: 10.1037/1072-5245.11.3.282

For peer review only

For peer review only - http://bmjopen.bmj.com/site/about/guidelines.xhtml

77. Swami M, Mathur D, Pushp B. Emotional intelligence, perceived stress and burnout among resident doctors: an assessment of the relationship. *The National medical journal of India* 2013;26(4):210-13.

78. Teles R, Valle A, Rodríguez S, et al. Perceived Stress and Indicators of Burnout in Teachers at Portuguese Higher Education Institutions (HEI). *Int J Environ Res Public Health* 2020;17(9):3248. doi: 10.3390/ijerph17093248

79. Ben-Itzhak S, Dvash J, Maor M, et al. Sense of meaning as a predictor of burnout in emergency physicians in Israel: a national survey. *CLINICAL AND EXPERIMENTAL EMERGENCY MEDICINE* 2015;2(4):217-25.

80. Facit.org. FACIT-Sp Non-Illness [Available from: https://www.facit.org/measures/FACIT-Sp-Non-Illness accessed 02/07/2021.

81. Hungelmann J, Kenkel-Rossi E, Klassen L, et al. Focus on Spiritual Well-Being: Harmonious interconnectedness of mind-body-spirit—Use of the JAREL Spiritual Well-Being Scale: Assessment of spiritual well-being is essential to the health of individuals. *Geriatric Nursing* 1996;17(6):262-66. doi: https://doi.org/10.1016/S0197-4572(96)80238-2

82. Hatch RL, Burg MA, Naberhaus DS, et al. The spiritual involvement and beliefs scale. *Journal of Family Practice* 1998;46(6):476-86.

83. Büssing A, Hirdes AT, Baumann K, et al. Aspects of Spirituality in Medical Doctors and Their Relation to Specific Views of Illness and Dealing with Their Patients' Individual Situation. *Evidence-Based Complementary and Alternative Medicine* 2013;2013:734392. doi: 10.1155/2013/734392

84. Institute F. Multidimensional Measurement of Religiousness/Spirituality for Use in Health Research [Available from: https://fetzer.org/resources/multidimensional-measurement-religiousnessspirituality-use-health-research.

85. Daaleman TP, Frey BB, Wallace D, et al. The Spirituality Index of Well-Being: Development and testing of a new measure. *J Fam Pract* 2002;51(11):952.

86. Monod S, Brennan M, Rochat E, et al. Instruments Measuring Spirituality in Clinical Research: A Systematic Review. *Journal of General Internal Medicine* 2011;26(11):1345. doi: 10.1007/s11606-011-1769-7

87. Carver CS. You want to measure coping but your protocol's too long: consider the brief COPE. *Int J Behav Med* 1997;4(1):92-100. doi: 10.1207/s15327558ijbm0401_6 [published Online First: 1997/01/01]

88. Hobfoll SE. Conservation of Resources: A New Attempt at Conceptualizing Stress. *The American psychologist* 1989;44(3):513-24. doi: 10.1037/0003-066X.44.3.513

89. Koenig H, Parkerson Jr GR, Meador KG. Religion index for psychiatric research. *The American Journal of Psychiatry* 1997;154(6):885-86. doi: 10.1176/ajp.154.6.885b

90. Zgliczyńska M, Zgliczyński S, Ciebiera M, et al. Occupational Burnout Syndrome in Polish Physicians: A Systematic Review. *Int J Environ Res Public Health* 2019;16(24) doi: 10.3390/ijerph16245026

91. Williams ES, Rathert C, Buttigieg SC. The Personal and Professional Consequences of Physician Burnout: A Systematic Review of the Literature. *Medical Care Research and Review* 2019;77(5):371-86. doi: 10.1177/1077558719856787

92. Balendran B, Bath MF, Awopetu AI, et al. Burnout within UK surgical specialties: a systematic review. *The Annals of The Royal College of Surgeons of England* 2021;103(7):464-70. doi: 10.1308/rcsann.2020.7058

93. Chow HHE, Chew QH, Sim K. Spirituality and religion in residents and inter-relationships with clinical practice and residency training: A scoping review. *BMJ Open* 2021;11(5):e044321. doi: http://dx.doi.org/10.1136/bmjopen-2020-044321

94. Sibeoni J, Bellon-Champel L, Mousty A, et al. Physicians' Perspectives About Burnout: a Systematic Review and Metasynthesis. *Journal of General Internal Medicine* 2019;34(8):1578-90. doi: 10.1007/s11606-019-05062-y

95. Kang MJ, Park JH. The effects of COVID-19 on physician's burnout: a systematic review. *J Korean Med Assoc* 2021;64(9):636-46. doi: 10.5124/jkma.2021.64.9.636

96. Jefferson L, Golder S, Heathcote C, et al. GP wellbeing during the COVID-19 pandemic: a systematic review. *British Journal of General Practice* 2022;72(718):e325. doi: 10.3399/BJGP.2021.0680

97. Williamson K, Lank PM, Cheema N, et al. Comparing the Maslach Burnout Inventory to Other Well-Being Instruments in Emergency Medicine Residents. *J Grad Med Educ* 2018;10(5):532-36. doi: 10.4300/jgme-d-18-00155.1 [published Online First: 2018/11/06]

98. de Jager Meezenbroek E, Garssen B, van den Berg M, et al. Measuring Spirituality as a Universal Human Experience: A Review of Spirituality Questionnaires. *Journal of Religion and Health* 2012;51(2):336-54. doi: 10.1007/s10943-010-9376-1

99. Lu J, Lo C-L, Bang NM, et al. A Critical Review of Spiritual Well-Being Scales: Implications for Research, Practice, and Education in Rehabilitation Counseling. *Rehabilitation Counseling Bulletin* 2018;62(3):144-56. doi: 10.1177/0034355218776764

For peer review only - http://bmjopen.bmj.com/site/about/guidelines.xhtml

100. Assing Hvidt E, Søndergaard J, Hansen DG, et al. 'We are the barriers': Danish general practitioners' interpretations of why the existential and spiritual dimensions are neglected in patient care. *Commun Med* 2017;14(2):108-20. doi: 10.1558/cam.32147 [published Online First: 2017/01/01]

101. Scheepers RA, Emke H, Epstein RM, et al. The impact of mindfulness-based interventions on doctors' well-being and performance: A systematic review. *Medical Education* 2020;54(2):138-49. doi: https://doi.org/10.1111/medu.14020

102. Nightingale S, Spiby H, Sheen K, et al. The impact of emotional intelligence in health care professionals on caring behaviour towards patients in clinical and long-term care settings: Findings from an integrative review. *Int J Nurs Stud* 2017;80:106-17.

Search terms:

1. spiritual*.mp. or exp religion/ or religio*.mp. or religiosity.mp. or exp philosophy/ or exp spiritual healing/ or theology/ or sikh.mp. or jain.mp. or bahai.mp. or pagan.mp. or shinto.mp. or cao dai.mp. or zoroast*.mp. or spirit*.mp. or Faith.mp. or holistic health.mp. or existential.mp. or salutogenesis.mp. or religious personnel/ or chaplain*.mp. or Buddhism.mp. or Christian*.mp. or Hinduism.mp. or islam.mp. or Judaism.mp. or humanism.mp. or Confucian*.mp. or "sense of coherence".mp.
2. exp job stress/ or compassion fatigue.mp. or occupational stress.mp. or burnout.mp. or exp burnout/ or exp Maslach Burnout Inventory/ or "work related

For peer review only

For peer review only - http://bmjopen.bmj.com/site/about/guidelines.xhtml

	stress*".mp. or "occupational stress*".mp. or "work stress*".mp. or "job stress".mp. or Job Satisfaction/ or "job satisfaction".mp. or moral injury.mp. or burn&out.mp.
3.	exp medical personnel/ or medical personnel.mp. or physician$.mp. or allergist$.mp. or cardiologists.mp. or dermatologists.mp. or endocrinologists.mp. or gastroenterologists.mp. or general practitioner$.mp. or geriatrician$.mp. or nephrologist$.mp. or neurologist$.mp. or occupational health physician$.mp. or oncologist$.mp. or ophthalmologist$.mp. or pathologist$.mp. or p$ediatrician$.mp. or neonatologist$.mp. or physiatrist$.mp. or pulmonologist$.mp. or psychiatrist$.mp. or radiologist$.mp. or rheumatologist$.mp. or surgeon$.mp. or urologist$.mp. or doctor*.mp. or medical staff.mp. or an$esthetist$.mp. or medical school/ or medical faculty.mp.

For peer review only

4. epidemiology/ or exp clinical study/ or cohort analysis/ or case study/ or follow up/ or cross-sectional study/ or observational study/ or correlational study/ or epidemiologic.ab,ti. Or case control.ab,ti. Or case referent.ab,ti. Or case stud$.ab,ti. Or case series.ab,ti. Or cohort?.ab,ti. Or cross sectional.ab,ti. Or follow up.ab,ti. Or longitudinal.ab,ti. Or retrospective$.ab,ti. Or prospective$.ab,ti. Or observational.ab,ti. Or adverse effect?.ab,ti. or (Controlled before and after).ab,ti. Or Interrupted time series.ab,ti. or Correlational.ab,ti. Or ecological stud$.ab,ti. or Descriptive stud$.ab,ti.

List of figures

Figure 1: Prisma Diagram[102]

Figure 2: Medical specialty of participants

Figure 3: Geographical location of participants of studies included in this review

FIGURE 2:

For peer review only

For peer review only - http://bmjopen.bmj.com/site/about/guidelines.xhtml

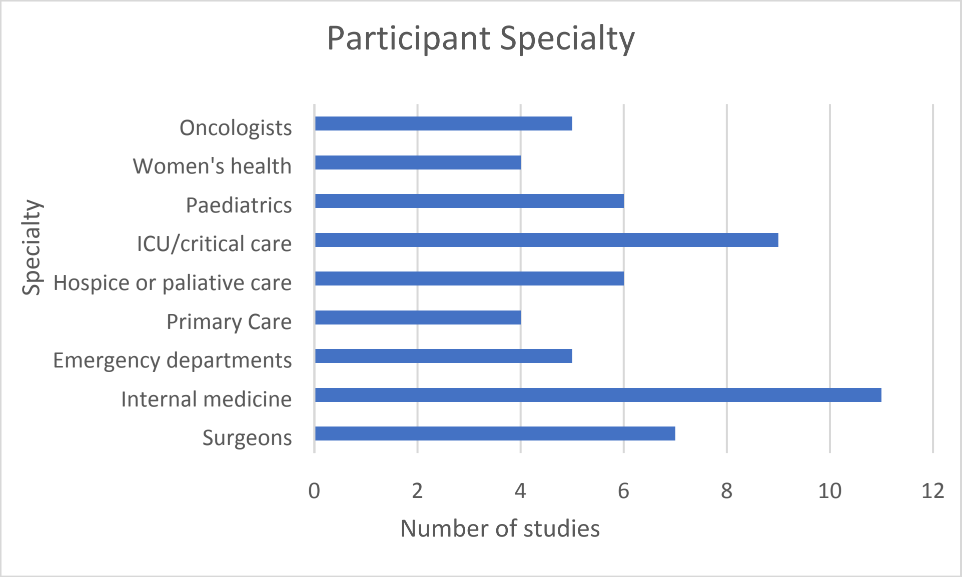

FIGURE 3:

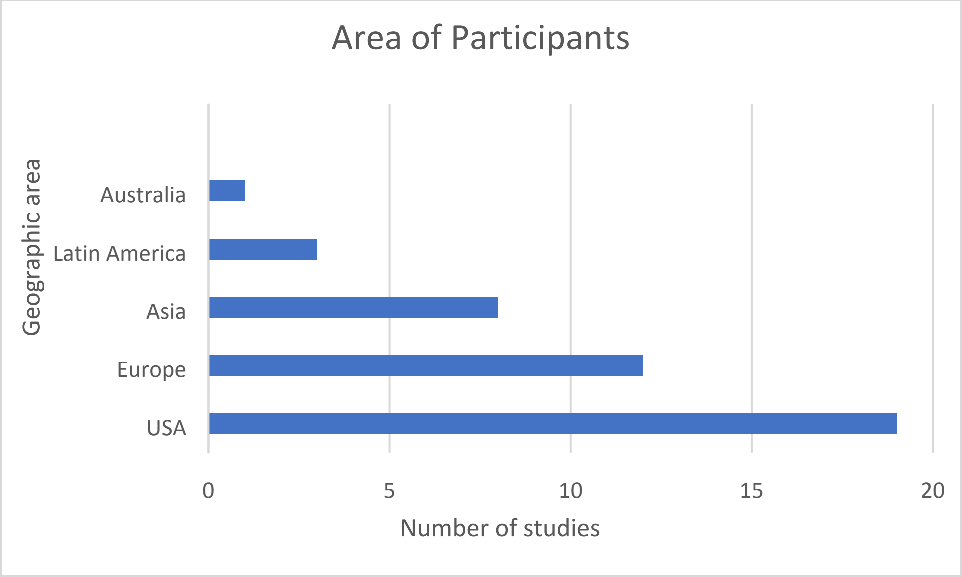

For peer review only - http://bmjopen.bmj.com/site/about/guidelines.xhtml

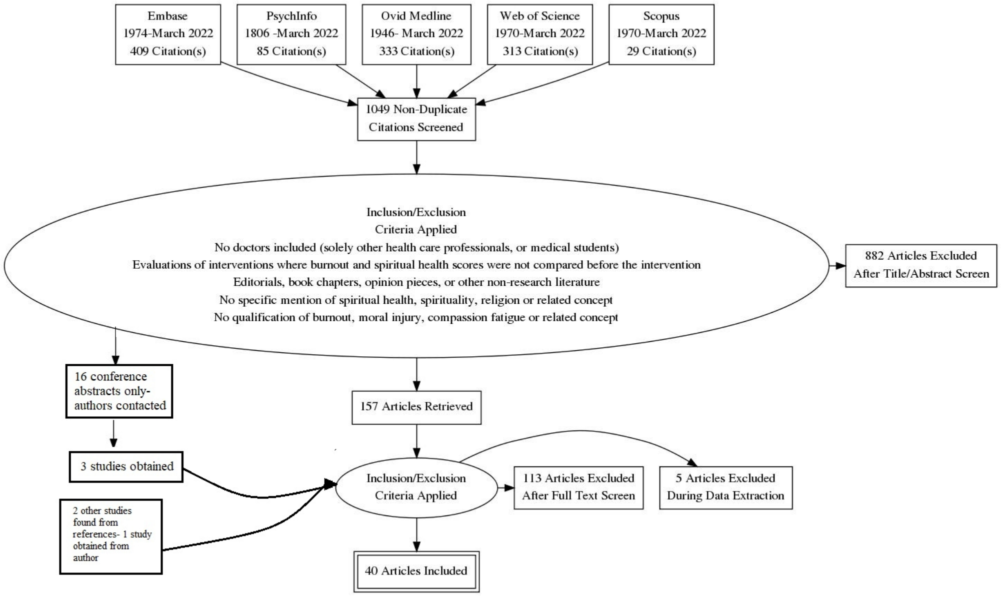

Figure 1: Prisma Diagram

383x227mm (96 x 96 DPI)

Systematic Review Protocol & Support Template

**Title of the review**	A systematic review: Is there a relationship between burnout scores in doctors and their spiritual health
**First reviewer**	Orla Whitehead
**Team of reviewers**	Ishbel Orla Whitehead,<br>Suzanne Moffatt,<br>Stephanie Warwick,<br>Gemma Frances<br>Spiersa, Tafadzwa<br>Patience Kunonga,<br>Eugene Yee Hing Tang,<br>Barbara Hanratty
**Supervisor/Project PI**	Professor Barbara<br>Hanratty
**Clinical Portfolio Group**	
**Project title (if different from review title)**	

**Support** – please state if advice/training or personnel required at each stage	
**SR overview**	
**Protocol development**	“”
**Literature searching**	Advice taken from Catherine Richmond, information scientist at the University
**Quality appraisal**	
**Data Extraction**	“”
**Synthesis**	“”
**Writing up**	“”

For peer review only - http://bmjopen.bmj.com/site/about/guidelines.xhtml

**1. Background to review**<br>Brief introduction to the subject of the review, including rationale for undertaking the review and overall aim
Burnout and moral injury are two terms used to describe a form of psychological distress associated with work as a doctor. Burnout is defined by the World Health Organisation not as an illness, but as an occupational phenomenon, resulting in exhaustion, mental distance or feelings of negativity or cynicism towards the job, and reduced professional efficacy.[1] Others describe a loss of meaning in work, and objectification of patients and their families, rather than engaging with their humanity.[2] Moral injury is a term taken from military personnel returning from battle, where the realties conflict with our moral and ethical code.[3] It has been argued that doctors caught between patient and societal demands, limited resources, and their own ethical code and training, are suffering injury analogous to soldiers, leaving them wounded and hopeless.[3] Reframing burnout as moral injury replaces a view of a defective individual, to that of a defective system, which could be more effective in terms of change[4], and move the dialogue away from expecting doctors to become more and more resilient in the face of increasing challenges. The co-option of the term from conflict has been critised[5], and arguments made that while moral injury can occur in medicine, burn out is not synonymous, and is more common.[6] While both the terms burnout and moral injury place the issue in the context of occupation, it is evident that mental illness accompanies these issues, as suicide rates for female doctors are up to 4 times those of age-matched controls.[7] With current recruitment and retention issues, we cannot afford to lose good doctors to burnout.[2] Supporting doctors' holistic health has to become a priority, to help prevent and treat burnout and moral injury.<br><br>Holistic health includes spiritual health. Literature discussing how doctors should address their patients' spiritual health often mention that self awareness of spiritual health, needs, and distress, is key to be able to begin to meet patients' needs in this area. In a recent survey of GPs in England, 69% described themselves as 'a spiritual person', with 48% also describing themselves as 'a religious person'. These GPs defined spiritual health as self-actualisation and meaning, transcendence and relationships beyond the self, and expressions of spirituality. Self-actualisation included concepts of being able to follow their personal ethical and moral codes. Meaning included having personal meaning to life and relationships. Transcendence included concepts of a spiritual sphere, a concept of a soul, and relationships with communities, friends, family, nature and/or the divine. Expressions of spirituality included both personal and group religious and spiritual practice, for example attendance at church, religious meetings, meditation, prayer and mindfulness practice. Burnout could be seen as contrasting with these definitions of spiritual health, as mental distance from others and the job, negativity and lack of meaning in the job, and lack of relationships with patients and their families. Spiritual health as defined by GPs requires meaning, following their ethical code. Could burnout and spiritual distress among doctors be related phenomena? Viewing burnout through the lens of spiritual health and distress, as well as other lenses and perspectives, could generate potential beneficial interventions for both prevention and treatment of burnout.<br><br>Aims:<br>To investigate the association between burnout in doctors and spiritual health, by conducting a systematic review of studies which use a measure burnout with measures of aspects of spiritual health, spirituality, religiosity or spiritual distress.

For peer review only - http://bmjopen.bmj.com/site/about/guidelines.xhtml

**2. Specific objectives**
1. To investigate the quantity and quality of data addressing doctors' burnout, and spiritual heath<br>2. To investigate any link between doctors' burnout and spiritual health, spirituality, spiritual distress or religiosity.

For peer review only

For peer review only - http://bmjopen.bmj.com/site/about/guidelines.xhtml

**3. a) Criteria for including studies in the review**<br>If the PICOS format does not fit the research question of interest, please split up the question into separate concepts and put one under each heading	
**i. Population, or participants and conditions of interest**	Qualified doctors/medical practitioners, worldwide of either sex and any age, whether they have experienced any level of burnout or not.
**ii. Interventions or exposures**	Quantification of burnout (for example, use of a burnout inventory such as the Maslach Burnout Inventory, or the Copenhagen Burnout Inventory, or others)
**iii. Comparisons or control groups**	Quantification of spiritual health, spirituality, spiritual distress, or religiosity, either using a scale (for example the Spiritual Wellbeing Scale, Spiritual Distress Scale, or other measures of spiritual health or religiosity) or a yes/no or self-rating of 'spirituality' or religiosity.
**iv. Outcomes of interest**	Associations between spiritual health, spiritual distress or religiosity with burnout scores.
**v. Setting**	Any setting where doctors/medical practitioners work (primary, secondary or tertiary care, including laboratory or other non-patient facing settings)
**vi. Study designs**	All research studies with a quantification of burnout, and spiritual health, wellbeing, distress or religiosity, or similar.

**3. b) Criteria for excluding studies not covered in inclusion criteria**<br>Any specific populations excluded, date range, language, whether abstracts or full text available, etc
Medical students, nursing staff, or other hospital staff where doctors are not included.<br>Course evaluations<br>Editorials, book chapters, or other non-research study articles<br>No specific mention of spiritual health, spirituality, or religiosity.<br>No quantification of burnout, or spiritual health, wellbeing or distress, or similar.

For peer review only

**4. Search methods**	
**Electronic databases**<br>Please list all databases that are to be searched and include the interface (eg NHS, EBSCO, etc) and date ranges searched for each	PUBMED/MEDLINE<br>EMBASE<br>PsychInfo<br>Web of Science<br>Scopus
**Other methods used for identifying relevant research**<br>ie contacting experts and reference checking	Reference checking and hand searching of these. Contacting experts in this field.
**Journals hand searched**<br>If any are to be hand searched, please list which journals and date searched from, including a rationale.	

**5. Methods of review**	
**Details of methods**<br>Number of reviewers, how agreements to be reached and disagreements dealt with, etc.	Data extraction will be carried out by the primary reviewer, with 10% checked by a second reviewer
**Quality assessment**<br>Tools or checklists used with references or URLs	Appraisal of Cross-sectional Studies tool (AXIS).[8]

For peer review only - http://bmjopen.bmj.com/site/about/guidelines.xhtml

**Data extraction** What information is to be collected on each included study. If databases or forms on Word or Excel are used and how this is recorded and by how many reviewers	Custom made word table to be used
**Narrative synthesis** Details of what and how synthesis will be done	Synthesis of findings regarding the research question (are burnout and spiritual health related)<br>Synthesis of measures used for burnout, and spiritual health
**Meta-analysis** Details of what and how analysis and testing will be done. If no meta-analysis is to be conducted, please give reason.	If possible, meta-analysis will be conducted of the relationship between burnout scores (e.g. the Maslach Burnout Inventory) and spiritual health scores (e.g. the Brief COPE). If different validated scores are used, effect sizes will be compared.
**Grading evidence** System used, if any, such as GRADE	

**6. Presentation of results**	
**Additional material** Summary tables, flowcharts, etc, to be included in the final paper	
**Outputs from review** Papers and target journals, conference presentations, reports, etc	Conference presentation at the Society for Academic Primary Care Annual Scientific Meeting<br>Submission to journal

For peer review only

7. Timeline for review – when do you aim to complete each stage of the review	
**Protocol**	July 2020
**Literature searching**	July 2020
**Quality appraisal**	August 2020
**Data extraction**	September 2020
**Synthesis**	September 2020
**Writing up**	October 2020

1. World Heath Organisation. Burn-out an "occupational phenomenon": International Classification of Diseases 2019 [Available from: https://www.who.int/mental_health/evidence/burn-out/en/.
2. Fred HL, Scheid MS. Physician Burnout: Causes, Consequences, and (?) Cures. *Tex Heart Inst J* 2018;45(4):198-202. doi: 10.14503/THIJ-18-6842
3. Talbot SG, Dean W. Physicians aren't 'burning out.'They're suffering from moral injury. *Stat* 2018
4. Dean W, Talbot S, Dean A. Reframing Clinician Distress: Moral Injury Not Burnout. *Fed Pract* 2019;36(9):400-02.
5. Asken MJ. Physician burnout: moral injury is a questionable term. *BMJ* 2019;365:l2375. doi: 10.1136/bmj.l2375
6. Parker J. Moral injury and burnout are not the same 2019 [Available from: https://blogs.bmj.com/bmj/2019/06/06/joshua-parker-moral-injury-and-burnout-are-not-the-same/ accessed 11/12/2019.
7. Practitioner Health Programme. Suicide in doctors and other health professionals 2019 [Available from: https://php.nhs.uk/resources/suicide-in-doctors/ accessed 11/12/2019.
8. Downes MJ, Brennan ML, Williams HC, et al. Development of a critical appraisal tool to assess the quality of cross-sectional studies (AXIS). *BMJ Open* 2016;6(12):e011458. doi: 10.1136/bmjopen-2016-011458

For peer review only - http://bmjopen.bmj.com/site/about/guidelines.xhtml

PRISMA 2009 Checklist

Section/topic	#	Checklist item	Reported on page #
**TITLE**			
Title	1	Identify the report as a systematic review, meta-analysis, or both.	1
**ABSTRACT**			
Structured summary	2	Provide a structured summary including, as applicable: background; objectives; data sources; study eligibility criteria, participants, and interventions; study appraisal and synthesis methods; results; limitations; conclusions and implications of key findings; systematic review registration number.	1, 2
**INTRODUCTION**			
Rationale	3	Describe the rationale for the review in the context of what is already known.	3,4
Objectives	4	Provide an explicit statement of questions being addressed with reference to participants, interventions, comparisons, outcomes, and study design (PICOS).	4
**METHODS**			
Protocol and registration	5	Indicate if a review protocol exists, if and where it can be accessed (e.g., Web address), and, if available, provide registration information including registration number.	5
Eligibility criteria	6	Specify study characteristics (e.g., PICOS, length of follow-up) and report characteristics (e.g., years considered, language, publication status) used as criteria for eligibility, giving rationale.	4
Information sources	7	Describe all information sources (e.g., databases with dates of coverage, contact with study authors to identify additional studies) in the search and date last searched.	4
Search	8	Present full electronic search strategy for at least one database, including any limits used, such that it could be repeated.	4, 29,30
Study selection	9	State the process for selecting studies (i.e., screening, eligibility, included in systematic review, and, if applicable, included in the meta-analysis).	5
Data collection process	10	Describe method of data extraction from reports (e.g., piloted forms, independently, in duplicate) and any processes for obtaining and confirming data from investigators.	5
Data items	11	List and define all variables for which data were sought (e.g., PICOS, funding sources) and any assumptions and simplifications made.	5
Risk of bias in individual studies	12	Describe methods used for assessing risk of bias of individual studies (including specification of whether this was done at the study or outcome level), and how this information is to be used in any data synthesis.	5
Summary measures	13	State the principal summary measures (e.g., risk ratio, difference in means).	N/A

For peer review only - http://bmjopen.bmj.com/site/about/guidelines.xhtml

Synthesis of results	14	Describe the methods of handling data and combining results of studies, if done, including measures of consistency (e.g., I^2) for each meta-analysis.	5

PRISMA 2009 Checklist

Section/topic	#	Checklist item	Reported on page #
Risk of bias across studies	15	Specify any assessment of risk of bias that may affect the cumulative evidence (e.g., publication bias, selective reporting within studies).	4,5
Additional analyses	16	Describe methods of additional analyses (e.g., sensitivity or subgroup analyses, meta-regression), if done, indicating which were pre-specified.	n/a
**RESULTS**			
Study selection	17	Give numbers of studies screened, assessed for eligibility, and included in the review, with reasons for exclusions at each stage, ideally with a flow diagram.	6
Study characteristics	18	For each study, present characteristics for which data were extracted (e.g., study size, PICOS, follow-up period) and provide the citations.	5-20
Risk of bias within studies	19	Present data on risk of bias of each study and, if available, any outcome level assessment (see item 12).	6-7, 15-20
Results of individual studies	20	For all outcomes considered (benefits or harms), present, for each study: (a) simple summary data for each intervention group (b) effect estimates and confidence intervals, ideally with a forest plot.	Table 1
Synthesis of results	21	Present results of each meta-analysis done, including confidence intervals and measures of consistency.	Narrative synthesis 7-21
Risk of bias across studies	22	Present results of any assessment of risk of bias across studies (see Item 15).	7, 20-21
Additional analysis	23	Give results of additional analyses, if done (e.g., sensitivity or subgroup analyses, meta-regression [see Item 16]).	7-8
**DISCUSSION**			
Summary of evidence	24	Summarize the main findings including the strength of evidence for each main outcome; consider their relevance to key groups (e.g., healthcare providers, users, and policy makers).	21-23
Limitations	25	Discuss limitations at study and outcome level (e.g., risk of bias), and at review-level (e.g., incomplete retrieval of identified research, reporting bias).	22-23
Conclusions	26	Provide a general interpretation of the results in the context of other evidence, and implications for future research.	24
**FUNDING**			

For peer review only

Funding	27	Describe sources of funding for the systematic review and other support (e.g., supply of data); role of funders for the systematic review.	1

From: Moher D, Liberati A, Tetzlaff J, Altman DG, The PRISMA Group (2009). Preferred Reporting Items for Systematic Reviews and Meta-Analyses: The PRISMA Statement. PLoS Med 6(7): e1000097. doi:10.1371/journal.pmed1000097

For more information, visit: **www.prisma-statement.org**.

For peer review only

For peer review only - http://bmjopen.bmj.com/site/about/guidelines.xhtml

PRISMA

PRISMA 2020 for Abstracts Checklist

Section and Topic	Item #	Checklist item	Reported (Yes/No)
**TITLE**			
Title	1	Identify the report as a systematic review.	yes
**BACKGROUND**			
Objectives	2	Provide an explicit statement of the main objective(s) or question(s) the review addresses.	yes
**METHODS**			
Eligibility criteria	3	Specify the inclusion and exclusion criteria for the review.	yes
Information sources	4	Specify the information sources (e.g. databases, registers) used to identify studies and the date when each was last searched.	yes
Risk of bias	5	Specify the methods used to assess risk of bias in the included studies.	yes
Synthesis of results	6	Specify the methods used to present and synthesise results.	yes
**RESULTS**			
Included studies	7	Give the total number of included studies and participants and summarise relevant characteristics of studies.	yes
Synthesis of results	8	Present results for main outcomes, preferably indicating the number of included studies and participants for each. If meta-analysis was done, report the summary estimate and confidence/credible interval. If comparing groups, indicate the direction of the effect (i.e. which group is favoured).	yes
**DISCUSSION**			
Limitations of evidence	9	Provide a brief summary of the limitations of the evidence included in the review (e.g. study risk of bias, inconsistency and imprecision).	yes
Interpretation	10	Provide a general interpretation of the results and important implications.	yes
**OTHER**			
Funding	11	Specify the primary source of funding for the review.	yes
Registration	12	Provide the register name and registration number.	yes

For peer review only

From: Page MJ, McKenzie JE, Bossuyt PM, Boutron I, Hoffmann TC, Mulrow CD, et al. The PRISMA 2020 statement: an updated guideline for reporting systematic reviews. BMJ 2021;372:n71. doi: 10.1136/bmj.n71

For more information, visit: http://www.prisma-statement.org/

For peer review only

A systematic review of the relationship between burnout and spiritual health in doctors

Journal:	*BMJ Open*
Manuscript ID	bmjopen-2022-068402.R1
Article Type:	Original research
Date Submitted by the Author:	16-Mar-2023
Complete List of Authors:	Whitehead, Ishbel Orla; Newcastle University Population Health Sciences Institute, Biomedical Research Building<br>Moffatt, Suzanne; Newcastle University Population Health Sciences Institute<br>Warwick, Stephanie; Newcastle University Population Health Sciences Institute, Biomedical Research Building<br>Spiers, Gemma; Newcastle University, Population & Health Sciences Institute<br>Kunonga, Tafadzwa; Newcastle University Population Health Sciences Institute<br>Tang, Eugene; Newcastle University Faculty of Medical Sciences<br>Hanratty, Barbara; Newcastle University Population Health Sciences Institute
**Primary Subject Heading**:	General practice / Family practice
Secondary Subject Heading:	Occupational and environmental medicine, Public health
Keywords:	Human resource management < HEALTH SERVICES ADMINISTRATION & MANAGEMENT, OCCUPATIONAL & INDUSTRIAL MEDICINE, PUBLIC HEALTH, Primary Care < Primary Health Care, Primary Health Care

I, the Submitting Author has the right to grant and does grant on behalf of all authors of the Work (as defined in the below author licence), an exclusive licence and/or a non-exclusive licence for contributions from authors who are: i) UK Crown employees; ii) where BMJ has agreed a CC-BY licence shall apply, and/or iii) in accordance with the terms applicable for US Federal Government officers or employees acting as part of their official duties; on a worldwide, perpetual, irrevocable, royalty-free basis to BMJ Publishing Group Ltd ("BMJ") its licensees and where the relevant Journal is co-owned by BMJ to the co-owners of the Journal, to publish the Work in this journal and any other BMJ products and to exploit all rights, as set out in our licence.

The Submitting Author accepts and understands that any supply made under these terms is made by BMJ to the Submitting Author unless you are acting as an employee on behalf of your employer or a postgraduate student of an affiliated institution which is paying any applicable article publishing charge ("APC") for Open Access articles. Where the Submitting Author wishes to make the Work available on an Open Access basis (and intends to pay the relevant APC), the terms of reuse of such Open Access shall be governed by a Creative Commons licence – details of these licences and which Creative Commons licence will apply to this Work are set out in our licence referred to above.

Other than as permitted in any relevant BMJ Author's Self Archiving Policies, I confirm this Work has not been accepted for publication elsewhere, is not being considered for publication elsewhere and does not duplicate material already published. I confirm all authors consent to publication of this Work and authorise the granting of this licence.

For peer review only

A systematic review of the relationship between burnout and spiritual health in doctors

Short title: Doctors' burnout and spiritual health- a systematic review

Ishbel Orla Whitehead[a], Suzanne Moffatt[b], Stephanie Warwick[a], Gemma Frances Spiers[a], Tafadzwa Patience Kunonga[a], Eugene Yee Hing Tang[a], Barbara Hanratty[a]

[a]Population Health Sciences Institute, Faculty of Medical Sciences, Newcastle University, Biomedical Research Building, Campus for Ageing and Vitality, Newcastle upon Tyne, NE4 5PL

[b] Population and Health Sciences Institute, Newcastle University, Baddiley-Clark, Newcastle upon Tyne, NE2 4AX

Corresponding author: Orla Whitehead. Email: orla.whitehead@newcastle.ac.uk orcid ID- 0000-0002-4171-8583Declarations

Competing Interests: None declared.

Registration of review: Prospero- CRD42020200145

https://www.crd.york.ac.uk/prospero/display_record.php?RecordID=200145

Funding statement: OW is funded by the National Institute for Health Research (NIHR) on an in practice fellowship, grant number NIHR301074. The views expressed are those of the authors and not necessarily those of the NIHR or the Department of Health and Social Care. BH and SM are funded by the NIHR Applied Research Collaboration (ARC) North East and North Cumbria (grant number not applicable)

Ethics approval: Not required

Contributorship: Conception and design of study: IOW, SM, and BH. Search design and searching: IOW Screening records: IOW, SM, SW, GS, ET, TPK, BH. Data curation: IOW, BH. Analysis and interpretation of data: IOW, SW, GS. Visualisation and drafting the manuscript: OW, SM, GS, SW, CJ, BH Revising the manuscript critically for important intellectual content and authorizing for publication: OW, SM, CJ, BH, GS, PK, ET

Acknowledgements: Thank you to Catherine Richmond and Hannah O'Keefe for their information science input. Thank you to Carol Jagger for her input.

For peer review only

Data availability: Template collection forms, data extracted from included studies and used for all analyses are available on contacting the authors.

Abstract

Objective: To investigate the relationship between burnout and spiritual health amongst medical doctors

Design: Systematic literature review and narrative synthesis of cross -sectional studies

Setting: Any setting, worldwide

Data sources: Five databases were searched from inception to March 2022, including Medline, Embase, Psychinfo, Scopus and Web of Science

Eligibility Criteria: Any study design that involved Medical doctors (and other healthcare staff if assessed alongside medical doctors), that in any way measured both.

Burnout (or similar) and spiritual health (or similar) medical doctorsData Extraction and Synthesis: Searches yielded 1049 studies. 40 studies met eligibility criteria and were included in this review. Quality was assessed using the Appraisal of Cross-sectional Studies tool (AXIS). Due to heterogeneity of studies, a narrative review was undertaken.

Results: Low reported levels of spirituality were associated with high burnout scores, and vice versa. Religion was not significantly associated with lower levels of burnout. Few studies reported statistically significant findings, few used validated spiritual scores, and most were vulnerable to sampling bias.

Conclusions: Published research suggests that burnout is linked to spiritual health in medical doctors but not to religion. Robust research is needed to confirm these findings and develop effective interventions.

For peer review only

Prospero registration CRD42020200145.

Strengths and limitations of this study

- A comprehensive, systematic approach was taken to searches, with expert information scientist input.
- Broad search terms were used to elicit all the available evidence, as there is inconsistency in headings used in different databases, cultures, and countries.
- A validated tool was used for quality assessment- the Appraisal of Cross-sectional Studies tool.
- The heterogeneity of measures used for burnout and spiritual health meant that meta-analysis was not possible.

Keywords

Burnout, spiritual health, wellbeing, spirituality, religion, compassion fatigue, moral injury

For peer review only

Definitions:

Spiritual Health- The authors use a definition of spiritual health developed by UK General Practitioners: self-actualisation and meaning, transcendence and relationships beyond the self, and expressions of spirituality. This term is used to reflect the broad and diverse experiences of medical doctors.

Burnout- The authors use a definition in use by the World Health Organisation of an occupational phenomenon resulting in exhaustion; distance, negativity or cynicism towards the job and reduced professional efficacy.

Introduction

Burnout is defined by the World Health Organisation as an occupational phenomenon, resulting in exhaustion, mental distance or feelings of negativity or cynicism towards the job, and reduced professional efficacy.(1) This has long been a concern for medical doctors, and the COVID-19 pandemic has increased attention on the health and wellbeing of health professionals.(2, 3) Medical doctors' descriptions of burnout

include a loss of meaning in work, and objectification of patients and their families, rather than engaging with their humanity.[4] As health services globally are placed under increasing pressure, there is growing concern around physician burnout, moral injury and related concepts causing harm to the workforce.(5, 6) Burnout leads to retirement and resignation(7) which adds to the workforce crisis, as well as patient safety concerns.(8) The Covid-19 pandemic has presented huge challenges to the health care workforce.[9, 10] Burnout appears to have a malign effect on all aspects of health,(11, 12) as well as being impacted itself by all aspects of health.(13) The aeitology of burnout is still not fully understood, is likely multi-factorial, and merits further research.(14-16)

The biomedical model that shapes much modern medical practice, has so far been unable to offer a comprehensive and holistic understanding of the burnout phenomenon.(17-19) This, along with the framing of burnout as an occupational phenomenon rather than a mental illness,(1) suggests that a different lens may be needed to develop effective approaches to burnout . In a recent survey, general practitioners (GPs) defined spiritual health in ways that overlapped with current constructs of burnout.(12) Spiritual health was framed by medical doctors as a positive concept.(12) Spiritual health encompasses meaning, purpose, self-actualisation, transcendence and relationships with others beyond the self.(12) Spiritual or religious practices, including mindfulness and yoga, were also included in spiritual health definitions, and both are promoted as approaches to reduce burnout in the workplace.(20, 21) Burnout has been associated with moral injury,(22) referring to the harm caused when someone is required to act contrary to their internal ethical code.(23) Being true to a personal or religious ethical code, was also an aspect of spiritual health reported by GPs.(12) Some authors argue that religiosity and spirituality are one and the same(24), whereas English GPs defined spiritual health as including, but not exclusive to, religion,(12) and there is a growth in the population that consider themselves 'spiritual but not religious.'(25) The concepts of spiritual health and burnout have been found to overlap in other groups of professionals.(26-28) This raises the possibility that promoting spiritual health may offer an effective approach to prevention and mitigation of burnout for medical doctors, with opportunities for intervention at an individual and organizational level.

For peer review only

This systematic review asks whether there is a measured association between burnout and spiritual health amongst medical doctors. It will consider the quantity and quality of quantitative evidence on burnout, spiritual health, and related concepts including spiritual wellbeing and distress, and religiosity by comparing all studies that have performed a measure of burnout and a measure of spiritual health, both in the broadest terms, in qualified medical doctors worldwide.

Methods

Searches and selection of studies

Studies to be included in the review were identified by searching the following databases: (1) EMBASE (1974–March 2022), (2) PsycINFO (1806– March 2022), (3) Ovid MEDLINE (1946–March 2022), (4) Web of Science and (5) Scopus. The last search was performed on the 8th March 2022. The search strategy in the supplementary materials was developed in Embase and adapted for use in other databases by an information scientist. For comprehensiveness both MESH headings and key terms were used. All searches were completely re-run in March 2022. A Cochrane filter(29) and term 'not editorial or letter' was added to filter out non-studies and opinion pieces. Citations from the studies found were searched for previous similar studies. The searches were peer reviewed by an information scientist at Newcastle University.

Eligible studies were those reporting quantified evidence about both burnout (or similar concepts including occupational stress, moral injury from work, occupational compassion fatigue etc.) and spiritual health (or concepts that included this aspect of health). These data must be reported in a study population that included medical doctors from any speciality or in post-graduate training. Any study design was eligible, but commentaries and editorials were excluded. The WHO definition for burnout[1] and the Whitehead et al[2] definitions were referred to and considered by two reviewers at screening stage.There were no language restrictions to the searches.

Population: Medical doctors (post-graduate registered medical professionals from any speciality)

Exposure: Spiritual health, spiritual wellness, spiritual wellbeing, spirituality, religiosity or similar concepts or sub-phenomena, as defined by doctors.(12)

Outcome: Burnout, moral injury, job satisfaction, compassion fatigue, work stress, or similar concepts that could come within the WHO definition for burnout.(1)

For peer review only

Study Design: Any study with quantification of the interventions and outcomes of interest.

2.

Search terms were deliberately comprehensive, and related or similar terms to burnout or spiritual health were included in the searches, for example compassion fatigue, moral injury, mindfulness etc. Studies were included if we felt that the study measured any phenomenon very close to occupational burnout, **and** any measure of any aspect of wellbeing close to spiritual health.

Data extraction and synthesis

All records were double screened at title and abstract, with disagreements resolved by discussion with a third reviewer, or taken to full text screening. De-duplication was done in Endnote X9.(30) Rayyan software was used to manage records.(31) Attempts were made to retrieve all articles, with requests to authors. Emails to authors suggested anecdotally that there could be publication and availability bias, and therefore unpublished studies, found via conference abstracts, were included, and authors were contacted for more information where limited data were presented. No other grey literature was included. Data extraction was performed by one reviewer and a proportion (10%) checked by a second reviewer. Study quality was assessed using the Appraisal of Cross-sectional Studies tool (AXIS).(32) While this tool does not give a 'numerical score', it assessed the methodology and internal consistency of the studies, as well as risk of bias, and as such allows assessment of the merits of each criterion as applied to that study. Heterogeneity in study design, scales, and methods of analysis meant that meta-analysis was not possible, and a narrative synthesis was conducted. Subgroups were considered to see if this gave a greater sense of direction of the evidence. If statistics, for example odds ratios, or relative risks, were provided, these have been summarised within the narrative analysis. Custom made tables were used to analyse the assessments used for burnout and spiritual health, and synthesised the data, including common biases.

The review was registered on Prospero, available from:

https://www.crd.york.ac.uk/prospero/display_record.php?ID=CRD42020200145

Patient and public Involvement

For peer review only

This systematic review was prompted by patient and public involvement in a previous study looking at spiritual health in primary care. The public felt that the health of medical doctors, and burnout, was a priority for this topic.

Results

Forty studies met the inclusion criteria, from screening of the 1049 records identified in the electronic searches (Fig 1). All included studies provided an assessment of burnout and spiritual health (or related concepts) but there was a high level of heterogeneity in the measures used.

FIG 1 HERE

The number of study participants ranged from 4501 in the largest study(33) to 7 in the smallest.(34) Twenty studies included other healthcare staff in addition to the medical staff participants.(34-53) Four studies included primary care physicians,(42, 54-56) with most studies involving medical doctors from intensive or critical care, emergency departments and internal medicine.(33-36, 39, 47, 48, 50, 51, 54, 56-66)(Figure 2) No studies were identified including medical doctors in psychiatry or public health. Data from medical doctors were not always analysed separately.(35, 36, 38, 39, 44, 49, 51, 52)

The oldest study was also the largest, published in 1999 (33). Three studies were conducted during the COVID-19 pandemic (51, 52, 59). A USA study showing lower burnout scores in those who had a faith (including 'spiritual but not religious') (51), a Singapore study with participants who preferred religious coping to thanks from the public (52) and a Portuguese study which showed no association between burnout scores and religiosity in medical doctors (59). Five of the forty studies reported findings from surveys in 2020 onwards.(51, 52, 57, 59, 67)

FIGURE 2 HERE

For peer review only

FIGURE 3 HERE

Almost half of the studies (19) were from the USA (33, 37, 38, 44, 47, 51, 53, 54, 60-62, 64-66, 68-72) reflecting the dominance of North American researchers in this area (Figure 3). Twelve studies included participants from Europe.(36, 39, 43, 45, 49, 50, 55, 57-59, 63, 73) The largest Asian study recruited widely, pooling data from diverse countries in wealth, religion and culture, such as Saudi Arabia, Laos and Bangladesh.(48)

Study Quality

The majority of studies were assessed to be poor quality, with risks of selection bias, response bias, availability bias, and bias. One study, Roslan et al,(56) met all the AXIS criteria fully, including justification of sample size, and there were seven studies which were of otherwise high quality as assessed by the AXIS tool (meeting 18-19 of the 20 criteria).(40-42, 44, 46, 62, 69) Three studies were not yet published in full, and therefore not had peer review.(38, 53, 55) Authors reported difficulty publishing their findings.(38, 74) This, as well as other studies where data was gathered on burnout and spiritual health, but not all comparison data was published,(35, 45, 52, 55, 56, 68) may have introduced availability bias. These were included as it was suspected this topic may be affected by publication bias. Most studies did not use a validated measure of spiritual health, with many studies using non-validated assessments of burnout.

Relationship between burnout and spiritual health in medical doctors

These heterogenous studies drew a wide range of conclusions (Table 1). Seventeen studies found an association between spiritual health or related concepts and lower levels of burnout or related concepts,(37, 39, 40, 42-45, 48, 51, 53, 55, 56, 58, 66, 71, 75, 76) seventeen studies found no statistically significant association,(33-35, 38, 41, 47, 49, 50, 54, 59-61, 63-65, 73, 77) and one study found higher compassion fatigue in those using spiritual type behaviours.(62) Across the eight studies rated high in quality, evidence remained inconsistent. There was evidence of: an inverse association between religiosity and burnout; (42, 44) no association between spiritual concepts and burnout scores;(63, 69) an association between prayer and meditation and higher compassion

For peer review only

fatigue; (62) an association between irregular spirituality routines and burnout; (56) a link between no spirituality and low personal accomplishment;(40) and an association between spirituality and high emotional exhaustion on univariate analysis but not on multivariate analysis.(46) One study found an association between personal accomplishment and burnout, on exclusion of respondents who omitted the spirituality question.(41) Where statistically significant associations between spiritual health (or similar), or religiosity, and burnout (or similar) were found, the effects were typically small.

The removal of studies rated low in quality (score 12 or under on AXIS criteria) does not enhance consistency the findings. Further, there does not appear to be a pattern in terms of whether an association is shown based on country, whether that country has a state religion, but this assessment is limited by the predominance of the USA in these studies. The three most recent studies from the COVID pandemic(51, 52, 59) do not clarify the direction of evidence, with one study finding an association,(51) one did in nurses but not medical doctors,(59) and one where participants agreed they used religious coping, but did not compare religious coping and burnout scores.(52)

Studies which looked at wider spiritual health only, rather than asking about religion, were more likely to find an association between burnout and spiritual health. Seven studies of those which asked or measure spirituality and not religion found an association,(37, 39, 40, 43, 53, 58, 71) and three studies did not.(36, 61, 65) Religion, or religious coping, alone does not appear to have a consistent effect on burnout rates, however, wider spiritual health may have some effect.

Please see Supplementary Table 1: Findings from each study (supplementary materials)

Please see SupplementaryTable 2: Study details, AXIS quality assessment, spiritual wellness assessment and burnout score used (supplementary materials)

Quantification of burnout

For peer review only

Twenty one studies(34, 35, 39-41, 47, 48, 50, 51, 53, 54, 57, 58, 61, 63, 66, 68, 69, 71, 73, 76) used the complete Maslach Burnout Inventory (MBI) (78) (Table 1). One study used a 2-item 'MBI' (64) in an emergency department population, that has been found to correlate with the full MBI in a similar population.(79) Two studies used the Oldenburg Inventory,(45, 59) a measure similar to MBI, but more applicable to people not directly involved in patient care. It also measures engagement with work, not including professional accomplishment. (80) Three more recent studies (52, 56, 65) used the Copenhagen Burnout Inventory (CBI),(81) a free to use (MBI is commercially available) measure of personal, work and patient related burnout, which identifies a similar burned out group to the MBI. (82) Other tools used included the perceived stress scale (PSS), (42, 75) Cool Down Index (36) and Work Related Strain Inventory (WRSI). (70) Whilst these do not measure burnout directly, they have been found to correlate with burnout as measured by the MBI.(36, 70, 83, 84) The Professional quality of life (proQOLl) scale used by two studies (55, 60) measures compassion fatigue, which is considered to be a phenomenon including burnout by the ProQOL authors. (85) One study (62) used the Compassion Fatigue Test for Helpers. Clark et al (37) used an 8 item job satisfaction scale, which included some domains that could be considered as spiritual, for example the sense of calling, meaning and purpose in life and being of worth. (37) Frank et al(33) also measured job satisfaction and desire to become a physician again. Shetach et al(49) also used a job satisfaction questionnaire, translated into Hebrew, designed for an unpublished thesis in marketing, and not validated with healthcare staff.

Assessing spiritual health, wellbeing, spirituality and/or religiosity

An array of methods were used to assess spiritual health or similar concepts (table 2). Validated tools include the Functional Assessment of Chronic Illness Therapy- Spiritual Well-Being Scale , a modified version for non-illness (FACIT-Sp-Non-Illness);(86) the Jarel spiritual wellbeing scale,(87) and Hatch spiritual involvement and belief scale.(88) Büssing et al(36) used the Aspects of Spirituality questionnaire, developed by the authors in a niche setting of a particular philosophical institution.(89) The Fetzer Institute Multidimensional Measurement of Religiousness/Spirituality for Use in Health Research (90) was also used to assess religious practice, along with general, unvalidated, questions about religious commitment, and spiritualty.(64) A systematic review identified the FACIT and the Spirituality Index of Wellbeing (91) as being the highest most well validated instruments,(92)

For peer review only

however only one unpublished study (53) used the former, and none used the latter. Many studies used general coping scores, such as Brief COPE (93) and the Hobfoll's Questionnaire,(94) general quality of life scores or including spiritual practice in general questions, which have aspects of spiritual health as part of a wider assessment. Some studies used single questions, e.g. "my job gives me meaning",(85) "my religious and spiritual beliefs strongly influence my work and patients",(41) "Spirituality/religion is important in my life"(66) or "do you consider yourself spiritual".(40, 71) Seventeen studies which asked about religion only (table 2). The Duke University religion index (DUREL) (95) measures organisational religiosity, measuring religious practice, and was used alongside Hoge's Intrinsic Religiosity Scale by Ramondetta et al.(70) The difference between spirituality and religiosity is not always delineated by the authors: Frank et al used religiosity data asking how 'strongly' participants were religious, and then argue that physicians could be encouraged to explore spirituality to increase satisfaction,(33) however spirituality is not addressed in the data, and Macuka et al discussed religiosity, but their question appeared to ask more generally about spirituality.(45) Ramondetta et al asked participants whether they were religious, spiritual, both or neither, but only quantified religiosity.(70)

Discussion

Key findings

There is increasing attention on both burnout and on the relationship between medical doctors' burnout and their spiritual health. This has become even more critical in the aftermath of the COVID-19 pandemic. Most of the research in this review reported positive associations between higher levels of spiritual health or related concepts, and lower levels of burnout or related phenomena. A number of studies found no association, with two describing greater distress with higher levels of religiosity,(57, 62) perhaps due to use of religious coping as a negative strategy.(96) Similar to other reviews in this area, research from the USA is dominant, with few studies from more secular areas. (The USA has high levels of religiosity(97, 98) than, for example, many northern European countries.(99)) The one high quality study on the topic stated that spirituality routines lower the risk of burnout,(56) as they found that regular spiritual routines were associated with lower burnout risk, however, we cannot infer causation from the study design. Few studies reported statistically significant findings and the marked study heterogeneity did not support metanalysis. While this review suggests that there is probably a link

For peer review only

between spiritual health and burnout in medical doctors, the quality of the evidence is poor, and high quality robust quantitative studies of the relationship between spiritual health and burnout in medical doctors is needed.

Comparison with other work

To our knowledge, this is the most comprehensive review of burnout and spiritual health in medical doctors to date. This review offers a different perspective from other work in this area that utilise a biomedical approach to burnout rather than a holistic approach to include spiritual health.(100-102) Williams et al's systematic review of the consequences of physician burnout considered the physical and mental health implications of burnout in their review, but neglected to mention spiritual health or distress.(101) Chow et al's scoping review of religion and spirituality in residents included discussion of the link between reduced burnout and spirituality. (103) Sibeoni et al's systematic review and meta synthesis of physician's views of burnout includes aspects of spiritual health in 'calmness or letting go', but did not analyse spiritual health as an aspect of overall health. (104) These authors reported that individual factors (including spiritual) and relational factors were more important than organizational concerns in protecting medical doctors from burnout.(104) Current emphasis is on the role of COVID-19 in medical doctors' burnout.(105) with Jefferson et al finding studies considered gender and age effects on burnout during COVID (10) but spiritual health is not mentioned. While this review is limited by the quality of the studies included, there does appear to be merit in the hypothesis of looking wider than the biomedical model.

Common study limitations

There were several limitations common to the studies in this review: response bias, social desirability bias, sampling bias, survivorship/selection bias, and cultural or language bias. There also appears to be availability and publication bias, with the topic of spiritual health being controversial- with data on spiritual health not reported in the published paper, despite being collected. (55, 57) The majority of studies were vulnerable to non-response and social desirability bias. Response rates were highly variable, range 5-99%. Use of gatekeepers, such as heads of departments(85) or directors(48, 50, 71) could cause sampling bias, as could use of convenience sampling at conferences.(57, 60, 66) Non-responders were seldom (eight studies) described. (33, 36, 40, 44, 56, 62, 67, 106) Pressures from the COVID pandemic may have affected studies carried out during this period.(51,

For peer review only

52, 59) Social desirability bias was possible due to staff feeling unable to respond honestly in very religious, or very secular environments, or share the true depth of their burnout symptoms(40). Studies that took place in the work setting were vulnerable to survivorship/selection bias.(38-42, 46, 66) Purvis et al excluded residents, as they were not permanent staff.(47)

Just under half (n=19) of the studies used the Maslach Burnout Inventory (MBI) (78) a measure of burnout often considered the 'gold standard'(107) burnout inventory, Often, studies presented data on the influence of factors such as spiritual health on burnout, when one domain only was affected.(40, 41, 61) The use of other inventories were not justified as to whether this was because these were better tools in their context, because they are free to use, or another reason.

Despite there being multiple available scales of religiosity or spiritual health and wellbeing,(92, 108, 109) few studies used a validated tool to assess personal spiritual health or related concepts. With the growth of the "spiritual, but not religious"(51) and secular societies,(110) there is merit in using validated tool to assess current spiritual health state.(92) The single questions about spiritual wellbeing or similar were untested and unvalidated, and therefore may have introduced bias to the studies, and limit the utility of the data.

The use of measures in English with populations whose first language is not English may affect the quality of results. In some studies, measures were translated into the native language.(34-36, 39, 45, 49, 76, 85) However in other studies, English measures were used, without regard to the language and culture of participants,(40, 48, 57, 75) or without stating which language/translation was used.

Strengths and Limitations of this review

This review is broad, and novel, including a wide definition of aspects of spiritual health. The lack of consistency in indexing for spiritual health and religiosity across electronic database (Medical Subject Headings) meant development of comprehensive searches was a challenge. However, the diverse range and number of papers screened suggests that the searches were sufficiently broad. While most studies are from the USA, the other studies are diverse in country of setting, and cover multiple different cultures and faiths. An objective assessment was made of study quality, using the AXIS tool. Unfortunately, heterogeneity of the measures and

For peer review only

analysis used in the studies has meant meta analysis was not possible. Some published abstracts were unobtainable, and publication bias likely affects this review, although the direction of this effect is unknown. This was mitigated by the inclusion of unpublished data on the topic.(38, 53, 55)

Future research

While there have been studies that have identified this topic as being of interest, these have rarely used validated measures of both variables- burnout and spiritual health. Research utilising accepted and validated tools to measure both spiritual health and burnout are needed, especially in Northern Europe, where research is limited. Future works should also strive to recruit study populations that are not limited to people with specific interest in the topic or strong religious views. Identification, or refutation, of a link between spiritual health and burnout will allow fresh assessment of current organizational and individual interventions for burnout. Developing and evaluating interventions that improve medical doctors' spiritual health may mitigate against burnout.

Conclusions

A link between spiritual health and burnout in medical doctors has been hypothesised by multiple studies, and interest in the link appears to be growing. However, studies investigating this have been vulnerable to many methodological flaws. Despite this, and limited evidence for efficacy,(111) practices rooted in spiritual practice, such as mindfulness, meditation, and yoga, are often recommended to prevent burnout.(47, 112, 113) Since there does not appear to be a relationship between the narrower concept of religiosity and burnout, research into any association between broader spiritual health and burnout, using validated tools, is needed to examine the hypothesis that spiritual health and burnout are associated concepts, and to prompt research into interventions that could prevent burnout in medical doctors.

References

1. World Heath Organisation. Burn-out an "occupational phenomenon": International Classification of Diseases 2019 [Available from: https://www.who.int/mental_health/evidence/burn-out/en/.

For peer review only

2. Locke T. Medscape UK Doctors' Burnout & Lifestyle Survey 2020 2020 [Available from: https://www.medscape.com/slideshow/uk-doctors-burnout-2020-6013312?src=mkm_ret_11012020_mscpmrk_uk_burnout_int&faf=1.
3. Lemaire JB, Wallace JE. Burnout among doctors. BMJ. 2017;358:j3360.
4. Fred HL, Scheid MS. Physician Burnout: Causes, Consequences, and (?) Cures. Tex Heart Inst J. 2018;45(4):198-202.
5. Sundararaman L, Chow SY, Elakkumanan LB, Rajagopalan V, Verma A. Physician Burnout: A Pandemic Uncovered by a Pandemic! ASA Monitor. 2022;86(1):19-20.
6. Melnikow J, Padovani A, Miller M. Frontline physician burnout during the COVID-19 pandemic: national survey findings. BMC Health Services Research. 2022;22(1):365.
7. Sheather J, Slattery D. The great resignation-how do we support and retain staff already stretched to their limit? Bmj. 2021;375:n2533.
8. Garcia CdL, Abreu LCd, Ramos JLS, Castro CFDd, Smiderle FRN, Santos JAd, et al. Influence of Burnout on Patient Safety: Systematic Review and Meta-Analysis. Medicina. 2019;55(9):553.
9. Chor WPD, Ng WM, Cheng L, Situ W, Chong JW, Ng LYA, et al. Burnout amongst emergency healthcare workers during the COVID-19 pandemic: A multi-center study. Am J Emerg Med. 2021;46:700-2.
10. Jefferson L, Golder S, Heathcote C, Avila AC, Dale V, Essex H, et al. GP wellbeing during the COVID-19 pandemic: a systematic review. British Journal of General Practice. 2022;72(718):e325.
11. Salvagioni DAJ, Melanda FN, Mesas AE, González AD, Gabani FL, Andrade SM. Physical, psychological and occupational consequences of job burnout: A systematic review of prospective studies. PLoS One. 2017;12(10):e0185781.
12. Whitehead O, Jagger C, Hanratty B. What do doctors understand by spiritual health? A survey of UK general practitioners. BMJ Open. 2021;11(8):e045110.
13. Diez-Pinol M, Dolan S, Sierra V, Cannings K. Personal and organizational determinants of well-being at work: The case of Swedish physicians. International Journal of Health Care Quality Assurance. 2008.
14. Weber A, Jaekel-Reinhard A. Burnout syndrome: a disease of modern societies? Occupational medicine. 2000;50(7).
15. Boisaubin EV. Causes and treatment of impairment and burnout in physicians: The epidemic within. Faculty health in academic medicine. 2009:29.
16. Kumar S, Hatcher S, Huggard P. Burnout in Psychiatrists: An Etiological Model. International journal of psychiatry in medicine. 2005;35(4):405-16.
17. Engebretsen KM, Bjorbækmo WS. Naked in the eyes of the public: A phenomenological study of the lived experience of suffering from burnout while waiting for recognition to be ill. Journal of Evaluation in Clinical Practice. 2019;25(6):1017-26.
18. Engebretsen KM. Suffering without a medical diagnosis. A critical view on the biomedical attitudes towards persons suffering from burnout and the implications for medical care. Journal of Evaluation in Clinical Practice. 2018;24(5):1150-7.
19. Heinemann LV, Heinemann T. Burnout Research: Emergence and Scientific Investigation of a Contested Diagnosis. SAGE Open. 2017;7(1):2158244017697154.
20. Goodman MJ, Schorling JB. A Mindfulness Course Decreases Burnout and Improves Well-Being among Healthcare Providers. The International Journal of Psychiatry in Medicine. 2012;43(2):119-28.
21. Ireland MJ, Clough B, Gill K, Langan F, O'Connor A, Spencer L. A randomized controlled trial of mindfulness to reduce stress and burnout among intern medical practitioners. Medical Teacher. 2017;39(4):409-14.
22. Dean W, Talbot S, Dean A. Reframing Clinician Distress: Moral Injury Not Burnout. Fed Pract. 2019;36(9):400-2.
23. Talbot SG, Dean W. Physicians aren't 'burning out.'They're suffering from moral injury. Stat. 2018.
24. Koenig HG. Religion, Spirituality, and Medicine: Application to Clinical Practice. JAMA. 2000;284(13):1708-.
25. Lee L. Secular or nonreligious? Investigating and interpreting generic 'not religious' categories and populations. Religion. 2014;44(3):466-82.
26. Hardiman P, Simmonds JG. Spiritual well-being, burnout and trauma in counsellors and psychotherapists. Mental Health, Religion & Culture. 2013;16(10):1044-55.
27. Wachholtz A, Rogoff M. The relationship between spirituality and burnout among medical students. J Contemp Med Educ. 2013;1(2):83-91.
28. Lizano EL, Godoy AJ, Allen N. Spirituality and worker well-being: Examining the relationship between spirituality, job burnout, and work engagement. Journal of Religion & Spirituality in Social Work: Social Thought. 2019;38(2):197-216.
29. Cochrane library. Search strategies to identify observational studies in MEDLINE and Embase [Available from: https://www.cochranelibrary.com/cdsr/doi/10.1002/14651858.MR000041.pub2/appendices.

For peer review only

30. The EndNote Team. Endnote. Endnote X9 ed. Philadelphia, PA2013.
31. Ouzzani M, Hammady H, Fedorowicz Z, Elmagarmid A. Rayyan—a web and mobile app for systematic reviews. Systematic Reviews. 2016;5(1):210.
32. Downes MJ, Brennan ML, Williams HC, Dean RS. Development of a critical appraisal tool to assess the quality of cross-sectional studies (AXIS). BMJ Open. 2016;6(12):e011458.
33. Frank E, McMurray J, Linzer M, Elon L. Career satisfaction of US women physicians - Results from the Women Physicians' Health Study. ARCHIVES OF INTERNAL MEDICINE. 1999;159(13):1417-26.
34. Das S, Barman S, Datta S, Bardhan N, Baishya M, Das B, et al. Degree of burnout among emergency healthcare workers and factors influencing level of burnout: a pilot study. Delhi Psychiatry Journal. 2016;19:36-47.
35. Baruah A, Das S, Dutta A, Das B, Sharma T, Hazarika M. DEGREE AND FACTORS OF BURNOUT AMONG EMERGENCY HEALTHCARE WORKERS IN INDIA. Int J Sci Res (Ahmedabad). 2019;8(4):41-5.
36. Büssing A, Loetzke D, Gloeckler M, Heusser P. Influence of Spirituality on Cool Down Reactions, Work Engagement, and Life Satisfaction in Anthroposophic Health Care Professionals. Evidence-Based Complementary and Alternative Medicine. 2015.
37. Clark L, Leedy S, Mcdonald L, Muller B, Lamb C, Mendez T, et al. Spirituality and job satisfaction among hospice interdisciplinary team members. JOURNAL OF PALLIATIVE MEDICINE. 2007;10(6):1321-8.
38. Guay MD, Reyes Donoso MM, Lopez Saca JM, Riveros Rios ME, Pastrana T. Spirituality and Religiosity and Burnout in Latin-American Palliative Care Health Care Professionals (LAPC) (S818). Journal of Pain and Symptom Management. 2019;57(2):490-1.
39. Glebocka A, Lisowska E, Glebocka A, Lisowska E. Professional burnout and stress among polish physicians explained by the Hobfoll resources theory. JOURNAL OF PHYSIOLOGY AND PHARMACOLOGY. 2007;58:243-52.
40. Koh MYH, Chong PH, Neo PSH, Ong YJ, Yong WC, Ong WY, et al. Burnout, psychological morbidity and use of coping mechanisms among palliative care practitioners: A multi-centre cross-sectional study. Palliative Medicine. 2015;29(7):633-42.
41. Lal A, Tharyan A, Tharyan P. The prevalence, determinants and the role of empathy and religious or spiritual beliefs on job stress, job satisfaction, coping, burnout, and mental health in medical and surgical faculty of a teaching hospital: A cross-sectional survey. Revue de Medecine Interne. 2020;41(4):232-40.
42. Leonelli LB, Andreoni S, Martins P, Kozasa EH, Salvo VLd, Sopezki D, et al. Perceived stress among Primary Health Care Professionals in Brazil. Estresse percebido em profissionais da Estrategia Saude da Familia. 2017;20(2):286-98.
43. Macuka I, Tucak Junakovic I. A Cross-Sectional Study of Job Satisfaction and Intention to Leave Job in Palliative Care in Croatia. Journal of palliative care. 2021:8258597211046704.
44. Mantri S, Lawson JM, Wang ZZ, Koenig HG. Prevalence and Predictors of Moral Injury Symptoms in Health Care Professionals. JOURNAL OF NERVOUS AND MENTAL DISEASE. 2021;209(3):174-80.
45. Macuka I, Junakovic I, Bozic D, Macuka I, Junakovic IT, Bozic D. Burnout among Palliative Care Professionals. DRUSTVENA ISTRAZIVANJA. 2020;29(2):287-308.
46. Ntantana A, Matamis D, Savvidou S, Giannakou M, Gouva M, Nakos G, et al. Burnout and job satisfaction of intensive care personnel and the relationship with personality and religious traits: An observational, multicenter, cross-sectional study. Intensive and Critical Care Nursing. 2017;41:11-7.
47. Purvis T, Saylor D, Powell B, Biba G, Conti D, Crowe T, et al. Burnout and Resilience Among Neurosciences Critical Care Unit Staff. NEUROCRITICAL CARE. 2019;31(2):406-10.
48. See K, Zhao M, Nakataki E, Chittawatanarat K, Fang W, Faruq M, et al. Professional burnout among physicians and nurses in Asian intensive care units: a multinational survey. INTENSIVE CARE MEDICINE. 2018;44(12):2079-90.
49. Shetach A, Marcus O, Shetach A, Marcus O. The critical managerial capabilities of medical and nursing managers in an Israeli hospital. EVIDENCE-BASED HRM-A GLOBAL FORUM FOR EMPIRICAL SCHOLARSHIP. 2015;3(1):81-102.
50. Teixeira C, Ribeiro O, Fonseca AM, Carvalho AS. Burnout in intensive care units - a consideration of the possible prevalence and frequency of new risk factors: A descriptive correlational multicentre study. BMC Anesthesiology. 2013;13:38.
51. Antonsdottir I, Rushton CH, Nelson KE, Heinze KE, Swoboda SM, Hanson GC. Burnout and moral resilience in interdisciplinary healthcare professionals. Journal of Clinical Nursing. 2022;31(1):196-208.
52. Chor WPD, Ng WM, Cheng L, Situ W, Chong JW, Ng LYA, et al. Burnout amongst emergency healthcare workers during the COVID-19 pandemic: A multi-center study. American Journal of Emergency Medicine. 2021;46:700-2.
53. Schmidt C, Roffler M. Who isn't burned out? The role of spirituality in graduate medical education (GME). 2018.

For peer review only

54. Doolittle B, Windish D, Doolittle BR, Windish DM. Correlation of burnout syndrome with specific coping strategies, behaviors, and spiritual attitudes among interns at Yale University, New Haven, USA. JOURNAL OF EDUCATIONAL EVALUATION FOR HEALTH PROFESSIONS. 2015;12.
55. McKinley N, McCain RS, Convie L, Clarke M, Dempster M, Campbell WJ, et al. Resilience, burnout and coping mechanisms in UK doctors: a cross-sectional study. BMJ Open. 2020;10(1):e031765.
56. Roslan NS, Yusoff MSB, Ab Razak A, Morgan K, Shauki NIA, Kukreja A, et al. Training Characteristics, Personal Factors and Coping Strategies Associated with Burnout in Junior Doctors: A Multi-Center Study. HEALTHCARE. 2021;9(9).
57. Azoulay E, De Waele J, Ferrer R, Staudinger T, Borkowska M, Povoa P, et al. Symptoms of burnout in intensive care unit specialists facing the COVID-19 outbreak. Annals of Intensive Care. 2020;10(1):110.
58. Ben-Itzhak S, Dvash J, Maor M, Rosenberg N, Halpern P. Sense of meaning as a predictor of burnout in emergency physicians in Israel: a national survey. Clinical and Experimental Emergency Medicine. 2015;2(4):217-25.
59. Correia I, Almeida AE. Organizational Justice, Professional Identification, Empathy, and Meaningful Work During COVID-19 Pandemic: Are They Burnout Protectors in Physicians and Nurses? FRONTIERS IN PSYCHOLOGY. 2020;11.
60. Doolittle BR. Association of Burnout with Emotional Coping Strategies, Friendship, and Institutional Support Among Internal Medicine Physicians. JOURNAL OF CLINICAL PSYCHOLOGY IN MEDICAL SETTINGS. 2020.
61. Doolittle BR, Windish DM, Seelig CB. Burnout, coping, and spirituality among internal medicine resident physicians. J Grad Med Educ. 2013;5(2):257-61.
62. Gribben J, Kase S, Waldman E, Weintraub A, Gribben JL, Kase SM, et al. A Cross-Sectional Analysis of Compassion Fatigue, Burnout, and Compassion Satisfaction in Pediatric Critical Care Physicians in the United States*. PEDIATRIC CRITICAL CARE MEDICINE. 2019;20(3):213-22.
63. Ntantana A, Matamis D, Savvidou S, Giannakou M, Gouva M, Nakos G, et al. Burnout and job satisfaction of intensive care personnel and the relationship with personality and religious traits: An observational, multicenter, cross-sectional study. Intensive and Critical Care Nursing. 2017;41:11-7.
64. Salmoirago-Blotcher E, Fitchett G, Leung K, Volturo G, Boudreaux E, Crawford S, et al. An exploration of the role of religion/spirituality in the promotion of physicians' wellbeing in Emergency Medicine. Preventive Medicine Reports. 2016;3:189-95.
65. St Onge JE, Allespach H, Diaz Y, Poitier A, Tamariz L, Paidas C, et al. Burnout: exploring the differences between U.S. and international medical graduates. BMC medical education. 2022;22(1):69.
66. Watson A, Saggar V, MacDowell C, McCoy J, Watson AG, Saggar V, et al. Self-reported modifying effects of resilience factors on perceptions of workload, patient outcomes, and burnout in physician-attendees of an international emergency medicine conference. PSYCHOLOGY HEALTH & MEDICINE. 2019;24(10):1220-34.
67. Mantri S, Lawson J, Wang Z, Koenig H, Mantri S, Lawson JM, et al. Identifying Moral Injury in Healthcare Professionals: The Moral Injury Symptom Scale-HP. JOURNAL OF RELIGION & HEALTH. 2021.
68. Guest RS, Baser R, Li Y, Scardino PT, Brown AE, Kissane DW. Cancer surgeons' distress and well-being, II: modifiable factors and the potential for organizational interventions. Annals of surgical oncology. 2011;18(5):1236-42.
69. Harper L, Alshammari D, Ferdynus C, Kalfa N. Burnout amongst members of the French-speaking Society of Pediatric and Adolescent Urology (SFUPA). Are there specific risk factors? Journal of pediatric urology. 2020;16(4):482-6.
70. Ramondetta L, Urbauer D, Brown A, Richardson G, Thaker P, Koenig H, et al. Work related stress among gynecologic oncologists. GYNECOLOGIC ONCOLOGY. 2011;123(2):365-9.
71. Suttle M, Chase M, Sasser W, Moore-Clingenpeel M, Maa T, Werner J, et al. Burnout in Pediatric Critical Care Medicine Fellows*. CRITICAL CARE MEDICINE. 2020;48(6):872-80.
72. Yoon J, Rasinski K, Curlin F, Yoon JD, Rasinski KA, Curlin FA. Conflict and emotional exhaustion in obstetrician-gynaecologists: a national survey. JOURNAL OF MEDICAL ETHICS. 2010;36(12):731-5.
73. Leu S, Vuille-dit-Bille R, Fink L, Soll C, Staerkle R, Leu S, et al. Burnout in Swiss and Australian surgeons in training-a cross-sectional study. EUROPEAN SURGERY-ACTA CHIRURGICA AUSTRIACA.
74. Schmidt C, Roffler M. Coping with the Practice of Medicine: Religion, Spirituality, and Other Personal Strategies. JOURNAL OF RELIGION & HEALTH. 2021;60(3):2092-108.
75. Alosaimi FD, Alawad HS, Alamri AK, Saeed AI, Aljuaydi KA, Alotaibi AS, et al. Stress and coping among consultant physicians working in Saudi Arabia. ANNALS OF SAUDI MEDICINE. 2018;38(3):214-24.
76. Glasberg J, Horiuti L, Novais M, Canavezzi A, Miranda V, Chicoli F, et al. Prevalence of the burnout syndrome among Brazilian medical oncologists. REVISTA DA ASSOCIACAO MEDICA BRASILEIRA. 2007;53(1):85-9.

For peer review only

77. Ramondetta LM, Urbauer D, Brown AJ, Richardson G, Thaker PH, Koenig HG, et al. Work related stress among gynecologic oncologists. GYNECOLOGIC ONCOLOGY. 2011;123(2):365-9.
78. Maslach C, Leiter MP. The Truth About Burnout: How Organizations Cause Personal Stress and What to Do About It: Wiley; 2008.
79. Li-Sauerwine S, Rebillot K, Melamed M, Addo N, Lin M. A 2-Question Summative Score Correlates with the Maslach Burnout Inventory. West J Emerg Med. 2020;21(3):610-7.
80. Demerouti E, Bakker AB. The Oldenburg Burnout Inventory: A good alternative to measure burnout and engagement. Handbook of stress and burnout in health care. 2008:65-78.
81. Kristensen TS, Borritz M, Villadsen E, Christensen KB. Copenhagen burnout inventory. Work & Stress. 1999.
82. Winwood PC, Winefield AH. Comparing Two Measures of Burnout Among Dentists in Australia. International Journal of Stress Management. 2004;11(3):282-9.
83. Swami M, Mathur D, Pushp B. Emotional intelligence, perceived stress and burnout among resident doctors: an assessment of the relationship. The National medical journal of India. 2013;26(4):210-3.
84. Teles R, Valle A, Rodríguez S, Piñeiro I, Regueiro B. Perceived Stress and Indicators of Burnout in Teachers at Portuguese Higher Education Institutions (HEI). Int J Environ Res Public Health. 2020;17(9):3248.
85. Ben-Itzhak S, Dvash J, Maor M, Rosenberg N, Halpern P, Ben-Itzhak S, et al. Sense of meaning as a predictor of burnout in emergency physicians in Israel: a national survey. CLINICAL AND EXPERIMENTAL EMERGENCY MEDICINE. 2015;2(4):217-25.
86. Facit.org. FACIT-Sp Non-Illness [Available from: https://www.facit.org/measures/FACIT-Sp-Non-Illness.
87. Hungelmann J, Kenkel-Rossi E, Klassen L, Stollenwerk R. Focus on Spiritual Well-Being: Harmonious interconnectedness of mind-body-spirit—Use of the JAREL Spiritual Well-Being Scale: Assessment of spiritual well-being is essential to the health of individuals. Geriatric Nursing. 1996;17(6):262-6.
88. Hatch RL, Burg MA, Naberhaus DS, Hellmich LK. The spiritual involvement and beliefs scale. Journal of Family Practice. 1998;46(6):476-86.
89. Büssing A, Hirdes AT, Baumann K, Hvidt NC, Heusser P. Aspects of Spirituality in Medical Doctors and Their Relation to Specific Views of Illness and Dealing with Their Patients' Individual Situation. Evidence-Based Complementary and Alternative Medicine. 2013;2013:734392.
90. Institute F. Multidimensional Measurement of Religiousness/Spirituality for Use in Health Research [Available from: https://fetzer.org/resources/multidimensional-measurement-religiousnessspirituality-use-health-research.
91. Daaleman TP, Frey BB, Wallace D, Studenski SA. The Spirituality Index of Well-Being: Development and testing of a new measure. J Fam Pract. 2002;51(11):952.
92. Monod S, Brennan M, Rochat E, Martin E, Rochat S, Büla CJ. Instruments Measuring Spirituality in Clinical Research: A Systematic Review. Journal of General Internal Medicine. 2011;26(11):1345.
93. Carver CS. You want to measure coping but your protocol's too long: consider the brief COPE. Int J Behav Med. 1997;4(1):92-100.
94. Hobfoll SE. Conservation of Resources: A New Attempt at Conceptualizing Stress. The American psychologist. 1989;44(3):513-24.
95. Koenig H, Parkerson Jr GR, Meador KG. Religion index for psychiatric research. The American Journal of Psychiatry. 1997;154(6):885-6.
96. Gerber MM, Boals A, Schuettler D. The unique contributions of positive and negative religious coping to posttraumatic growth and PTSD. Psychology of religion and spirituality. 2011;3(4):298-307.
97. PRRI. The 2020 Census of American Religion 2021 [07.08.2021]. Available from: https://www.prri.org/research/2020-census-of-american-religion/.
98. Centre PR. With high levels of prayer, U.S. is an outlier among wealthy nations 2019 [Available from: https://www.pewresearch.org/fact-tank/2019/05/01/with-high-levels-of-prayer-u-s-is-an-outlier-among-wealthy-nations/.
99. Centre PR. How do European countries differ in religious commitment? Use our interactive map to find out 2018 [Available from: https://www.pewresearch.org/fact-tank/2018/12/05/how-do-european-countries-differ-in-religious-commitment/.
100. Zgliczyńska M, Zgliczyński S, Ciebiera M, Kosińska-Kaczyńska K. Occupational Burnout Syndrome in Polish Physicians: A Systematic Review. Int J Environ Res Public Health. 2019;16(24).
101. Williams ES, Rathert C, Buttigieg SC. The Personal and Professional Consequences of Physician Burnout: A Systematic Review of the Literature. Medical Care Research and Review. 2019;77(5):371-86.
102. Balendran B, Bath MF, Awopetu AI, Kreckler SM. Burnout within UK surgical specialties: a systematic review. The Annals of The Royal College of Surgeons of England. 2021;103(7):464-70.
103. Chow HHE, Chew QH, Sim K. Spirituality and religion in residents and inter-relationships with clinical practice and residency training: A scoping review. BMJ Open. 2021;11(5):e044321.

For peer review only

104. Sibeoni J, Bellon-Champel L, Mousty A, Manolios E, Verneuil L, Revah-Levy A. Physicians' Perspectives About Burnout: a Systematic Review and Metasynthesis. Journal of General Internal Medicine. 2019;34(8):1578-90.

105. Kang MJ, Park JH. The effects of COVID-19 on physician's burnout: a systematic review. J Korean Med Assoc. 2021;64(9):636-46.

106. Lal A, Tharyan A, Tharyan P. The prevalence, determinants and the role of empathy and religious or spiritual beliefs on job stress, job satisfaction, coping, burnout, and mental health in medical and surgical faculty of a teaching hospital: A cross-sectional survey. La Revue de Médecine Interne. 2020.

107. Williamson K, Lank PM, Cheema N, Hartman N, Lovell EO. Comparing the Maslach Burnout Inventory to Other Well-Being Instruments in Emergency Medicine Residents. J Grad Med Educ. 2018;10(5):532-6.

108. de Jager Meezenbroek E, Garssen B, van den Berg M, van Dierendonck D, Visser A, Schaufeli WB. Measuring Spirituality as a Universal Human Experience: A Review of Spirituality Questionnaires. Journal of Religion and Health. 2012;51(2):336-54.

109. Lu J, Lo C-L, Bang NM, Romero D. A Critical Review of Spiritual Well-Being Scales: Implications for Research, Practice, and Education in Rehabilitation Counseling. Rehabilitation Counseling Bulletin. 2018;62(3):144-56.

110. Assing Hvidt E, Søndergaard J, Hansen DG, Gulbrandsen P, Ammentorp J, Timmermann C, et al. 'We are the barriers': Danish general practitioners' interpretations of why the existential and spiritual dimensions are neglected in patient care. Commun Med. 2017;14(2):108-20.

111. Scheepers RA, Emke H, Epstein RM, Lombarts KMJMH. The impact of mindfulness-based interventions on doctors' well-being and performance: A systematic review. Medical Education. 2020;54(2):138-49.

112. Enhancement CL. Preventing Burnout [Available from: https://inside.ewu.edu/calelearning/psychological-skills/preventing-burnout/.

113. HelpGuide.org. Burnout Prevention and Treatment [Available from: https://www.helpguide.org/articles/stress/burnout-prevention-and-recovery.htm.

Supplementary Materials-

Supplementary Table 1, Supplementary Table 2

Prisma S, Prisma Abstract and Prisma 2020 checklists

Full protocol (supplementary file 1)

Full search strategy (supplementary file 2)

List of figures

Figure 1: Prisma Diagram

Figure 2: Medical specialty of participants

Figure 3: Geographical location of participants of studies included in this review

For peer review only

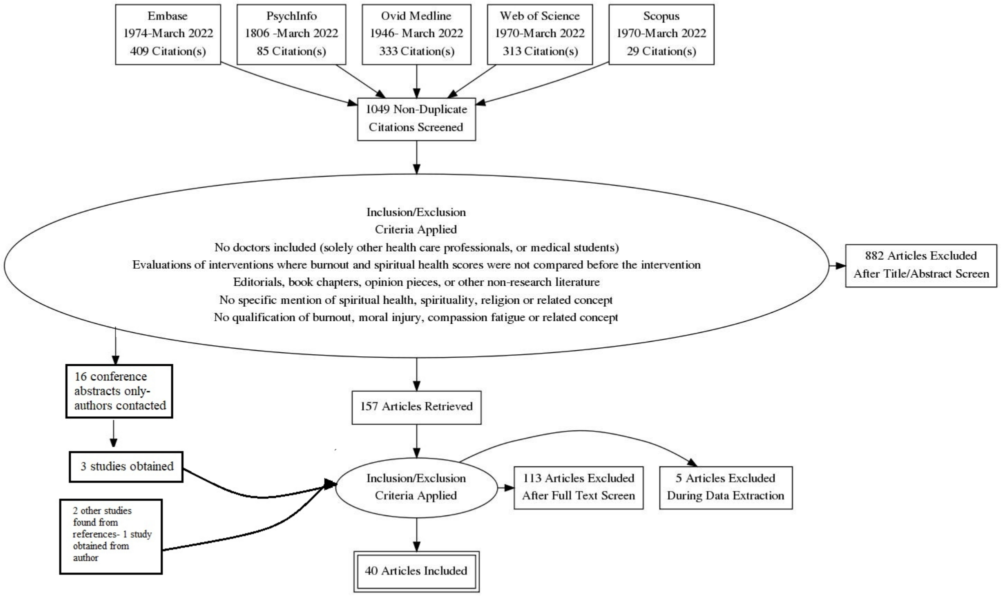

Figure 1: Prisma Diagram

383x227mm (96 x 96 DPI)

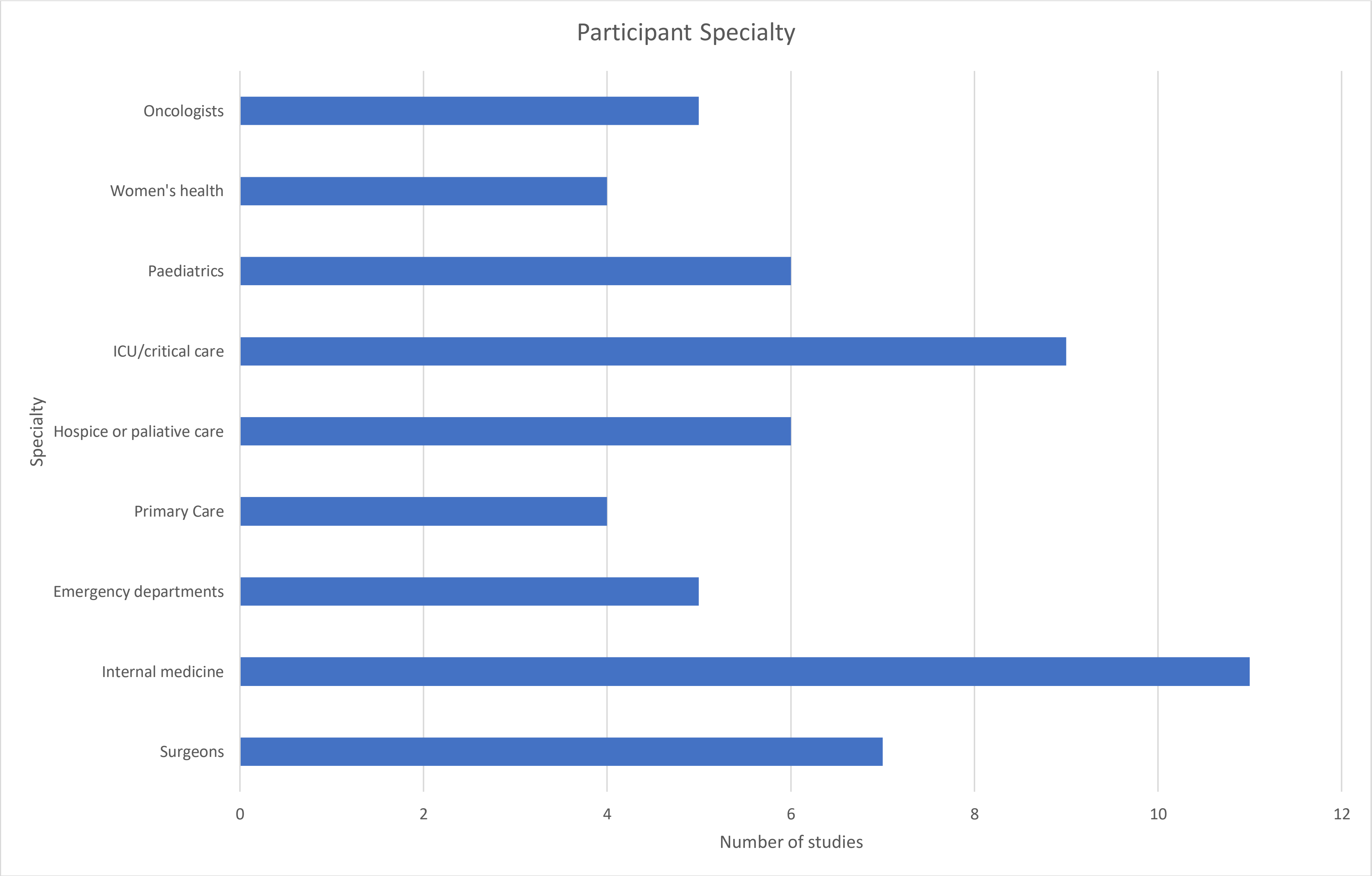
Participant Specialty
Oncologists
Women's health
Paediatrics
ICU/critical care
Hospice or paliative care
Primary Care
Emergency departments
Internal medicine
Surgeons
Specialty
0
2
4
6
8
10
12
Number of studies

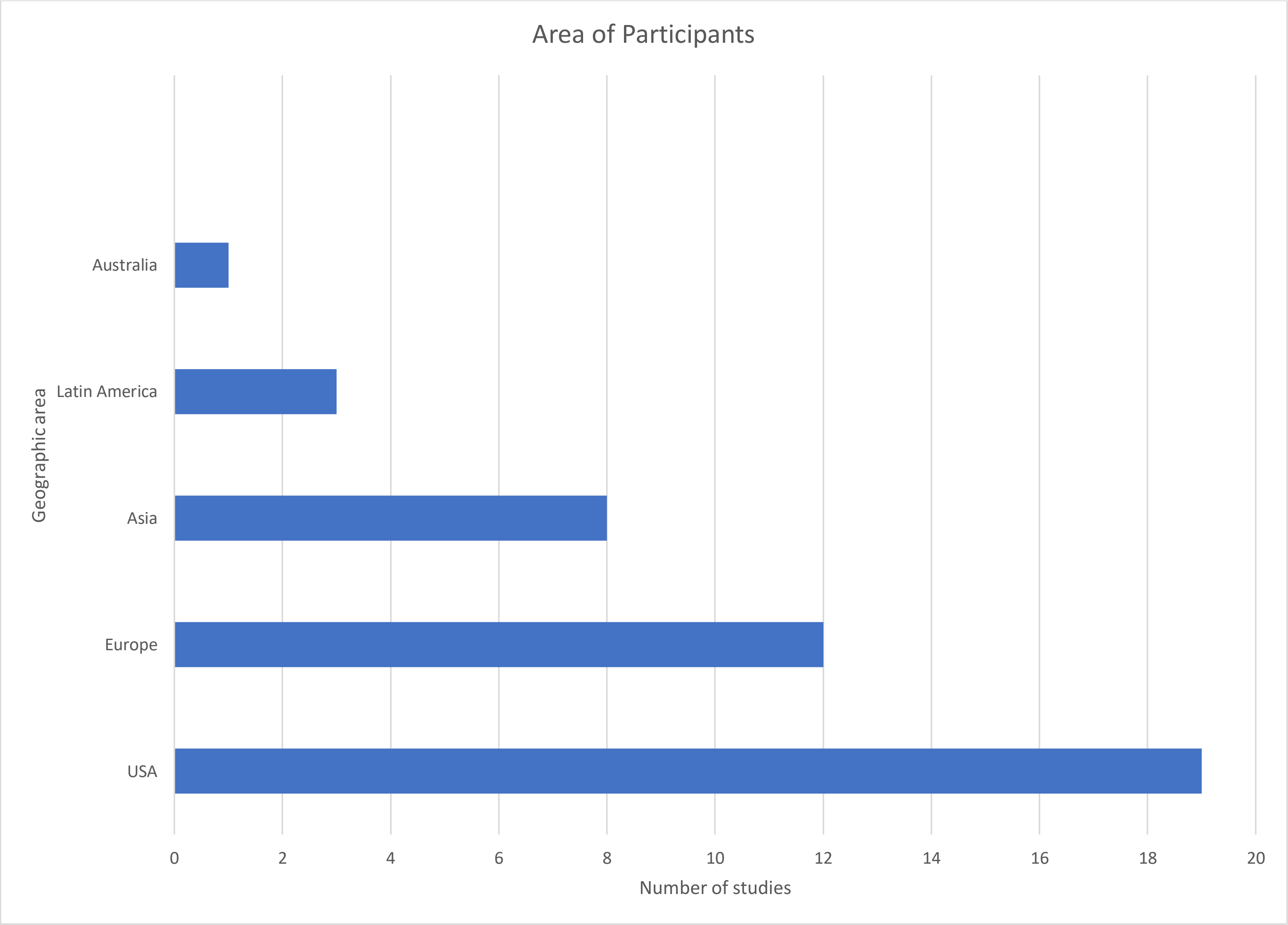
Area of Participants
Geographic area
Australia
Latin America
Asia
Europe
USA
0
2
4
6
8
10
12
14
16
18
20
Number of studies

Table 1: Findings from each study

Study	Comparisons of Burnout and Spiritual Health	Estimate or test statistic (95% confidence intervals in brackets, if given)	P value	The association of burnout (or similar concepts) with spiritual health (or similar concepts)
Alosaimi et al(1) 2018 Saudi Arabia	Perceived stress score was compared with use of religious coping (Brief-COPE)	Spearman's rho −0.09	0.030	Lower stress scores in those with higher religious coping
	Perceived stress score was compared for those who use religious coping (open ended question)	Number of participants:<br>Low PSS-66<br>Medium PSS- 48<br>High PSS- 33	0.004	
Antonsdottir et al(2) 2022 USA	Religion was compared with each domain of the MBI	DP β / EE β / PA β<br>Christian −2.73*** / −4.97** / 1.76*<br>Jewish −0.55 / −1.82 / 0.03<br>Spiritual, not religious −2.35** / −2.64 / 1.86<br>Other −2.58*** / −3.59 / 1.53	*** p<0.001<br>**p<0.01<br>*p<0.05	Lower burnout scores in those with a religious/spirituality.
Azoulay(3) 2020 Europe	Anxiety and depression scores were compared with religiosity			Burnout score and religiosity not commented on.
Baruah(4) 2019 India	MBI compared with type of religion (no non-religious)	EE: χ2= 1.881	DP p=0.7579<br>EE p=0.3905<br>PA p=1.00	No association found
Ben-Itzak(5) 2015 Israel	The extent to which work provides meaning was associated with burnout scores	β=-3.144 (0.045- 6.24)	P<0.05	Higher work related meaning was associated with lower burnout scores.

Büssing et al(6) 2014 Germany	The aspects of spirituality domains were compared with the cool down index and perceived work burden	Aspects of spirituality domains / Cool down index / Perceived work burden<br>*Religious orientation* ρ=−0.078 ρ=−0.044<br>*Search for insight/wisdom* ρ=−0.107 ρ=−0.036<br>*Conscious interactions* ρ=−0.14* ρ=−0.165*<br>*Transcendence conviction* ρ=−0.106 ρ=−0.044	*P<0.01	No relationship- no statistical association between spiritual score and cool down reactions or perceived work burden
Chor et al(7) 2021 Singapore	55.8% used religious coping more than acts of gratitude from the public.			Burnout and religious coping use not compared.
Clark(8) 2007 USA	The statistical model chosen assumes a relationship between spirituality and job satisfaction via integration and self-actualisation	χ^2 0.614	p- 0.433	Higher job satisfaction was associated with higher spirituality
Correia et al(9) 2020 Portugal	Religiosity was compared with burnout score domains	Exhaustion- β= 0.07<br>Disengagement- β= 0.02		No association
Das(10) 2016 India	Religion was compared with burnout domains	EE- χ^2= 1.556<br>DP- χ^2= 2.917<br>PA- χ^2= 1.556	EE- p= 0.212<br>DP- p= 0.233<br>PA- p= 0.459	No association

Doolittle et al(11)2013 USA	Correlation between SIBS score and MBI	EE- r=-0.11<br>DP- r=-0.13<br>PA- r=0.28	EE- p=0.25<br>DP- p=0.17<br>PA- p=0.003	No statistical association
Doolittle(12) 2020 USA	Three domains from the Durel are reported as Pearson's correlation coefficients	EE / DP / PA<br>Organized religion: − 0.12 / − 0.21 / + 0.14<br>Private practices: − 0.21 / − 0.12 / + 0.23<br>Religiosity: − 0.18 / − 0.18 / + 0.20		No association
Doolittle and Windish(13) 2015 USA	Spearman's rank correlation coefficient	EE- 'not significant'<br>DP- 'not significant'<br>PA- 0.35	PA- p=0.03	No association
Frank(14) 1999 USA	Religious fervour was compared with desire to become a physician again	Odds ratio- 1.3 (1.1-1.6)		No direct association found
Glasberg et al(15) 2007 Brazil	Religious identity was compared with MBI scores		p=0.038	Higher burnout in the non-religious
Glebocka et al(16) 2007 Poland	Hobfall's gains and losses were compared with MBI scores (translated), correlation coefficients reported	EE / DP / Professional Cynicism<br>Spiritual resources gain: 0.14 / 0.13 / 0.29<br>Spiritual resources loss: 0.09 / 0.35* / -0.06	*p<0.05	Positive correlation between burnout and spiritual resource loss.
Gribben et al(17) 2019 USA	Compares prayer/meditation and compassion fatigue	β=0.12	p<0.001	Higher compassion fatigue in those using spiritual type behaviours

Guay et al(18) 2019 Americas	Number of participants with burnout compared by religion.	χ^2 values not given	Religious affiliation p= 0.184 Importance of spirituality p=0.984 Importance of religion p=0.084	No association
Guest et al(19) 2011 USA	Spirituality mentioned as protective factor against burnout	Numeric data not given		MBI and spirituality not compared
Harper et al(20) 2020 USA	Believer/non-believers MBI scores compared	χ^2 values not given	p=0.69	No association
Koh et al(21) 2015 Singapore	The odds of being burned out and answering 'no' to being spiritual were compared.	odds ratios:<br>High DP- 2.83 (1.2-6.3)<br>Low PA- 2.85 (1.4-6.0)<br>High EE- 1.67 (0.76-3.64)<br>Overall burnout- 1.80 (0.86-3.71)		Those who answered 'no' to the spiritual question had lower personal accomplishment
Lal et al(22) 2020 India	MBI domains were compared with whether religious or spiritual beliefs influence work	ANOVA values not given	PA p=0.002	No overall association
Leonelli et al(23) 2017 Brazil	Perceived stress was compared with religious activity	β= 3.1 [0.3- 5.8]	p=0.03	Lower religious activity associated with higher perceived stress
Leu et al(24) 2020 Switzerland and Australia	Atheism vs any religion was compared with burnout	β= -52.5	p=0.13	No association

Macuka et al(25) 2020 Croatia	Compares spiritual question and burnout scores	EE- β= -0.20<br>r=-0.19<br>Disengagement- β=-0.18<br>r=-0.19	$p<0.05$<br>$p<0.05$<br>$p<0.05$<br>$p<0.05$	Religiosity was associated with lower emotional exhaustion.
Macuka et al 2021(26) Croatia	Spiritual question and job satisfaction were compared	r=0.15	$p>0.05$	Higher job satisfaction in those with higher religiosity
Mantri et al 2021(27) USA	Compares religiosity and moral injury scores	data not given		Moral injury symptoms and burnout inversely related to religiosity.
McKinley et al 2020(28) full data from Dr McKinley's thesis(29)	Religious coping was compared with burnout score	Unadjusted β= -0.42	$p<0.01$	Higher religious coping associated with lower burnout
Ntantana et al(30) 2017 Greece	Spirituality and burnout were compared, only EE reported	OR for high burnout given high EE- 1.482 (0.823-2.670)		No association
Purviss et al(31) 2019 USA	Religion (Catholics vs other) was compared with burnout score.		p = 0.026	No association
Ramondetta et al(32) 2011 Greece	Correlation between religiosity and Work Related Strain Inventory (WRSI)	Duke religiosity index and WRSI r=0.08<br>Intrinsic religiosity scale and WRSI r=0.05		No association
Roslan et al(33) 2021 Malaysia	Irregular spirituality routines were compared with burnout scores	OR- 2.24 (1.49–3.37))	$p<0.001$	Work related burnout was associated with irregular spirituality routines

Salmoirago-Blotcher(34)<br>2016<br>USA	Various religious/spiritual questions were compared with burnout scores	**Organized religiosity** — **Adjusted OR**<br>Never — Ref.<br>Any — 0.89 (0.36 to 2.20)<br>**Religious Affiliation**<br>None — Ref.<br>Any — 0.51 (0.19 to 1.39)<br>***Private prayer***<br>Never — Ref.<br>Any — 0.66 (0.27 to 1.60)<br>***Meditation***<br>Never — Ref.<br>Any — 0.56 (0.19 to 1.62)<br>**Self-rated spirituality**<br>Slightly/not spiritual — Ref.<br>Moderately spiritual — 1.14 (0.45 to 2.88)<br>Very spiritual — 0.15 (0.02 to 1.46)<br>**Religious rest**<br>Never — Ref.<br>Any — 0.51 (0.16 to 1.65)<br>***"I try hard to carry my religious beliefs over into all my life"***		No association

		Disagree Ref.<br>Agree 0.70 (0.28 to 1.74)		
Schmidt and Roffler(35) 2021<br><br>Quantitative data given by author, unpublished(36)	Correlation between spiritual score and burnout score	DP ρ= -0.37<br>EE ρ= -0.55<br>PA ρ= 0.62	p<0.05<br>p<0.05<br>p<0.05	Association between all domains of the burnout score and the spiritual score.
See et al(37) 2018<br>Large study, across Asia	Compared religious background or belief and high burnout	OR 0.69 (0.49–0.96)	P- 0.027	Religiosity was found to be protective against burnout in medical doctors (and nurses).
Shetach et al(38) 2015<br>Israel	Job satisfaction scores for Christians were compared with non-Christians	Religion β<br>Christian 0.46<br>Non-Christian 0.41	p>0.05	No association
St Onge et al(39) 2022<br>USA	Spiritual question was compared with burnout scores	Personal burnout β= -0.77<br>Work burnout β= -1.87885<br>Patient burnout β= −1.65<br><br>Odds ratio for overall burnout: 0.55(0.23-1.31)	p>0.05<br>p>0.05<br>p>0.05<br><br>p= 0.18	No association
Suttle et al(40) 2020<br>Asia	Mean MBI scores were compared with yes/no to spiritual question	Mean<br>EE- 26.77<br>DP- 11.278<br>PA-36.620	 p= 0.036<br>p= 0.011<br>p= 0.021	Higher burnout scores were associated with identifying as non-spiritual.
Teixeira et al(41) 2013<br>Portugal	Burnout scores in the religious were compared to non-religious	Odds ratio for burnout: 0.907	p=0.747	No association

Watson(42) 2019 USA	Mean burnout domain scores were compared with religious/spiritual question	Spirituality/religion	Disagree	Neutral	Agree		Higher emotional exhaustion and lower personal accomplishment were correlated with those who disagreed with the spiritual question. No correlation with depersonalization. One-way ANOVA showed no difference between means.
		Mean EE	3.98	3.80	3.41	p=0.36	
		Mean DP	3.95	3.71	3.18	p=0.13	
		Mean PA	5.31	5.87	5.65	p=0.43	

MBI- Maslach Burnout Inventory DP- Depersonalisation EE- Emotional Exhaustion PA- Personal Accomplishment
SIBS- Hatch Spiritual Involvement and Beliefs Scale WRSI- Work Related Strain Inventory
Brief COPE- Brief Coping Orientation to Problems Experienced Inventory

1. Alosaimi FD, Alawad HS, Alamri AK, Saeed AI, Aljuaydi KA, Alotaibi AS, et al. Stress and coping among consultant physicians working in Saudi Arabia. Annals of Saudi Medicine. 2018;38(3):214-24.
2. Antonsdottir I, Rushton CH, Nelson KE, Heinze KE, Swoboda SM, Hanson GC. Burnout and moral resilience in interdisciplinary healthcare professionals. Journal of Clinical Nursing. 2022;31(1):196-208.
3. Azoulay E, De Waele J, Ferrer R, Staudinger T, Borkowska M, Povoa P, et al. Symptoms of burnout in intensive care unit specialists facing the COVID-19 outbreak. Annals of Intensive Care. 2020;10(1):110.
4. Baruah A, Das S, Dutta A, Das B, Sharma T, Hazarika M. DEGREE AND FACTORS OF BURNOUT AMONG EMERGENCY HEALTHCARE WORKERS IN INDIA. Int J Sci Res (Ahmedabad). 2019;8(4):41-5.
5. Ben-Itzhak S, Dvash J, Maor M, Rosenberg N, Halpern P. Sense of meaning as a predictor of burnout in emergency physicians in Israel: a national survey. Clinical and Experimental Emergency Medicine. 2015;2(4):217-25.
6. Büssing A, Lotzke D, Glockler M, Heusser P. Influence of Spirituality on Cool Down Reactions, Work Engagement, and Life Satisfaction in Anthroposophic Health Care Professionals. Evidence-Based Complementary and Alternative Medicine. 2015.
7. Chor WPD, Ng WM, Cheng L, Situ W, Chong JW, Ng LYA, et al. Burnout amongst emergency healthcare workers during the COVID-19 pandemic: A multi-center study. American Journal of Emergency Medicine. 2021;46:700-2.
8. Clark L, Leedy S, McDonald L, Muller B, Lamb C, Mendez T, et al. Spirituality and job satisfaction among hospice interdisciplinary team members. Journal of Palliative Medicine. 2007;10(6):1321-8.
9. Correia I, Almeida AE. Organizational Justice, Professional Identification, Empathy, and Meaningful Work During COVID-19 Pandemic: Are They Burnout Protectors in Physicians and Nurses? FRONTIERS IN PSYCHOLOGY. 2020;11.
10. Das S, Barman S, Datta S, Bardhan N, Baishya M, Das B, et al. Degree of burnout among emergency healthcare workers and factors influencing level of burnout: a pilot study. Delhi Psychiatry Journal. 2016;19:36-47.
11. Doolittle BR, Windish DM, Seelig CB. Burnout, coping, and spirituality among internal medicine resident physicians. Journal of graduate medical education. 2013;5(2):257-61.

For peer review only

12. Doolittle BR. Association of Burnout with Emotional Coping Strategies, Friendship, and Institutional Support Among Internal Medicine Physicians. Journal of Clinical Psychology in Medical Settings. 2020.

13. Doolittle BR, Windish DM. Correlation of burnout syndrome with specific coping strategies, behaviors, and spiritual attitudes among interns at Yale University, New Haven, USA. Journal of Educational Evaluation for Health Professions. 2015;12.

14. Frank E, McMurray J, Linzer M, Elon L. Career satisfaction of US women physicians - Results from the Women Physicians' Health Study. ARCHIVES OF INTERNAL MEDICINE. 1999;159(13):1417-26.

15. Glasberg J, Horiuti L, Novais M, Canavezzi A, Miranda V, Chicoli F, et al. Prevalence of the burnout syndrome among Brazilian medical oncologists. REVISTA DA ASSOCIACAO MEDICA BRASILEIRA. 2007;53(1):85-9.

16. Glebocka A, Lisowska E, Glebocka A, Lisowska E. Professional burnout and stress among polish physicians explained by the Hobfoll resources theory. JOURNAL OF PHYSIOLOGY AND PHARMACOLOGY. 2007;58:243-52.

17. Gribben J, Kase S, Waldman E, Weintraub A, Gribben JL, Kase SM, et al. A Cross-Sectional Analysis of Compassion Fatigue, Burnout, and Compassion Satisfaction in Pediatric Critical Care Physicians in the United States*. PEDIATRIC CRITICAL CARE MEDICINE. 2019;20(3):213-22.

18. Guay MD, Reyes Donoso MM, Lopez Saca JM, Riveros Rios ME, Pastrana T. Spirituality and Religiosity and Burnout in Latin-American Palliative Care Health Care Professionals (LAPC) (S818). Journal of Pain and Symptom Management. 2019;57(2):490-1.

19. Guest RS, Baser R, Li Y, Scardino PT, Brown AE, Kissane DW. Cancer surgeons' distress and well-being, II: modifiable factors and the potential for organizational interventions. Annals of surgical oncology. 2011;18(5):1236-42.

20. Harper L, Alshammari D, Ferdynus C, Kalfa N. Burnout amongst members of the French-speaking Society of Pediatric and Adolescent Urology (SFUPA). Are there specific risk factors? Journal of pediatric urology. 2020;16(4):482-6.

21. Koh MYH, Chong PH, Neo PSH, Ong YJ, Yong WC, Ong WY, et al. Burnout, psychological morbidity and use of coping mechanisms among palliative care practitioners: A multi-centre cross-sectional study. Palliative Medicine. 2015;29(7):633-42.

22. Lal A, Tharyan A, Tharyan P. The prevalence, determinants and the role of empathy and religious or spiritual beliefs on job stress, job satisfaction, coping, burnout, and mental health in medical and surgical faculty of a teaching hospital: A cross-sectional survey. La Revue de Médecine Interne. 2020.

23. Leonelli LB, Andreoni S, Martins P, Kozasa EH, Salvo VLd, Sopezki D, et al. Perceived stress among Primary Health Care Professionals in Brazil. Estresse percebido em profissionais da Estrategia Saude da Familia. 2017;20(2):286-98.

24. Leu S, Vuille-dit-Bille R, Fink L, Soll C, Staerkle R, Leu S, et al. Burnout in Swiss and Australian surgeons in training-a cross-sectional study. EUROPEAN SURGERY-ACTA CHIRURGICA AUSTRIACA.

25. Macuka I, Junakovic I, Bozic D, Macuka I, Junakovic IT, Bozic D. Burnout among Palliative Care Professionals. DRUSTVENA ISTRAZIVANJA. 2020;29(2):287-308.

26. Macuka I, Tucak Junakovic I. A Cross-Sectional Study of Job Satisfaction and Intention to Leave Job in Palliative Care in Croatia. Journal of palliative care. 2021:8258597211046704.

27. Mantri S, Lawson JM, Wang ZZ, Koenig HG. Prevalence and Predictors of Moral Injury Symptoms in Health Care Professionals. JOURNAL OF NERVOUS AND MENTAL DISEASE. 2021;209(3):174-80.

28. McKinley N, McCain RS, Convie L, Clarke M, Dempster M, Campbell WJ, et al. Resilience, burnout and coping mechanisms in UK doctors: a cross-sectional study. BMJ Open. 2020;10(1):e031765.

29. McKinley NC. Resilience, professional quality of life and coping mechanisms in doctors and medical students.: Queens University Belfast; 2021.

30. Ntantana A, Matamis D, Savvidou S, Giannakou M, Gouva M, Nakos G, et al. Burnout and job satisfaction of intensive care personnel and the relationship with personality and religious traits: An observational, multicenter, cross-sectional study. Intensive and Critical Care Nursing. 2017;41:11-7.

31. Purvis T, Saylor D, Powell B, Biba G, Conti D, Crowe T, et al. Burnout and Resilience Among Neurosciences Critical Care Unit Staff. NEUROCRITICAL CARE. 2019;31(2):406-10.
32. Ramondetta L, Urbauer D, Brown A, Richardson G, Thaker P, Koenig H, et al. Work related stress among gynecologic oncologists. GYNECOLOGIC ONCOLOGY. 2011;123(2):365-9.
33. Roslan NS, Yusoff MSB, Ab Razak A, Morgan K, Shauki NIA, Kukreja A, et al. Training Characteristics, Personal Factors and Coping Strategies Associated with Burnout in Junior Doctors: A Multi-Center Study. HEALTHCARE. 2021;9(9).
34. Salmoirago-Blotcher E, Fitchett G, Leung K, Volturo G, Boudreaux E, Crawford S, et al. An exploration of the role of religion/spirituality in the promotion of physicians' wellbeing in Emergency Medicine. Preventive Medicine Reports. 2016;3:189-95.
35. Schmidt C, Roffler M. Coping with the Practice of Medicine: Religion, Spirituality, and Other Personal Strategies. JOURNAL OF RELIGION & HEALTH. 2021;60(3):2092-108.
36. Schmidt C, Roffler M. Who isn't burned out? The role of spirituality in graduate medical education (GME). 2018.
37. See K, Zhao M, Nakataki E, Chittawatanarat K, Fang W, Faruq M, et al. Professional burnout among physicians and nurses in Asian intensive care units: a multinational survey. INTENSIVE CARE MEDICINE. 2018;44(12):2079-90.
38. Shetach A, Marcus O, Shetach A, Marcus O. The critical managerial capabilities of medical and nursing managers in an Israeli hospital. EVIDENCE-BASED HRM-A GLOBAL FORUM FOR EMPIRICAL SCHOLARSHIP. 2015;3(1):81-102.
39. St Onge JE, Allespach H, Diaz Y, Poitier A, Tamariz L, Paidas C, et al. Burnout: exploring the differences between U.S. and international medical graduates. BMC medical education. 2022;22(1):69.
40. Suttle M, Chase M, Sasser W, Moore-Clingenpeel M, Maa T, Werner J, et al. Burnout in Pediatric Critical Care Medicine Fellows*. CRITICAL CARE MEDICINE. 2020;48(6):872-80.
41. Teixeira C, Ribeiro O, Fonseca AM, Carvalho AS. Burnout in intensive care units - a consideration of the possible prevalence and frequency of new risk factors: A descriptive correlational multicentre study. BMC Anesthesiology. 2013;13:38.
42. Watson AG, Saggar V, MacDowell C, McCoy JV. Self-reported modifying effects of resilience factors on perceptions of workload, patient outcomes, and burnout in physician-attendees of an international emergency medicine conference. Psychology, health & medicine. 2019;24(10):1220-34.

For peer review only

Table 2: Study details, AXIS quality assessment, spiritual wellness assessment and burnout score used

Study	Study design	AXIS Quality rating	Population	Measure of spirituality	Measure of burnout
Alosaimi et al(1) 2018 Saudi Arabia	Cross-sectional study	16	582 consultant physicians	Brief COPE (2 items to score religious coping)	Perceived stress Scale
Antonsdottir et al(2) 2022 USA	Cross-sectional study	12	639 (96 physicians)	Religious affiliation (including 'spiritual but not religious")	MBI
Azoulay(3) 2020 Europe	Cross-sectional study	12	1132 respondents- ICU specialists	Self-rated religiosity 1-100	MBI
Baruah(4) 2019 India	Cross-sectional study	15	68 participants (doctors, nurses, and paramedics)- Emergency Department	Religion	MBI
Ben-Itzak(5) 2015 Israel	Cross-sectional study	10	70 emergency physicians	"My job gives me meaning" yes/no<br>Open ended questions: 1. Self-fulfilment<br>2. Meaningful relationships<br>3. Excitement and adrenaline rush<br>4. Providing meaningful assistance.	MBI
Büssing et al(6) 2014 Germany	Cross-sectional study	16	489 anthroposophic medical staff (41% physicians)	Aspects of Spirituality Questionnaire (ASQ)	Cool down Index<br>Utrecht Work engagement scale.

Chor et al(7) 2021 Singapore	Cross-sectional study	14	337 participants (127 doctors) Emergency department	Use of religious coping	Copenhagen Burnout Inventory
Clark(8) 2007 USA	Cross-sectional study	14	215 hospice staff, 2.3% physicians	Jarel spiritual wellbeing scale	8-item job satisfaction scale
Correia et al(9) 2020 Portugal	Cross-sectional study	12	497 health professionals working during COVID-19 (229 physicians)	"Regardless of whether you belong to a particular religion, how religious would you say you are?"	Oldenburg Burnout Inventory
Das(10) 2016 India	Cross-sectional study	12	7 emergency department participants (4 doctors)	Religion	MBI
Doolittle et al(11)2013 USA	Cross-sectional study	17	108 hospital residents	Hatch Spiritual Involvement and Beliefs Scale (SIBS), chosen to try and avoid cultural bias.	MBI
Doolittle(12) 2020 USA	Cross-sectional study	17	337 internal medicine physicians	COPE and DUREL, self reported spiritual and/or religious	ProQoL (domains of burnout, secondary stress, and compassion satisfaction)
Doolittle and Windish(13) 2015 USA	Cross-sectional study	15	Interns 66 in the summer, and 53 in winter cohorts	Coping Orientation to Problems Experienced (COPE), Hatch SIBS, self perception of spirituality and religiosity.	MBI

Frank(14) 1999 USA	Cross-sectional study	17	4501 female physicians	"Religious identification" none, low strong or very strong	"Are you generally satisfied with your career?" "If you lived your life again, would you become a physician?"
Glasberg et al(15) 2007 Brazil	Cross-sectional study	16	102 oncologists	Religious affiliation	MBI
Glebocka et al(16) 2007 Poland	Cross-sectional study	8	77 people (41 doctors)	Hobfoll's Questionnaire of Self-Assessment of Gains and Losses)7 statements of 'spiritual resources' gained in last 12 months)	MBI
Gribben et al(17) 2019 USA	Cross-sectional study	18	390 paediatric emergency medicine trainees	Prayer/meditation as self care activity	Compassion Fatigue and Satisfaction Self-Test for Helpers (adapted)
Guay et al(18) 2019 Americas	Cross-sectional study	8	221 (RR 63%) health care professions, 58.8% physicians,	Importance of religion<br>Importance of spirituality<br>Self-definition as religious person<br>Self-definition as spiritual person<br>Frequency of religious practice (praying/meditation)	Respondents categorised as burned out if they agreed with: ''I am definitely burning out and have one or more symptoms of burnout, such as physical and emotional exhaustion,'' ''The symptoms of burnout that I'm experiencing won't go away. I think about frustration at work a lot,'' ''I feel completely burned out and often wonder if I can go on. I am at the point where I may need some changes or may need to seek some sort of help.''<br><br>Respondents were categorized as not burned out if they agreed to either of the statements: (1) ''I am definitely burning out and have one or more symptoms of burnout, such as physical and emotional exhaustion, (2) Occasionally I am under stress, and I don't always have as much energy as I once did, but I don't feel burned out.''

Guest et al(19) 2011 USA	Cross-sectional study	10	72 cancer surgeons	Linear Analog Scale Assessment of Quality of Life (LASA QOL) which includes spiritual QoL	MBI
Harper et al(20) 2020 USA	Cross-sectional study	18	70 Urologists	Believer/non-believer	MBI
Koh et al(21) 2015 Singapore	Cross-sectional study	19	273 palliative care staff (28.1% doctors)	Single question: “Do you consider yourself spiritual?”	MBI
Lal et al(22) 2020 India	Cross-sectional study	19	345 of medical and surgical faculty	Single question- “My religious or spiritual beliefs strongly influence my approach to my work and patients.”	MBI<br>Job stress and job satisfaction questionnaire<br>Jefferson scale of physician empathy
Leonelli et al(23) 2017 Brazil	Cross-sectional study	19	450 primary health care professionals (27 doctors)	Religious activity	Perceived stress scale (PSS)continuous
Leu et al(24) 2020 Switzerland and Australia	Cross-sectional study	16	412 participants (237 Swiss and 175 Australian)	Religion	MBI
Macuka et al(25) 2020 Croatia	Cross-sectional study	12	123 palliative care staff (18 doctors)	“Whether or not you consider yourself a believer or not, how much of a spiritual person you consider yourself, that is, how much you are interested in the sacred or the supernatural?” likert scale 1-5	Oldenburg burnout inventory
Macuka et al 2021(26) Croatia		9			
Mantri et al 2021(27) USA	Cross-sectional study	18	181 participants	Religious affiliation, and ‘religious commitment’ using Belief into Action (BIAC) scale	Moral Injury Symptom Scale-Healthcare Professionals version and MBI

McKinley et al 2020(28) full data from Dr McKinley's thesis(29)	Cross-sectional study	17	1382 participants, 69% hospital, 31% primary care	BRIEF COPE, includes religious coping	ProQOL-V includes burnout, compassion satisfaction and secondary traumatic stress sub domains.
Ntantana et al(30) 2017 Greece	Cross-sectional study	18	149 physicians, 320 nurses from intensive care	Religious and Spiritual attitudes questionnaire(31), designed by Büssing for use with patients, rather than staff. The authors report it was chosen as it avoids exclusive religious terminology, however in the paper by Büssing referred to, they report that a positive attitude towards religion and spirituality is necessary for some aspects of the scale.	MBI
Purviss et al(32) 2019 USA	Cross-sectional study	16	64 neurosciences critical care staff (10 doctors)	Religion	MBI
Ramondetta et al(33) 2011 Greece	Cross-sectional study	15	273 oncologists	Religious affiliation, self-rated religiosity and spirituality, Intrinsic Religiosity Survey, and Duke religion Index (DRI- a measure organisational religiosity)	Work related strain inventory (WRSI)- correlates with MBI
Roslan et al(34) 2021 Malaysia	Cross-sectional study	20	754 hospital interns	BRIEF COPE, includes religious coping, and descriptors of regular versus irregular spirituality routines based on several major religions professed in the country.	Copenhagen Burnout Inventory
Salmoirago-Blotcher(35) 2016 USA	Cross-sectional study	16	138 emergency physicians	Religious affiliation, Self-rated spirituality, Fetzer Institute Multidimensional Measurement of Religiousness/Spirituality for Use in Health Research- used to assess religious/spiritual practice, and religious commitment ("I try hard to carry my religious beliefs over into all my other dealings in life), questions added to assess religious rest, and spiritual counsel.	2 item MBI

Schmidt and Roffler(36) 2021<br><br>Quantitative data given by author, unpublished(37)	Cross-sectional study	10	65 medical education faculty	The<br>Functional Assessment of Chronic Illness Therapy-Spiritual Well-Being Scale, a modified version for non-illness (FACIT-Sp-Non-Illness)	MBI
See et al(38) 2018<br>Large study, across Asia	Cross-sectional study	16	992 intensive care physicians	Religious background or belief	MBI
Shetach et al(39) 2015<br>Israel	Cross-sectional study	13	107 respondents (40 doctors)	Religion- (Christian vs non-Christian)	Job satisfaction questionnaire
St Onge et al(40) 2022<br>USA	Cross-sectional study	17	222 interns	“My spiritual beliefs are very important in helping me cope with stressors.”	Copenhagen Burnout Inventory
Suttle et al(41) 2020<br>Asia	Cross-sectional study	12	47 programme directors and 187 fellows in paediatric critical care	“Do you consider yourself to be a spiritual person?” yes/no	MBI
Teixeira et al(42) 2013<br>Portugal	Cross-sectional study	14	300 Intensive Care staff (82 physicians)	Religion yes/no	MBI
Watson(43) 2019<br>USA	Cross-sectional study	13	54 emergency physicians	Single question- “spirituality/religion is important in my life”	MBI

MBI-Maslach Burnout Inventory

1. Alosaimi FD, Alawad HS, Alamri AK, Saeed AI, Aljuaydi KA, Alotaibi AS, et al. Stress and coping among consultant physicians working in Saudi Arabia. Annals of Saudi Medicine. 2018;38(3):214-24.
2. Antonsdottir I, Rushton CH, Nelson KE, Heinze KE, Swoboda SM, Hanson GC. Burnout and moral resilience in interdisciplinary healthcare professionals. Journal of Clinical Nursing. 2022;31(1):196-208.
3. Azoulay E, De Waele J, Ferrer R, Staudinger T, Borkowska M, Povoa P, et al. Symptoms of burnout in intensive care unit specialists facing the COVID-19 outbreak. Annals of Intensive Care. 2020;10(1):110.
4. Baruah A, Das S, Dutta A, Das B, Sharma T, Hazarika M. DEGREE AND FACTORS OF BURNOUT AMONG EMERGENCY HEALTHCARE WORKERS IN INDIA. Int J Sci Res (Ahmedabad). 2019;8(4):41-5.
5. Ben-Itzhak S, Dvash J, Maor M, Rosenberg N, Halpern P. Sense of meaning as a predictor of burnout in emergency physicians in Israel: a national survey. Clinical and Experimental Emergency Medicine. 2015;2(4):217-25.
6. Büssing A, Lotzke D, Glockler M, Heusser P. Influence of Spirituality on Cool Down Reactions, Work Engagement, and Life Satisfaction in Anthroposophic Health Care Professionals. Evidence-Based Complementary and Alternative Medicine. 2015.
7. Chor WPD, Ng WM, Cheng L, Situ W, Chong JW, Ng LYA, et al. Burnout amongst emergency healthcare workers during the COVID-19 pandemic: A multi-center study. American Journal of Emergency Medicine. 2021;46:700-2.
8. Clark L, Leedy S, McDonald L, Muller B, Lamb C, Mendez T, et al. Spirituality and job satisfaction among hospice interdisciplinary team members. Journal of Palliative Medicine. 2007;10(6):1321-8.
9. Correia I, Almeida AE. Organizational Justice, Professional Identification, Empathy, and Meaningful Work During COVID-19 Pandemic: Are They Burnout Protectors in Physicians and Nurses? FRONTIERS IN PSYCHOLOGY. 2020;11.
10. Das S, Barman S, Datta S, Bardhan N, Baishya M, Das B, et al. Degree of burnout among emergency healthcare workers and factors influencing level of burnout: a pilot study. Delhi Psychiatry Journal. 2016;19:36-47.
11. Doolittle BR, Windish DM, Seelig CB. Burnout, coping, and spirituality among internal medicine resident physicians. Journal of graduate medical education. 2013;5(2):257-61.
12. Doolittle BR. Association of Burnout with Emotional Coping Strategies, Friendship, and Institutional Support Among Internal Medicine Physicians. Journal of Clinical Psychology in Medical Settings. 2020.
13. Doolittle BR, Windish DM. Correlation of burnout syndrome with specific coping strategies, behaviors, and spiritual attitudes among interns at Yale University, New Haven, USA. Journal of Educational Evaluation for Health Professions. 2015;12.
14. Frank E, McMurray J, Linzer M, Elon L. Career satisfaction of US women physicians - Results from the Women Physicians' Health Study. ARCHIVES OF INTERNAL MEDICINE. 1999;159(13):1417-26.
15. Glasberg J, Horiuti L, Novais M, Canavezzi A, Miranda V, Chicoli F, et al. Prevalence of the burnout syndrome among Brazilian medical oncologists. REVISTA DA ASSOCIACAO MEDICA BRASILEIRA. 2007;53(1):85-9.
16. Glebocka A, Lisowska E, Glebocka A, Lisowska E. Professional burnout and stress among polish physicians explained by the Hobfoll resources theory. JOURNAL OF PHYSIOLOGY AND PHARMACOLOGY. 2007;58:243-52.
17. Gribben J, Kase S, Waldman E, Weintraub A, Gribben JL, Kase SM, et al. A Cross-Sectional Analysis of Compassion Fatigue, Burnout, and Compassion Satisfaction in Pediatric Critical Care Physicians in the United States*. PEDIATRIC CRITICAL CARE MEDICINE. 2019;20(3):213-22.
18. Guay MD, Reyes Donoso MM, Lopez Saca JM, Riveros Rios ME, Pastrana T. Spirituality and Religiosity and Burnout in Latin-American Palliative Care Health Care Professionals (LAPC) (S818). Journal of Pain and Symptom Management. 2019;57(2):490-1.

For peer review only

19. Guest RS, Baser R, Li Y, Scardino PT, Brown AE, Kissane DW. Cancer surgeons' distress and well-being, II: modifiable factors and the potential for organizational interventions. Annals of surgical oncology. 2011;18(5):1236-42.
20. Harper L, Alshammari D, Ferdynus C, Kalfa N. Burnout amongst members of the French-speaking Society of Pediatric and Adolescent Urology (SFUPA). Are there specific risk factors? Journal of pediatric urology. 2020;16(4):482-6.
21. Koh MYH, Chong PH, Neo PSH, Ong YJ, Yong WC, Ong WY, et al. Burnout, psychological morbidity and use of coping mechanisms among palliative care practitioners: A multi-centre cross-sectional study. Palliative Medicine. 2015;29(7):633-42.
22. Lal A, Tharyan A, Tharyan P. The prevalence, determinants and the role of empathy and religious or spiritual beliefs on job stress, job satisfaction, coping, burnout, and mental health in medical and surgical faculty of a teaching hospital: A cross-sectional survey. La Revue de Médecine Interne. 2020.
23. Leonelli LB, Andreoni S, Martins P, Kozasa EH, Salvo VLd, Sopezki D, et al. Perceived stress among Primary Health Care Professionals in Brazil. Estresse percebido em profissionais da Estrategia Saude da Familia. 2017;20(2):286-98.
24. Leu S, Vuille-dit-Bille R, Fink L, Soll C, Staerkle R, Leu S, et al. Burnout in Swiss and Australian surgeons in training-a cross-sectional study. EUROPEAN SURGERY-ACTA CHIRURGICA AUSTRIACA.
25. Macuka I, Junakovic I, Bozic D, Macuka I, Junakovic IT, Bozic D. Burnout among Palliative Care Professionals. DRUSTVENA ISTRAZIVANJA. 2020;29(2):287-308.
26. Macuka I, Tucak Junakovic I. A Cross-Sectional Study of Job Satisfaction and Intention to Leave Job in Palliative Care in Croatia. Journal of palliative care. 2021:8258597211046704.
27. Mantri S, Lawson JM, Wang ZZ, Koenig HG. Prevalence and Predictors of Moral Injury Symptoms in Health Care Professionals. JOURNAL OF NERVOUS AND MENTAL DISEASE. 2021;209(3):174-80.
28. McKinley N, McCain RS, Convie L, Clarke M, Dempster M, Campbell WJ, et al. Resilience, burnout and coping mechanisms in UK doctors: a cross-sectional study. BMJ Open. 2020;10(1):e031765.
29. McKinley NC. Resilience, professional quality of life and coping mechanisms in doctors and medical students.: Queens University Belfast; 2021.
30. Ntantana A, Matamis D, Savvidou S, Giannakou M, Gouva M, Nakos G, et al. Burnout and job satisfaction of intensive care personnel and the relationship with personality and religious traits: An observational, multicenter, cross-sectional study. Intensive and Critical Care Nursing. 2017;41:11-7.
31. Büssing A, Ostermann T, Matthiessen PF. Role of religion and spirituality in medical patients: confirmatory results with the SpREUK questionnaire. Health and quality of life outcomes. 2005;3(1):10.
32. Purvis T, Saylor D, Powell B, Biba G, Conti D, Crowe T, et al. Burnout and Resilience Among Neurosciences Critical Care Unit Staff. NEUROCRITICAL CARE. 2019;31(2):406-10.
33. Ramondetta L, Urbauer D, Brown A, Richardson G, Thaker P, Koenig H, et al. Work related stress among gynecologic oncologists. GYNECOLOGIC ONCOLOGY. 2011;123(2):365-9.
34. Roslan NS, Yusoff MSB, Ab Razak A, Morgan K, Shauki NIA, Kukreja A, et al. Training Characteristics, Personal Factors and Coping Strategies Associated with Burnout in Junior Doctors: A Multi-Center Study. HEALTHCARE. 2021;9(9).
35. Salmoirago-Blotcher E, Fitchett G, Leung K, Volturo G, Boudreaux E, Crawford S, et al. An exploration of the role of religion/spirituality in the promotion of physicians' wellbeing in Emergency Medicine. Preventive Medicine Reports. 2016;3:189-95.
36. Schmidt C, Roffler M. Coping with the Practice of Medicine: Religion, Spirituality, and Other Personal Strategies. JOURNAL OF RELIGION & HEALTH. 2021;60(3):2092-108.
37. Schmidt C, Roffler M. Who isn't burned out? The role of spirituality in graduate medical education (GME). 2018.

For peer review only

38. See K, Zhao M, Nakataki E, Chittawatanarat K, Fang W, Faruq M, et al. Professional burnout among physicians and nurses in Asian intensive care units: a multinational survey. INTENSIVE CARE MEDICINE. 2018;44(12):2079-90.
39. Shetach A, Marcus O, Shetach A, Marcus O. The critical managerial capabilities of medical and nursing managers in an Israeli hospital. EVIDENCE-BASED HRM-A GLOBAL FORUM FOR EMPIRICAL SCHOLARSHIP. 2015;3(1):81-102.
40. St Onge JE, Allespach H, Diaz Y, Poitier A, Tamariz L, Paidas C, et al. Burnout: exploring the differences between U.S. and international medical graduates. BMC medical education. 2022;22(1):69.
41. Suttle M, Chase M, Sasser W, Moore-Clingenpeel M, Maa T, Werner J, et al. Burnout in Pediatric Critical Care Medicine Fellows*. CRITICAL CARE MEDICINE. 2020;48(6):872-80.
42. Teixeira C, Ribeiro O, Fonseca AM, Carvalho AS. Burnout in intensive care units - a consideration of the possible prevalence and frequency of new risk factors: A descriptive correlational multicentre study. BMC Anesthesiology. 2013;13:38.
43. Watson AG, Saggar V, MacDowell C, McCoy JV. Self-reported modifying effects of resilience factors on perceptions of workload, patient outcomes, and burnout in physician-attendees of an international emergency medicine conference. Psychology, health & medicine. 2019;24(10):1220-34.

For peer review only

Systematic Review Protocol & Support Template

**Title of the review**	A systematic review: Is there a relationship between burnout scores in doctors and their spiritual health
**First reviewer**	Orla Whitehead
**Team of reviewers**	Ishbel Orla Whitehead,<br>Suzanne Moffatt,<br>Stephanie Warwick,<br>Gemma Frances<br>Spiersa, Tafadzwa<br>Patience Kunonga,<br>Eugene Yee Hing Tang,<br>Barbara Hanratty
**Supervisor/Project PI**	Professor Barbara<br>Hanratty
**Clinical Portfolio Group**	
**Project title (if different from review title)**	

**Support** – please state if advice/training or personnel required at each stage	
**SR overview**	
**Protocol development**	“”
**Literature searching**	Advice taken from Catherine Richmond, information scientist at the University
**Quality appraisal**	
**Data Extraction**	“”
**Synthesis**	“”
**Writing up**	“”

For peer review only - http://bmjopen.bmj.com/site/about/guidelines.xhtml

1. Background to review

Brief introduction to the subject of the review, including rationale for undertaking the review and overall aim

Burnout and moral injury are two terms used to describe a form of psychological distress associated with work as a doctor. Burnout is defined by the World Health Organisation not as an illness, but as an occupational phenomenon, resulting in exhaustion, mental distance or feelings of negativity or cynicism towards the job, and reduced professional efficacy.[1] Others describe a loss of meaning in work, and objectification of patients and their families, rather than engaging with their humanity.[2] Moral injury is a term taken from military personnel returning from battle, where the realties conflict with our moral and ethical code.[3] It has been argued that doctors caught between patient and societal demands, limited resources, and their own ethical code and training, are suffering injury analogous to soldiers, leaving them wounded and hopeless.[3] Reframing burnout as moral injury replaces a view of a defective individual, to that of a defective system, which could be more effective in terms of change[4], and move the dialogue away from expecting doctors to become more and more resilient in the face of increasing challenges. The co-option of the term from conflict has been critised[5], and arguments made that while moral injury can occur in medicine, burn out is not synonymous, and is more common.[6] While both the terms burnout and moral injury place the issue in the context of occupation, it is evident that mental illness accompanies these issues, as suicide rates for female doctors are up to 4 times those of age-matched controls.[7] With current recruitment and retention issues, we cannot afford to lose good doctors to burnout.[2] Supporting doctors' holistic health has to become a priority, to help prevent and treat burnout and moral injury.

Holistic health includes spiritual health. Literature discussing how doctors should address their patients' spiritual health often mention that self awareness of spiritual health, needs, and distress, is key to be able to begin to meet patients' needs in this area. In a recent survey of GPs in England, 69% described themselves as 'a spiritual person', with 48% also describing themselves as 'a religious person'. These GPs defined spiritual health as self-actualisation and meaning, transcendence and relationships beyond the self, and expressions of spirituality. Self-actualisation included concepts of being able to follow their personal ethical and moral codes. Meaning included having personal meaning to life and relationships. Transcendence included concepts of a spiritual sphere, a concept of a soul, and relationships with communities, friends, family, nature and/or the divine. Expressions of spirituality included both personal and group religious and spiritual practice, for example attendance at church, religious meetings, meditation, prayer and mindfulness practice. Burnout could be seen as contrasting with these definitions of spiritual health, as mental distance from others and the job, negativity and lack of meaning in the job, and lack of relationships with patients and their families. Spiritual health as defined by GPs requires meaning, following their ethical code. Could burnout and spiritual distress among doctors be related phenomena? Viewing burnout through the lens of spiritual health and distress, as well as other lenses and perspectives, could generate potential beneficial interventions for both prevention and treatment of burnout.

Aims:
To investigate the association between burnout in doctors and spiritual health, by conducting a systematic review of studies which use a measure burnout with measures of aspects of spiritual health, spirituality, religiosity or spiritual distress.

For peer review only

2. Specific objectives

1. To investigate the quantity and quality of data addressing doctors' burnout, and spiritual heath
2. To investigate any link between doctors' burnout and spiritual health, spirituality, spiritual distress or religiosity.

For peer review only

For peer review only - http://bmjopen.bmj.com/site/about/guidelines.xhtml

**3. a) Criteria for including studies in the review**<br>If the PICOS format does not fit the research question of interest, please split up the question into separate concepts and put one under each heading	
**i. Population, or participants and conditions of interest**	Qualified doctors/medical practitioners, worldwide of either sex and any age, whether they have experienced any level of burnout or not.
**ii. Interventions or exposures**	Quantification of burnout (for example, use of a burnout inventory such as the Maslach Burnout Inventory, or the Copenhagen Burnout Inventory, or others)
**iii. Comparisons or control groups**	Quantification of spiritual health, spirituality, spiritual distress, or religiosity, either using a scale (for example the Spiritual Wellbeing Scale, Spiritual Distress Scale, or other measures of spiritual health or religiosity) or a yes/no or self-rating of 'spirituality' or religiosity.
**iv. Outcomes of interest**	Associations between spiritual health, spiritual distress or religiosity with burnout scores.
**v. Setting**	Any setting where doctors/medical practitioners work (primary, secondary or tertiary care, including laboratory or other non-patient facing settings)
**vi. Study designs**	All research studies with a quantification of burnout, and spiritual health, wellbeing, distress or religiosity, or similar.

3. b) Criteria for excluding studies not covered in inclusion criteria
Any specific populations excluded, date range, language, whether abstracts or full text available, etc

Medical students, nursing staff, or other hospital staff where doctors are not included.
Course evaluations
Editorials, book chapters, or other non-research study articles
No specific mention of spiritual health, spirituality, or religiosity.
No quantification of burnout, or spiritual health, wellbeing or distress, or similar.

For peer review only - http://bmjopen.bmj.com/site/about/guidelines.xhtml

4. Search methods	
**Electronic databases**<br>Please list all databases that are to be searched and include the interface (eg NHS, EBSCO, etc) and date ranges searched for each	PUBMED/MEDLINE<br>EMBASE<br>PsychInfo<br>Web of Science<br>Scopus
**Other methods used for identifying relevant research**<br>ie contacting experts and reference checking	Reference checking and hand searching of these. Contacting experts in this field.
**Journals hand searched**<br>If any are to be hand searched, please list which journals and date searched from, including a rationale.	

5. Methods of review	
**Details of methods**<br>Number of reviewers, how agreements to be reached and disagreements dealt with, etc.	Data extraction will be carried out by the primary reviewer, with 10% checked by a second reviewer
**Quality assessment**<br>Tools or checklists used with references or URLs	Appraisal of Cross-sectional Studies tool (AXIS).[8]

For peer review only - http://bmjopen.bmj.com/site/about/guidelines.xhtml

**Data extraction** What information is to be collected on each included study. If databases or forms on Word or Excel are used and how this is recorded and by how many reviewers	Custom made word table to be used
**Narrative synthesis** Details of what and how synthesis will be done	Synthesis of findings regarding the research question (are burnout and spiritual health related)<br>Synthesis of measures used for burnout, and spiritual health
**Meta-analysis** Details of what and how analysis and testing will be done. If no meta-analysis is to be conducted, please give reason.	If possible, meta-analysis will be conducted of the relationship between burnout scores (e.g. the Maslach Burnout Inventory) and spiritual health scores (e.g. the Brief COPE). If different validated scores are used, effect sizes will be compared.
**Grading evidence** System used, if any, such as GRADE	

**6. Presentation of results**	
**Additional material** Summary tables, flowcharts, etc, to be included in the final paper	
**Outputs from review** Papers and target journals, conference presentations, reports, etc	Conference presentation at the Society for Academic Primary Care Annual Scientific Meeting<br>Submission to journal

For peer review only

7. Timeline for review – when do you aim to complete each stage of the review	
**Protocol**	July 2020
**Literature searching**	July 2020
**Quality appraisal**	August 2020
**Data extraction**	September 2020
**Synthesis**	September 2020
**Writing up**	October 2020

1. World Heath Organisation. Burn-out an "occupational phenomenon": International Classification of Diseases 2019 [Available from: https://www.who.int/mental_health/evidence/burn-out/en/.
2. Fred HL, Scheid MS. Physician Burnout: Causes, Consequences, and (?) Cures. *Tex Heart Inst J* 2018;45(4):198-202. doi: 10.14503/THIJ-18-6842
3. Talbot SG, Dean W. Physicians aren't 'burning out.'They're suffering from moral injury. *Stat* 2018
4. Dean W, Talbot S, Dean A. Reframing Clinician Distress: Moral Injury Not Burnout. *Fed Pract* 2019;36(9):400-02.
5. Asken MJ. Physician burnout: moral injury is a questionable term. *BMJ* 2019;365:l2375. doi: 10.1136/bmj.l2375
6. Parker J. Moral injury and burnout are not the same 2019 [Available from: https://blogs.bmj.com/bmj/2019/06/06/joshua-parker-moral-injury-and-burnout-are-not-the-same/ accessed 11/12/2019.
7. Practitioner Health Programme. Suicide in doctors and other health professionals 2019 [Available from: https://php.nhs.uk/resources/suicide-in-doctors/ accessed 11/12/2019.
8. Downes MJ, Brennan ML, Williams HC, et al. Development of a critical appraisal tool to assess the quality of cross-sectional studies (AXIS). *BMJ Open* 2016;6(12):e011458. doi: 10.1136/bmjopen-2016-011458

For peer review only

Embase (accessed via Ovid):

1. exp religion/ or religio*.mp. or religiosity.mp. or exp philosophy/ or exp spiritual healing/ or theology/ or sikh.mp. or jain.mp. or bahai.mp. or pagan.mp. or shinto.mp. or cao dai.mp. or zoroast*.mp. or spirit*.mp. or Faith.mp. or holistic health.mp. or existential.mp. or salutogenesis.mp. or spiritual*.mp. or religious personnel/ or chaplain*.mp. or Buddhism.mp. or Christian*.mp. or Hinduism.mp. or islam.mp. or Judaism.mp. or humanism.mp. or Confucian*.mp. or "sense of coherence".mp.

2. exp job stress/ or compassion fatigue.mp. or occupational stress.mp. or burnout.mp. or exp burnout/ or exp Maslach Burnout Inventory/ or "work related stress*".mp. or "occupational stress*".mp. or "work stress*".mp. or "job stress".mp. or Job Satisfaction/ or "job satisfaction".mp. or moral injury.mp. or burn&out.mp.

3. exp medical personnel/ or medical personnel.mp. or physician$.mp. or allergist$.mp. or cardiologists.mp. or dermatologists.mp. or endocrinologists.mp. or gastroenterologists.mp. or general practitioner$.mp. or geriatrician$.mp. or nephrologist$.mp. or neurologist$.mp. or occupational health physician$.mp. or oncologist$.mp. or ophthalmologist$.mp. or pathologist$.mp. or p$ediatrician$.mp. or neonatologist$.mp. or physiatrist$.mp. or pulmonologist$.mp. or psychistrist$.mp. or radiologist$.mp. or rheumatologist$.mp. or surgeon$.mp. or urologist$.mp. or doctor*.mp. or medical staff.mp. or an$esthetist$.mp. or medical school/ or medical faculty.mp.

Filter, from:
https://www.cochranelibrary.com/cdsr/doi/10.1002/14651858.MR000041.pub2/appendices

epidemiology/ or exp clinical study/ or cohort analysis/ or case study/ or follow up/ or cross-sectional study/ or observational study/ or correlational study/ or epidemiologic.ab,ti. Or case control.ab,ti. Or case referent.ab,ti. Or case stud$.ab,ti. Or case series.ab,ti. Or cohort?.ab,ti. Or cross sectional.ab,ti. Or follow up.ab,ti. Or longitudinal.ab,ti. Or retrospective$.ab,ti. Or prospective$.ab,ti. Or observational.ab,ti. Or adverse effect?.ab,ti. or (Controlled before and after).ab,ti. Or Interrupted time series.ab,ti. or Correlational.ab,ti. Or ecological stud$.ab,ti. or Descriptive stud$.ab,ti.

Not Editorial or letter

Psych info(accessed via Ovid)::

For peer review only

1. exp spirituality/ or exp religion/ or exp religiosity/ or exp religious beliefs/ or religious experiences/ or exp soul/ or exp spiritual care/ or religio*.mp. or religiosity.mp. or exp philosophy/ or exp faith/ or theology/ or sikh.mp. or jain.mp. or bahai.mp. or pagan.mp. or shinto.mp. or cao dai.mp. or zoroast*.mp. or spirit*.mp. or Faith.mp. or holistic health.mp. or existential.mp. or salutogenesis.mp. or spiritual*.mp. or chaplain*.mp. or Buddhism.mp. or Christian*.mp. or Hinduism.mp. or islam.mp. or Judaism.mp. or humanism.mp. or Confucian*.mp. or exp philosophies/ or exp religious personnel/ or god concepts/ or theology/ or "sense of coherence".mp.
2. exp occupational stress/ or occupational neurosis/ or "quality of work life"/ or exp moral injury/ or job satisfaction/ or compassion fatigue.mp. or occupational stress.mp. or burnout.mp. or "Maslach Burnout Inventory".mp. or "work related stress*".mp. or "occupational stress*".mp. or "work stress*".mp. or "job stress".mp. or "job satisfaction".mp. or moral injury.mp. or burn&out.mp.
3. exp medical personnel/ or medical personnel.mp. or physician$.mp. or allergist$.mp. or cardiologists.mp. or dermatologists.mp. or endocrinologists.mp. or gastroenterologists.mp. or general practitioner$.mp. or geriatrician$.mp. or nephrologist$.mp. or neurologist$.mp. or occupational health physician$.mp. or oncologist$.mp. or ophthalmologist$.mp. or pathologist$.mp. or p$ediatrician$.mp. or neonatologist$.mp. or physiatrist$.mp. or pulmonologist$.mp. or psychistrist$.mp. or radiologist$.mp. or rheumatologist$.mp. or surgeon$.mp. or urologist$.mp. or doctor*.mp. or medical staff.mp. or an$esthetist$.mp. or medical faculty.mp.
4.
(epidemiologic or case control or case referent or case stud$ or case series or cohort? or cross sectional or follow up or longitudinal or retrospective$ or prospective$ or observational or adverse effect? or (Controlled before and after) or Interrupted time series or Correlational or ecological stud$ or Descriptive stud$).ab,ti. Or exp experimental design or epidemiology/

Medline(accessed via Ovid)::

1. exp philosophy/ or exp religion/ or exp "religion and medicine"/ or exp "religion and psychology"/ or "religion and science"/ or exp religious philosophies/ or exp theology/ or religio*.mp. or sikh.mp. or jain.mp. or bahai.mp. or Humanism.mp. or pagan.mp. or shinto.mp. or cao dai.mp. or zoroast*.mp. or spirit*.mp. or faith.mp. or exp holistic health/ or existential.mp. or salutogenesis.mp. or exp Spiritual Therapies/ or religious personnel/ or holistic health/ or "sense of coherence"/

2.

exp occupational stress/ or compassion fatigue.mp. or occupational stress.mp. or burnout.mp. or caregiver burnout/ or exp burnout, psychological/ or "Maslach Burnout Inventory".mp. or "work related stress*".mp. or exp Occupational Stress/ or "occupational stress*".mp. or "work stress*".mp. or "job stress".mp. or Job Satisfaction/ or "job satisfaction".mp. or moral injury.mp. or "work-life balance"/ or burn&out.mp.

3.

anesthetists/ or anesthesiologists/ or faculty, medical/ or exp medical staff/ or exp physicians/ or medical personnel.mp. or physician$.mp. or allergist$.mp. or cardiologists.mp. or dermatologists.mp. or endocrinologists.mp. or gastroenterologists.mp. or general practitioner$.mp. or geriatrician$.mp. or nephrologist$.mp. or neurologist$.mp. or occupational health physician$.mp. or oncologist$.mp. or ophthalmologist$.mp. or pathologist$.mp. or p$ediatrician$.mp. or neonatologist$.mp. or physiatrist$.mp. or pulmonologist$.mp. or psychistrist$.mp. or radiologist$.mp. or rheumatologist$.mp. or surgeon$.mp. or urologist$.mp. or doctor*.mp. or medical staff.mp. or an$esthetist$.mp. or medical faculty.mp.

4. Epidemiologic Studies/ or exp Case Control Studies/ or exp Cohort Studies/ or Cross-Sectional Studies/ or (epidemiologic adj (study or studies)).ab,ti. Or case control.ab,ti. Or (cohort adj (study or studies)).ab,ti. Or cross sectional.ab,ti. Or cohort analy$.ab,ti. or (follow up adj (study or studies)).ab,ti. Or longitudinal.ab,ti. Or retrospective$.ab,ti. Or prospective$.ab,ti. Or (observ$ adj3 (study or studies)).ab,ti. Or adverse effect?.ab,ti.

5. editorial or letter

Web of science accessed via Clarivate:

1. TS=(religio* or philosop* or spirit* or theolog* or sikh or jain or bahai or pagan or shinto or "cao dai" or zoroast* or Faith or "holistic health" or existential or salutogenesis or spiritual* or chaplain* or Buddh* or Christian* or Hindu* or islam* or Juda* or humanism or Confucian* or "sense of coherence")
2. TS=(burnout or "compassion fatigue" or "Maslach Burnout Inventory" or "work related stress*" or "occupational stress*" or "work stress*" or "job stress" or "job satisfaction" or "moral injury" or burn&out)

3. (TS=("medical personnel" or physician$ or allergist$ or cardiologist$ or dermatologist$ or endocrinologist$ or gastroenterologist$ or general practitioner$ or geriatrician$ or nephrologist$ or neurologist$ or occupational health physician$ or oncologist$ or ophthalmologist$ or pathologist$ or p$ediatrician* or neonatologist$ or physiatrist$ or pulmonologist$ or psychistrist$ or radiologist$ or rheumatologist$ or surgeon$ or urologist$ or doctor* or "medical staff" or an$esthetist* or "medical school" or "medical faculty")
4. AND DOCUMENT TYPES: (Article OR Abstract of Published Item OR Correction OR Correction, Addition OR Data Paper OR Database Review OR Discussion OR Early Access OR Meeting Abstract OR Meeting Summary OR News Item OR Note OR Proceedings Paper OR Record Review OR Review OR Script)

Scopus accessed via https://www.scopus.com/home.uri:

1. Title-abs-key(religio* or philosop* or spirit* or theolog* or sikh or jain or bahai or pagan or shinto or "cao dai" or zoroast* or Faith or "holistic health" or existential or salutogenesis or spiritual* or chaplain* or Buddh* or Christian* or Hindu* or islam* or Juda* or humanism or Confucian* or "sense of coherence")
2. Title-abs-key(burnout or "compassion fatigue" or "Maslach Burnout Inventory" or "work related stress*" or "occupational stress*" or "work stress*" or "job stress" or "job satisfaction" or "moral injury" or burn&out)
3. Title-abs-key("medical personnel" or physician$ or allergist$ or cardiologist$ or dermatologist$ or endocrinologist$ or gastroenterologist$ or general practitioner$ or geriatrician$ or nephrologist$ or neurologist$ or occupational health physician$ or oncologist$ or ophthalmologist$ or pathologist$ or p$ediatrician* or neonatologist$ or physiatrist$ or pulmonologist$ or psychistrist$ or radiologist$ or rheumatologist$ or surgeon$ or urologist$ or doctor* or "medical staff" or an$esthetist* or "medical school" or "medical faculty")

All searches concluded by 8th March 2022

PRISMA 2020 for Abstracts Checklist

Section and Topic	Item #	Checklist item	Reported (Yes/No)
**TITLE**			
Title	1	Identify the report as a systematic review.	yes
**BACKGROUND**			
Objectives	2	Provide an explicit statement of the main objective(s) or question(s) the review addresses.	yes
**METHODS**			
Eligibility criteria	3	Specify the inclusion and exclusion criteria for the review.	yes
Information sources	4	Specify the information sources (e.g. databases, registers) used to identify studies and the date when each was last searched.	yes
Risk of bias	5	Specify the methods used to assess risk of bias in the included studies.	yes
Synthesis of results	6	Specify the methods used to present and synthesise results.	yes
**RESULTS**			
Included studies	7	Give the total number of included studies and participants and summarise relevant characteristics of studies.	yes
Synthesis of results	8	Present results for main outcomes, preferably indicating the number of included studies and participants for each. If meta-analysis was done, report the summary estimate and confidence/credible interval. If comparing groups, indicate the direction of the effect (i.e. which group is favoured).	yes
**DISCUSSION**			
Limitations of evidence	9	Provide a brief summary of the limitations of the evidence included in the review (e.g. study risk of bias, inconsistency and imprecision).	yes
Interpretation	10	Provide a general interpretation of the results and important implications.	yes
**OTHER**			
Funding	11	Specify the primary source of funding for the review.	yes
Registration	12	Provide the register name and registration number.	yes

For peer review only

From: Page MJ, McKenzie JE, Bossuyt PM, Boutron I, Hoffmann TC, Mulrow CD, et al. The PRISMA 2020 statement: an updated guideline for reporting systematic reviews. BMJ 2021;372:n71. doi: 10.1136/bmj.n71

For more information, visit: http://www.prisma-statement.org/

For peer review only

PRISMA-S Checklist

Section/topic		Checklist item<br>Checklist	Location(s) Reported
INFORMATION SOURCES AND METHODS			
Database name	1	Name each individual database searched, stating the platform for each.	search file
Multi-database searching	2	If databases were searched simultaneously on a single platform, state the name of the platform, listing all of the databases searched.	n/a
Study registries	3	List any study registries searched.	none
Online resources and browsing	4	Describe any online or print source purposefully searched or browsed (e.g., tables of contents, print conference proceedings, web sites), and how this was done.	none
Citation searching	5	Indicate whether cited references or citing references were examined, and describe any methods used for locating cited/citing references (e.g., browsing reference lists, using a citation index, setting up email alerts for references citing included studies).	main document methods
Contacts	6	Indicate whether additional studies or data were sought by contacting authors, experts, manufacturers, or others.	main document methods
Other methods	7	Describe any additional information sources or search methods used.	none
SEARCH STRATEGIES			
Full search strategies	8	Include the search strategies for each database and information source, copied and pasted exactly as run.	search file
Limits and restrictions	9	Specify that no limits were used, or describe any limits or restrictions applied to a search (e.g., date or time period, language, study design) and provide justification for their use.	search file and main document
Search filters	10	Indicate whether published search filters were used (as originally designed or modified), and if so, cite the filter(s) used.	search file and main document methods
Prior work	11	Indicate when search strategies from other literature reviews were adapted or reused for a substantive part or all of the search, citing the previous review(s).	n/a
Updates	12	Report the methods used to update the search(es) (e.g., rerunning searches, email alerts).	main document methods
Dates of searches	13	For each search strategy, provide the date when the last search occurred.	main document methods and search document

For peer review only

PEER REVIEW			
Peer review	14	Describe any search peer review process.	Main document methods
MANAGING RECORDS			
Total Records	15	Document the total number of records identified from each database and other information sources.	Prisma diagram
Deduplication	16	Describe the processes and any software used to deduplicate records from multiple database searches and other information sources.	Main document methods

PRISMA-S: An Extension to the PRISMA Statement for Reporting Literature Searches in Systematic Reviews Rethlefsen ML, Kirtley S, Waffenschmidt S, Ayala AP, Moher D, Page M], Koffel JB, PRISMA-S Group. Last updated February 27, 2020.

For peer review only

PRISMA 2020 Checklist

Section and Topic	Item #	Checklist item	Location where item is reported
**TITLE**			
Title	1	Identify the report as a systematic review.	p1
**ABSTRACT**			
Abstract	2	See the PRISMA 2020 for Abstracts checklist.	attached
**INTRODUCTION**			
Rationale	3	Describe the rationale for the review in the context of existing knowledge.	p4-5
Objectives	4	Provide an explicit statement of the objective(s) or question(s) the review addresses.	lines 94-98
**METHODS**			
Eligibility criteria	5	Specify the inclusion and exclusion criteria for the review and how studies were grouped for the syntheses.	p5-7
Information sources	6	Specify all databases, registers, websites, organisations, reference lists and other sources searched or consulted to identify studies. Specify the date when each source was last searched or consulted.	p5-7 and attached search strategy
Search strategy	7	Present the full search strategies for all databases, registers and websites, including any filters and limits used.	search strategy
Selection process	8	Specify the methods used to decide whether a study met the inclusion criteria of the review, including how many reviewers screened each record and each report retrieved, whether they worked independently, and if applicable, details of automation tools used in the process.	p5-7
Data collection process	9	Specify the methods used to collect data from reports, including how many reviewers collected data from each report, whether they worked independently, any processes for obtaining or confirming data from study investigators, and if applicable, details of automation tools used in the process.	p6-7
Data items	10a	List and define all outcomes for which data were sought. Specify whether all results that were compatible with each outcome domain in each study were sought (e.g. for all measures, time points, analyses), and if not, the methods used to decide which results to collect.	p5-6
	10b	List and define all other variables for which data were sought (e.g. participant and intervention characteristics, funding sources). Describe any assumptions made about any missing or unclear information.	p5-6
Study risk of bias assessment	11	Specify the methods used to assess risk of bias in the included studies, including details of the tool(s) used, how many reviewers assessed each study and whether they worked independently, and if applicable, details of automation tools used in the process.	p6-7
Effect measures	12	Specify for each outcome the effect measure(s) (e.g. risk ratio, mean difference) used in the synthesis or presentation of results.	p6-7
Synthesis methods	13a	Describe the processes used to decide which studies were eligible for each synthesis (e.g. tabulating the study intervention characteristics and comparing against the planned groups for each synthesis (item #5)).	p6-7
	13b	Describe any methods required to prepare the data for presentation or synthesis, such as handling of missing summary statistics, or data conversions.	n/a
	13c	Describe any methods used to tabulate or visually display results of individual studies and syntheses.	p7
	13d	Describe any methods used to synthesize results and provide a rationale for the choice(s). If meta-analysis was performed, describe the model(s), method(s) to identify the presence and extent of statistical heterogeneity, and software package(s) used.	p6-7
	13e	Describe any methods used to explore possible causes of heterogeneity among study results (e.g. subgroup analysis, meta-regression).	p7
	13f	Describe any sensitivity analyses conducted to assess robustness of the synthesized results.	n/a
Reporting bias	14	Describe any methods used to assess risk of bias due to missing results in a synthesis (arising from reporting biases).	n/a

For peer review only - http://bmjopen.bmj.com/site/about/guidelines.xhtml

PRISMA 2020 Checklist

Section and Topic	Item #	Checklist item	Location where item is reported
assessment			
Certainty assessment	15	Describe any methods used to assess certainty (or confidence) in the body of evidence for an outcome.	n/a
**RESULTS**			
Study selection	16a	Describe the results of the search and selection process, from the number of records identified in the search to the number of studies included in the review, ideally using a flow diagram.	prisma diagram
	16b	Cite studies that might appear to meet the inclusion criteria, but which were excluded, and explain why they were excluded.	n/a
Study characteristics	17	Cite each included study and present its characteristics.	table 2
Risk of bias in studies	18	Present assessments of risk of bias for each included study.	table 2
Results of individual studies	19	For all outcomes, present, for each study: (a) summary statistics for each group (where appropriate) and (b) an effect estimate and its precision (e.g. confidence/credible interval), ideally using structured tables or plots.	table 1
Results of syntheses	20a	For each synthesis, briefly summarise the characteristics and risk of bias among contributing studies.	table 2 and p8-9
	20b	Present results of all statistical syntheses conducted. If meta-analysis was done, present for each the summary estimate and its precision (e.g. confidence/credible interval) and measures of statistical heterogeneity. If comparing groups, describe the direction of the effect.	table 1 and p8-9
	20c	Present results of all investigations of possible causes of heterogeneity among study results.	p9-10
	20d	Present results of all sensitivity analyses conducted to assess the robustness of the synthesized results.	n/a
Reporting biases	21	Present assessments of risk of bias due to missing results (arising from reporting biases) for each synthesis assessed.	p9-10
Certainty of evidence	22	Present assessments of certainty (or confidence) in the body of evidence for each outcome assessed.	p8-23
**DISCUSSION**			
Discussion	23a	Provide a general interpretation of the results in the context of other evidence.	p23-24
	23b	Discuss any limitations of the evidence included in the review.	p24-25
	23c	Discuss any limitations of the review processes used.	p25-26
	23d	Discuss implications of the results for practice, policy, and future research.	p26
**OTHER INFORMATION**			
Registration and protocol	24a	Provide registration information for the review, including register name and registration number, or state that the review was not registered.	p1
	24b	Indicate where the review protocol can be accessed, or state that a protocol was not prepared.	p1
	24c	Describe and explain any amendments to information provided at registration or in the protocol.	n/a
Support	25	Describe sources of financial or non-financial support for the review, and the role of the funders or sponsors in the review.	p1
Competing interests	26	Declare any competing interests of review authors.	p1
Availability of data, code and	27	Report which of the following are publicly available and where they can be found: template data collection forms; data extracted from included studies; data used for all analyses; analytic code; any other materials used in the review.	p1

For peer review only

PRISMA 2020 Checklist

Section and Topic	Item #	Checklist item	Location where item is reported
other materials			

From: Page MJ, McKenzie JE, Bossuyt PM, Boutron I, Hoffmann TC, Mulrow CD, et al. The PRISMA 2020 statement: an updated guideline for reporting systematic reviews. BMJ 2021;372:n71. doi: 10.1136/bmj.n71

For more information, visit: http://www.prisma-statement.org/

For peer review only

BMJ Open

A systematic review of the relationship between burnout and spiritual health in doctors

Journal:	*BMJ Open*
Manuscript ID	bmjopen-2022-068402.R2
Article Type:	Original research
Date Submitted by the Author:	27-Apr-2023
Complete List of Authors:	Whitehead, Ishbel Orla; Newcastle University Population Health Sciences Institute, Biomedical Research Building<br>Moffatt, Suzanne; Newcastle University Population Health Sciences Institute<br>Warwick, Stephanie; Newcastle University Population Health Sciences Institute, Biomedical Research Building<br>Spiers, Gemma; Newcastle University, Population & Health Sciences Institute<br>Kunonga, Tafadzwa; Newcastle University Population Health Sciences Institute<br>Tang, Eugene; Newcastle University Faculty of Medical Sciences<br>Hanratty, Barbara; Newcastle University Population Health Sciences Institute
**Primary Subject Heading**:	General practice / Family practice
Secondary Subject Heading:	Occupational and environmental medicine, Public health
Keywords:	Human resource management < HEALTH SERVICES ADMINISTRATION & MANAGEMENT, OCCUPATIONAL & INDUSTRIAL MEDICINE, PUBLIC HEALTH, Primary Care < Primary Health Care, Primary Health Care

SCHOLARONE™
Manuscripts

I, the Submitting Author has the right to grant and does grant on behalf of all authors of the Work (as defined in the below author licence), an exclusive licence and/or a non-exclusive licence for contributions from authors who are: i) UK Crown employees; ii) where BMJ has agreed a CC-BY licence shall apply, and/or iii) in accordance with the terms applicable for US Federal Government officers or employees acting as part of their official duties; on a worldwide, perpetual, irrevocable, royalty-free basis to BMJ Publishing Group Ltd ("BMJ") its licensees and where the relevant Journal is co-owned by BMJ to the co-owners of the Journal, to publish the Work in this journal and any other BMJ products and to exploit all rights, as set out in our licence.

The Submitting Author accepts and understands that any supply made under these terms is made by BMJ to the Submitting Author unless you are acting as an employee on behalf of your employer or a postgraduate student of an affiliated institution which is paying any applicable article publishing charge ("APC") for Open Access articles. Where the Submitting Author wishes to make the Work available on an Open Access basis (and intends to pay the relevant APC), the terms of reuse of such Open Access shall be governed by a Creative Commons licence – details of these licences and which Creative Commons licence will apply to this Work are set out in our licence referred to above.

Other than as permitted in any relevant BMJ Author's Self Archiving Policies, I confirm this Work has not been accepted for publication elsewhere, is not being considered for publication elsewhere and does not duplicate material already published. I confirm all authors consent to publication of this Work and authorise the granting of this licence.

For peer review only

A systematic review of the relationship between burnout and spiritual health in doctors

Short title: Doctors' burnout and spiritual health- a systematic review

Ishbel Orla Whitehead[a], Suzanne Moffatt[b], Stephanie Warwick[a], Gemma Frances Spiers[a], Tafadzwa Patience Kunonga[a], Eugene Yee Hing Tang[a], Barbara Hanratty[a]

[a]Population Health Sciences Institute, Faculty of Medical Sciences, Newcastle University, Biomedical Research Building, Campus for Ageing and Vitality, Newcastle upon Tyne, NE4 5PL

[b] Population and Health Sciences Institute, Newcastle University, Baddiley-Clark, Newcastle upon Tyne, NE2 4AX

Corresponding author: Orla Whitehead. Email: orla.whitehead@newcastle.ac.uk orcid ID- 0000-0002-4171-8583Declarations

Competing Interests: None declared.

Registration of review: Prospero- CRD42020200145

https://www.crd.york.ac.uk/prospero/display_record.php?RecordID=200145

Funding statement: OW is funded by the National Institute for Health Research (NIHR) on an in practice fellowship, grant number NIHR301074. The views expressed are those of the authors and not necessarily those of the NIHR or the Department of Health and Social Care. BH and SM are funded by the NIHR Applied Research Collaboration (ARC) North East and North Cumbria (grant number not applicable)

Ethics approval: Not required

Contributorship: Conception and design of study: IOW, SM, and BH. Search design and searching: IOW Screening records: IOW, SM, SW, GS, ET, TPK, BH. Data curation: IOW, BH. Analysis and interpretation of data: IOW, SW, GS. Visualisation and drafting the manuscript: OW, SM, GS, SW, CJ, BH Revising the manuscript critically for important intellectual content and authorizing for publication: OW, SM, CJ, BH, GS, PK, ET

Acknowledgements: Thank you to Catherine Richmond and Hannah O'Keefe for their information science input. Thank you to Carol Jagger for her input.

For peer review only

Data availability: All data relevant is included in the study itself, as a systematic review, the data used is in the studies referenced. Template collection forms, data extracted from included studies and used for all analyses are available on contacting the authors.

Abstract

Objective: To investigate the relationship between burnout and spiritual health amongst medical doctors

Design: Systematic literature review and narrative synthesis of cross -sectional studies

Setting: Any setting, worldwide

Data sources: Five databases were searched from inception to March 2022, including Medline, Embase, Psychinfo, Scopus and Web of Science

Eligibility Criteria: Any study design that involved Medical doctors (and other healthcare staff if assessed alongside medical doctors), that measured (in any way) both Burnout (or similar) and spiritual health (or similar) medical doctorsData Extraction and Synthesis: All records were double screened. Data extraction was performed by one reviewer and a proportion (10%) checked by a second reviewer. Quality was assessed using the Appraisal of Cross-sectional Studies tool (AXIS). Due to the heterogeneity of the included studies, a narrative review was undertaken without a meta-analysis.

Results: Searches yielded 1049 studies. 40 studies met eligibility criteria and were included in this review. Low reported levels of spirituality were associated with high burnout scores, and vice versa. Religion was not significantly associated with lower levels of burnout. Few studies reported statistically significant findings, few used validated spiritual scores, and most were vulnerable to sampling bias.

For peer review only

Conclusions: Published research suggests that burnout is linked to spiritual health in medical doctors but not to religion. Robust research is needed to confirm these findings and develop effective interventions.

Prospero registration CRD42020200145.

Strengths and limitations of this study

- A comprehensive, systematic approach was taken to searches, with expert information scientist input.
- Broad search terms were used to elicit all the available evidence, as there is inconsistency in headings used in different databases, cultures, and countries.
- A validated tool was used for quality assessment- the Appraisal of Cross-sectional Studies tool.
- The heterogeneity of measures used for burnout and spiritual health meant that meta-analysis was not possible.

Keywords

Burnout, spiritual health, wellbeing, spirituality, religion, compassion fatigue, moral injury

Definitions:

Spiritual Health- The authors use a definition of spiritual health developed by UK General Practitioners: self-actualisation and meaning, transcendence and relationships beyond the self, and expressions of spirituality. This term is used to reflect the broad and diverse experiences of medical doctors.

Burnout- The authors use a definition in use by the World Health Organisation of an occupational phenomenon resulting in exhaustion; distance, negativity or cynicism towards the job and reduced professional efficacy.

Introduction

For peer review only

Burnout is defined by the World Health Organisation as an occupational phenomenon, resulting in exhaustion, mental distance or feelings of negativity or cynicism towards the job, and reduced professional efficacy.(1) This has long been a concern for medical doctors, and the COVID-19 pandemic has increased attention on the health and wellbeing of health professionals.(2, 3) Medical doctors' descriptions of burnout include a loss of meaning in work, and objectification of patients and their families, rather than engaging with their humanity.[(4)] As health services globally are placed under increasing pressure, there is growing concern around physician burnout, moral injury and related concepts causing harm to the workforce.(5, 6) Burnout leads to retirement and resignation(7) which adds to the workforce crisis, as well as patient safety concerns.(8) The Covid-19 pandemic has presented huge challenges to the health care workforce.[(9, 10)] Burnout appears to have a malign effect on all aspects of health,(11, 12) as well as being impacted itself by all aspects of health.(13) The aeitology of burnout is still not fully understood, is likely multi-factorial, and merits further research.(14-16)

The biomedical model that shapes much modern medical practice, has so far been unable to offer a comprehensive and holistic understanding of the burnout phenomenon.(17-19) This, along with the framing of burnout as an occupational phenomenon rather than a mental illness,(1) suggests that a different lens may be needed to develop effective approaches to burnout . In a recent survey, general practitioners (GPs) defined spiritual health in ways that overlapped with current constructs of burnout.(12) Spiritual health was framed by medical doctors as a positive concept.(12) Spiritual health encompasses meaning, purpose, self-actualisation, transcendence and relationships with others beyond the self.(12) Spiritual or religious practices, including mindfulness and yoga, were also included in spiritual health definitions, and both are promoted as approaches to reduce burnout in the workplace.(20, 21) Burnout has been associated with moral injury,(22) referring to the harm caused when someone is required to act contrary to their internal ethical code.(23) Being true to a personal or religious ethical code, was also an aspect of spiritual health reported by GPs.(12) Some authors argue that religiosity and spirituality are one and the same(24), whereas English GPs defined spiritual health as including, but not exclusive to, religion,(12) and there is a growth in the population that consider themselves 'spiritual but not religious.'(25) The concepts of spiritual health and burnout have been found to overlap in other groups of professionals.(26-28) This raises the possibility that promoting spiritual health may offer an effective approach

For peer review only

to prevention and mitigation of burnout for medical doctors, with opportunities for intervention at an individual and organizational level.

This systematic review asks whether there is a measured association between burnout and spiritual health amongst medical doctors. It will consider the quantity and quality of quantitative evidence on burnout, spiritual health, and related concepts including spiritual wellbeing and distress, and religiosity by comparing all studies that have performed a measure of burnout and a measure of spiritual health, both in the broadest terms, in qualified medical doctors worldwide.

Methods

Searches and selection of studies

Studies to be included in the review were identified by searching the following databases: (1) EMBASE (1974–March 2022), (2) PsycINFO (1806– March 2022), (3) Ovid MEDLINE (1946–March 2022), (4) Web of Science and (5) Scopus. The last search was performed on the 8th March 2022. The search strategy (supplementary file 1) was developed in Embase and adapted for use in other databases by an information scientist. For comprehensiveness both MESH headings and key terms were used. All searches were completely re-run in March 2022. A Cochrane filter(29) and term 'not editorial or letter' was added to filter out non-studies and opinion pieces. Citations from the studies found were searched for previous similar studies. The searches were peer reviewed by an information scientist at Newcastle University.

Eligible studies were those reporting quantified evidence about both burnout (or similar concepts including occupational stress, moral injury from work, occupational compassion fatigue etc.) and spiritual health (or concepts that included this aspect of health). These data must be reported in a study population that included medical doctors from any speciality or in post-graduate training. Any study design was eligible, but commentaries and editorials were excluded. The WHO definition for burnout[1] and the Whitehead et al[2] definitions were referred to and considered by two reviewers at screening stage.There were no language restrictions to the searches.

Population: Medical doctors (post-graduate registered medical professionals from any speciality)

For peer review only

Exposure: Spiritual health, spiritual wellness, spiritual wellbeing, spirituality, religiosity or similar concepts or sub-phenomena, as defined by doctors.(12)

Outcome: Burnout, moral injury, job satisfaction, compassion fatigue, work stress, or similar concepts that could come within the WHO definition for burnout.(1)

Study Design: Any study with quantification of the interventions and outcomes of interest.

2.

Search terms were deliberately comprehensive, and related or similar terms to burnout or spiritual health were included in the searches, for example compassion fatigue, moral injury, mindfulness etc. Studies were included if we felt that the study measured any phenomenon very close to occupational burnout, **and** any measure of any aspect of wellbeing close to spiritual health.

Data extraction and synthesis

All records were double screened at title and abstract, with disagreements resolved by discussion with a third reviewer, or taken to full text screening. De-duplication was done in Endnote X9.(30) Rayyan software was used to manage records.(31) Attempts were made to retrieve all articles, with requests to authors. Emails to authors suggested anecdotally that there could be publication and availability bias, and therefore unpublished studies, found via conference abstracts, were included, and authors were contacted for more information where limited data were presented. No other grey literature was included. Data extraction was performed by one reviewer and a proportion (10%) checked by a second reviewer. Study quality was assessed using the Appraisal of Cross-sectional Studies tool (AXIS).(32) While this tool does not give a 'numerical score', it assessed the methodology and internal consistency of the studies, as well as risk of bias, and as such allows assessment of the merits of each criterion as applied to that study. Heterogeneity in study design, scales, and methods of analysis meant that meta-analysis was not possible, and a narrative synthesis was conducted. Subgroups were considered to see if this gave a greater sense of direction of the evidence. If statistics, for example odds ratios, or relative risks, were provided, these have been summarised within the narrative analysis. Custom made tables were used to analyse the assessments used for burnout and spiritual health, and synthesised the data, including common biases.

For peer review only

The review was registered on Prospero, available from: https://www.crd.york.ac.uk/prospero/display_record.php?ID=CRD42020200145 and the protocol can be found in supplementary file 2.

Patient and public Involvement

This systematic review was prompted by patient and public involvement in a previous study looking at spiritual health in primary care. The public felt that the health of medical doctors, and burnout, was a priority for this topic.

Results

Forty studies met the inclusion criteria, from screening of the 1049 records identified in the electronic searches (Fig 1). All included studies provided an assessment of burnout and spiritual health (or related concepts) but there was a high level of heterogeneity in the measures used.

FIG 1 HERE

The number of study participants ranged from 4501 in the largest study(33) to 7 in the smallest.(34) Twenty studies included other healthcare staff in addition to the medical staff participants.(34-53) Four studies included primary care physicians,(42, 54-56) with most studies involving medical doctors from intensive or critical care, emergency departments and internal medicine.(33-36, 39, 47, 48, 50, 51, 54, 56-66)(Figure 2) No studies were identified including medical doctors in psychiatry or public health. Data from medical doctors were not always analysed separately.(35, 36, 38, 39, 44, 49, 51, 52)

The oldest study was also the largest, published in 1999 (33). Three studies were conducted during the COVID-19 pandemic (51, 52, 59). A USA study showing lower burnout scores in those who had a faith (including 'spiritual but not religious') (51), a Singapore study with participants who preferred religious coping to thanks from the public (52) and a Portuguese study which showed no association between burnout scores and religiosity in

For peer review only

For peer review only - http://bmjopen.bmj.com/site/about/guidelines.xhtml

medical doctors (59). Five of the forty studies reported findings from surveys in 2020 onwards.(51, 52, 57, 59, 67)

FIGURE 2 HERE

FIGURE 3 HERE

Almost half of the studies (19) were from the USA (33, 37, 38, 44, 47, 51, 53, 54, 60-62, 64-66, 68-72) reflecting the dominance of North American researchers in this area (Figure 3). Twelve studies included participants from Europe.(36, 39, 43, 45, 49, 50, 55, 57-59, 63, 73) The largest Asian study recruited widely, pooling data from diverse countries in wealth, religion and culture, such as Saudi Arabia, Laos and Bangladesh.(48)

Study Quality

The majority of studies were assessed to be poor quality, with risks of selection bias, response bias, availability bias, and bias. One study, Roslan et al,(56) met all the AXIS criteria fully, including justification of sample size, and there were seven studies which were of otherwise high quality as assessed by the AXIS tool (meeting 18-19 of the 20 criteria).(40-42, 44, 46, 62, 69) Three studies were not yet published in full, and therefore not had peer review.(38, 53, 55) Authors reported difficulty publishing their findings.(38, 74) This, as well as other studies where data was gathered on burnout and spiritual health, but not all comparison data was published,(35, 45, 52, 55, 56, 68) may have introduced availability bias. These were included as it was suspected this topic may be affected by publication bias. Most studies did not use a validated measure of spiritual health, with many studies using non-validated assessments of burnout.

Relationship between burnout and spiritual health in medical doctors

These heterogenous studies drew a wide range of conclusions (Supplementary Table 1). Seventeen studies found an association between spiritual health or related concepts and lower levels of burnout or related concepts,(37,

For peer review only

39, 40, 42-45, 48, 51, 53, 55, 56, 58, 66, 71, 75, 76) seventeen studies found no statistically significant association,(33-35, 38, 41, 47, 49, 50, 54, 59-61, 63-65, 73, 77) and one study found higher compassion fatigue in those using spiritual type behaviours.(62) Across the eight studies rated high in quality, evidence remained inconsistent. There was evidence of: an inverse association between religiosity and burnout; (42, 44) no association between spiritual concepts and burnout scores;(63, 69) an association between prayer and meditation and higher compassion fatigue; (62) an association between irregular spirituality routines and burnout; (56) a link between no spirituality and low personal accomplishment;(40) and an association between spirituality and high emotional exhaustion on univariate analysis but not on multivariate analysis.(46) One study found an association between personal accomplishment and burnout, on exclusion of respondents who omitted the spirituality question.(41) Where statistically significant associations between spiritual health (or similar), or religiosity, and burnout (or similar) were found, the effects were typically small.

The removal of studies rated low in quality (score 12 or under on AXIS criteria) does not enhance consistency the findings. Further, there does not appear to be a pattern in terms of whether an association is shown based on country, whether that country has a state religion, but this assessment is limited by the predominance of the USA in these studies. The three most recent studies from the COVID pandemic(51, 52, 59) do not clarify the direction of evidence, with one study finding an association,(51) one did in nurses but not medical doctors,(59) and one where participants agreed they used religious coping, but did not compare religious coping and burnout scores.(52)

Studies which looked at wider spiritual health only, rather than asking about religion, were more likely to find an association between burnout and spiritual health. Seven studies of those which asked or measure spirituality and not religion found an association,(37, 39, 40, 43, 53, 58, 71) and three studies did not.(36, 61, 65) Religion, or religious coping, alone does not appear to have a consistent effect on burnout rates, however, wider spiritual health may have some effect.

Please see Supplementary Table 1: Findings from each study (supplementary materials)

For peer review only

Please see Supplementary Table 2: Study details, AXIS quality assessment, spiritual wellness assessment and burnout score used (supplementary materials)

Quantification of burnout

Twenty one studies(34, 35, 39-41, 47, 48, 50, 51, 53, 54, 57, 58, 61, 63, 66, 68, 69, 71, 73, 76) used the complete Maslach Burnout Inventory (MBI) (78) (Supplementary Table 1). One study used a 2-item 'MBI' (64) in an emergency department population, that has been found to correlate with the full MBI in a similar population.(79) Two studies used the Oldenburg Inventory,(45, 59) a measure similar to MBI, but more applicable to people not directly involved in patient care. It also measures engagement with work, not including professional accomplishment. (80) Three more recent studies (52, 56, 65) used the Copenhagen Burnout Inventory (CBI),(81) a free to use (MBI is commercially available) measure of personal, work and patient related burnout, which identifies a similar burned out group to the MBI. (82) Other tools used included the perceived stress scale (PSS), (42, 75) Cool Down Index (36) and Work Related Strain Inventory (WRSI). (70) Whilst these do not measure burnout directly, they have been found to correlate with burnout as measured by the MBI.(36, 70, 83, 84) The Professional quality of life (proQOLl) scale used by two studies (55, 60) measures compassion fatigue, which is considered to be a phenomenon including burnout by the ProQOL authors. (85) One study (62) used the Compassion Fatigue Test for Helpers. Clark et al (37) used an 8 item job satisfaction scale, which included some domains that could be considered as spiritual, for example the sense of calling, meaning and purpose in life and being of worth. (37) Frank et al(33) also measured job satisfaction and desire to become a physician again. Shetach et al(49) also used a job satisfaction questionnaire, translated into Hebrew, designed for an unpublished thesis in marketing, and not validated with healthcare staff.

Assessing spiritual health, wellbeing, spirituality and/or religiosity

An array of methods were used to assess spiritual health or similar concepts (Supplementary Table 2). Validated tools include the Functional Assessment of Chronic Illness Therapy- Spiritual Well-Being Scale , a modified

For peer review only

version for non-illness (FACIT-Sp-Non-Illness);(86) the Jarel spiritual wellbeing scale,(87) and Hatch spiritual involvement and belief scale.(88) Büssing et al(36) used the Aspects of Spirituality questionnaire, developed by the authors in a niche setting of a particular philosophical institution.(89) The Fetzer Institute Multidimensional Measurement of Religiousness/Spirituality for Use in Health Research (90) was also used to assess religious practice, along with general, unvalidated, questions about religious commitment, and spiritualty.(64) A systematic review identified the FACIT and the Spirituality Index of Wellbeing (91) as being the highest most well validated instruments,(92) however only one unpublished study (53) used the former, and none used the latter. Many studies used general coping scores, such as Brief COPE (93) and the Hobfoll's Questionnaire,(94) general quality of life scores or including spiritual practice in general questions, which have aspects of spiritual health as part of a wider assessment. Some studies used single questions, e.g. "my job gives me meaning",(85) "my religious and spiritual beliefs strongly influence my work and patients",(41) "Spirituality/religion is important in my life"(66) or "do you consider yourself spiritual".(40, 71) Seventeen studies which asked about religion only (Supplementary Table 2). The Duke University religion index (DUREL) (95) measures organisational religiosity, measuring religious practice, and was used alongside Hoge's Intrinsic Religiosity Scale by Ramondetta et al.(70) The difference between spirituality and religiosity is not always delineated by the authors: Frank et al used religiosity data asking how 'strongly' participants were religious, and then argue that physicians could be encouraged to explore spirituality to increase satisfaction,(33) however spirituality is not addressed in the data, and Macuka et al discussed religiosity, but their question appeared to ask more generally about spirituality.(45) Ramondetta et al asked participants whether they were religious, spiritual, both or neither, but only quantified religiosity.(70)

Discussion

Key findings

There is increasing attention on both burnout and on the relationship between medical doctors' burnout and their spiritual health. This has become even more critical in the aftermath of the COVID-19 pandemic. Most of the research in this review reported positive associations between higher levels of spiritual health or related concepts, and lower levels of burnout or related phenomena. A number of studies found no association, with

For peer review only

two describing greater distress with higher levels of religiosity,(57, 62) perhaps due to use of religious coping as a negative strategy.(96) Similar to other reviews in this area, research from the USA is dominant, with few studies from more secular areas. (The USA has high levels of religiosity(97, 98) than, for example, many northern European countries.(99)) The one high quality study on the topic stated that spirituality routines lower the risk of burnout,(56) as they found that regular spiritual routines were associated with lower burnout risk, however, we cannot infer causation from the study design. Few studies reported statistically significant findings and the marked study heterogeneity did not support metanalysis. While this review suggests that there is probably a link between spiritual health and burnout in medical doctors, the quality of the evidence is poor, and high quality robust quantitative studies of the relationship between spiritual health and burnout in medical doctors is needed.

Comparison with other work

To our knowledge, this is the most comprehensive review of burnout and spiritual health in medical doctors to date. This review offers a different perspective from other work in this area that utilise a biomedical approach to burnout rather than a holistic approach to include spiritual health.(100-102) Williams et al's systematic review of the consequences of physician burnout considered the physical and mental health implications of burnout in their review, but neglected to mention spiritual health or distress.(101) Chow et al's scoping review of religion and spirituality in residents included discussion of the link between reduced burnout and spirituality. (103) Sibeoni et al's systematic review and meta synthesis of physician's views of burnout includes aspects of spiritual health in 'calmness or letting go', but did not analyse spiritual health as an aspect of overall health. (104) These authors reported that individual factors (including spiritual) and relational factors were more important than organizational concerns in protecting medical doctors from burnout.(104) Current emphasis is on the role of COVID-19 in medical doctors' burnout.(105) with Jefferson et al finding studies considered gender and age effects on burnout during COVID (10) but spiritual health is not mentioned. While this review is limited by the quality of the studies included, there does appear to be merit in the hypothesis of looking wider than the biomedical model.

Common study limitations

For peer review only

There were several limitations common to the studies in this review: response bias, social desirability bias, sampling bias, survivorship/selection bias, and cultural or language bias. There also appears to be availability and publication bias, with the topic of spiritual health being controversial- with data on spiritual health not reported in the published paper, despite being collected. (55, 57) The majority of studies were vulnerable to non-response and social desirability bias. Response rates were highly variable, range 5-99%. Use of gatekeepers, such as heads of departments(85) or directors(48, 50, 71) could cause sampling bias, as could use of convenience sampling at conferences.(57, 60, 66) Non-responders were seldom (eight studies) described. (33, 36, 40, 44, 56, 62, 67, 106) Pressures from the COVID pandemic may have affected studies carried out during this period.(51, 52, 59) Social desirability bias was possible due to staff feeling unable to respond honestly in very religious, or very secular environments, or share the true depth of their burnout symptoms(40). Studies that took place in the work setting were vulnerable to survivorship/selection bias.(38-42, 46, 66) Purvis et al excluded residents, as they were not permanent staff.(47)

Just under half (n=19) of the studies used the Maslach Burnout Inventory (MBI) (78) a measure of burnout often considered the 'gold standard'(107) burnout inventory, Often, studies presented data on the influence of factors such as spiritual health on burnout, when one domain only was affected.(40, 41, 61) The use of other inventories were not justified as to whether this was because these were better tools in their context, because they are free to use, or another reason.

Despite there being multiple available scales of religiosity or spiritual health and wellbeing,(92, 108, 109) few studies used a validated tool to assess personal spiritual health or related concepts. With the growth of the "spiritual, but not religious"(51) and secular societies,(110) there is merit in using validated tool to assess current spiritual health state.(92) The single questions about spiritual wellbeing or similar were untested and unvalidated, and therefore may have introduced information bias to the studies, and limit the utility of the data.

The use of measures in English with populations whose first language is not English may affect the quality of results. In some studies, measures were translated into the native language.(34-36, 39, 45, 49, 76, 85) However in other studies, English measures were used, without regard to the language and culture of participants,(40, 48, 57, 75) or without stating which language/translation was used.

For peer review only - http://bmjopen.bmj.com/site/about/guidelines.xhtml

Strengths and Limitations of this review

This review is broad, and novel, including a wide definition of aspects of spiritual health. The lack of consistency in indexing for spiritual health and religiosity across electronic database (Medical Subject Headings) meant development of comprehensive searches was a challenge. However, the diverse range and number of papers screened suggests that the searches were sufficiently broad. Only observational, cross-sectional studies, were found, and therefore we were unable to analyse whether there is any causal relationship between spiritual health and burnout. While most studies are from the USA, the other studies are diverse in country of setting, and cover multiple different cultures and faiths. An objective assessment was made of study quality, using the AXIS tool. Unfortunately, heterogeneity of the measures and analysis used in the studies has meant meta analysis was not possible. Some published abstracts were unobtainable, and publication bias likely affects this review, although the direction of this effect is unknown. This was mitigated by the inclusion of unpublished data on the topic.(38, 53, 55)

Future research

While there have been studies that have identified this topic as being of interest, these have rarely used validated measures of both variables- burnout and spiritual health. Research utilising accepted and validated tools to measure both spiritual health and burnout are needed, especially in Northern Europe, where research is limited. Future works should also strive to recruit study populations that are not limited to people with specific interest in the topic or strong religious views. Identification, or refutation, of a link between spiritual health and burnout will allow fresh assessment of current organizational and individual interventions for burnout. Developing and evaluating interventions that improve medical doctors' spiritual health may mitigate against burnout.

Conclusions

A link between spiritual health and burnout in medical doctors has been hypothesised by multiple studies, and interest in the link appears to be growing. However, studies investigating this have been vulnerable to many methodological flaws. Despite this, and limited evidence for efficacy,(111) practices rooted in spiritual practice, such as mindfulness, meditation, and yoga, are often recommended to prevent burnout.(47, 112, 113) Since

For peer review only

there does not appear to be a relationship between the narrower concept of religiosity and burnout, research into any association between broader spiritual health and burnout, using validated tools, is needed to examine the hypothesis that spiritual health and burnout are associated concepts, and to prompt research into interventions that could prevent burnout in medical doctors.

References

1. World Heath Organisation. Burn-out an "occupational phenomenon": International Classification of Diseases 2019 [Available from: https://www.who.int/mental_health/evidence/burn-out/en/.
2. Locke T. Medscape UK Doctors' Burnout & Lifestyle Survey 2020 2020 [Available from: https://www.medscape.com/slideshow/uk-doctors-burnout-2020-6013312?src=mkm_ret_11012020_mscpmrk_uk_burnout_int&faf=1.
3. Lemaire JB, Wallace JE. Burnout among doctors. BMJ. 2017;358:j3360.
4. Fred HL, Scheid MS. Physician Burnout: Causes, Consequences, and (?) Cures. Tex Heart Inst J. 2018;45(4):198-202.
5. Sundararaman L, Chow SY, Elakkumanan LB, Rajagopalan V, Verma A. Physician Burnout: A Pandemic Uncovered by a Pandemic! ASA Monitor. 2022;86(1):19-20.
6. Melnikow J, Padovani A, Miller M. Frontline physician burnout during the COVID-19 pandemic: national survey findings. BMC Health Services Research. 2022;22(1):365.
7. Sheather J, Slattery D. The great resignation-how do we support and retain staff already stretched to their limit? Bmj. 2021;375:n2533.
8. Garcia CdL, Abreu LCd, Ramos JLS, Castro CFDd, Smiderle FRN, Santos JAd, et al. Influence of Burnout on Patient Safety: Systematic Review and Meta-Analysis. Medicina. 2019;55(9):553.
9. Chor WPD, Ng WM, Cheng L, Situ W, Chong JW, Ng LYA, et al. Burnout amongst emergency healthcare workers during the COVID-19 pandemic: A multi-center study. Am J Emerg Med. 2021;46:700-2.
10. Jefferson L, Golder S, Heathcote C, Avila AC, Dale V, Essex H, et al. GP wellbeing during the COVID-19 pandemic: a systematic review. British Journal of General Practice. 2022;72(718):e325.
11. Salvagioni DAJ, Melanda FN, Mesas AE, González AD, Gabani FL, Andrade SM. Physical, psychological and occupational consequences of job burnout: A systematic review of prospective studies. PLoS One. 2017;12(10):e0185781.
12. Whitehead O, Jagger C, Hanratty B. What do doctors understand by spiritual health? A survey of UK general practitioners. BMJ Open. 2021;11(8):e045110.
13. Diez-Pinol M, Dolan S, Sierra V, Cannings K. Personal and organizational determinants of well-being at work: The case of Swedish physicians. International Journal of Health Care Quality Assurance. 2008.
14. Weber A, Jaekel-Reinhard A. Burnout syndrome: a disease of modern societies? Occupational medicine. 2000;50(7).
15. Boisaubin EV. Causes and treatment of impairment and burnout in physicians: The epidemic within. Faculty health in academic medicine. 2009:29.
16. Kumar S, Hatcher S, Huggard P. Burnout in Psychiatrists: An Etiological Model. International journal of psychiatry in medicine. 2005;35(4):405-16.
17. Engebretsen KM, Bjorbækmo WS. Naked in the eyes of the public: A phenomenological study of the lived experience of suffering from burnout while waiting for recognition to be ill. Journal of Evaluation in Clinical Practice. 2019;25(6):1017-26.
18. Engebretsen KM. Suffering without a medical diagnosis. A critical view on the biomedical attitudes towards persons suffering from burnout and the implications for medical care. Journal of Evaluation in Clinical Practice. 2018;24(5):1150-7.
19. Heinemann LV, Heinemann T. Burnout Research: Emergence and Scientific Investigation of a Contested Diagnosis. SAGE Open. 2017;7(1):2158244017697154.
20. Goodman MJ, Schorling JB. A Mindfulness Course Decreases Burnout and Improves Well-Being among Healthcare Providers. The International Journal of Psychiatry in Medicine. 2012;43(2):119-28.

For peer review only

21. Ireland MJ, Clough B, Gill K, Langan F, O'Connor A, Spencer L. A randomized controlled trial of mindfulness to reduce stress and burnout among intern medical practitioners. Medical Teacher. 2017;39(4):409-14.

22. Dean W, Talbot S, Dean A. Reframing Clinician Distress: Moral Injury Not Burnout. Fed Pract. 2019;36(9):400-2.

23. Talbot SG, Dean W. Physicians aren't 'burning out.'They're suffering from moral injury. Stat. 2018.

24. Koenig HG. Religion, Spirituality, and Medicine: Application to Clinical Practice. JAMA. 2000;284(13):1708-.

25. Lee L. Secular or nonreligious? Investigating and interpreting generic 'not religious' categories and populations. Religion. 2014;44(3):466-82.

26. Hardiman P, Simmonds JG. Spiritual well-being, burnout and trauma in counsellors and psychotherapists. Mental Health, Religion & Culture. 2013;16(10):1044-55.

27. Wachholtz A, Rogoff M. The relationship between spirituality and burnout among medical students. J Contemp Med Educ. 2013;1(2):83-91.

28. Lizano EL, Godoy AJ, Allen N. Spirituality and worker well-being: Examining the relationship between spirituality, job burnout, and work engagement. Journal of Religion & Spirituality in Social Work: Social Thought. 2019;38(2):197-216.

29. Cochrane library. Search strategies to identify observational studies in MEDLINE and Embase [Available from: https://www.cochranelibrary.com/cdsr/doi/10.1002/14651858.MR000041.pub2/appendices.

30. The EndNote Team. Endnote. Endnote X9 ed. Philadelphia, PA2013.

31. Ouzzani M, Hammady H, Fedorowicz Z, Elmagarmid A. Rayyan—a web and mobile app for systematic reviews. Systematic Reviews. 2016;5(1):210.

32. Downes MJ, Brennan ML, Williams HC, Dean RS. Development of a critical appraisal tool to assess the quality of cross-sectional studies (AXIS). BMJ Open. 2016;6(12):e011458.

33. Frank E, McMurray J, Linzer M, Elon L. Career satisfaction of US women physicians - Results from the Women Physicians' Health Study. ARCHIVES OF INTERNAL MEDICINE. 1999;159(13):1417-26.

34. Das S, Barman S, Datta S, Bardhan N, Baishya M, Das B, et al. Degree of burnout among emergency healthcare workers and factors influencing level of burnout: a pilot study. Delhi Psychiatry Journal. 2016;19:36-47.

35. Baruah A, Das S, Dutta A, Das B, Sharma T, Hazarika M. DEGREE AND FACTORS OF BURNOUT AMONG EMERGENCY HEALTHCARE WORKERS IN INDIA. Int J Sci Res (Ahmedabad). 2019;8(4):41-5.

36. Büssing A, Loetzke D, Gloeckler M, Heusser P. Influence of Spirituality on Cool Down Reactions, Work Engagement, and Life Satisfaction in Anthroposophic Health Care Professionals. Evidence-Based Complementary and Alternative Medicine. 2015.

37. Clark L, Leedy S, Mcdonald L, Muller B, Lamb C, Mendez T, et al. Spirituality and job satisfaction among hospice interdisciplinary team members. JOURNAL OF PALLIATIVE MEDICINE. 2007;10(6):1321-8.

38. Guay MD, Reyes Donoso MM, Lopez Saca JM, Riveros Rios ME, Pastrana T. Spirituality and Religiosity and Burnout in Latin-American Palliative Care Health Care Professionals (LAPC) (S818). Journal of Pain and Symptom Management. 2019;57(2):490-1.

39. Glebocka A, Lisowska E, Glebocka A, Lisowska E. Professional burnout and stress among polish physicians explained by the Hobfoll resources theory. JOURNAL OF PHYSIOLOGY AND PHARMACOLOGY. 2007;58:243-52.

40. Koh MYH, Chong PH, Neo PSH, Ong YJ, Yong WC, Ong WY, et al. Burnout, psychological morbidity and use of coping mechanisms among palliative care practitioners: A multi-centre cross-sectional study. Palliative Medicine. 2015;29(7):633-42.

41. Lal A, Tharyan A, Tharyan P. The prevalence, determinants and the role of empathy and religious or spiritual beliefs on job stress, job satisfaction, coping, burnout, and mental health in medical and surgical faculty of a teaching hospital: A cross-sectional survey. Revue de Medecine Interne. 2020;41(4):232-40.

42. Leonelli LB, Andreoni S, Martins P, Kozasa EH, Salvo VLd, Sopezki D, et al. Perceived stress among Primary Health Care Professionals in Brazil. Estresse percebido em profissionais da Estrategia Saude da Familia. 2017;20(2):286-98.

43. Macuka I, Tucak Junakovic I. A Cross-Sectional Study of Job Satisfaction and Intention to Leave Job in Palliative Care in Croatia. Journal of palliative care. 2021:8258597211046704.

44. Mantri S, Lawson JM, Wang ZZ, Koenig HG. Prevalence and Predictors of Moral Injury Symptoms in Health Care Professionals. JOURNAL OF NERVOUS AND MENTAL DISEASE. 2021;209(3):174-80.

45. Macuka I, Junakovic I, Bozic D, Macuka I, Junakovic IT, Bozic D. Burnout among Palliative Care Professionals. DRUSTVENA ISTRAZIVANJA. 2020;29(2):287-308.

46. Ntantana A, Matamis D, Savvidou S, Giannakou M, Gouva M, Nakos G, et al. Burnout and job satisfaction of intensive care personnel and the relationship with personality and religious traits: An observational, multicenter, cross-sectional study. Intensive and Critical Care Nursing. 2017;41:11-7.

For peer review only

47. Purvis T, Saylor D, Powell B, Biba G, Conti D, Crowe T, et al. Burnout and Resilience Among Neurosciences Critical Care Unit Staff. NEUROCRITICAL CARE. 2019;31(2):406-10.
48. See K, Zhao M, Nakataki E, Chittawatanarat K, Fang W, Faruq M, et al. Professional burnout among physicians and nurses in Asian intensive care units: a multinational survey. INTENSIVE CARE MEDICINE. 2018;44(12):2079-90.
49. Shetach A, Marcus O, Shetach A, Marcus O. The critical managerial capabilities of medical and nursing managers in an Israeli hospital. EVIDENCE-BASED HRM-A GLOBAL FORUM FOR EMPIRICAL SCHOLARSHIP. 2015;3(1):81-102.
50. Teixeira C, Ribeiro O, Fonseca AM, Carvalho AS. Burnout in intensive care units - a consideration of the possible prevalence and frequency of new risk factors: A descriptive correlational multicentre study. BMC Anesthesiology. 2013;13:38.
51. Antonsdottir I, Rushton CH, Nelson KE, Heinze KE, Swoboda SM, Hanson GC. Burnout and moral resilience in interdisciplinary healthcare professionals. Journal of Clinical Nursing. 2022;31(1):196-208.
52. Chor WPD, Ng WM, Cheng L, Situ W, Chong JW, Ng LYA, et al. Burnout amongst emergency healthcare workers during the COVID-19 pandemic: A multi-center study. American Journal of Emergency Medicine. 2021;46:700-2.
53. Schmidt C, Roffler M. Who isn't burned out? The role of spirituality in graduate medical education (GME). 2018.
54. Doolittle B, Windish D, Doolittle BR, Windish DM. Correlation of burnout syndrome with specific coping strategies, behaviors, and spiritual attitudes among interns at Yale University, New Haven, USA. JOURNAL OF EDUCATIONAL EVALUATION FOR HEALTH PROFESSIONS. 2015;12.
55. McKinley N, McCain RS, Convie L, Clarke M, Dempster M, Campbell WJ, et al. Resilience, burnout and coping mechanisms in UK doctors: a cross-sectional study. BMJ open. 2020;10(1):e031765.
56. Roslan NS, Yusoff MSB, Ab Razak A, Morgan K, Shauki NIA, Kukreja A, et al. Training Characteristics, Personal Factors and Coping Strategies Associated with Burnout in Junior Doctors: A Multi-Center Study. HEALTHCARE. 2021;9(9).
57. Azoulay E, De Waele J, Ferrer R, Staudinger T, Borkowska M, Povoa P, et al. Symptoms of burnout in intensive care unit specialists facing the COVID-19 outbreak. Annals of Intensive Care. 2020;10(1):110.
58. Ben-Itzhak S, Dvash J, Maor M, Rosenberg N, Halpern P. Sense of meaning as a predictor of burnout in emergency physicians in Israel: a national survey. Clinical and Experimental Emergency Medicine. 2015;2(4):217-25.
59. Correia I, Almeida AE. Organizational Justice, Professional Identification, Empathy, and Meaningful Work During COVID-19 Pandemic: Are They Burnout Protectors in Physicians and Nurses? FRONTIERS IN PSYCHOLOGY. 2020;11.
60. Doolittle BR. Association of Burnout with Emotional Coping Strategies, Friendship, and Institutional Support Among Internal Medicine Physicians. JOURNAL OF CLINICAL PSYCHOLOGY IN MEDICAL SETTINGS. 2020.
61. Doolittle BR, Windish DM, Seelig CB. Burnout, coping, and spirituality among internal medicine resident physicians. J Grad Med Educ. 2013;5(2):257-61.
62. Gribben J, Kase S, Waldman E, Weintraub A, Gribben JL, Kase SM, et al. A Cross-Sectional Analysis of Compassion Fatigue, Burnout, and Compassion Satisfaction in Pediatric Critical Care Physicians in the United States*. PEDIATRIC CRITICAL CARE MEDICINE. 2019;20(3):213-22.
63. Ntantana A, Matamis D, Savvidou S, Giannakou M, Gouva M, Nakos G, et al. Burnout and job satisfaction of intensive care personnel and the relationship with personality and religious traits: An observational, multicenter, cross-sectional study. Intensive and Critical Care Nursing. 2017;41:11-7.
64. Salmoirago-Blotcher E, Fitchett G, Leung K, Volturo G, Boudreaux E, Crawford S, et al. An exploration of the role of religion/spirituality in the promotion of physicians' wellbeing in Emergency Medicine. Preventive Medicine Reports. 2016;3:189-95.
65. St Onge JE, Allespach H, Diaz Y, Poitier A, Tamariz L, Paidas C, et al. Burnout: exploring the differences between U.S. and international medical graduates. BMC medical education. 2022;22(1):69.
66. Watson A, Saggar V, MacDowell C, McCoy J, Watson AG, Saggar V, et al. Self-reported modifying effects of resilience factors on perceptions of workload, patient outcomes, and burnout in physician-attendees of an international emergency medicine conference. PSYCHOLOGY HEALTH & MEDICINE. 2019;24(10):1220-34.
67. Mantri S, Lawson J, Wang Z, Koenig H, Mantri S, Lawson JM, et al. Identifying Moral Injury in Healthcare Professionals: The Moral Injury Symptom Scale-HP. JOURNAL OF RELIGION & HEALTH. 2021.
68. Guest RS, Baser R, Li Y, Scardino PT, Brown AE, Kissane DW. Cancer surgeons' distress and well-being, II: modifiable factors and the potential for organizational interventions. Annals of surgical oncology. 2011;18(5):1236-42.

For peer review only

69. Harper L, Alshammari D, Ferdynus C, Kalfa N. Burnout amongst members of the French-speaking Society of Pediatric and Adolescent Urology (SFUPA). Are there specific risk factors? Journal of pediatric urology. 2020;16(4):482-6.
70. Ramondetta L, Urbauer D, Brown A, Richardson G, Thaker P, Koenig H, et al. Work related stress among gynecologic oncologists. GYNECOLOGIC ONCOLOGY. 2011;123(2):365-9.
71. Suttle M, Chase M, Sasser W, Moore-Clingenpeel M, Maa T, Werner J, et al. Burnout in Pediatric Critical Care Medicine Fellows*. CRITICAL CARE MEDICINE. 2020;48(6):872-80.
72. Yoon J, Rasinski K, Curlin F, Yoon JD, Rasinski KA, Curlin FA. Conflict and emotional exhaustion in obstetrician-gynaecologists: a national survey. JOURNAL OF MEDICAL ETHICS. 2010;36(12):731-5.
73. Leu S, Vuille-dit-Bille R, Fink L, Soll C, Staerkle R, Leu S, et al. Burnout in Swiss and Australian surgeons in training-a cross-sectional study. EUROPEAN SURGERY-ACTA CHIRURGICA AUSTRIACA.
74. Schmidt C, Roffler M. Coping with the Practice of Medicine: Religion, Spirituality, and Other Personal Strategies. JOURNAL OF RELIGION & HEALTH. 2021;60(3):2092-108.
75. Alosaimi FD, Alawad HS, Alamri AK, Saeed AI, Aljuaydi KA, Alotaibi AS, et al. Stress and coping among consultant physicians working in Saudi Arabia. ANNALS OF SAUDI MEDICINE. 2018;38(3):214-24.
76. Glasberg J, Horiuti L, Novais M, Canavezzi A, Miranda V, Chicoli F, et al. Prevalence of the burnout syndrome among Brazilian medical oncologists. REVISTA DA ASSOCIACAO MEDICA BRASILEIRA. 2007;53(1):85-9.
77. Ramondetta LM, Urbauer D, Brown AJ, Richardson G, Thaker PH, Koenig HG, et al. Work related stress among gynecologic oncologists. GYNECOLOGIC ONCOLOGY. 2011;123(2):365-9.
78. Maslach C, Leiter MP. The Truth About Burnout: How Organizations Cause Personal Stress and What to Do About It: Wiley; 2008.
79. Li-Sauerwine S, Rebillot K, Melamed M, Addo N, Lin M. A 2-Question Summative Score Correlates with the Maslach Burnout Inventory. West J Emerg Med. 2020;21(3):610-7.
80. Demerouti E, Bakker AB. The Oldenburg Burnout Inventory: A good alternative to measure burnout and engagement. Handbook of stress and burnout in health care. 2008:65-78.
81. Kristensen TS, Borritz M, Villadsen E, Christensen KB. Copenhagen burnout inventory. Work & Stress. 1999.
82. Winwood PC, Winefield AH. Comparing Two Measures of Burnout Among Dentists in Australia. International Journal of Stress Management. 2004;11(3):282-9.
83. Swami M, Mathur D, Pushp B. Emotional intelligence, perceived stress and burnout among resident doctors: an assessment of the relationship. The National medical journal of India. 2013;26(4):210-3.
84. Teles R, Valle A, Rodríguez S, Piñeiro I, Regueiro B. Perceived Stress and Indicators of Burnout in Teachers at Portuguese Higher Education Institutions (HEI). Int J Environ Res Public Health. 2020;17(9):3248.
85. Ben-Itzhak S, Dvash J, Maor M, Rosenberg N, Halpern P, Ben-Itzhak S, et al. Sense of meaning as a predictor of burnout in emergency physicians in Israel: a national survey. CLINICAL AND EXPERIMENTAL EMERGENCY MEDICINE. 2015;2(4):217-25.
86. Facit.org. FACIT-Sp Non-Illness [Available from: https://www.facit.org/measures/FACIT-Sp-Non-Illness.
87. Hungelmann J, Kenkel-Rossi E, Klassen L, Stollenwerk R. Focus on Spiritual Well-Being: Harmonious interconnectedness of mind-body-spirit—Use of the JAREL Spiritual Well-Being Scale: Assessment of spiritual well-being is essential to the health of individuals. Geriatric Nursing. 1996;17(6):262-6.
88. Hatch RL, Burg MA, Naberhaus DS, Hellmich LK. The spiritual involvement and beliefs scale. Journal of Family Practice. 1998;46(6):476-86.
89. Büssing A, Hirdes AT, Baumann K, Hvidt NC, Heusser P. Aspects of Spirituality in Medical Doctors and Their Relation to Specific Views of Illness and Dealing with Their Patients' Individual Situation. Evidence-Based Complementary and Alternative Medicine. 2013;2013:734392.
90. Institute F. Multidimensional Measurement of Religiousness/Spirituality for Use in Health Research [Available from: https://fetzer.org/resources/multidimensional-measurement-religiousnessspirituality-use-health-research.
91. Daaleman TP, Frey BB, Wallace D, Studenski SA. The Spirituality Index of Well-Being: Development and testing of a new measure. J Fam Pract. 2002;51(11):952.
92. Monod S, Brennan M, Rochat E, Martin E, Rochat S, Büla CJ. Instruments Measuring Spirituality in Clinical Research: A Systematic Review. Journal of General Internal Medicine. 2011;26(11):1345.
93. Carver CS. You want to measure coping but your protocol's too long: consider the brief COPE. Int J Behav Med. 1997;4(1):92-100.
94. Hobfoll SE. Conservation of Resources: A New Attempt at Conceptualizing Stress. The American psychologist. 1989;44(3):513-24.
95. Koenig H, Parkerson Jr GR, Meador KG. Religion index for psychiatric research. The American Journal of Psychiatry. 1997;154(6):885-6.

For peer review only

96. Gerber MM, Boals A, Schuettler D. The unique contributions of positive and negative religious coping to posttraumatic growth and PTSD. Psychology of religion and spirituality. 2011;3(4):298-307.
97. PRRI. The 2020 Census of American Religion 2021 [07.08.2021]. Available from: https://www.prri.org/research/2020-census-of-american-religion/.
98. Centre PR. With high levels of prayer, U.S. is an outlier among wealthy nations 2019 [Available from: https://www.pewresearch.org/fact-tank/2019/05/01/with-high-levels-of-prayer-u-s-is-an-outlier-among-wealthy-nations/.
99. Centre PR. How do European countries differ in religious commitment? Use our interactive map to find out 2018 [Available from: https://www.pewresearch.org/fact-tank/2018/12/05/how-do-european-countries-differ-in-religious-commitment/.
100. Zgliczyńska M, Zgliczyński S, Ciebiera M, Kosińska-Kaczyńska K. Occupational Burnout Syndrome in Polish Physicians: A Systematic Review. Int J Environ Res Public Health. 2019;16(24).
101. Williams ES, Rathert C, Buttigieg SC. The Personal and Professional Consequences of Physician Burnout: A Systematic Review of the Literature. Medical Care Research and Review. 2019;77(5):371-86.
102. Balendran B, Bath MF, Awopetu AI, Kreckler SM. Burnout within UK surgical specialties: a systematic review. The Annals of The Royal College of Surgeons of England. 2021;103(7):464-70.
103. Chow HHE, Chew QH, Sim K. Spirituality and religion in residents and inter-relationships with clinical practice and residency training: A scoping review. BMJ Open. 2021;11(5):e044321.
104. Sibeoni J, Bellon-Champel L, Mousty A, Manolios E, Verneuil L, Revah-Levy A. Physicians' Perspectives About Burnout: a Systematic Review and Metasynthesis. Journal of General Internal Medicine. 2019;34(8):1578-90.
105. Kang MJ, Park JH. The effects of COVID-19 on physician's burnout: a systematic review. J Korean Med Assoc. 2021;64(9):636-46.
106. Lal A, Tharyan A, Tharyan P. The prevalence, determinants and the role of empathy and religious or spiritual beliefs on job stress, job satisfaction, coping, burnout, and mental health in medical and surgical faculty of a teaching hospital: A cross-sectional survey. La Revue de Médecine Interne. 2020.
107. Williamson K, Lank PM, Cheema N, Hartman N, Lovell EO. Comparing the Maslach Burnout Inventory to Other Well-Being Instruments in Emergency Medicine Residents. J Grad Med Educ. 2018;10(5):532-6.
108. de Jager Meezenbroek E, Garssen B, van den Berg M, van Dierendonck D, Visser A, Schaufeli WB. Measuring Spirituality as a Universal Human Experience: A Review of Spirituality Questionnaires. Journal of Religion and Health. 2012;51(2):336-54.
109. Lu J, Lo C-L, Bang NM, Romero D. A Critical Review of Spiritual Well-Being Scales: Implications for Research, Practice, and Education in Rehabilitation Counseling. Rehabilitation Counseling Bulletin. 2018;62(3):144-56.
110. Assing Hvidt E, Søndergaard J, Hansen DG, Gulbrandsen P, Ammentorp J, Timmermann C, et al. 'We are the barriers': Danish general practitioners' interpretations of why the existential and spiritual dimensions are neglected in patient care. Commun Med. 2017;14(2):108-20.
111. Scheepers RA, Emke H, Epstein RM, Lombarts KMJMH. The impact of mindfulness-based interventions on doctors' well-being and performance: A systematic review. Medical Education. 2020;54(2):138-49.
112. Enhancement CL. Preventing Burnout [Available from: https://inside.ewu.edu/calelearning/psychological-skills/preventing-burnout/.
113. HelpGuide.org. Burnout Prevention and Treatment [Available from: https://www.helpguide.org/articles/stress/burnout-prevention-and-recovery.htm.

Supplementary Materials-

Supplementary Table 1, Supplementary Table 2
Prisma S, Prisma Abstract and Prisma 2020 checklists
Full protocol (supplementary file 2)
Full search strategy (supplementary file 1)

For peer review only

List of figures

Figure 1: Prisma Diagram

Figure 2: Medical specialty of participants

Figure 3: Geographical location of participants of studies included in this review

For peer review only

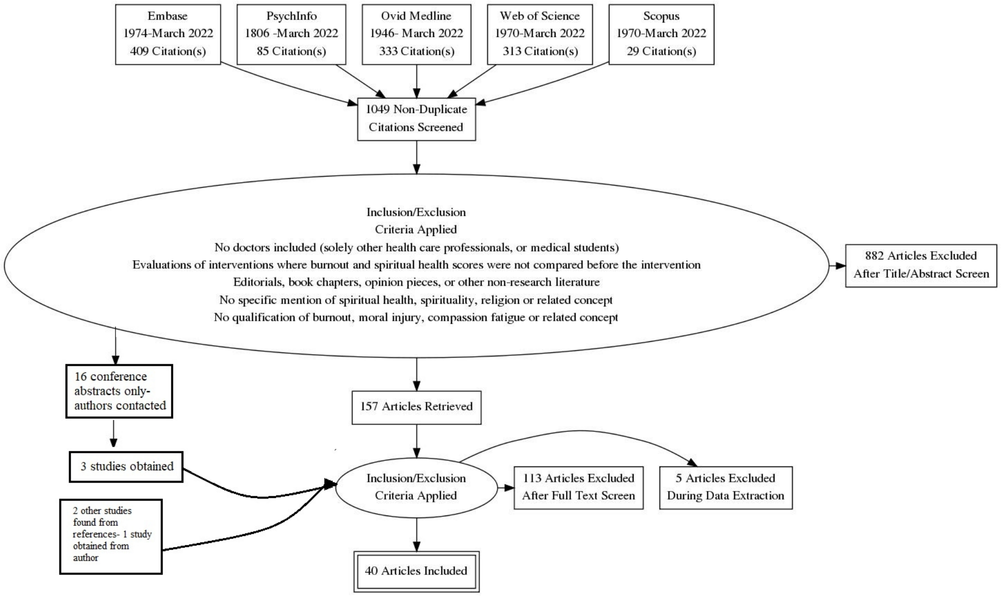

Figure 1: Prisma Diagram

383x227mm (96 x 96 DPI)

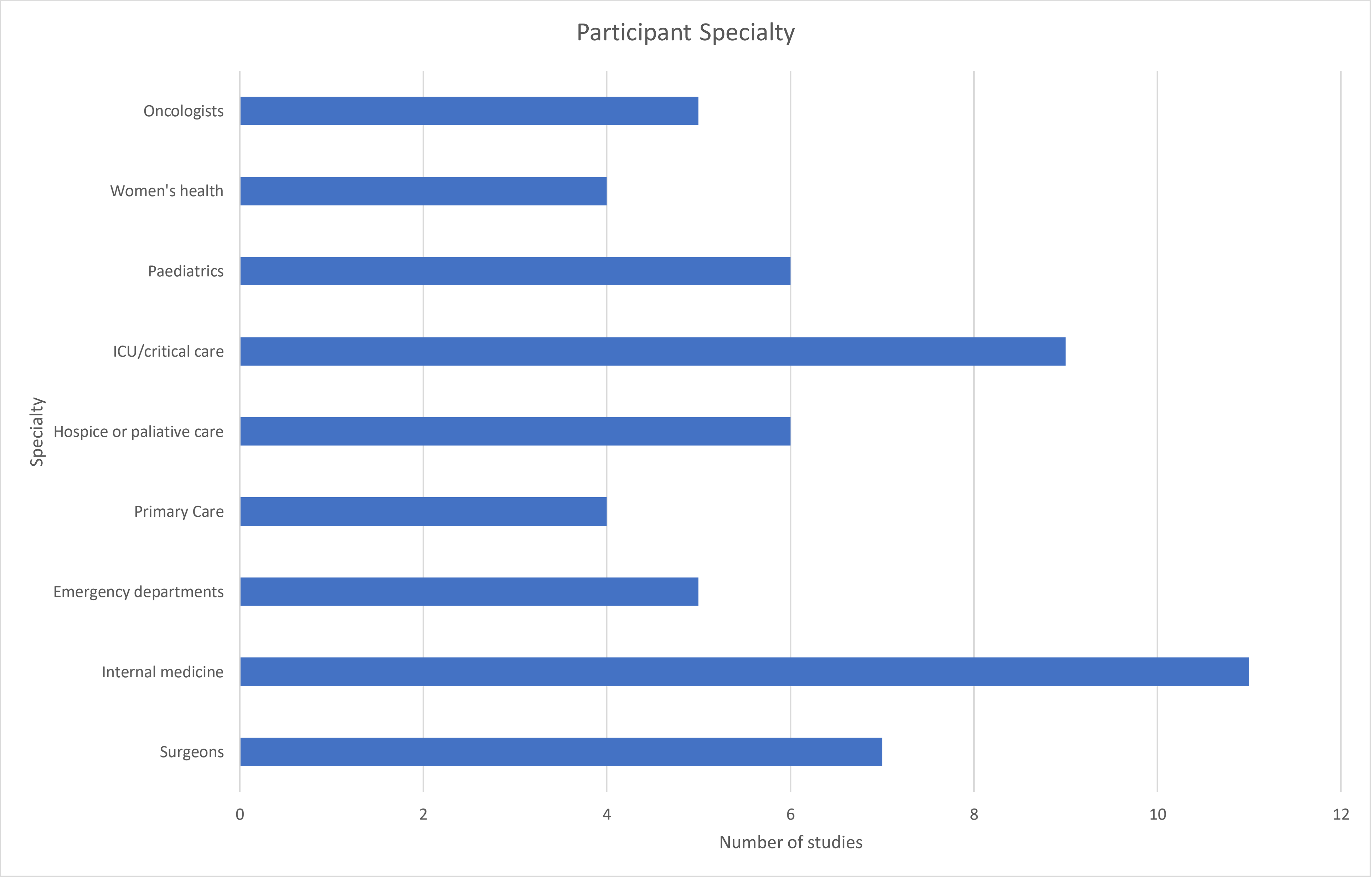
Participant Specialty
Oncologists
Women's health
Paediatrics
ICU/critical care
Hospice or paliative care
Primary Care
Emergency departments
Internal medicine
Surgeons
Specialty
0
2
4
6
8
10
12
Number of studies

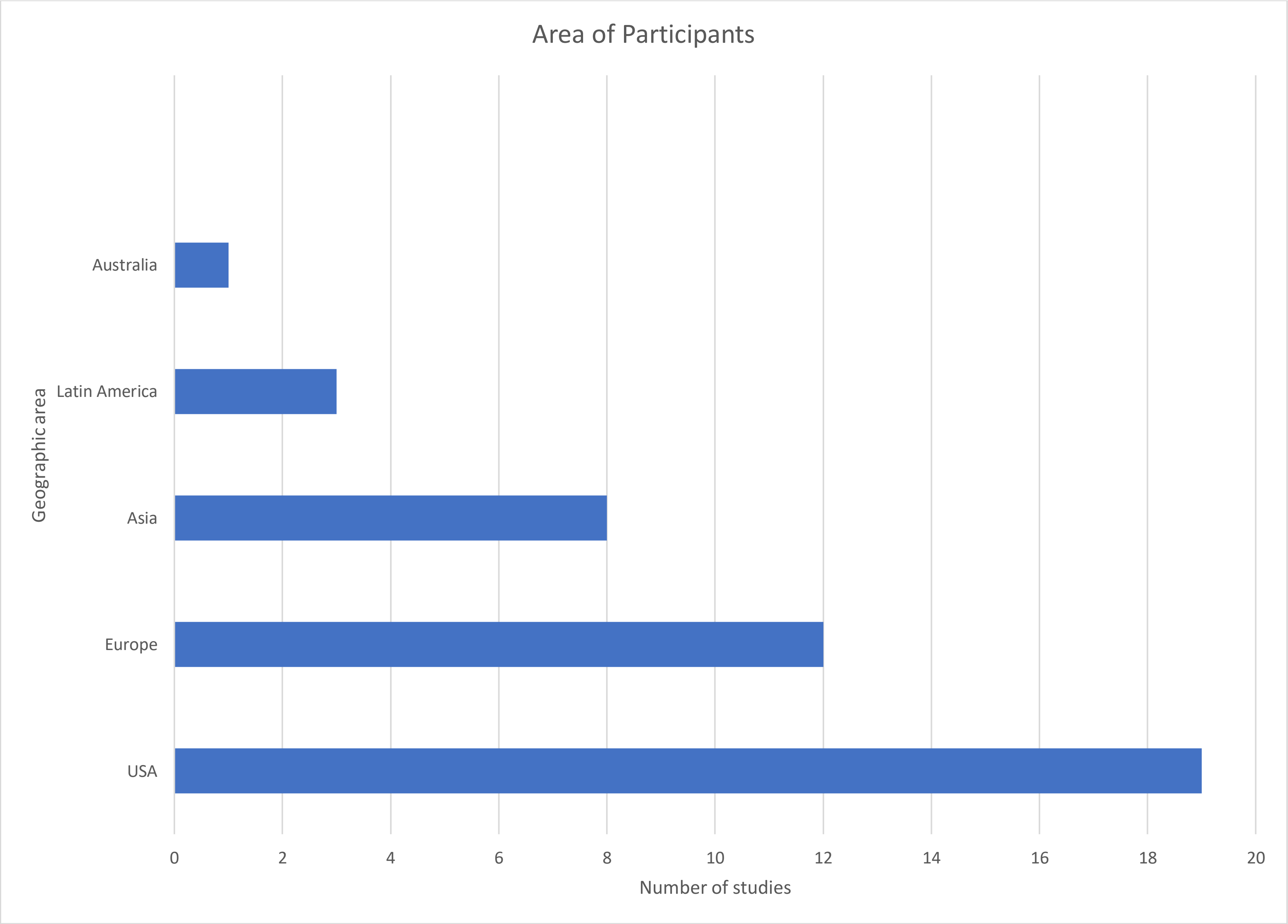
Area of Participants
Geographic area
Australia
Latin America
Asia
Europe
USA
0
2
4
6
8
10
12
14
16
18
20
Number of studies

Table 1: Findings from each study

Study	Comparisons of Burnout and Spiritual Health	Estimate or test statistic (95% confidence intervals in brackets, if given)	P value	The association of burnout (or similar concepts) with spiritual health (or similar concepts)
Alosaimi et al(1) 2018 Saudi Arabia	Perceived stress score was compared with use of religious coping (Brief-COPE)	Spearman's rho −0.09	0.030	Lower stress scores in those with higher religious coping
	Perceived stress score was compared for those who use religious coping (open ended question)	Number of participants:<br>Low PSS-66<br>Medium PSS- 48<br>High PSS- 33	0.004	
Antonsdottir et al(2) 2022 USA	Religion was compared with each domain of the MBI	DP β / EE β / PA β<br>Christian: −2.73*** / −4.97** / 1.76*<br>Jewish: −0.55 / −1.82 / 0.03<br>Spiritual, not religious: −2.35** / −2.64 / 1.86<br>Other: −2.58*** / −3.59 / 1.53	*** p<0.001<br>**p<0.01<br>*p<0.05	Lower burnout scores in those with a religious/spirituality.
Azoulay(3) 2020 Europe	Anxiety and depression scores were compared with religiosity			Burnout score and religiosity not commented on.
Baruah(4) 2019 India	MBI compared with type of religion (no non-religious)	EE: χ2= 1.881	DP p=0.7579<br>EE p=0.3905<br>PA p=1.00	No association found
Ben-Itzak(5) 2015 Israel	The extent to which work provides meaning was associated with burnout scores	β=-3.144 (0.045- 6.24)	P<0.05	Higher work related meaning was associated with lower burnout scores.

Büssing et al(6) 2014 Germany	The aspects of spirituality domains were compared with the cool down index and perceived work burden	Aspects of spirituality domains / Cool down index / Perceived work burden<br>*Religious orientation* ρ=−0.078 ρ=−0.044<br>*Search for insight/wisdom* ρ=−0.107 ρ=−0.036<br>*Conscious interactions* ρ=−0.14* ρ=−0.165*<br>*Transcendence conviction* ρ=−0.106 ρ=−0.044	*P<0.01	No relationship- no statistical association between spiritual score and cool down reactions or perceived work burden
Chor et al(7) 2021 Singapore	55.8% used religious coping more than acts of gratitude from the public.			Burnout and religious coping use not compared.
Clark(8) 2007 USA	The statistical model chosen assumes a relationship between spirituality and job satisfaction via integration and self-actualisation	χ^2 0.614	p- 0.433	Higher job satisfaction was associated with higher spirituality
Correia et al(9) 2020 Portugal	Religiosity was compared with burnout score domains	Exhaustion- β= 0.07<br>Disengagement- β= 0.02		No association
Das(10) 2016 India	Religion was compared with burnout domains	EE- χ^2= 1.556<br>DP- χ^2= 2.917<br>PA- χ^2= 1.556	EE- p= 0.212<br>DP- p= 0.233<br>PA- p= 0.459	No association

Doolittle et al(11)2013<br>USA	Correlation between SIBS score and MBI	EE- r=-0.11<br>DP- r=-0.13<br>PA- r=0.28	EE- p=0.25<br>DP- p=0.17<br>PA- p=0.003	No statistical association
Doolittle(12) 2020<br>USA	Three domains from the Durel are reported as Pearson's correlation coefficients	EE / DP / PA<br>Organized religion: − 0.12 / − 0.21 / + 0.14<br>Private practices: − 0.21 / − 0.12 / + 0.23<br>Religiosity: − 0.18 / − 0.18 / + 0.20		No association
Doolittle and Windish(13) 2015<br>USA	Spearman's rank correlation coefficient	EE- 'not significant'<br>DP- 'not significant'<br>PA- 0.35	PA- p=0.03	No association
Frank(14) 1999<br>USA	Religious fervour was compared with desire to become a physician again	Odds ratio- 1.3 (1.1-1.6)		No direct association found
Glasberg et al(15) 2007<br>Brazil	Religious identity was compared with MBI scores		p=0.038	Higher burnout in the non-religious
Glebocka et al(16) 2007<br>Poland	Hobfall's gains and losses were compared with MBI scores (translated), correlation coefficients reported	EE / DP / Professional Cynicism<br>Spiritual resources gain: 0.14 / 0.13 / 0.29<br>Spiritual resources loss: 0.09 / 0.35* / -0.06	*p<0.05	Positive correlation between burnout and spiritual resource loss.
Gribben et al(17) 2019<br>USA	Compares prayer/meditation and compassion fatigue	β=0.12	p<0.001	Higher compassion fatigue in those using spiritual type behaviours

Guay et al(18) 2019 Americas	Number of participants with burnout compared by religion.	χ^2 values not given	Religious affiliation p= 0.184 Importance of spirituality p=0.984 Importance of religion p=0.084	No association
Guest et al(19) 2011 USA	Spirituality mentioned as protective factor against burnout	Numeric data not given		MBI and spirituality not compared
Harper et al(20) 2020 USA	Believer/non-believers MBI scores compared	χ^2 values not given	p=0.69	No association
Koh et al(21) 2015 Singapore	The odds of being burned out and answering 'no' to being spiritual were compared.	odds ratios:<br>High DP- 2.83 (1.2-6.3)<br>Low PA- 2.85 (1.4-6.0)<br>High EE- 1.67 (0.76-3.64)<br>Overall burnout- 1.80 (0.86-3.71)		Those who answered 'no' to the spiritual question had lower personal accomplishment
Lal et al(22) 2020 India	MBI domains were compared with whether religious or spiritual beliefs influence work	ANOVA values not given	PA p=0.002	No overall association
Leonelli et al(23) 2017 Brazil	Perceived stress was compared with religious activity	β= 3.1 [0.3- 5.8]	p=0.03	Lower religious activity associated with higher perceived stress
Leu et al(24) 2020 Switzerland and Australia	Atheism vs any religion was compared with burnout	β= -52.5	p=0.13	No association

Macuka et al(25) 2020 Croatia	Compares spiritual question and burnout scores	EE- β= -0.20<br>r=-0.19<br>Disengagement- β=-0.18<br>r=-0.19	$p<0.05$<br>$p<0.05$<br>$p<0.05$<br>$p<0.05$	Religiosity was associated with lower emotional exhaustion.
Macuka et al 2021(26) Croatia	Spiritual question and job satisfaction were compared	r=0.15	$p>0.05$	Higher job satisfaction in those with higher religiosity
Mantri et al 2021(27) USA	Compares religiosity and moral injury scores	data not given		Moral injury symptoms and burnout inversely related to religiosity.
McKinley et al 2020(28) full data from Dr McKinley's thesis(29)	Religious coping was compared with burnout score	Unadjusted β= -0.42	$p<0.01$	Higher religious coping associated with lower burnout
Ntantana et al(30) 2017 Greece	Spirituality and burnout were compared, only EE reported	OR for high burnout given high EE- 1.482 (0.823-2.670)		No association
Purviss et al(31) 2019 USA	Religion (Catholics vs other) was compared with burnout score.		p = 0.026	No association
Ramondetta et al(32) 2011 Greece	Correlation between religiosity and Work Related Strain Inventory (WRSI)	Duke religiosity index and WRSI r=0.08<br>Intrinsic religiosity scale and WRSI r=0.05		No association
Roslan et al(33) 2021 Malaysia	Irregular spirituality routines were compared with burnout scores	OR- 2.24 (1.49–3.37))	$p<0.001$	Work related burnout was associated with irregular spirituality routines

Salmoirago-Blotcher(34)<br>2016<br>USA	Various religious/spiritual questions were compared with burnout scores	**Organized religiosity** — **Adjusted OR**<br>Never — Ref.<br>Any — 0.89 (0.36 to 2.20)<br>**Religious Affiliation**<br>None — Ref.<br>Any — 0.51 (0.19 to 1.39)<br>***Private prayer***<br>Never — Ref.<br>Any — 0.66 (0.27 to 1.60)<br>***Meditation***<br>Never — Ref.<br>Any — 0.56 (0.19 to 1.62)<br>**Self-rated spirituality**<br>Slightly/not spiritual — Ref.<br>Moderately spiritual — 1.14 (0.45 to 2.88)<br>Very spiritual — 0.15 (0.02 to 1.46)<br>**Religious rest**<br>Never — Ref.<br>Any — 0.51 (0.16 to 1.65)<br>***"I try hard to carry my religious beliefs over into all my life"***		No association

		Disagree Ref.<br>Agree 0.70 (0.28 to 1.74)		
Schmidt and Roffler(35) 2021<br><br>Quantitative data given by author, unpublished(36)	Correlation between spiritual score and burnout score	DP ρ= -0.37<br>EE ρ= -0.55<br>PA ρ= 0.62	p<0.05<br>p<0.05<br>p<0.05	Association between all domains of the burnout score and the spiritual score.
See et al(37) 2018<br>Large study, across Asia	Compared religious background or belief and high burnout	OR 0.69 (0.49–0.96)	P- 0.027	Religiosity was found to be protective against burnout in medical doctors (and nurses).
Shetach et al(38) 2015<br>Israel	Job satisfaction scores for Christians were compared with non-Christians	Religion β<br>Christian 0.46<br>Non-Christian 0.41	p>0.05	No association
St Onge et al(39) 2022<br>USA	Spiritual question was compared with burnout scores	Personal burnout β= -0.77<br>Work burnout β= -1.87885<br>Patient burnout β= −1.65<br><br>Odds ratio for overall burnout: 0.55(0.23-1.31)	p>0.05<br>p>0.05<br>p>0.05<br><br>p= 0.18	No association
Suttle et al(40) 2020<br>Asia	Mean MBI scores were compared with yes/no to spiritual question	Mean<br>EE- 26.77<br>DP- 11.278<br>PA-36.620	 p= 0.036<br>p= 0.011<br>p= 0.021	Higher burnout scores were associated with identifying as non-spiritual.
Teixeira et al(41) 2013<br>Portugal	Burnout scores in the religious were compared to non-religious	Odds ratio for burnout: 0.907	p=0.747	No association

Watson(42) 2019 USA	Mean burnout domain scores were compared with religious/spiritual question	Spirituality/religion	Disagree	Neutral	Agree		Higher emotional exhaustion and lower personal accomplishment were correlated with those who disagreed with the spiritual question. No correlation with depersonalization. One-way ANOVA showed no difference between means.
		Mean EE	3.98	3.80	3.41	p=0.36	
		Mean DP	3.95	3.71	3.18	p=0.13	
		Mean PA	5.31	5.87	5.65	p=0.43	

MBI- Maslach Burnout Inventory DP- Depersonalisation EE- Emotional Exhaustion PA- Personal Accomplishment
SIBS- Hatch Spiritual Involvement and Beliefs Scale WRSI- Work Related Strain Inventory
Brief COPE- Brief Coping Orientation to Problems Experienced Inventory

For peer review only

1. Alosaimi FD, Alawad HS, Alamri AK, Saeed AI, Aljuaydi KA, Alotaibi AS, et al. Stress and coping among consultant physicians working in Saudi Arabia. Annals of Saudi Medicine. 2018;38(3):214-24.
2. Antonsdottir I, Rushton CH, Nelson KE, Heinze KE, Swoboda SM, Hanson GC. Burnout and moral resilience in interdisciplinary healthcare professionals. Journal of Clinical Nursing. 2022;31(1):196-208.
3. Azoulay E, De Waele J, Ferrer R, Staudinger T, Borkowska M, Povoa P, et al. Symptoms of burnout in intensive care unit specialists facing the COVID-19 outbreak. Annals of Intensive Care. 2020;10(1):110.
4. Baruah A, Das S, Dutta A, Das B, Sharma T, Hazarika M. DEGREE AND FACTORS OF BURNOUT AMONG EMERGENCY HEALTHCARE WORKERS IN INDIA. Int J Sci Res (Ahmedabad). 2019;8(4):41-5.
5. Ben-Itzhak S, Dvash J, Maor M, Rosenberg N, Halpern P. Sense of meaning as a predictor of burnout in emergency physicians in Israel: a national survey. Clinical and Experimental Emergency Medicine. 2015;2(4):217-25.
6. Büssing A, Lotzke D, Glockler M, Heusser P. Influence of Spirituality on Cool Down Reactions, Work Engagement, and Life Satisfaction in Anthroposophic Health Care Professionals. Evidence-Based Complementary and Alternative Medicine. 2015.
7. Chor WPD, Ng WM, Cheng L, Situ W, Chong JW, Ng LYA, et al. Burnout amongst emergency healthcare workers during the COVID-19 pandemic: A multi-center study. American Journal of Emergency Medicine. 2021;46:700-2.
8. Clark L, Leedy S, McDonald L, Muller B, Lamb C, Mendez T, et al. Spirituality and job satisfaction among hospice interdisciplinary team members. Journal of Palliative Medicine. 2007;10(6):1321-8.
9. Correia I, Almeida AE. Organizational Justice, Professional Identification, Empathy, and Meaningful Work During COVID-19 Pandemic: Are They Burnout Protectors in Physicians and Nurses? FRONTIERS IN PSYCHOLOGY. 2020;11.
10. Das S, Barman S, Datta S, Bardhan N, Baishya M, Das B, et al. Degree of burnout among emergency healthcare workers and factors influencing level of burnout: a pilot study. Delhi Psychiatry Journal. 2016;19:36-47.
11. Doolittle BR, Windish DM, Seelig CB. Burnout, coping, and spirituality among internal medicine resident physicians. Journal of graduate medical education. 2013;5(2):257-61.

12. Doolittle BR. Association of Burnout with Emotional Coping Strategies, Friendship, and Institutional Support Among Internal Medicine Physicians. Journal of Clinical Psychology in Medical Settings. 2020.
13. Doolittle BR, Windish DM. Correlation of burnout syndrome with specific coping strategies, behaviors, and spiritual attitudes among interns at Yale University, New Haven, USA. Journal of Educational Evaluation for Health Professions. 2015;12.
14. Frank E, McMurray J, Linzer M, Elon L. Career satisfaction of US women physicians - Results from the Women Physicians' Health Study. ARCHIVES OF INTERNAL MEDICINE. 1999;159(13):1417-26.
15. Glasberg J, Horiuti L, Novais M, Canavezzi A, Miranda V, Chicoli F, et al. Prevalence of the burnout syndrome among Brazilian medical oncologists. REVISTA DA ASSOCIACAO MEDICA BRASILEIRA. 2007;53(1):85-9.
16. Glebocka A, Lisowska E, Glebocka A, Lisowska E. Professional burnout and stress among polish physicians explained by the Hobfoll resources theory. JOURNAL OF PHYSIOLOGY AND PHARMACOLOGY. 2007;58:243-52.
17. Gribben J, Kase S, Waldman E, Weintraub A, Gribben JL, Kase SM, et al. A Cross-Sectional Analysis of Compassion Fatigue, Burnout, and Compassion Satisfaction in Pediatric Critical Care Physicians in the United States*. PEDIATRIC CRITICAL CARE MEDICINE. 2019;20(3):213-22.
18. Guay MD, Reyes Donoso MM, Lopez Saca JM, Riveros Rios ME, Pastrana T. Spirituality and Religiosity and Burnout in Latin-American Palliative Care Health Care Professionals (LAPC) (S818). Journal of Pain and Symptom Management. 2019;57(2):490-1.
19. Guest RS, Baser R, Li Y, Scardino PT, Brown AE, Kissane DW. Cancer surgeons' distress and well-being, II: modifiable factors and the potential for organizational interventions. Annals of surgical oncology. 2011;18(5):1236-42.
20. Harper L, Alshammari D, Ferdynus C, Kalfa N. Burnout amongst members of the French-speaking Society of Pediatric and Adolescent Urology (SFUPA). Are there specific risk factors? Journal of pediatric urology. 2020;16(4):482-6.
21. Koh MYH, Chong PH, Neo PSH, Ong YJ, Yong WC, Ong WY, et al. Burnout, psychological morbidity and use of coping mechanisms among palliative care practitioners: A multi-centre cross-sectional study. Palliative Medicine. 2015;29(7):633-42.
22. Lal A, Tharyan A, Tharyan P. The prevalence, determinants and the role of empathy and religious or spiritual beliefs on job stress, job satisfaction, coping, burnout, and mental health in medical and surgical faculty of a teaching hospital: A cross-sectional survey. La Revue de Médecine Interne. 2020.
23. Leonelli LB, Andreoni S, Martins P, Kozasa EH, Salvo VLd, Sopezki D, et al. Perceived stress among Primary Health Care Professionals in Brazil. Estresse percebido em profissionais da Estrategia Saude da Familia. 2017;20(2):286-98.
24. Leu S, Vuille-dit-Bille R, Fink L, Soll C, Staerkle R, Leu S, et al. Burnout in Swiss and Australian surgeons in training-a cross-sectional study. EUROPEAN SURGERY-ACTA CHIRURGICA AUSTRIACA.
25. Macuka I, Junakovic I, Bozic D, Macuka I, Junakovic IT, Bozic D. Burnout among Palliative Care Professionals. DRUSTVENA ISTRAZIVANJA. 2020;29(2):287-308.
26. Macuka I, Tucak Junakovic I. A Cross-Sectional Study of Job Satisfaction and Intention to Leave Job in Palliative Care in Croatia. Journal of palliative care. 2021:8258597211046704.
27. Mantri S, Lawson JM, Wang ZZ, Koenig HG. Prevalence and Predictors of Moral Injury Symptoms in Health Care Professionals. JOURNAL OF NERVOUS AND MENTAL DISEASE. 2021;209(3):174-80.
28. McKinley N, McCain RS, Convie L, Clarke M, Dempster M, Campbell WJ, et al. Resilience, burnout and coping mechanisms in UK doctors: a cross-sectional study. BMJ Open. 2020;10(1):e031765.
29. McKinley NC. Resilience, professional quality of life and coping mechanisms in doctors and medical students.: Queens University Belfast; 2021.
30. Ntantana A, Matamis D, Savvidou S, Giannakou M, Gouva M, Nakos G, et al. Burnout and job satisfaction of intensive care personnel and the relationship with personality and religious traits: An observational, multicenter, cross-sectional study. Intensive and Critical Care Nursing. 2017;41:11-7.

31. Purvis T, Saylor D, Powell B, Biba G, Conti D, Crowe T, et al. Burnout and Resilience Among Neurosciences Critical Care Unit Staff. NEUROCRITICAL CARE. 2019;31(2):406-10.
32. Ramondetta L, Urbauer D, Brown A, Richardson G, Thaker P, Koenig H, et al. Work related stress among gynecologic oncologists. GYNECOLOGIC ONCOLOGY. 2011;123(2):365-9.
33. Roslan NS, Yusoff MSB, Ab Razak A, Morgan K, Shauki NIA, Kukreja A, et al. Training Characteristics, Personal Factors and Coping Strategies Associated with Burnout in Junior Doctors: A Multi-Center Study. HEALTHCARE. 2021;9(9).
34. Salmoirago-Blotcher E, Fitchett G, Leung K, Volturo G, Boudreaux E, Crawford S, et al. An exploration of the role of religion/spirituality in the promotion of physicians' wellbeing in Emergency Medicine. Preventive Medicine Reports. 2016;3:189-95.
35. Schmidt C, Roffler M. Coping with the Practice of Medicine: Religion, Spirituality, and Other Personal Strategies. JOURNAL OF RELIGION & HEALTH. 2021;60(3):2092-108.
36. Schmidt C, Roffler M. Who isn't burned out? The role of spirituality in graduate
medical education (GME). 2018.
37. See K, Zhao M, Nakataki E, Chittawatanarat K, Fang W, Faruq M, et al. Professional burnout among physicians and nurses in Asian intensive care units: a multinational survey. INTENSIVE CARE MEDICINE. 2018;44(12):2079-90.
38. Shetach A, Marcus O, Shetach A, Marcus O. The critical managerial capabilities of medical and nursing managers in an Israeli hospital. EVIDENCE-BASED HRM-A GLOBAL FORUM FOR EMPIRICAL SCHOLARSHIP. 2015;3(1):81-102.
39. St Onge JE, Allespach H, Diaz Y, Poitier A, Tamariz L, Paidas C, et al. Burnout: exploring the differences between U.S. and international medical graduates. BMC medical education. 2022;22(1):69.
40. Suttle M, Chase M, Sasser W, Moore-Clingenpeel M, Maa T, Werner J, et al. Burnout in Pediatric Critical Care Medicine Fellows*. CRITICAL CARE MEDICINE. 2020;48(6):872-80.
41. Teixeira C, Ribeiro O, Fonseca AM, Carvalho AS. Burnout in intensive care units - a consideration of the possible prevalence and frequency of new risk factors: A descriptive correlational multicentre study. BMC Anesthesiology. 2013;13:38.
42. Watson AG, Saggar V, MacDowell C, McCoy JV. Self-reported modifying effects of resilience factors on perceptions of workload, patient outcomes, and burnout in physician-attendees of an international emergency medicine conference. Psychology, health & medicine. 2019;24(10):1220-34.

For peer review only

Table 2: Study details, AXIS quality assessment, spiritual wellness assessment and burnout score used

Study	Study design	AXIS Quality rating	Population	Measure of spirituality	Measure of burnout
Alosaimi et al(1) 2018 Saudi Arabia	Cross-sectional study	16	582 consultant physicians	Brief COPE (2 items to score religious coping)	Perceived stress Scale
Antonsdottir et al(2) 2022 USA	Cross-sectional study	12	639 (96 physicians)	Religious affiliation (including 'spiritual but not religious")	MBI
Azoulay(3) 2020 Europe	Cross-sectional study	12	1132 respondents- ICU specialists	Self-rated religiosity 1-100	MBI
Baruah(4) 2019 India	Cross-sectional study	15	68 participants (doctors, nurses, and paramedics)- Emergency Department	Religion	MBI
Ben-Itzak(5) 2015 Israel	Cross-sectional study	10	70 emergency physicians	"My job gives me meaning" yes/no<br>Open ended questions: 1. Self-fulfilment<br>2. Meaningful relationships<br>3. Excitement and adrenaline rush<br>4. Providing meaningful assistance.	MBI
Büssing et al(6) 2014 Germany	Cross-sectional study	16	489 anthroposophic medical staff (41% physicians)	Aspects of Spirituality Questionnaire (ASQ)	Cool down Index<br>Utrecht Work engagement scale.

Chor et al(7) 2021 Singapore	Cross-sectional study	14	337 participants (127 doctors) Emergency department	Use of religious coping	Copenhagen Burnout Inventory
Clark(8) 2007 USA	Cross-sectional study	14	215 hospice staff, 2.3% physicians	Jarel spiritual wellbeing scale	8-item job satisfaction scale
Correia et al(9) 2020 Portugal	Cross-sectional study	12	497 health professionals working during COVID-19 (229 physicians)	"Regardless of whether you belong to a particular religion, how religious would you say you are?"	Oldenburg Burnout Inventory
Das(10) 2016 India	Cross-sectional study	12	7 emergency department participants (4 doctors)	Religion	MBI
Doolittle et al(11)2013 USA	Cross-sectional study	17	108 hospital residents	Hatch Spiritual Involvement and Beliefs Scale (SIBS), chosen to try and avoid cultural bias.	MBI
Doolittle(12) 2020 USA	Cross-sectional study	17	337 internal medicine physicians	COPE and DUREL, self reported spiritual and/or religious	ProQoL (domains of burnout, secondary stress, and compassion satisfaction)
Doolittle and Windish(13) 2015 USA	Cross-sectional study	15	Interns 66 in the summer, and 53 in winter cohorts	Coping Orientation to Problems Experienced (COPE), Hatch SIBS, self perception of spirituality and religiosity.	MBI

Frank(14) 1999 USA	Cross-sectional study	17	4501 female physicians	"Religious identification" none, low strong or very strong	"Are you generally satisfied with your career?" "If you lived your life again, would you become a physician?"
Glasberg et al(15) 2007 Brazil	Cross-sectional study	16	102 oncologists	Religious affiliation	MBI
Glebocka et al(16) 2007 Poland	Cross-sectional study	8	77 people (41 doctors)	Hobfoll's Questionnaire of Self-Assessment of Gains and Losses)7 statements of 'spiritual resources' gained in last 12 months)	MBI
Gribben et al(17) 2019 USA	Cross-sectional study	18	390 paediatric emergency medicine trainees	Prayer/meditation as self care activity	Compassion Fatigue and Satisfaction Self-Test for Helpers (adapted)
Guay et al(18) 2019 Americas	Cross-sectional study	8	221 (RR 63%) health care professions, 58.8% physicians,	Importance of religion<br>Importance of spirituality<br>Self-definition as religious person<br>Self-definition as spiritual person<br>Frequency of religious practice (praying/meditation)	Respondents categorised as burned out if they agreed with: ''I am definitely burning out and have one or more symptoms of burnout, such as physical and emotional exhaustion,'' ''The symptoms of burnout that I'm experiencing won't go away. I think about frustration at work a lot,'' ''I feel completely burned out and often wonder if I can go on. I am at the point where I may need some changes or may need to seek some sort of help.''<br><br>Respondents were categorized as not burned out if they agreed to either of the statements: (1) ''I am definitely burning out and have one or more symptoms of burnout, such as physical and emotional exhaustion, (2) Occasionally I am under stress, and I don't always have as much energy as I once did, but I don't feel burned out.''

Guest et al(19) 2011 USA	Cross-sectional study	10	72 cancer surgeons	Linear Analog Scale Assessment of Quality of Life (LASA QOL) which includes spiritual QoL	MBI
Harper et al(20) 2020 USA	Cross-sectional study	18	70 Urologists	Believer/non-believer	MBI
Koh et al(21) 2015 Singapore	Cross-sectional study	19	273 palliative care staff (28.1% doctors)	Single question: “Do you consider yourself spiritual?”	MBI
Lal et al(22) 2020 India	Cross-sectional study	19	345 of medical and surgical faculty	Single question- “My religious or spiritual beliefs strongly influence my approach to my work and patients.”	MBI<br>Job stress and job satisfaction questionnaire<br>Jefferson scale of physician empathy
Leonelli et al(23) 2017 Brazil	Cross-sectional study	19	450 primary health care professionals (27 doctors)	Religious activity	Perceived stress scale (PSS)continuous
Leu et al(24) 2020 Switzerland and Australia	Cross-sectional study	16	412 participants (237 Swiss and 175 Australian)	Religion	MBI
Macuka et al(25) 2020 Croatia	Cross-sectional study	12	123 palliative care staff (18 doctors)	“Whether or not you consider yourself a believer or not, how much of a spiritual person you consider yourself, that is, how much you are interested in the sacred or the supernatural?” likert scale 1-5	Oldenburg burnout inventory
Macuka et al 2021(26) Croatia		9			
Mantri et al 2021(27) USA	Cross-sectional study	18	181 participants	Religious affiliation, and ‘religious commitment’ using Belief into Action (BIAC) scale	Moral Injury Symptom Scale-Healthcare Professionals version and MBI

McKinley et al 2020(28) full data from Dr McKinley's thesis(29)	Cross-sectional study	17	1382 participants, 69% hospital, 31% primary care	BRIEF COPE, includes religious coping	ProQOL-V includes burnout, compassion satisfaction and secondary traumatic stress sub domains.
Ntantana et al(30) 2017 Greece	Cross-sectional study	18	149 physicians, 320 nurses from intensive care	Religious and Spiritual attitudes questionnaire(31), designed by Büssing for use with patients, rather than staff. The authors report it was chosen as it avoids exclusive religious terminology, however in the paper by Büssing referred to, they report that a positive attitude towards religion and spirituality is necessary for some aspects of the scale.	MBI
Purviss et al(32) 2019 USA	Cross-sectional study	16	64 neurosciences critical care staff (10 doctors)	Religion	MBI
Ramondetta et al(33) 2011 Greece	Cross-sectional study	15	273 oncologists	Religious affiliation, self-rated religiosity and spirituality, Intrinsic Religiosity Survey, and Duke religion Index (DRI- a measure organisational religiosity)	Work related strain inventory (WRSI)- correlates with MBI
Roslan et al(34) 2021 Malaysia	Cross-sectional study	20	754 hospital interns	BRIEF COPE, includes religious coping, and descriptors of regular versus irregular spirituality routines based on several major religions professed in the country.	Copenhagen Burnout Inventory
Salmoirago-Blotcher(35) 2016 USA	Cross-sectional study	16	138 emergency physicians	Religious affiliation, Self-rated spirituality, Fetzer Institute Multidimensional Measurement of Religiousness/Spirituality for Use in Health Research- used to assess religious/spiritual practice, and religious commitment ("I try hard to carry my religious beliefs over into all my other dealings in life), questions added to assess religious rest, and spiritual counsel.	2 item MBI

Schmidt and Roffler(36) 2021<br><br>Quantitative data given by author, unpublished(37)	Cross-sectional study	10	65 medical education faculty	The<br>Functional Assessment of Chronic Illness Therapy-Spiritual Well-Being Scale, a modified version for non-illness (FACIT-Sp-Non-Illness)	MBI
See et al(38) 2018<br>Large study, across Asia	Cross-sectional study	16	992 intensive care physicians	Religious background or belief	MBI
Shetach et al(39) 2015<br>Israel	Cross-sectional study	13	107 respondents (40 doctors)	Religion- (Christian vs non-Christian)	Job satisfaction questionnaire
St Onge et al(40) 2022<br>USA	Cross-sectional study	17	222 interns	"My spiritual beliefs are very important in helping me cope with stressors."	Copenhagen Burnout Inventory
Suttle et al(41) 2020<br>Asia	Cross-sectional study	12	47 programme directors and 187 fellows in paediatric critical care	"Do you consider yourself to be a spiritual person?" yes/no	MBI
Teixeira et al(42) 2013<br>Portugal	Cross-sectional study	14	300 Intensive Care staff (82 physicians)	Religion yes/no	MBI
Watson(43) 2019<br>USA	Cross-sectional study	13	54 emergency physicians	Single question- "spirituality/religion is important in my life"	MBI

MBI-Maslach Burnout Inventory

1. Alosaimi FD, Alawad HS, Alamri AK, Saeed AI, Aljuaydi KA, Alotaibi AS, et al. Stress and coping among consultant physicians working in Saudi Arabia. Annals of Saudi Medicine. 2018;38(3):214-24.
2. Antonsdottir I, Rushton CH, Nelson KE, Heinze KE, Swoboda SM, Hanson GC. Burnout and moral resilience in interdisciplinary healthcare professionals. Journal of Clinical Nursing. 2022;31(1):196-208.
3. Azoulay E, De Waele J, Ferrer R, Staudinger T, Borkowska M, Povoa P, et al. Symptoms of burnout in intensive care unit specialists facing the COVID-19 outbreak. Annals of Intensive Care. 2020;10(1):110.
4. Baruah A, Das S, Dutta A, Das B, Sharma T, Hazarika M. DEGREE AND FACTORS OF BURNOUT AMONG EMERGENCY HEALTHCARE WORKERS IN INDIA. Int J Sci Res (Ahmedabad). 2019;8(4):41-5.
5. Ben-Itzhak S, Dvash J, Maor M, Rosenberg N, Halpern P. Sense of meaning as a predictor of burnout in emergency physicians in Israel: a national survey. Clinical and Experimental Emergency Medicine. 2015;2(4):217-25.
6. Büssing A, Lotzke D, Glockler M, Heusser P. Influence of Spirituality on Cool Down Reactions, Work Engagement, and Life Satisfaction in Anthroposophic Health Care Professionals. Evidence-Based Complementary and Alternative Medicine. 2015.
7. Chor WPD, Ng WM, Cheng L, Situ W, Chong JW, Ng LYA, et al. Burnout amongst emergency healthcare workers during the COVID-19 pandemic: A multi-center study. American Journal of Emergency Medicine. 2021;46:700-2.
8. Clark L, Leedy S, McDonald L, Muller B, Lamb C, Mendez T, et al. Spirituality and job satisfaction among hospice interdisciplinary team members. Journal of Palliative Medicine. 2007;10(6):1321-8.
9. Correia I, Almeida AE. Organizational Justice, Professional Identification, Empathy, and Meaningful Work During COVID-19 Pandemic: Are They Burnout Protectors in Physicians and Nurses? FRONTIERS IN PSYCHOLOGY. 2020;11.
10. Das S, Barman S, Datta S, Bardhan N, Baishya M, Das B, et al. Degree of burnout among emergency healthcare workers and factors influencing level of burnout: a pilot study. Delhi Psychiatry Journal. 2016;19:36-47.
11. Doolittle BR, Windish DM, Seelig CB. Burnout, coping, and spirituality among internal medicine resident physicians. Journal of graduate medical education. 2013;5(2):257-61.
12. Doolittle BR. Association of Burnout with Emotional Coping Strategies, Friendship, and Institutional Support Among Internal Medicine Physicians. Journal of Clinical Psychology in Medical Settings. 2020.
13. Doolittle BR, Windish DM. Correlation of burnout syndrome with specific coping strategies, behaviors, and spiritual attitudes among interns at Yale University, New Haven, USA. Journal of Educational Evaluation for Health Professions. 2015;12.
14. Frank E, McMurray J, Linzer M, Elon L. Career satisfaction of US women physicians - Results from the Women Physicians' Health Study. ARCHIVES OF INTERNAL MEDICINE. 1999;159(13):1417-26.
15. Glasberg J, Horiuti L, Novais M, Canavezzi A, Miranda V, Chicoli F, et al. Prevalence of the burnout syndrome among Brazilian medical oncologists. REVISTA DA ASSOCIACAO MEDICA BRASILEIRA. 2007;53(1):85-9.
16. Glebocka A, Lisowska E, Glebocka A, Lisowska E. Professional burnout and stress among polish physicians explained by the Hobfoll resources theory. JOURNAL OF PHYSIOLOGY AND PHARMACOLOGY. 2007;58:243-52.
17. Gribben J, Kase S, Waldman E, Weintraub A, Gribben JL, Kase SM, et al. A Cross-Sectional Analysis of Compassion Fatigue, Burnout, and Compassion Satisfaction in Pediatric Critical Care Physicians in the United States*. PEDIATRIC CRITICAL CARE MEDICINE. 2019;20(3):213-22.
18. Guay MD, Reyes Donoso MM, Lopez Saca JM, Riveros Rios ME, Pastrana T. Spirituality and Religiosity and Burnout in Latin-American Palliative Care Health Care Professionals (LAPC) (S818). Journal of Pain and Symptom Management. 2019;57(2):490-1.

For peer review only

19. Guest RS, Baser R, Li Y, Scardino PT, Brown AE, Kissane DW. Cancer surgeons' distress and well-being, II: modifiable factors and the potential for organizational interventions. Annals of surgical oncology. 2011;18(5):1236-42.
20. Harper L, Alshammari D, Ferdynus C, Kalfa N. Burnout amongst members of the French-speaking Society of Pediatric and Adolescent Urology (SFUPA). Are there specific risk factors? Journal of pediatric urology. 2020;16(4):482-6.
21. Koh MYH, Chong PH, Neo PSH, Ong YJ, Yong WC, Ong WY, et al. Burnout, psychological morbidity and use of coping mechanisms among palliative care practitioners: A multi-centre cross-sectional study. Palliative Medicine. 2015;29(7):633-42.
22. Lal A, Tharyan A, Tharyan P. The prevalence, determinants and the role of empathy and religious or spiritual beliefs on job stress, job satisfaction, coping, burnout, and mental health in medical and surgical faculty of a teaching hospital: A cross-sectional survey. La Revue de Médecine Interne. 2020.
23. Leonelli LB, Andreoni S, Martins P, Kozasa EH, Salvo VLd, Sopezki D, et al. Perceived stress among Primary Health Care Professionals in Brazil. Estresse percebido em profissionais da Estrategia Saude da Familia. 2017;20(2):286-98.
24. Leu S, Vuille-dit-Bille R, Fink L, Soll C, Staerkle R, Leu S, et al. Burnout in Swiss and Australian surgeons in training-a cross-sectional study. EUROPEAN SURGERY-ACTA CHIRURGICA AUSTRIACA.
25. Macuka I, Junakovic I, Bozic D, Macuka I, Junakovic IT, Bozic D. Burnout among Palliative Care Professionals. DRUSTVENA ISTRAZIVANJA. 2020;29(2):287-308.
26. Macuka I, Tucak Junakovic I. A Cross-Sectional Study of Job Satisfaction and Intention to Leave Job in Palliative Care in Croatia. Journal of palliative care. 2021:8258597211046704.
27. Mantri S, Lawson JM, Wang ZZ, Koenig HG. Prevalence and Predictors of Moral Injury Symptoms in Health Care Professionals. JOURNAL OF NERVOUS AND MENTAL DISEASE. 2021;209(3):174-80.
28. McKinley N, McCain RS, Convie L, Clarke M, Dempster M, Campbell WJ, et al. Resilience, burnout and coping mechanisms in UK doctors: a cross-sectional study. BMJ Open. 2020;10(1):e031765.
29. McKinley NC. Resilience, professional quality of life and coping mechanisms in doctors and medical students.: Queens University Belfast; 2021.
30. Ntantana A, Matamis D, Savvidou S, Giannakou M, Gouva M, Nakos G, et al. Burnout and job satisfaction of intensive care personnel and the relationship with personality and religious traits: An observational, multicenter, cross-sectional study. Intensive and Critical Care Nursing. 2017;41:11-7.
31. Büssing A, Ostermann T, Matthiessen PF. Role of religion and spirituality in medical patients: confirmatory results with the SpREUK questionnaire. Health and quality of life outcomes. 2005;3(1):10.
32. Purvis T, Saylor D, Powell B, Biba G, Conti D, Crowe T, et al. Burnout and Resilience Among Neurosciences Critical Care Unit Staff. NEUROCRITICAL CARE. 2019;31(2):406-10.
33. Ramondetta L, Urbauer D, Brown A, Richardson G, Thaker P, Koenig H, et al. Work related stress among gynecologic oncologists. GYNECOLOGIC ONCOLOGY. 2011;123(2):365-9.
34. Roslan NS, Yusoff MSB, Ab Razak A, Morgan K, Shauki NIA, Kukreja A, et al. Training Characteristics, Personal Factors and Coping Strategies Associated with Burnout in Junior Doctors: A Multi-Center Study. HEALTHCARE. 2021;9(9).
35. Salmoirago-Blotcher E, Fitchett G, Leung K, Volturo G, Boudreaux E, Crawford S, et al. An exploration of the role of religion/spirituality in the promotion of physicians' wellbeing in Emergency Medicine. Preventive Medicine Reports. 2016;3:189-95.
36. Schmidt C, Roffler M. Coping with the Practice of Medicine: Religion, Spirituality, and Other Personal Strategies. JOURNAL OF RELIGION & HEALTH. 2021;60(3):2092-108.
37. Schmidt C, Roffler M. Who isn't burned out? The role of spirituality in graduate
medical education (GME). 2018.

38. See K, Zhao M, Nakataki E, Chittawatanarat K, Fang W, Faruq M, et al. Professional burnout among physicians and nurses in Asian intensive care units: a multinational survey. INTENSIVE CARE MEDICINE. 2018;44(12):2079-90.

39. Shetach A, Marcus O, Shetach A, Marcus O. The critical managerial capabilities of medical and nursing managers in an Israeli hospital. EVIDENCE-BASED HRM-A GLOBAL FORUM FOR EMPIRICAL SCHOLARSHIP. 2015;3(1):81-102.

40. St Onge JE, Allespach H, Diaz Y, Poitier A, Tamariz L, Paidas C, et al. Burnout: exploring the differences between U.S. and international medical graduates. BMC medical education. 2022;22(1):69.

41. Suttle M, Chase M, Sasser W, Moore-Clingenpeel M, Maa T, Werner J, et al. Burnout in Pediatric Critical Care Medicine Fellows*. CRITICAL CARE MEDICINE. 2020;48(6):872-80.

42. Teixeira C, Ribeiro O, Fonseca AM, Carvalho AS. Burnout in intensive care units - a consideration of the possible prevalence and frequency of new risk factors: A descriptive correlational multicentre study. BMC Anesthesiology. 2013;13:38.

43. Watson AG, Saggar V, MacDowell C, McCoy JV. Self-reported modifying effects of resilience factors on perceptions of workload, patient outcomes, and burnout in physician-attendees of an international emergency medicine conference. Psychology, health & medicine. 2019;24(10):1220-34.

For peer review only

Embase (accessed via Ovid):

1. exp religion/ or religio*.mp. or religiosity.mp. or exp philosophy/ or exp spiritual healing/ or theology/ or sikh.mp. or jain.mp. or bahai.mp. or pagan.mp. or shinto.mp. or cao dai.mp. or zoroast*.mp. or spirit*.mp. or Faith.mp. or holistic health.mp. or existential.mp. or salutogenesis.mp. or spiritual*.mp. or religious personnel/ or chaplain*.mp. or Buddhism.mp. or Christian*.mp. or Hinduism.mp. or islam.mp. or Judaism.mp. or humanism.mp. or Confucian*.mp. or "sense of coherence".mp.

2. exp job stress/ or compassion fatigue.mp. or occupational stress.mp. or burnout.mp. or exp burnout/ or exp Maslach Burnout Inventory/ or "work related stress*".mp. or "occupational stress*".mp. or "work stress*".mp. or "job stress".mp. or Job Satisfaction/ or "job satisfaction".mp. or moral injury.mp. or burn&out.mp.

3. exp medical personnel/ or medical personnel.mp. or physician$.mp. or allergist$.mp. or cardiologists.mp. or dermatologists.mp. or endocrinologists.mp. or gastroenterologists.mp. or general practitioner$.mp. or geriatrician$.mp. or nephrologist$.mp. or neurologist$.mp. or occupational health physician$.mp. or oncologist$.mp. or ophthalmologist$.mp. or pathologist$.mp. or p$ediatrician$.mp. or neonatologist$.mp. or physiatrist$.mp. or pulmonologist$.mp. or psychistrist$.mp. or radiologist$.mp. or rheumatologist$.mp. or surgeon$.mp. or urologist$.mp. or doctor*.mp. or medical staff.mp. or an$esthetist$.mp. or medical school/ or medical faculty.mp.

Filter, from:
https://www.cochranelibrary.com/cdsr/doi/10.1002/14651858.MR000041.pub2/appendices

epidemiology/ or exp clinical study/ or cohort analysis/ or case study/ or follow up/ or cross-sectional study/ or observational study/ or correlational study/ or epidemiologic.ab,ti. Or case control.ab,ti. Or case referent.ab,ti. Or case stud$.ab,ti. Or case series.ab,ti. Or cohort?.ab,ti. Or cross sectional.ab,ti. Or follow up.ab,ti. Or longitudinal.ab,ti. Or retrospective$.ab,ti. Or prospective$.ab,ti. Or observational.ab,ti. Or adverse effect?.ab,ti. or (Controlled before and after).ab,ti. Or Interrupted time series.ab,ti. or Correlational.ab,ti. Or ecological stud$.ab,ti. or Descriptive stud$.ab,ti.

Not Editorial or letter

Psych info(accessed via Ovid)::

1. exp spirituality/ or exp religion/ or exp religiosity/ or exp religious beliefs/ or religious experiences/ or exp soul/ or exp spiritual care/ or religio*.mp. or religiosity.mp. or exp philosophy/ or exp faith/ or theology/ or sikh.mp. or jain.mp. or bahai.mp. or pagan.mp. or shinto.mp. or cao dai.mp. or zoroast*.mp. or spirit*.mp. or Faith.mp. or holistic health.mp. or existential.mp. or salutogenesis.mp. or spiritual*.mp. or chaplain*.mp. or Buddhism.mp. or Christian*.mp. or Hinduism.mp. or islam.mp. or Judaism.mp. or humanism.mp. or Confucian*.mp. or exp philosophies/ or exp religious personnel/ or god concepts/ or theology/ or "sense of coherence".mp.
2. exp occupational stress/ or occupational neurosis/ or "quality of work life"/ or exp moral injury/ or job satisfaction/ or compassion fatigue.mp. or occupational stress.mp. or burnout.mp. or "Maslach Burnout Inventory".mp. or "work related stress*".mp. or "occupational stress*".mp. or "work stress*".mp. or "job stress".mp. or "job satisfaction".mp. or moral injury.mp. or burn&out.mp.
3. exp medical personnel/ or medical personnel.mp. or physician$.mp. or allergist$.mp. or cardiologists.mp. or dermatologists.mp. or endocrinologists.mp. or gastroenterologists.mp. or general practitioner$.mp. or geriatrician$.mp. or nephrologist$.mp. or neurologist$.mp. or occupational health physician$.mp. or oncologist$.mp. or ophthalmologist$.mp. or pathologist$.mp. or p$ediatrician$.mp. or neonatologist$.mp. or physiatrist$.mp. or pulmonologist$.mp. or psychistrist$.mp. or radiologist$.mp. or rheumatologist$.mp. or surgeon$.mp. or urologist$.mp. or doctor*.mp. or medical staff.mp. or an$esthetist$.mp. or medical faculty.mp.
4.
(epidemiologic or case control or case referent or case stud$ or case series or cohort? or cross sectional or follow up or longitudinal or retrospective$ or prospective$ or observational or adverse effect? or (Controlled before and after) or Interrupted time series or Correlational or ecological stud$ or Descriptive stud$).ab,ti. Or exp experimental design or epidemiology/

Medline(accessed via Ovid)::

1. exp philosophy/ or exp religion/ or exp "religion and medicine"/ or exp "religion and psychology"/ or "religion and science"/ or exp religious philosophies/ or exp theology/ or religio*.mp. or sikh.mp. or jain.mp. or bahai.mp. or Humanism.mp. or pagan.mp. or shinto.mp. or cao dai.mp. or zoroast*.mp. or spirit*.mp. or faith.mp. or exp holistic health/ or existential.mp. or salutogenesis.mp. or exp Spiritual Therapies/ or religious personnel/ or holistic health/ or "sense of coherence"/

2.

exp occupational stress/ or compassion fatigue.mp. or occupational stress.mp. or burnout.mp. or caregiver burnout/ or exp burnout, psychological/ or "Maslach Burnout Inventory".mp. or "work related stress*".mp. or exp Occupational Stress/ or "occupational stress*".mp. or "work stress*".mp. or "job stress".mp. or Job Satisfaction/ or "job satisfaction".mp. or moral injury.mp. or "work-life balance"/ or burn&out.mp.

3.

anesthetists/ or anesthesiologists/ or faculty, medical/ or exp medical staff/ or exp physicians/ or medical personnel.mp. or physician$.mp. or allergist$.mp. or cardiologists.mp. or dermatologists.mp. or endocrinologists.mp. or gastroenterologists.mp. or general practitioner$.mp. or geriatrician$.mp. or nephrologist$.mp. or neurologist$.mp. or occupational health physician$.mp. or oncologist$.mp. or ophthalmologist$.mp. or pathologist$.mp. or p$ediatrician$.mp. or neonatologist$.mp. or physiatrist$.mp. or pulmonologist$.mp. or psychistrist$.mp. or radiologist$.mp. or rheumatologist$.mp. or surgeon$.mp. or urologist$.mp. or doctor*.mp. or medical staff.mp. or an$esthetist$.mp. or medical faculty.mp.

4. Epidemiologic Studies/ or exp Case Control Studies/ or exp Cohort Studies/ or Cross-Sectional Studies/ or (epidemiologic adj (study or studies)).ab,ti. Or case control.ab,ti. Or (cohort adj (study or studies)).ab,ti. Or cross sectional.ab,ti. Or cohort analy$.ab,ti. or (follow up adj (study or studies)).ab,ti. Or longitudinal.ab,ti. Or retrospective$.ab,ti. Or prospective$.ab,ti. Or (observ$ adj3 (study or studies)).ab,ti. Or adverse effect?.ab,ti.

5. editorial or letter

Web of science accessed via Clarivate:

1. TS=(religio* or philosop* or spirit* or theolog* or sikh or jain or bahai or pagan or shinto or "cao dai" or zoroast* or Faith or "holistic health" or existential or salutogenesis or spiritual* or chaplain* or Buddh* or Christian* or Hindu* or islam* or Juda* or humanism or Confucian* or "sense of coherence")
2. TS=(burnout or "compassion fatigue" or "Maslach Burnout Inventory" or "work related stress*" or "occupational stress*" or "work stress*" or "job stress" or "job satisfaction" or "moral injury" or burn&out)

3. (TS=("medical personnel" or physician$ or allergist$ or cardiologist$ or dermatologist$ or endocrinologist$ or gastroenterologist$ or general practitioner$ or geriatrician$ or nephrologist$ or neurologist$ or occupational health physician$ or oncologist$ or ophthalmologist$ or pathologist$ or p$ediatrician* or neonatologist$ or physiatrist$ or pulmonologist$ or psychistrist$ or radiologist$ or rheumatologist$ or surgeon$ or urologist$ or doctor* or "medical staff" or an$esthetist* or "medical school" or "medical faculty")
4. AND DOCUMENT TYPES: (Article OR Abstract of Published Item OR Correction OR Correction, Addition OR Data Paper OR Database Review OR Discussion OR Early Access OR Meeting Abstract OR Meeting Summary OR News Item OR Note OR Proceedings Paper OR Record Review OR Review OR Script)

Scopus accessed via https://www.scopus.com/home.uri:

1. Title-abs-key(religio* or philosop* or spirit* or theolog* or sikh or jain or bahai or pagan or shinto or "cao dai" or zoroast* or Faith or "holistic health" or existential or salutogenesis or spiritual* or chaplain* or Buddh* or Christian* or Hindu* or islam* or Juda* or humanism or Confucian* or "sense of coherence")
2. Title-abs-key(burnout or "compassion fatigue" or "Maslach Burnout Inventory" or "work related stress*" or "occupational stress*" or "work stress*" or "job stress" or "job satisfaction" or "moral injury" or burn&out)
3. Title-abs-key("medical personnel" or physician$ or allergist$ or cardiologist$ or dermatologist$ or endocrinologist$ or gastroenterologist$ or general practitioner$ or geriatrician$ or nephrologist$ or neurologist$ or occupational health physician$ or oncologist$ or ophthalmologist$ or pathologist$ or p$ediatrician* or neonatologist$ or physiatrist$ or pulmonologist$ or psychistrist$ or radiologist$ or rheumatologist$ or surgeon$ or urologist$ or doctor* or "medical staff" or an$esthetist* or "medical school" or "medical faculty")

All searches concluded by 8th March 2022

Systematic Review Protocol & Support Template

**Title of the review**	A systematic review: Is there a relationship between burnout scores in doctors and their spiritual health
**First reviewer**	Orla Whitehead
**Team of reviewers**	Ishbel Orla Whitehead, Suzanne Moffatt, Stephanie Warwick, Gemma Frances Spiersa, Tafadzwa Patience Kunonga, Eugene Yee Hing Tang, Barbara Hanratty
**Supervisor/Project PI**	Professor Barbara Hanratty
**Clinical Portfolio Group**	
**Project title (if different from review title)**	

**Support** – please state if advice/training or personnel required at each stage	
**SR overview**	
**Protocol development**	“”
**Literature searching**	Advice taken from Catherine Richmond, information scientist at the University
**Quality appraisal**	
**Data Extraction**	“”
**Synthesis**	“”
**Writing up**	“”

For peer review only

1. Background to review

Brief introduction to the subject of the review, including rationale for undertaking the review and overall aim

Burnout and moral injury are two terms used to describe a form of psychological distress associated with work as a doctor. Burnout is defined by the World Health Organisation not as an illness, but as an occupational phenomenon, resulting in exhaustion, mental distance or feelings of negativity or cynicism towards the job, and reduced professional efficacy.[1] Others describe a loss of meaning in work, and objectification of patients and their families, rather than engaging with their humanity.[2] Moral injury is a term taken from military personnel returning from battle, where the realties conflict with our moral and ethical code.[3] It has been argued that doctors caught between patient and societal demands, limited resources, and their own ethical code and training, are suffering injury analogous to soldiers, leaving them wounded and hopeless.[3] Reframing burnout as moral injury replaces a view of a defective individual, to that of a defective system, which could be more effective in terms of change[4], and move the dialogue away from expecting doctors to become more and more resilient in the face of increasing challenges. The co-option of the term from conflict has been critised[5], and arguments made that while moral injury can occur in medicine, burn out is not synonymous, and is more common.[6] While both the terms burnout and moral injury place the issue in the context of occupation, it is evident that mental illness accompanies these issues, as suicide rates for female doctors are up to 4 times those of age-matched controls.[7] With current recruitment and retention issues, we cannot afford to lose good doctors to burnout.[2] Supporting doctors' holistic health has to become a priority, to help prevent and treat burnout and moral injury.

Holistic health includes spiritual health. Literature discussing how doctors should address their patients' spiritual health often mention that self awareness of spiritual health, needs, and distress, is key to be able to begin to meet patients' needs in this area. In a recent survey of GPs in England, 69% described themselves as 'a spiritual person', with 48% also describing themselves as 'a religious person'. These GPs defined spiritual health as self-actualisation and meaning, transcendence and relationships beyond the self, and expressions of spirituality. Self-actualisation included concepts of being able to follow their personal ethical and moral codes. Meaning included having personal meaning to life and relationships. Transcendence included concepts of a spiritual sphere, a concept of a soul, and relationships with communities, friends, family, nature and/or the divine. Expressions of spirituality included both personal and group religious and spiritual practice, for example attendance at church, religious meetings, meditation, prayer and mindfulness practice. Burnout could be seen as contrasting with these definitions of spiritual health, as mental distance from others and the job, negativity and lack of meaning in the job, and lack of relationships with patients and their families. Spiritual health as defined by GPs requires meaning, following their ethical code. Could burnout and spiritual distress among doctors be related phenomena? Viewing burnout through the lens of spiritual health and distress, as well as other lenses and perspectives, could generate potential beneficial interventions for both prevention and treatment of burnout.

Aims:

To investigate the association between burnout in doctors and spiritual health, by conducting a systematic review of studies which use a measure burnout with measures of aspects of spiritual health, spirituality, religiosity or spiritual distress.

For peer review only

For peer review only - http://bmjopen.bmj.com/site/about/guidelines.xhtml

2. Specific objectives
1. To investigate the quantity and quality of data addressing doctors' burnout, and spiritual heath<br>2. To investigate any link between doctors' burnout and spiritual health, spirituality, spiritual distress or religiosity.

For peer review only

**3. a) Criteria for including studies in the review**<br>If the PICOS format does not fit the research question of interest, please split up the question into separate concepts and put one under each heading	
**i. Population, or participants and conditions of interest**	Qualified doctors/medical practitioners, worldwide of either sex and any age, whether they have experienced any level of burnout or not.
**ii. Interventions or exposures**	Quantification of burnout (for example, use of a burnout inventory such as the Maslach Burnout Inventory, or the Copenhagen Burnout Inventory, or others)
**iii. Comparisons or control groups**	Quantification of spiritual health, spirituality, spiritual distress, or religiosity, either using a scale (for example the Spiritual Wellbeing Scale, Spiritual Distress Scale, or other measures of spiritual health or religiosity) or a yes/no or self-rating of 'spirituality' or religiosity.
**iv. Outcomes of interest**	Associations between spiritual health, spiritual distress or religiosity with burnout scores.
**v. Setting**	Any setting where doctors/medical practitioners work (primary, secondary or tertiary care, including laboratory or other non-patient facing settings)
**vi. Study designs**	All research studies with a quantification of burnout, and spiritual health, wellbeing, distress or religiosity, or similar.

3. b) Criteria for excluding studies not covered in inclusion criteria
Any specific populations excluded, date range, language, whether abstracts or full text available, etc

Medical students, nursing staff, or other hospital staff where doctors are not included.
Course evaluations
Editorials, book chapters, or other non-research study articles
No specific mention of spiritual health, spirituality, or religiosity.
No quantification of burnout, or spiritual health, wellbeing or distress, or similar.

For peer review only - http://bmjopen.bmj.com/site/about/guidelines.xhtml

4. Search methods	
**Electronic databases** Please list all databases that are to be searched and include the interface (eg NHS, EBSCO, etc) and date ranges searched for each	PUBMED/MEDLINE<br>EMBASE<br>PsychInfo<br>Web of Science<br>Scopus
**Other methods used for identifying relevant research** ie contacting experts and reference checking	Reference checking and hand searching of these. Contacting experts in this field.
**Journals hand searched** If any are to be hand searched, please list which journals and date searched from, including a rationale.	

5. Methods of review	
**Details of methods** Number of reviewers, how agreements to be reached and disagreements dealt with, etc.	Data extraction will be carried out by the primary reviewer, with 10% checked by a second reviewer
**Quality assessment** Tools or checklists used with references or URLs	Appraisal of Cross-sectional Studies tool (AXIS).[8]

For peer review only

**Data extraction** What information is to be collected on each included study. If databases or forms on Word or Excel are used and how this is recorded and by how many reviewers	Custom made word table to be used
**Narrative synthesis** Details of what and how synthesis will be done	Synthesis of findings regarding the research question (are burnout and spiritual health related)<br>Synthesis of measures used for burnout, and spiritual health
**Meta-analysis** Details of what and how analysis and testing will be done. If no meta-analysis is to be conducted, please give reason.	If possible, meta-analysis will be conducted of the relationship between burnout scores (e.g. the Maslach Burnout Inventory) and spiritual health scores (e.g. the Brief COPE). If different validated scores are used, effect sizes will be compared.
**Grading evidence** System used, if any, such as GRADE	

**6. Presentation of results**	
**Additional material** Summary tables, flowcharts, etc, to be included in the final paper	
**Outputs from review** Papers and target journals, conference presentations, reports, etc	Conference presentation at the Society for Academic Primary Care Annual Scientific Meeting<br>Submission to journal

For peer review only

For peer review only - http://bmjopen.bmj.com/site/about/guidelines.xhtml

7. Timeline for review – when do you aim to complete each stage of the review	
**Protocol**	July 2020
**Literature searching**	July 2020
**Quality appraisal**	August 2020
**Data extraction**	September 2020
**Synthesis**	September 2020
**Writing up**	October 2020

1. World Heath Organisation. Burn-out an "occupational phenomenon": International Classification of Diseases 2019 [Available from: https://www.who.int/mental_health/evidence/burn-out/en/.
2. Fred HL, Scheid MS. Physician Burnout: Causes, Consequences, and (?) Cures. *Tex Heart Inst J* 2018;45(4):198-202. doi: 10.14503/THIJ-18-6842
3. Talbot SG, Dean W. Physicians aren't 'burning out.'They're suffering from moral injury. *Stat* 2018
4. Dean W, Talbot S, Dean A. Reframing Clinician Distress: Moral Injury Not Burnout. *Fed Pract* 2019;36(9):400-02.
5. Asken MJ. Physician burnout: moral injury is a questionable term. *BMJ* 2019;365:l2375. doi: 10.1136/bmj.l2375
6. Parker J. Moral injury and burnout are not the same 2019 [Available from: https://blogs.bmj.com/bmj/2019/06/06/joshua-parker-moral-injury-and-burnout-are-not-the-same/ accessed 11/12/2019.
7. Practitioner Health Programme. Suicide in doctors and other health professionals 2019 [Available from: https://php.nhs.uk/resources/suicide-in-doctors/ accessed 11/12/2019.
8. Downes MJ, Brennan ML, Williams HC, et al. Development of a critical appraisal tool to assess the quality of cross-sectional studies (AXIS). *BMJ Open* 2016;6(12):e011458. doi: 10.1136/bmjopen-2016-011458

For peer review only

PRISMA 2020 Checklist

Section and Topic	Item #	Checklist item	Location where item is reported
**TITLE**			
Title	1	Identify the report as a systematic review.	p1
**ABSTRACT**			
Abstract	2	See the PRISMA 2020 for Abstracts checklist.	attached
**INTRODUCTION**			
Rationale	3	Describe the rationale for the review in the context of existing knowledge.	p3-5
Objectives	4	Provide an explicit statement of the objective(s) or question(s) the review addresses.	lines 98-102
**METHODS**			
Eligibility criteria	5	Specify the inclusion and exclusion criteria for the review and how studies were grouped for the syntheses.	lines 115-132
Information sources	6	Specify all databases, registers, websites, organisations, reference lists and other sources searched or consulted to identify studies. Specify the date when each source was last searched or consulted.	p5-7 and attached search strategy
Search strategy	7	Present the full search strategies for all databases, registers and websites, including any filters and limits used.	search strategy and lines 104-132
Selection process	8	Specify the methods used to decide whether a study met the inclusion criteria of the review, including how many reviewers screened each record and each report retrieved, whether they worked independently, and if applicable, details of automation tools used in the process.	lines 136-150
Data collection process	9	Specify the methods used to collect data from reports, including how many reviewers collected data from each report, whether they worked independently, any processes for obtaining or confirming data from study investigators, and if applicable, details of automation tools used in the process.	lines 136-150
Data items	10a	List and define all outcomes for which data were sought. Specify whether all results that were compatible with each outcome domain in each study were sought (e.g. for all measures, time points, analyses), and if not, the methods used to decide which results to collect.	lines 123-126
	10b	List and define all other variables for which data were sought (e.g. participant and intervention characteristics, funding sources). Describe any assumptions made about any missing or unclear information.	lines 115-126
Study risk of bias assessment	11	Specify the methods used to assess risk of bias in the included studies, including details of the tool(s) used, how many reviewers assessed each study and whether they worked independently, and if applicable, details of automation tools used in the process.	line 142-143
Effect measures	12	Specify for each outcome the effect measure(s) (e.g. risk ratio, mean difference) used in the synthesis or presentation of results.	line 148
Synthesis methods	13a	Describe the processes used to decide which studies were eligible for each synthesis (e.g. tabulating the study intervention characteristics and comparing against the planned groups for each synthesis (item #5)).	line 148-150
	13b	Describe any methods required to prepare the data for presentation or synthesis, such as handling of missing summary statistics, or data conversions.	n/a
	13c	Describe any methods used to tabulate or visually display results of individual studies and syntheses.	line 148-150
	13d	Describe any methods used to synthesize results and provide a rationale for the choice(s). If meta-analysis was performed, describe the model(s), method(s) to identify the presence and extent of statistical heterogeneity, and software package(s) used.	lines 148-150, meta

For peer review only

For peer review only - http://bmjopen.bmj.com/site/about/guidelines.xhtml

PRISMA 2020 Checklist

Section and Topic	Item #	Checklist item	Location where item is reported
			analysis not performed
	13e	Describe any methods used to explore possible causes of heterogeneity among study results (e.g. subgroup analysis, meta-regression).	n/a
	13f	Describe any sensitivity analyses conducted to assess robustness of the synthesized results.	n/a
Reporting bias assessment	14	Describe any methods used to assess risk of bias due to missing results in a synthesis (arising from reporting biases).	n/a
Certainty assessment	15	Describe any methods used to assess certainty (or confidence) in the body of evidence for an outcome.	n/a
**RESULTS**			
Study selection	16a	Describe the results of the search and selection process, from the number of records identified in the search to the number of studies included in the review, ideally using a flow diagram.	prisma diagram
	16b	Cite studies that might appear to meet the inclusion criteria, but which were excluded, and explain why they were excluded.	n/a
Study characteristics	17	Cite each included study and present its characteristics.	table 2
Risk of bias in studies	18	Present assessments of risk of bias for each included study.	table 2
Results of individual studies	19	For all outcomes, present, for each study: (a) summary statistics for each group (where appropriate) and (b) an effect estimate and its precision (e.g. confidence/credible interval), ideally using structured tables or plots.	table 1
Results of syntheses	20a	For each synthesis, briefly summarise the characteristics and risk of bias among contributing studies.	table 2 and p7-111
	20b	Present results of all statistical syntheses conducted. If meta-analysis was done, present for each the summary estimate and its precision (e.g. confidence/credible interval) and measures of statistical heterogeneity. If comparing groups, describe the direction of the effect.	table 1 and p7-11
	20c	Present results of all investigations of possible causes of heterogeneity among study results.	p9
	20d	Present results of all sensitivity analyses conducted to assess the robustness of the synthesized results.	n/a
Reporting biases	21	Present assessments of risk of bias due to missing results (arising from reporting biases) for each synthesis assessed.	p9-10
Certainty of evidence	22	Present assessments of certainty (or confidence) in the body of evidence for each outcome assessed.<br>• Report the overall level of certainty (or confidence) in the body of evidence for each important outcome.<br>• Provide an explanation of reasons for rating down (or rating up) the certainty of evidence (e.g. in footnotes to an evidence summary table).<br>• Communicate certainty in the evidence wherever results are reported (i.e. abstract, evidence summary tables, results, conclusions), using a format appropriate for the section of the review.	p8-23<br>p24<br>p10<br>throughout
**DISCUSSION**			
Discussion	23a	Provide a general interpretation of the results in the context of other evidence.	p11-12
	23b	Discuss any limitations of the evidence included in the review.	lines 323-52
	23c	Discuss any limitations of the review processes used.	lines 354-365

For peer review only

For peer review only - http://bmjopen.bmj.com/site/about/guidelines.xhtml

PRISMA 2020 Checklist

Section and Topic	Item #	Checklist item	Location where item is reported
	23d	Discuss implications of the results for practice, policy, and future research.	lines 367-375
**OTHER INFORMATION**			
Registration and protocol	24a	Provide registration information for the review, including register name and registration number, or state that the review was not registered.	p1
	24b	Indicate where the review protocol can be accessed, or state that a protocol was not prepared.	p1
	24c	Describe and explain any amendments to information provided at registration or in the protocol.	n/a
Support	25	Describe sources of financial or non-financial support for the review, and the role of the funders or sponsors in the review.	p1
Competing interests	26	Declare any competing interests of review authors.	p1
Availability of data, code and other materials	27	Report which of the following are publicly available and where they can be found: template data collection forms; data extracted from included studies; data used for all analyses; analytic code; any other materials used in the review.	p1

From: Page MJ, McKenzie JE, Bossuyt PM, Boutron I, Hoffmann TC, Mulrow CD, et al. The PRISMA 2020 statement: an updated guideline for reporting systematic reviews. BMJ 2021;372:n71. doi: 10.1136/bmj.n71

For more information, visit: http://www.prisma-statement.org/

For peer review only

PRISMA-S Checklist

Section/topic		Checklist item	Location(s) Reported
INFORMATION SOURCES AND METHODS			
Database name	1	Name each individual database searched, stating the platform for each.	search file
Multi-database searching	2	If databases were searched simultaneously on a single platform, state the name of the platform, listing all of the databases searched.	n/a
Study registries	3	List any study registries searched.	none
Online resources and browsing	4	Describe any online or print source purposefully searched or browsed (e.g., tables of contents, print conference proceedings, web sites), and how this was done.	none
Citation searching	5	Indicate whether cited references or citing references were examined, and describe any methods used for locating cited/citing references (e.g., browsing reference lists, using a citation index, setting up email alerts for references citing included studies).	main document methods
Contacts	6	Indicate whether additional studies or data were sought by contacting authors, experts, manufacturers, or others.	main document methods
Other methods	7	Describe any additional information sources or search methods used.	none
SEARCH STRATEGIES			
Full search strategies	8	Include the search strategies for each database and information source, copied and pasted exactly as run.	search file
Limits and restrictions	9	Specify that no limits were used, or describe any limits or restrictions applied to a search (e.g., date or time period, language, study design) and provide justification for their use.	search file and main document
Search filters	10	Indicate whether published search filters were used (as originally designed or modified), and if so, cite the filter(s) used.	search file and main document methods
Prior work	11	Indicate when search strategies from other literature reviews were adapted or reused for a substantive part or all of the search, citing the previous review(s).	n/a
Updates	12	Report the methods used to update the search(es) (e.g., rerunning searches, email alerts).	main document methods
Dates of searches	13	For each search strategy, provide the date when the last search occurred.	main document methods and search document

For peer review only

PEER REVIEW			
Peer review	14	Describe any search peer review process.	Main document methods
MANAGING RECORDS			
Total Records	15	Document the total number of records identified from each database and other information sources.	Prisma diagram
Deduplication	16	Describe the processes and any software used to deduplicate records from multiple database searches and other information sources.	Main document methods

PRISMA-S: An Extension to the PRISMA Statement for Reporting Literature Searches in Systematic Reviews Rethlefsen ML, Kirtley S, Waffenschmidt S, Ayala AP, Moher D, Page M], Koffel JB, PRISMA-S Group. Last updated February 27, 2020.

For peer review only

PRISMA 2020 for Abstracts Checklist

Section and Topic	Item #	Checklist item	Reported (Yes/No)
**TITLE**			
Title	1	Identify the report as a systematic review.	yes
**BACKGROUND**			
Objectives	2	Provide an explicit statement of the main objective(s) or question(s) the review addresses.	yes
**METHODS**			
Eligibility criteria	3	Specify the inclusion and exclusion criteria for the review.	yes
Information sources	4	Specify the information sources (e.g. databases, registers) used to identify studies and the date when each was last searched.	yes
Risk of bias	5	Specify the methods used to assess risk of bias in the included studies.	yes
Synthesis of results	6	Specify the methods used to present and synthesise results.	yes
**RESULTS**			
Included studies	7	Give the total number of included studies and participants and summarise relevant characteristics of studies.	yes
Synthesis of results	8	Present results for main outcomes, preferably indicating the number of included studies and participants for each. If meta-analysis was done, report the summary estimate and confidence/credible interval. If comparing groups, indicate the direction of the effect (i.e. which group is favoured).	yes
**DISCUSSION**			
Limitations of evidence	9	Provide a brief summary of the limitations of the evidence included in the review (e.g. study risk of bias, inconsistency and imprecision).	yes
Interpretation	10	Provide a general interpretation of the results and important implications.	yes
**OTHER**			
Funding	11	Specify the primary source of funding for the review.	yes
Registration	12	Provide the register name and registration number.	yes

For peer review only

From: Page MJ, McKenzie JE, Bossuyt PM, Boutron I, Hoffmann TC, Mulrow CD, et al. The PRISMA 2020 statement: an updated guideline for reporting systematic reviews. BMJ 2021;372:n71. doi: 10.1136/bmj.n71

For more information, visit: http://www.prisma-statement.org/

For peer review only
